# Straightforward and Efficient Protocol for the Synthesis of Pyrazolo [4,3-*b*]pyridines and Indazoles

**DOI:** 10.3390/ijms24021758

**Published:** 2023-01-16

**Authors:** Vladislav V. Nikol’skiy, Mikhail E. Minyaev, Maxim A. Bastrakov, Alexey M. Starosotnikov

**Affiliations:** N.D. Zelinsky Institute of Organic Chemistry RAS, Leninsky Prosp. 47, 119991 Moscow, Russia

**Keywords:** pyrazolo [4,3-*b*]pyridines, indazoles, Japp–Klingemann reaction, nucleophilic substitution, aromatic nitro compounds

## Abstract

An efficient method for the synthesis of pyrazolo [4,3-*b*]pyridines has been developed on the basis of readily available 2-chloro-3-nitropyridines via a sequence of SNAr and modified Japp–Klingemann reactions. The method offers a number of advantages including utilization of stable arenediazonium tosylates, operational simplicity as well as combining the azo-coupling, deacylation and pyrazole ring annulation steps in a one-pot manner. An unusual rearrangement (C-N-migration of the acetyl group) was observed and a plausible mechanism was proposed based on the isolated intermediates and NMR experiments. In addition, the developed protocol was successfully applied to the synthesis of 1-arylindazoles combining the Japp–Klingemann reaction and cyclization of the resulting hydrazone as a one-pot procedure.

## 1. Introduction

**Figure 1:**
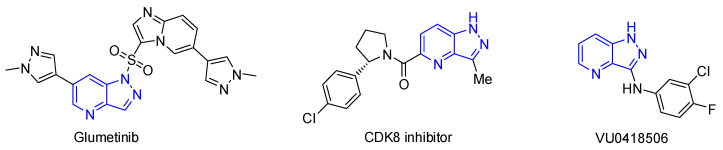
Biologically active pyrazolo [4,3-*b*]pyridines.

Polynitrogen heterocycles have found their application in various fields of chemistry and material science, such as perspective high-energy density compounds [1,2,3,4], purine antimetabolites used in the treatment of cancer [5], chemosensors [6] and many others. In particular, pyrazolo [4,3-*b*]pyridines are of considerable interest due to their diverse biological activity. They have been studied as potential HIV-1 non-nucleoside reverse transcriptase inhibitors [7], interleukin-2 inducible T-cell kinase inhibitors [8], transient receptor potential ankyrin-repeat 1 (TRK1) antagonists [9] and corticotropin-releasing factor receptor type-1 (CRF1) antagonists [10]. Some biologically active pyrazolo [4,3-*b*]pyridines are shown on Figure 1. In particular, Glumetinib—a highly selective inhibitor of the oncoprotein c-Met (hepatocyte growth factor receptor; HGFR) with potential antineoplastic activity [11]—as well as CDK8 inhibitor [12] and VU0418506, a positive allosteric modulator (PAM) of the metabotropic glutamate receptor 4 (mGlu4) [13].

**Scheme 1:**
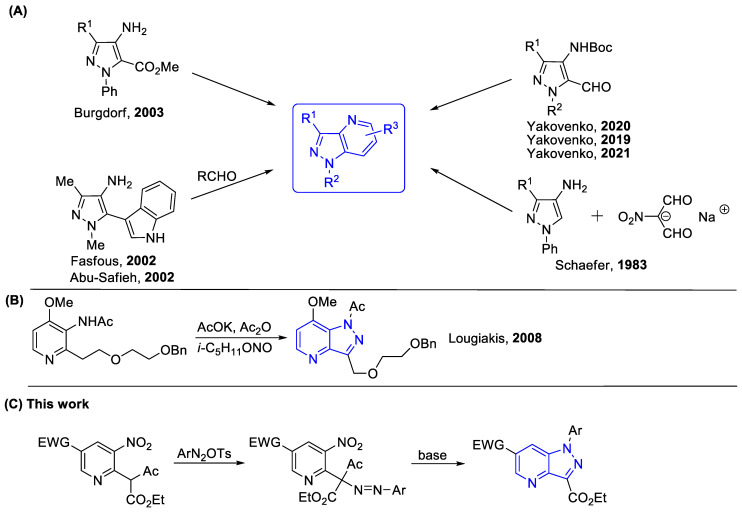
Methods for the synthesis of pyrazolo [4,3-*b*]pyridines: (**A**) annulation of a pyridine fragment to an amino substituted pyrazole ring [14,15,16,17,18,19,20]; (**B**) annulation of a pyrazole ring to functionalized pyridine core [22]; (**C**) synthesis from available 3-nitropyridines.

Methods for the synthesis of pyrazolo [4,3-*b*]pyridines are usually based on the annulation of a pyridine fragment to an amino-substituted pyrazole ring, Scheme 1A. Two main approaches include cyclocondensation of unstable 4-aminopyrazole-5-carbaldehydes in their N-protected forms [14,15,16], as well as cyclization of 5-functionalized 4-aminopyrazoles [17]. Another example deals with cyclization of 4-amino-5-(indol-3-yl)pyrazole with aldehydes [18,19] or sodium nitromalonodialdehyde [20]. Some additional methods for the conversion of 4-aminopyrazoles to pyrazolo [4,3-*b*]pyridines are summarized in review [21]. Otherwise, pyrazolo [4,3-*b*]pyridines can be obtained via annulation of a pyrazole ring to a functionalized pyridine core, though such reactions are quite rare [22], Scheme 1B.

In this work we propose an efficient method for the synthesis of pyrazolo [4,3-*b*]pyridines containing electron-withdrawing groups (EWG) starting from readily available 3-nitropyridines, Scheme 1C.

## 2. Results and Discussion

**Scheme 2:**
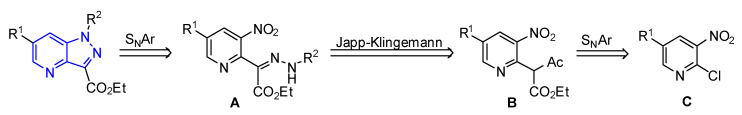
Retrosynthesis of pyrazolo [4,3-*b*]pyridines.

We proposed a retrosynthetic scheme for the synthesis of pyrazolo [4,3-*b*]pyridines, Scheme 2. The target molecules were planned to be synthesized via intramolecular nucleophilic substitution of the nitro group (S_N_Ar) with anions of hydrazones **A** which in turn could be prepared using the Japp–Klingemann reaction. The starting compounds—readily available 2-chloro-3-nitropyridines **C**—can be converted to pyridinyl keto esters **B** through a conventional S_N_Ar process. Such a synthetic route seemed reasonable because earlier we found that 3-NO_2_ in pyridines was prone to substitution under the action of anionic O-, N- and S-nucleophiles [23].

**Scheme 3:**
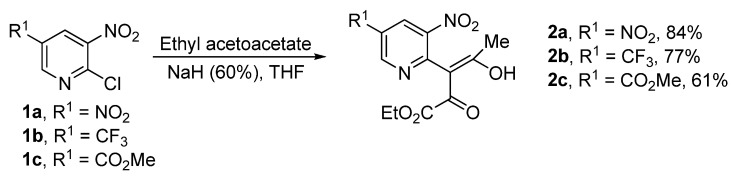
Synthesis of pyridin-2-yl keto esters **2a–c**.

2-Chloro-3-nitropyridines **1a–c** reacted with ethyl acetoacetate in the presence of NaH to give pyridinyl keto esters **2a–c** which exist in solutions as a mixture of ketone and enol tautomers with the enol form being predominant, Scheme 3. These results are in accordance with the previously reported [24].

**Figure 2:**
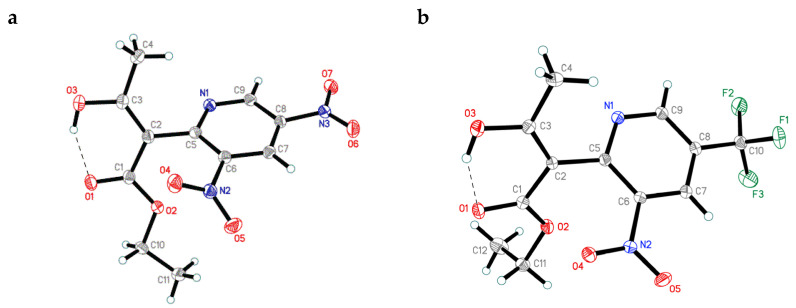
X-ray crystal structures of compounds (**a**) **2a** (only one crystallographically unique molecule is shown) and (**b**) **2b** with thermal ellipsoids set at a 50% probability level.

Structures of compounds **2a–c** were confirmed by NMR and HRMS data. Crystal structures of **2a** and **2b** were determined by the X-ray diffraction analysis, Figure 2. The structure of **2a** contained two crystallographically non-equivalent molecules.

**Scheme 4:**
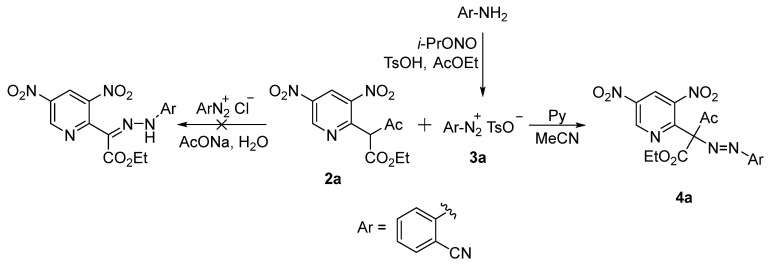
Reactions of keto ester **2a** with arenediazonium salts.

In the next step compound **2a** was selected for screening the reaction conditions. Conventional Japp–Klingemann reaction conditions [25] were found to be unsuitable in our case: reactions of **2a** with solutions of arenediazonium chlorides in the presence of sodium acetate yielded relatively stable azo-compounds **4** instead of desired hydrazones. Increasing temperature or pH only resulted in numerous side products. This prompted us to thoroughly study the azo-coupling and subsequent steps leading to target pyrazolo [4,3-*b*]pyridines. First of all, arenediazonium tosylate **3a** was prepared by a recently reported method [26] and used as an equivalent of arenediazonium chloride. These compounds are more stable than corresponding chlorides towards explosion or chemical decomposition. Arenediazonium tosylates **3** can be cleanly synthesized by diazotization of anilines in the presence of *p*-toluenesulfonic acid [27,28] and used without further purification, Scheme 4. Reaction of salt **3a** with pyridinyl keto ester **2a** under non-aqueous conditions in the presence of pyridine afforded azo-compound **4a** in quantitative yield. This model compound was used for the screening of various reagents that can affect deacylation and subsequent cyclization.

**Figure 3:**
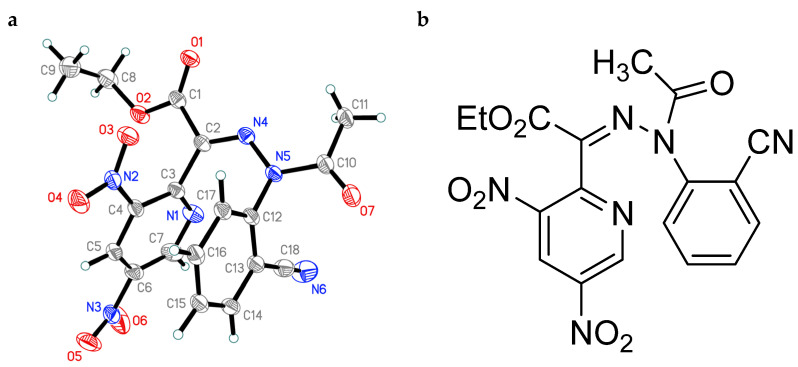
(**a**) X-ray crystal structure of compound **5a′** with thermal ellipsoids at a 50% probability level. Disorder of one NO_2_ group is omitted, (**b**) structural formula of **5a′**.

Treatment of **4a** with non-nucleophilic K_2_CO_3_ resulted in decomposition signifying the importance of nucleophiles for the deacetylation step. Both NaOH and MeONa were able to yield pyrazolo [4,3-*b*]pyridine product although a notable side reaction with an ester group makes those reagents impractical. Milder nucleophilic bases, such as DABCO and secondary amines, all reacted cleanly to give the expected ethyl 1-(2-cyanophenyl)-6-nitro-1H-pyrazolo [4,3-*b*]pyridine-3-carboxylate **5a** along with varying amounts of the unknown compound **5a′**. This compound was isolated and fully characterized by various methods including X-ray diffraction analysis (Figure 3). It turned out to be N-aryl-N-acetylhydrazone arising from C-N migration of the acetyl group.

**Figure 4:**
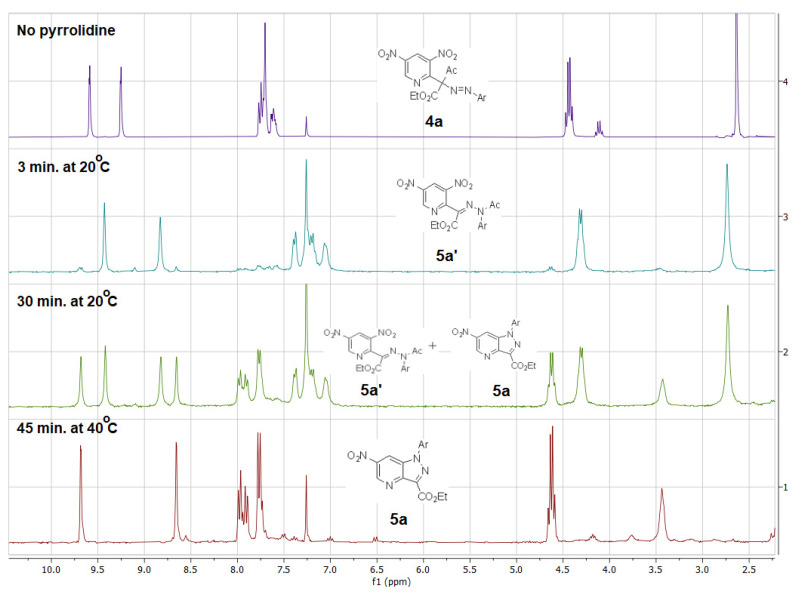
NMR spectra of the reaction mixture aliquots, collected and quenched before addition of pyrrolidine (violet) and then 3 (aquamarine), 30 (green) and 45 min (red) after addition.

To elucidate the role of compound **5a′** in the course of the reaction we performed a controlled experiment with a pure sample of **4a** and collected aliquots of the reaction mixture after 3, 30 and 45 min. Collected aliquots were immediately quenched with aqueous HCl, extracted and analyzed (Figure 4). ^1^H NMR spectra showed complete conversion of **4a** to **5a′** after 3 min with traces of **5a** present. Samples taken after 30 min at 20 °C contained almost equimolar mixture of **5a′** and **5a** and an additional 15 min at 40 °C yielded sample with mostly pure **5a**. This experiment allowed us to conclude that N-acetyl hydrazone **5a′** is in fact an intermediate and can be converted to the target pyrazolo [4,3-*b*]pyridine under appropriate conditions. Therefore, our final protocol for the synthesis of pyrazolo [4,3-*b*]pyridines **5** consists of azo-coupling in the presence of pyridine and subsequent one-pot cyclization by addition of pyrrolidine at 40 °C. Pyrrolidine has a favorable combination of nucleophilicity, basicity, ease of removal and affordability, thus making it the reagent of choice.

**Scheme 5:**
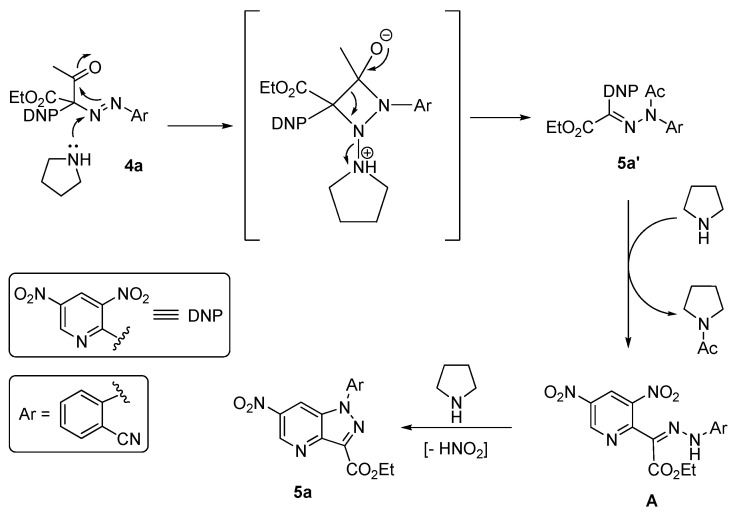
Plausible mechanism of the reaction of **4a** with pyrrolidine.

To the best of our knowledge such a rearrangement has not been previously described, although we were able to find a few articles about similar-looking C-N acyl migration in benzeneazotribenzoylmethanes (ArCO)_3_C-N=NAr [29,30]. This rearrangement requires prolonged heating above 130 °C and the mechanism is believed to be heterolytic cleavage and recombination. In contrast, rearrangement in our case occurs almost instantaneously at room temperature after the addition of suitable nucleophile. We propose the following mechanism which explains the role of a nucleophilic catalyst (Scheme 5). It starts with the attack of a nucleophile on an electron-deficient N=N double bond, thus creating a negative charge on the second nitrogen atom. In turn, this atom attacks the spatially close carbonyl group with the formation of a 4-membered cycle (which is drawn to illustrate a stepwise mechanism; however, a more concerted process can take place as well) which immediately opens to yield the product of acetyl migration. Significant decrease of steric strain around the quaternary carbon atom along with formation of a stable hydrazone fragment could be considered as the main driving forces. Formation of N-acetylpyrrolidine as a by-product can also be seen on Figure 3 (peak at 3.4 ppm). Absence of this compound in the reaction mixture directly after rearrangement also points towards intramolecular reaction because the formation of N-acetylpyrrolidine is virtually irreversible under reaction conditions.

**Scheme 6:**
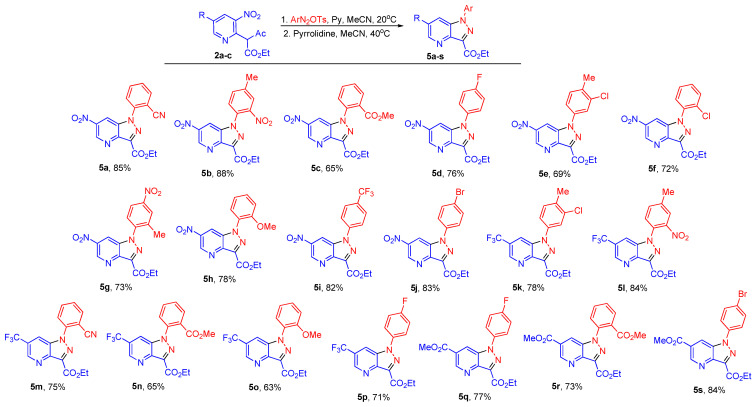
One-pot synthesis of pyrazolo [4,3-*b*]pyridines **5** (substrate scope).

The scope and limitations of the developed one-pot method was studied using compounds **2a–c** and various arenediazonium tosylates, Scheme 6. Generally, reactions proceeded smoothly in the case of both electron-withdrawing and electron-releasing groups in the aryl substituent, resulting in the formation of pyrazolo [4,3-*b*]pyridines **5a–s** in moderate to high yields.

**Figure 5:**
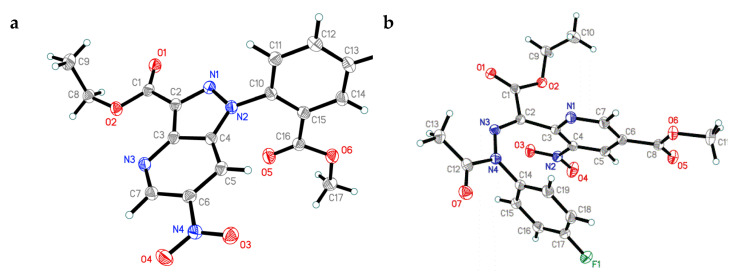
X-ray crystal structures of compounds (**a**) **5c** and (**b**) the intermediate **5q′** (only one of two crystallographically unique molecules is shown) with thermal ellipsoids at 50% probability level.

In all cases, formation of the intermediates of type **5a′** was observed by TLC, N-acetyl-N-arylhydrazone **5q′** was isolated and characterized by the spectral methods and X-ray analysis, Figure 5. Structures of compounds **5** were confirmed by NMR and HRMS, as well as by X-ray analysis for compound **5c**, Figure 5.

**Scheme 7:**
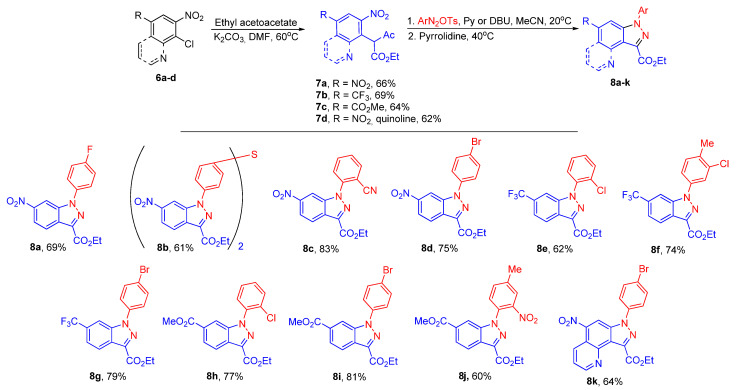
One-pot synthesis of indazoles **8** (substrate scope).

The developed method of pyrazole ring annulation can be applied to the synthesis of indazoles from nitrobenzene derivatives. However, in the case of nitrophenyl chlorides, which are less active compared to the corresponding chloronitropyridines, reaction conditions were changed in order to reach higher yields. Thus, 4-R-1-chloro-2-nitrobenzenes **6a–d** reacted with ethyl acetoacetate in the presence of K_2_CO_3_ in DMF at 60^o^C to give phenyl keto esters **7a–d**, Scheme 7. Compounds **7** were reacted with arenedizonium tosylates **3** under conditions elaborated for pyridyl acetoacetic esters **2a–d** in a one-pot manner, Scheme 7. DBU was used as a base in case of compounds **7b–d** since cyclization was found to proceed slowly in the presence of pyrrolidine. As a result, indazoles **8a–k** were obtained in 60–83% yields.

**Scheme 8:**
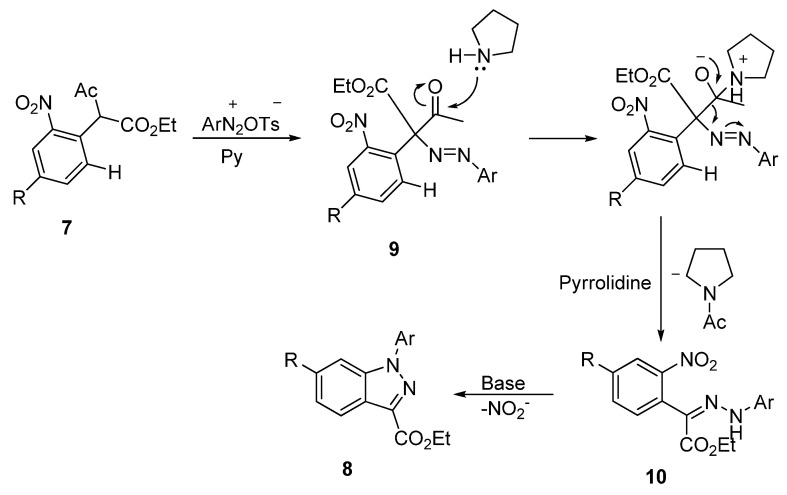
Plausible mechanism for the formation of indazole derivatives **8**.

TLC and NMR monitoring of the reaction mixtures did not reveal formation of the intermediate N-acetyl-N-arylhydrazones, indeed, azocompounds **9** formed as a result of azo-coupling were being converted directly to hydrazones **10** which were further cyclized under the action of a base. This allowed us to propose that reactions of benzene derivatives **7** proceed according to a conventional Japp–Klingemann mechanism, Scheme 8. Hydrazone **10e** was isolated and characterized to confirm the reaction scheme.

**Figure 6:**
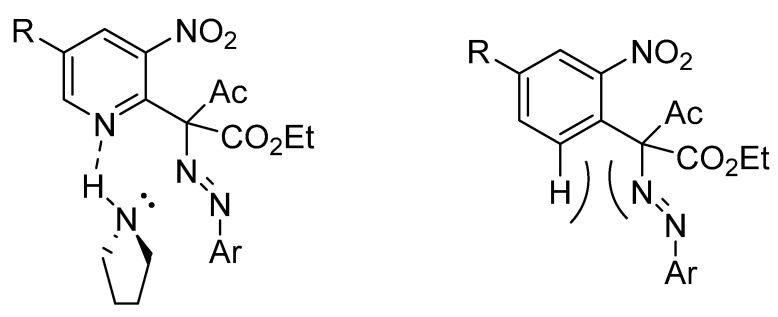
Possible factors affecting reaction pathway.

This consistent difference in reaction pathway between pyridine and benzene derivatives does not seem to be affected by substituents, therefore the heterocyclic nitrogen atom can be considered as the main reason for the unusual reactivity. One possible explanation is hydrogen bonding between this nitrogen atom and the attacking nucleophile which would coordinate it towards the azo-group, Figure 6. Another factor could be higher steric demand of the C-H fragment, disfavoring attack on the adjacent nitrogen atom of the azo-group.

## 3. Materials and Methods

### 3.1. General Information

All chemicals were of commercial grade and used directly without purification. Melting points were measured on a Stuart SMP20 apparatus (Stuart (Bibby Scientific), Stone, UK). ^1^H and ^13^C NMR spectra were recorded on a Bruker AM-300 (at 300.13 and 75.13 MHz, respectively, Bruker Biospin, Ettlingen, Germany) or Bruker Avance DRX 500 (at 500 and 125 MHz, respectively, Bruker Biospin, Germany) in DMSO-d_6_ or CDCl_3_. *J* values are given in Hz. HRMS spectra were recorded on a Bruker micrOTOF II mass spectrometer using ESI. All reactions were monitored by TLC analysis using ALUGRAM SIL G/UV254 plates, which were visualized with UV light. Compounds **1a**–**c** and **6a–c** were purchased from commercial suppliers. Compound **6d** was synthesized according to the previously described procedure [31].

### 3.2. General Procedure for the Synthesis of Compounds ***2a–c***

To a stirred suspension of NaH (60% in mineral oil, 1.60 g, 40 mmol) in anhydrous THF (50 mL), ethyl acetoacetate (2.55 mL, 20 mmol) was added dropwise. The suspension was stirred for 15 min and the corresponding chloronitroarene (20 mmol) was added in small portions. The reaction mixture was stirred at 40 °C for 2–6 h (monitored by TLC), poured in 250 mL of water and acidified with conc. HCl to pH 3. The red color of the solution disappeared and an oily product started to separate, which was extracted with CHCl_3_. The organic phase was dried over anhydrous Na_2_SO_4_, evaporated and purified via column chromatography (SiO_2_/CHCl_3_). Compounds **2a–c** have a strong tendency to overcool and stay in the form of viscous oil for several weeks before sudden crystallization occurs. Compounds **2a–c** exist as a mixture of ketone and enol tautomers in solutions with the enol form being dominant, thus only NMR data for the enol form were provided below, while compound names correspond to keto tautomers for clarity.

Ethyl 2-(3,5-dinitropyridin-2-yl)-3-oxobutanoate (**2a**), yellow solid; yield 84%; mp 84–85 °C; ^1^H NMR (300 MHz, CDCl_3_): δ 13.57 (s, 1H), 9.59 (d, 1H, J = 1.8 Hz), 9.05 (d, 1H, J = 1.8 Hz), 4.25–4.05 (m, 2H), 2.14 (s, 3H), 1.15 (t, 3H, J = 6.9 Hz). HRMS (ESI, m/z): calcd for C_11_H_11_N_3_O_7_ [M + Na]^+^: 320.0489; found: 320.0480.

Ethyl 2-(3-nitro-5-(trifluoromethyl)pyridin-2-yl)-3-oxobutanoate (**2b**), yellow prisms; yield 77%; mp 59–62 °C; ^1^H NMR (300 MHz, CDCl_3_): δ 13.38 (s, 1H), 9.03 (s, 1H), 8.49 (s, 1H), 4.25–3.95 (m, 2H), 2.01 (s, 3H), 1.10 (t, 3H, J = 6.9 Hz). HRMS (ESI, m/z): calcd for C_12_H_11_F_3_N_2_O_5_ [M + H]^+^: 321.0693; found: 321.0687.

Methyl 6-(1-ethoxy-1,3-dioxobutan-2-yl)-5-nitronicotinate (**2c**), yellow needles; yield 61%; mp 75–78 °C; ^1^H NMR (300 MHz, CDCl_3_): δ 13.39 (s, 1H), 9.37 (d, 1H, J = 1.8 Hz), 8.85 (d, 1H, J = 2.1 Hz), 4.22–4.00 (m, 2H), 4.04 (s, 3H), 2.08 (s, 3H), 1.14 (t, 3H, J = 6.9 Hz). HRMS (ESI, m/z): calcd for C_13_H_14_N_2_O_7_ [M + H]^+^: 311.0874; found: 311.0871.

### 3.3. General Procedure for the Synthesis of Compounds ***7a–d***

The corresponding chloronitroarene (20 mmol) and ethyl acetoacetate (3.19 mL, 25 mmol) were dissolved in 35 mL of DMF then anhydrous K_2_CO_3_ (5.52 g, 40 mmol) was added and the reaction mixture was stirred at 60 °C for 2–6 h (monitored by TLC), poured in 150 mL of water and acidified with conc. HCl to pH 3. The product was extracted with CHCl_3_, the combined organic phase was dried over anhydrous Na_2_SO_4_, evaporated and purified via column chromatography (SiO_2_/CHCl_3_). Compounds **7a–d** have a strong tendency to overcool and stay in the form of viscous oil for several weeks before sudden crystallization occurs. Compounds **7** exist as a mixture of ketone and enol tautomers in solutions with the enol form being dominant, thus only NMR data for the enol form are provided below, while compound names correspond to keto tautomers for clarity.

Ethyl 2-(2,4-dinitrophenyl)-3-oxobutanoate (**7a**), brown solid; yield 66%; mp 106–108 °C; ^1^H NMR (300 MHz, CDCl_3_): δ 13.17 (s, 1H), 8.86 (d, 1H, J = 2.4 Hz), 8.45 (dd, 1H, J = 8.4 Hz, J = 2.1 Hz), 4.30–4.00 (m, 2H), 1.94 (s, 3H), 1.15 (t, 3H, J = 7.2 Hz).

Ethyl 2-(2-nitro-4-(trifluoromethyl)phenyl)-3-oxobutanoate (**7b**), yellow solid; yield 69%; mp 60–62 °C; ^1^H NMR (300 MHz, CDCl_3_): δ 13.11 (s, 1H), 8.27 (d, 1H, J = 1.2 Hz), 7.87 (dd, 1H, J = 7.8 Hz, J = 1.2 Hz), 7.48 (d, 1H, J = 8.1 Hz), 4.30–4.00 (m, 2H), 1.91 (s, 3H), 1.15 (t, 3H, J = 6.9 Hz).

Methyl 4-(1-ethoxy-1,3-dioxobutan-2-yl)-3-nitrobenzoate (**7c**), yellow solid; yield 64%; mp 90–92 °C; ^1^H NMR (300 MHz, CDCl_3_): δ 13.05 (s, 1H), 8.60 (s, 1H), 8.21 (d, 1H, J = 7.8 Hz), 7.38 (d, 1H, J = 7.8 Hz), 4.25–3.90 (m, 2H), 3.97 (s, 3H), 1.87 (s, 3H), 1.09 (t, 3H, J = 6.9 Hz). HRMS (ESI, m/z): calcd for C_14_H_15_NO_7_ [M + H]^+^: 310.0921; found: 310.0926.

Ethyl 2-(5,7-dinitroquinolin-8-yl)-3-oxobutanoate (**7d**), brown solid; yield 62%; mp 92–93 °C; ^1^H NMR (300 MHz, CDCl_3_): δ 13.54 (s, 1H), 9.21 (dd, 1H, J = 4.2 Hz, J = 1.8 Hz), 9.10 (dd, 1H, J = 8.7 Hz, J = 1.8 Hz), 8.88 (s, 1H), 7.82 (dd, 1H, J = 8.7 Hz, J = 4.2 Hz), 4.25–3.95 (m, 2H), 1.70 (s, 3H), 1.05 (t, 3H, J = 7.2 Hz). HRMS (ESI, m/z): calcd for C_15_H_13_N_3_O_7_ [M + H]^+^: 348.0826; found: 348.0833.

### 3.4. General Procedure for the Synthesis of Aryldiazonium Tosylates ***3*** [26]

Although aryldiazonium tosylates are generally considered to be relatively safe compared to other aryldiazonium salts, and no incidents occurred during this work, these compounds should be handled with appropriate precautions.

TsOH*H_2_O (2.28 g, 12 mmol) was dissolved in ethyl acetate (50 mL) and the appropriate aniline (8 mmol) was added. The suspension of anilinium tosylate was stirred for 10 min and iPrONO (2.46 mL, 24 mmol) was added in one portion. The amine salt disappeared and a new precipitate of diazonium salt started to form. The reaction mixture was stirred at room temperature (r.t.) for 2 h and filtered. Diazonium salts were washed with Et_2_O, dried in air and used as is. Yields 67–93%. 2-Methoxyphenyldiazonium tosylate failed to crystallize and was used as oil. These salts can be stored at room temperature for a week with minor discoloration and no visible decomposition was observed after 3 months of storage in a freezer.

### 3.5. Synthesis of the Intermediates ***4a***, ***5a′*** and ***5q*′**

To a solution of compound **2a** (0.32 g, 1 mmol) in MeCN (5 mL) 2-cyanophenyldiazonium tosylate (1.1 mmol) was added followed by pyridine (0.08 mL, 1 mmol). The reaction mixture was stirred at r.t. for 30 min (monitored by TLC) then poured in 50 mL of water, acidified to pH 3 with conc. hydrochloric acid and extracted with CHCl_3_. Combined organic phase was dried over anhydrous Na_2_SO_4_, evaporated and the residue was purified by column chromatography (SiO_2_/CHCl_3_). 

Ethyl 2-((2-cyanophenyl)diazenyl)-2-(3,5-dinitropyridin-2-yl)-3-oxobutanoate (**4a**), orange oil, yield 98%; ^1^H NMR (300 MHz, CDCl_3_): δ 9.56 (d, 1H, J = 1.8 Hz), 9.22 (d, 1H, J = 2.1 Hz), 7.55–7.74 (m, 4H), 4.40 (q, 2H, J = 7.2 Hz), 2.61 (s, 3H), 1.31 (t, 3H, J = 7.2 Hz). HRMS (ESI, m/z): calcd for C_18_H_14_N_6_O_7_ [M + Na]^+^: 449.0816; found: 449.0812.

To a solution of the appropriate azo-compound **4** (obtained from 1 mmol of **2**) in MeCN (10 mL), pyrrolidine was added (0.5 mL). The dark-red solution was stirred at r.t. for 3 min, then poured in 50 mL of 1N hydrochloric acid and extracted with CHCl_3_. Organic phase was dried over anhydrous Na_2_SO_4_, evaporated and the residue was purified by column chromatography (SiO_2_/10% EtOAc in CHCl_3_).

Ethyl 2-(2-acetyl-2-(2-cyanophenyl)hydrazineylidene)-2-(3,5-dinitropyridin-2-yl)acetate (**5a′**), pale yellow solid; yield 89%; mp 151–152 °C; ^1^H NMR (300 MHz, CDCl_3_): δ 9.46 (s, 1H), 8.86 (s, 1H), 7.43–7.07 (m, 4H), 4.34 (q, 2H), J = 6.9 Hz), 2.76 (s, 3H), 1.35 (t, 3H, J = 6.9 Hz). HRMS (ESI, m/z): calcd for C_18_H_14_N_6_O_7_ [M + Na]^+^: 449.0816; found: 449.0811.

Methyl 6-(1-(2-acetyl-2-(4-fluorophenyl)hydrazineylidene)-2-ethoxy-2-oxoethyl)-5-nitronicotinate (**5q′**), pale yellow solid; yield 85%; mp 120–121 °C; ^1^H NMR (300 MHz, CDCl_3_): δ 9.21 (s, 1H), 8.69 (s, 1H), 6.86–6.81 (m, 2H), 6.70–6.64 (m, 2H), 4.35–4.25 (m, 2H), 4.04 (s, 1H), 2.70 (s, 3H), 1.30 (t, 3H, J = 6.9 Hz). HRMS (ESI, m/z): calcd for C_19_H_17_FN_4_O_7_ [M + Na]^+^: 455.0973; found: 455.0971.

### 3.6. General Procedure for the Synthesis of Pyrazolo [4,3-b]pyridines 5 and Indazoles 8

To a solution of nitroaryl-substituted acetoacetic ester **2** or **7** (1 mmol) in MeCN (5 mL), appropriate aryldiazonium tosylate (1.1 mmol) was added followed by 0.08 mL (1 mmol) of pyridine. The reaction mixture was stirred at r.t. for 5–60 min (monitored by TLC) then pyrrolidine (0.33 mL, 4 mmol) was added and the reaction mixture was stirred at 40 °C for another 15–90 min (monitored by TLC). The reaction mixture was cooled to r.t., poured in 50 mL of 1N hydrochloric acid and extracted with CHCl_3_. In some cases, product can be directly filtered from the cooled reaction mixture. Isolated products were purified by flash chromatography (SiO_2_/10% EtOAc in CHCl_3_) and/or recrystallization.

In the case of compounds **7b** and **7c**, pyridine was substituted for 0.45 mL (3 mmol) of DBU and the amount of pyrrolidine was reduced to 1 mmol.

Ethyl 1-(2-cyanophenyl)-6-nitro-1*H*-pyrazolo [4,3-*b*]pyridine-3-carboxylate (**5a**), white solid; yield 85%, mp 222–224 °C; ^1^H NMR (300 MHz, CDCl_3_): δ 9.70 (d, 1H, J = 2.1 Hz), 8.68 (d, 1H, J = 2.1 Hz), 8.05–7.90 (m, 2H), 7.85–7.75 (m, 2H), 4.65 (q, 2H, J = 7.2 Hz), 1.53 (t, 3H, J = 7.2 Hz). ^13^C NMR (75 MHz, CDCl_3_): δ 160.1, 143.7, 143.2, 142.3, 139.2, 139.1, 134.7, 134.6, 132.6, 130.7, 127.8, 115.2, 115.0, 110.3, 62.4, 14.4. HRMS (ESI, m/z): calcd for C_16_H_11_N_5_O_4_ [M + H]^+^: 338.0884; found: 338.0881.

Ethyl 1-(4-methyl-2-nitrophenyl)-6-nitro-1*H*-pyrazolo [4,3-*b*]pyridine-3-carboxylate (**5b**), white solid; yield 88%; mp 252–253 °C; ^1^H NMR (300 MHz, CDCl_3_): δ 9.63 (d, 1H, J = 1.8 Hz), 8.46 (d, 1H, J = 1.8 Hz), 8.06 (s, 1H), 7.67 (d, 1H, J = 8.1 Hz), 7.55 (d, 1H, J = 8.1 Hz), 4.57 (q, 2H, J = 7.2 Hz), 2.60 (s, 3H), 1.46 (t, 3H, J = 6.9 Hz). ^13^C NMR (75 MHz, CDCl_3_): δ 145.2, 143.5, 143.3, 142.9, 142.4, 135.1, 133.7, 129.5, 128.1, 126.8, 114.7, 62.2, 21.4, 14.4. HRMS (ESI, m/z): calcd for C_16_H_13_N_5_O_6_ [M + H]^+^: 372.0939; found: 372.0943.

Ethyl 1-(2-(methoxycarbonyl)phenyl)-6-nitro-1*H*-pyrazolo [4,3-*b*]pyridine-3-carboxylate (**5c**), colorless crystals; yield 65%; mp 136–137 °C; ^1^H NMR (300 MHz, CDCl_3_): δ 9.60 (s, 1H), 8.42 (s, 1H), 8.16 (d, 1H, J = 7.5 Hz), 7.80–7.68 (m, 2H), 7.57 (d, 1H, J = 7.5 Hz), 4.57 (q, 2H, 7.2 Hz), 3.58 (s, 3H), 1.46 (t, 3H, J = 6.9 Hz). ^13^C NMR (75 MHz, CDCl_3_): δ 164.5, 160.6, 143.0, 142.7, 142.1, 137.4, 136.5, 133.7, 133.6, 132.1, 130.9, 128.7, 128.4, 115.1, 62.0, 52.7, 14.4. HRMS (ESI, m/z): calcd for C_17_H_14_N_4_O_6_ [M + H]^+^: 371.0986; found: 371.0984.

Ethyl 1-(4-fluorophenyl)-6-nitro-1*H*-pyrazolo [4,3-*b*]pyridine-3-carboxylate (**5d**), white crystals; yield 76%; mp 214–215 °C; ^1^H NMR (300 MHz, CDCl_3_): δ 9.60 (d, 1H, J = 1.8 Hz), 8.78 (d, 1H, J = 2.1 Hz), 7.72–7.67 (m, 2H), 7.32 (t, 2H, J = 8.1 Hz), 4.58 (q, 2H, J = 7.2 Hz), 1.47 (t, 3H, J = 7.2 Hz). ^13^C NMR (75 MHz, CDCl_3_): δ 162.8 (d, ^1^J_CF_ = 249 Hz), 160.4, 143.3, 143.2, 142.1, 137.6, 133.9, 131.7, 125.7 (d, ^3^J_CF_ = 8.8 Hz), 117.3 (d, ^2^J_CF_ = 23.2 Hz), 115.3, 62.1, 14.4. HRMS (ESI, m/z): calcd for C_15_H_11_FN_4_O_4_ [M + Na]^+^: 353.0657; found: 353.0667.

Ethyl 1-(3-chloro-4-methylphenyl)-6-nitro-1*H*-pyrazolo [4,3-*b*]pyridine-3-carboxylate (**5e**), beige crystals; yield 69%; mp 203–205 °C; ^1^H NMR (300 MHz, CDCl_3_): δ 9.61 (d, 1H, J = 1.8 Hz), 8.81 (d, 1H, J = 1.8 Hz), 7.73 (s, 1H), 7.52–7.45 (m, 2H), 4.58 (q, 2H, J = 7.2 Hz), 2.47 (s, 3H), 1.48 (t, 3H, J = 7.2 Hz). ^13^C NMR (75 MHz, CDCl_3_): δ 160.4, 143.3, 143.2, 142.2, 137.9, 137.7, 136.4, 135.9, 132.1, 131.5, 124.3, 121.4, 115.4, 62.1, 19.4, 14.4. HRMS (ESI, m/z): calcd for C_16_H_13_ClN_4_O_4_ [M + H]^+^: 361.0698; found: 361.0695.

Ethyl 1-(2-chlorophenyl)-6-nitro-1*H*-pyrazolo [4,3-*b*]pyridine-3-carboxylate (**5f**), white solid; yield 72%; mp 234–235 °C; ^1^H NMR (300 MHz, CDCl_3_): δ 9.63 (s, 1H), 8.46 (s, 1H), 7.67–7.49 (m, 4H), 4.59 (q, 2H, J = 6.9 Hz), 1.48 (t, 3H, J = 7.2 Hz). ^13^C NMR (75 MHz, CDCl_3_): δ 160.4, 143.2, 142.7, 142.1, 138.1, 134.8, 133.1, 132.0, 131.2, 131.0, 129.7, 128.4, 115.7, 62.2, 14.4. HRMS (ESI, m/z): calcd for C_15_H_11_ClN_4_O_4_ [M + H]^+^: 347.0542; found: 347.0548.

Ethyl 1-(2-methyl-4-nitrophenyl)-6-nitro-1*H*-pyrazolo [4,3-*b*]pyridine-3-carboxylate (**5g**), white crystals; yield 73%; mp 191–193 °C; ^1^H NMR (300 MHz, CDCl_3_): δ 9.67 (d, 1H, J = 2.1 Hz), 8.51 (d, 1H, J = 1.8 Hz), 8.38 (s, 1H), 8.32 (dd, 1H, J = 8.4 Hz, J = 1.8 Hz), 7.66 (d, 1H, J = 8.7 Hz), 4.63 (q, 2H, J = 6.9 Hz), 2.34 (s, 3H), 1.52 (t, 3H, J = 6.9 Hz). ^13^C NMR (75 MHz, CDCl_3_): δ 160.2, 148.6, 143.6, 142.8, 142.4, 140.8, 138.5, 137.7, 132.9, 128.2, 127.2, 122.6, 114.8, 62.3, 18.3, 14.4. HRMS (ESI, m/z): calcd for C_16_H_13_N_5_O_6_ [M + H]^+^: 372.0939; found: 372.0931.

Ethyl 1-(2-methoxyphenyl)-6-nitro-1*H*-pyrazolo [4,3-*b*]pyridine-3-carboxylate (**5h**), white needles; yield 78%; mp 203–205 °C; ^1^H NMR (300 MHz, CDCl_3_): δ 9.60 (d, 1H, J = 2.1 Hz), 8.48 (d, 1H, J = 2.1 Hz), 7.60–7.50 (m, 2H), 7.20–7.15 (m, 2H), 4.60 (q, 2H, J = 7.2 Hz), 3.81 (s, 3H), 1.49 (t, 3H, J = 6.9 Hz). ^13^C NMR (75 MHz, CDCl_3_): δ 160.7, 153.5, 142.8, 141.9, 137.5, 133.2, 131.6, 128.5, 126.2, 121.5, 116.5, 112.5, 61.9, 56.0, 14.4. HRMS (ESI, m/z): calcd for C_16_H_14_N_4_O_5_ [M + H]^+^: 343.1037; found: 343.1031.

Ethyl 6-nitro-1-(4-(trifluoromethyl)phenyl)-1*H*-pyrazolo [4,3-*b*]pyridine-3-carboxylate (**5i**), beige needles; yield 82%; mp 193–194 °C; ^1^H NMR (300 MHz, CDCl_3_): δ 9.65 (d, 1H, J = 2.4 Hz), 8.93 (d, 1H, J = 2.4 Hz), 7.99–7.92 (m, 4H), 4.63 (q, 2H, J = 7.2 Hz), 1.52 (t, 3H, J = 7.2 Hz). ^13^C NMR (75 MHz, CDCl_3_): δ 160.3, 143.6, 143.5, 142.3, 140.6, 138.5, 131.5, 131.2 (q, ^2^J_CF_ = 30 Hz), 127.5 (q, ^3^J_CF_ = 3.6 Hz), 123.5, 123.4 (q, ^1^J_CF_ = 271 Hz), 115.3, 62.3, 14.4. HRMS (ESI, m/z): calcd for C_16_H_11_F_3_N_4_O_4_ [M + H]^+^: 381.0805; found: 381.0798.

Ethyl 1-(4-bromophenyl)-6-nitro-1*H*-pyrazolo [4,3-*b*]pyridine-3-carboxylate (**5j**), white crystals; yield 83%; mp 208–209 °C; ^1^H NMR (300 MHz, CDCl_3_): δ 9.64 (s, 1H), 8.64 (s, 1H), 7.79 (d, 2H, J = 8.4 Hz), 7.66 (d, 2H, J = 8.4 Hz), 4.62 (q, 2H, J = 6.9 Hz), 1.51 (t, 3H, J = 7.2 Hz). ^13^C NMR (75 MHz, CDCl_3_): δ 160.4, 143.4, 142.2, 138.0, 136.8, 133.4, 131.4, 124.9, 123.3, 115.3, 62.2, 14.4. HRMS (ESI, m/z): calcd for C_15_H_11_BrN_4_O_4_ [M + Na]^+^: 412.9856; found: 412.9843.

Ethyl 1-(3-chloro-4-methylphenyl)-6-(trifluoromethyl)-1*H*-pyrazolo [4,3-*b*]pyridine-3-carboxylate (**5k**), beige needles; yield 78%; mp 153–154 °C; ^1^H NMR (300 MHz, CDCl_3_): δ 9.02 (s, 1H), 8.23 (s, 1H), 7.68 (s, 1H), 7.48–7.40 (m, 4H), 4.56 (q, 2H, J = 7.2 Hz), 2.43 (s, 3H), 1.46 (t, 3H, J = 6.9 Hz). ^13^C NMR (75 MHz, CDCl_3_): δ 160.8, 145.0 (q, ^3^J_CF_ = 3.2 Hz), 142.3, 137.6, 137.5, 136.7, 135.8, 132.0, 131.7, 124.7 (q, ^2^J_CF_ = 33 Hz), 124.3, 123.4 (q, ^1^J_CF_ = 272 Hz), 121.5, 116.9 (q, ^3^J_CF_ = 4.1 Hz), 62.0, 19.9, 14.4. HRMS (ESI, m/z): calcd for C_17_H_13_ClF_3_N_3_O_2_ [M + H]^+^: 384.0721; found: 384.0721.

Ethyl 1-(4-methyl-2-nitrophenyl)-6-(trifluoromethyl)-1*H*-pyrazolo [4,3-*b*]pyridine-3-carboxylate (**5l**), white needles; yield 84%; mp 220–222 °C; ^1^H NMR (300 MHz, CDCl_3_): δ 9.10 (s, 1H), 8.05 (s, 1H), 7.94 (s, 1H), 7.69 (d, 1H, J = 7.8 Hz), 7.58 (d, 1H, J = 8.1 Hz), 4.59 (q, 2H, J = 7.2 Hz), 2.61 (s, 3H), 1.49 (t, 3H, J = 7.2 Hz). ^13^C NMR (75 MHz, CDCl_3_): δ 160.5, 145.3, 145.1 (q, ^3^J_CF_ = 3.3 Hz), 142.8, 142.1, 138.4, 134.9, 133.6, 129.3, 128.3, 126.6, 124.9 (q, ^2^J_CF_ = 33.4 Hz), 123.3 (q, ^1^J_CF_ = 270 Hz), 116.1 (q, ^3^J_CF_ = 4.1 Hz), 62.0, 21.3, 14.4. HRMS (ESI, m/z): calcd for C_17_H_13_F_3_N_4_O_4_ [M + H]^+^: 395.0962; found: 395.0959.

Ethyl 1-(2-cyanophenyl)-6-(trifluoromethyl)-1*H*-pyrazolo [4,3-*b*]pyridine-3-carboxylate (**5m**), beige needles; yield 75%; mp 199–200 °C; ^1^H NMR (300 MHz, CDCl_3_): δ 9.10 (d, 1H, J = 1.5 Hz), 8.11 (s, 1H), 7.96–7.85 (m, 2H), 7.76–7.69 (m, 2H), 4.61 (q, 2H, J = 7.2 Hz), 1.50 (t, 3H, J = 7.2 Hz). ^13^C NMR (75 MHz, CDCl_3_): δ 160.4, 145.3 (q, ^3^J_CF_ = 3.2 Hz), 142.4, 139.5, 138.9, 134.6, 134.5, 132.7, 130.3, 127.7, 125.0 (q, ^2^J_CF_ = 33 Hz), 123.3 (q, ^1^J_CF_ = 272 Hz), 116.8 (q, ^3^J_CF_ = 4.2 Hz), 115.1, 110.2, 62.1, 14.4. HRMS (ESI, m/z): calcd for C_17_H_11_F_3_N_4_O_2_ [M + H]^+^: 361.0907; found: 361.0896.

Ethyl 1-(2-(methoxycarbonyl)phenyl)-6-(trifluoromethyl)-1*H*-pyrazolo [4,3-*b*]pyridine-3-carboxylate (**5n**), white crystals; yield 65%; mp 157–158 °C; ^1^H NMR (300 MHz, CDCl_3_): δ 9.07 (d, 1H, J = 1.5 Hz), 8.15 (dd, 1H, J_1_ = 7.8 Hz, J_2_ = 1.5 Hz), 7.91 (s, 1H), 7.81–7.67 (m, 2H), 7.61 (d, 1H, J = 7.5 Hz), 4.60 (q, 2H, J = 7.2 Hz), 3.58 (s, 3H), 1.49 (t, 3H, J = 6.9 Hz). ^13^C NMR (75 MHz, CDCl_3_): δ 164.9, 160.9, 144.7 (q, ^3^J_CF_ = 3.3 Hz), 142.0, 137.2, 136.7, 133.6, 133.4, 132.0, 130.4, 128.4, 124.5 (q, ^2^J_CF_ = 33 Hz), 123.5 (q, ^1^J_CF_ = 271 Hz), 116.5 (q, ^3^J_CF_ = 4.2 Hz), 61.8, 52.6, 14.4. HRMS (ESI, m/z): calcd for C_18_H_14_F_3_N_3_O_4_ [M + H]^+^: 394.1009; found: 394.1006.

Ethyl 1-(2-methoxyphenyl)-6-(trifluoromethyl)-1*H*-pyrazolo [4,3-*b*]pyridine-3-carboxylate (**5o**), off-white solid; yield 63%; mp 142–144 °C; ^1^H NMR (300 MHz, CDCl_3_): δ 9.05 (d, 1H, J = 1.5 Hz), 7.90 (s, 1H), 7.58–7.51 (m, 2H), 7.19–7.14 (m, 2H), 4.60 (q, 2H, J = 7.2 Hz), 3.79 (s, 3H), 1.49 (t, 3H, J = 7.2 Hz). ^13^C NMR (125 MHz, CDCl_3_): δ 161.0, 153.6, 144.4, 141.9, 137.2, 133.2, 131.3, 128.5, 126.4, 124.0 (q, ^2^J_CF_ = 32.8 Hz), 123.6 (q, ^1^J_CF_ = 271 Hz), 121.4, 117.9 (q, ^3^J_CF_ = 4.1 Hz), 112.3, 61.7, 55.8, 14.4. HRMS (ESI, m/z): calcd for C_17_H_14_F_3_N_3_O_3_ [M + H]^+^: 366.1060; found: 366.1069.

Ethyl 1-(4-fluorophenyl)-6-(trifluoromethyl)-1*H*-pyrazolo [4,3-*b*]pyridine-3-carboxylate (**5p**), beige solid; yield 71%; mp 138–140 °C; ^1^H NMR (300 MHz, CDCl_3_): δ 9.11 (d, 1H, J = 1.8 Hz), 8.25 (s, 1H), 7.75–7.69 (m, 2H), 7.39–7.30 (m, 2H), 4.64 (q, 2H, J = 7.2 Hz), 1.53 (t, 3H, J = 6.9 Hz). ^13^C NMR (125 MHz, CDCl_3_): δ 162.6 (d, ^1^J_CF_ = 249 Hz), 160.8, 145.0, 142.4, 137.5, 134.1, 134.0, 131.8, 125.7 (d, ^3^J_CF_ = 8.8 Hz), 124.7 (q, ^2^J_CF_ = 33 Hz), 123.5 (q, ^1^J_CF_ = 272 Hz), 117.1 (d, ^2^J_CF_ = 23.3 Hz), 116.6 (q, ^3^J_CF_ = 4.1 Hz), 61.9, 14.4. HRMS (ESI, m/z): calcd for C_16_H_11_F_4_N_3_O_2_ [M + H]^+^: 354.0860; found: 354.0861.

3-Ethyl 6-methyl 1-(4-fluorophenyl)-1*H*-pyrazolo [4,3-*b*]pyridine-3,6-dicarboxylate (**5q**), beige needles; yield 77%; mp 218–220 °C; ^1^H NMR (300 MHz, CDCl_3_): δ 9.42 (d, 1H, J = 1.2 Hz), 8.62 (d, 1H, J = 1.2 Hz), 7.75–7.70 (m, 2H), 7.32 (t, 2H, J = 8.4 Hz), 4.62 (q, 2H, J = 6.9 Hz), 4.02 (s, 3H), 1.50 (t, 3H, J = 7.2 Hz). ^13^C NMR (75 MHz, CDCl_3_): δ 165.3, 162.5 (d, ^1^J_CF_ = 248 Hz), 161.0, 149.1, 142.9, 137.3, 134.3, 132.6, 125.7 (d, ^3^J_CF_ = 8.7 Hz), 124.0, 120.8, 117.0 (d, ^2^J_CF_ = 23.1 Hz), 61.8, 52.9, 14.5. HRMS (ESI, m/z): calcd for C_17_H_14_FN_3_O_4_ [M + H]^+^: 344.1041; found: 344.1048.

3-Ethyl 6-methyl 1-(2-(methoxycarbonyl)phenyl)-1*H*-pyrazolo [4,3-*b*]pyridine-3,6-dicarboxylate (**5r**), white solid; yield 73%; mp 174–176 °C; ^1^H NMR (300 MHz, DMSO-d_6_): δ 9.28 (s, 1H), 8.34 (s, 1H), 8.09 (d, 1H, J = 7.4 Hz), 7.92 (s, 2H), 7.83 (m, 1H), 4.46 (q, 2H, J = 6.9 Hz), 3.93 (s, 3H), 3.51 (s, 3H), 1.38 (t, 3H, J = 7.0 Hz). ^13^C NMR (75 MHz, DMSO-d_6_): δ 165.6, 165.3, 160.8, 148.4, 141.9, 136.6, 134.1, 131.7, 130.9, 128.6, 128.5, 124.0, 120.9, 61.4, 53.2, 52.9, 14.7. HRMS (ESI, m/z): calcd for C_19_H_17_N_3_O_6_ [M + H]^+^: 384.1190; found: 384.1207.

3-Ethyl 6-methyl 1-(4-bromophenyl)-1*H*-pyrazolo [4,3-*b*]pyridine-3,6-dicarboxylate (**5s**), white crystals; yield 84%; mp 195–196 °C; ^1^H NMR (300 MHz, CDCl_3_): δ 9.39 9d, 1H, J = 1.5 Hz), 8.64 (d, 1H, J = 1.2 Hz), 7.73 (d, 2H, J = 8.7 Hz), 7.64 (d, 2H, J = 9 Hz), 4.60 (q, 2H, J = 6.9 Hz), 4.00 (s, 3H), 1.49 (t, 3H, J = 6.9 Hz). ^13^C NMR (75 MHz, CDCl_3_): δ 165.2, 160.9, 149.2, 143.0, 137.6, 137.3, 133.1, 132.3, 124.9, 124.1, 122.6, 120.8, 61.9, 52.9, 14.5. HRMS (ESI, m/z): calcd for C_17_H_14_BrN_3_O_4_ [M + H]^+^: 404.0240; found: 404.0242.

Ethyl 1-(4-fluorophenyl)-6-nitro-1*H*-indazole-3-carboxylate, off-white needles (**8a**); yield 69%; mp 200–201 °C; ^1^H NMR (300 MHz, CDCl_3_): δ 8.51 (s, 1H), 8.42 (d, 1H, J = 9 Hz), 8.20 (d, 1H, J = 9 Hz), 7.71–7.67 (m, 2H), 7.28 (t, 2H, J = 8.4 Hz), 4.55 (q, 2H, J = 6.9 Hz), 1.49 (t, 3H, J = 6.9 Hz). ^13^C NMR (75 MHz, CDCl_3_): δ 162.5 (d, ^1^J_CF_ = 249 Hz), 161.7, 147.5, 139.3, 137.5, 134.3, 127.3, 126.1 (d, ^3^J_CF_ = 8.8 Hz), 123.7, 118.4, 117.0 (d, ^2^J_CF_ = 23.1 Hz), 107.4, 61.8, 14.4. HRMS (ESI, m/z): calcd for C_16_H_12_FN_3_O_4_ [M + Na]^+^: 352.0704; found: 352.0689.

Diethyl 1,1′-(thiobis(4,1-phenylene))bis(6-nitro-1*H*-indazole-3-carboxylate) (**8b**), yellow solid; yield 61%; mp 222–224 °C; ^1^H NMR (300 MHz, CDCl_3_): δ 8.60 (s, 2H), 8.43 (d, 2H, J = 8.7 Hz), 8.21 (d, 2H, J = 8.7 Hz), 7.72 (d, 4H, J = 8.4 Hz), 7.62 (d, 4H, J = 8.4 Hz), 4.54 (q, 4H, J = 7.2 Hz), 1.48 (t, 6H, J = 7.2 Hz). ^13^C NMR (75 MHz, CDCl_3_): δ 161.6, 147.5, 139.1, 137.8, 137.5, 136.5, 132.6, 127.5, 124.8, 123.8, 118.5, 107.5, 61.8, 14.4. HRMS (ESI, m/z): calcd for C_32_H_24_N_6_O_8_S [M + Na]^+^: 675.1250; found: 675.1269.

Ethyl 1-(2-cyanophenyl)-6-nitro-1*H*-indazole-3-carboxylate (**8c**), white solid; yield 83%; mp 228–230 °C; ^1^H NMR (300 MHz, CDCl_3_): δ 8.52 (d, 1H, J = 9 Hz), 8.37 (s, 1H), 8.30 (d, 1H, J = 9 Hz), 8.00–7.85 (m, 2H), 7.80–7.70 (m, 2H), 4.60 (q, 2H, J = 6.9 Hz), 1.53 (t, 3H, J = 6.9 Hz). ^13^C NMR (75 MHz, CDCl_3_): δ 161.4, 147.7, 140.1, 139.7, 134.7, 134.3, 130.3, 127.7, 124.1, 118.8, 115.2, 110.8, 107.1, 62.0, 14.4. HRMS (ESI, m/z): calcd for C_17_H_12_N_4_O_4_ [M + H]^+^: 337.0931; found: 337.0937.

Ethyl 1-(4-bromophenyl)-6-nitro-1*H*-indazole-3-carboxylate (**8d**), beige solid; yield 75%; mp 158–159 °C; ^1^H NMR (300 MHz, DMSO-d_6_): δ 8.61 (d, 1H, J = 1.5 Hz), 8.40 (d, 1H, J = 8.9 Hz)), 8.27 (dd, 1H, J = 8.9 Hz, J = 1.5 Hz), 7.88 (m, 4H), 4.49 (q, 2H, J = 7.1 Hz)), 1.43 (t, 3H, J = 7.0 Hz). HRMS (ESI, m/z): calcd for C_16_H_12_BrN_3_O_4_ [M + Na]^+^: 411.9903; found: 411.9912.

Ethyl 2-(2-(2-chlorophenyl)hydrazinylidene)-2-(2-nitro-4-(trifluoromethyl)phenyl)acetate (**10e**), yellow solid; yield 75%; mp 144–145 °C; ^1^H NMR (300 MHz, CDCl_3_): δ 12.93 (s, 1H), 8.34 (s, 1H), 7.95 (d, 1H, J = 8.1 Hz), 8.40 (d, 1H, J = 8.1 Hz), 7.66 (d, 1H, J = 8.1 Hz), 7.39 (d, 1H, J = 7.8 Hz), 7.28 (t, 1H, J = 7.5 Hz), 7.00 (t, 1H, J = 7.2 Hz), 4.28 (q, 2H, J = 7.2 Hz), 1.26 (t, 3H, J = 7.2 Hz). ^13^C NMR (75 MHz, CDCl_3_): δ 161.7, 148.6, 139.1, 135.0, 133.3, 131.3 (q, ^2^J_CF_ = 34.2 Hz), 129.7 (q, ^3^J_CF_ = 3.3 Hz), 129.6, 127.9, 126.8, 123.5, 122.9 (q, ^1^J_CF_ = 271 Hz), 121.7 (q, ^3^J_CF_ = 3.8 Hz), 119.9, 114.9, 61.9, 13.7. HRMS (ESI, m/z): calcd for C_17_H_13_ClF_3_N_3_O_4_ [M + Na]^+^: 438.0439; found: 438.0447.

Ethyl 1-(2-chlorophenyl)-6-(trifluoromethyl)-1*H*-indazole-3-carboxylate (**8e**), white crystals; yield 62%; mp 116–117 °C; ^1^H NMR (300 MHz, CDCl_3_): δ 8.46 (d, 1H, J = 8.4 Hz), 7.67–7.48 (m, 6H), 4.59 (q, 2H, J = 7.2 Hz), 1.52 (t, 3H, J = 6.9 Hz). ^13^C NMR (125 MHz, CDCl_3_): δ 162.0, 140.7, 137.6, 135.6, 131.8, 131.3, 130.8, 129.8 (q, ^2^J_CF_ = 32.4 Hz), 129.8, 128.0, 125.2, 124.0 (q, ^1^J_CF_ = 271 Hz), 123.5, 120.0 (q, ^3^J_CF_ = 3 Hz), 108.7 (q, ^3^J_CF_ = 4.5 Hz), 61.5, 14.4. HRMS (ESI, m/z): calcd for C_17_H_12_ClF_3_N_2_O_2_ [M + H]^+^: 369.0612; found: 369.0607.

Ethyl 1-(3-chloro-4-methylphenyl)-6-(trifluoromethyl)-1*H*-indazole-3-carboxylate (**8f**), off-white solid; yield 74%; mp 127–128 °C; ^1^H NMR (300 MHz, CDCl_3_): δ 8.43 (d, 1H, J = 8.4 Hz), 7.96 (s, 1H), 7.75 (d, 1H, J = 1.5 Hz), 7.61 (d, 1H, J = 8.4 Hz), 7.53 (dd, 1H, J = 8.1 Hz, J = 1.5 Hz), 7.45 (d, 1H, J = 8.1 Hz), 4.58 (q, 2H, J = 6.9 Hz), 2.49 (s, 3H), 1.53 (t, 3H, J = 7.2 Hz). ^13^C NMR (125 MHz, CDCl_3_): δ 161.9, 139.2, 137.3, 137.2, 136.8, 135.4, 131.7, 130.0 (q, ^2^J_CF_ = 32.3 Hz), 126.0, 124.7, 124.0 (q, ^1^J_CF_ = 271 Hz), 123.7, 121.8, 120.2 (q, ^3^J_CF_ = 2.8 Hz), 108.4 (q, ^3^J_CF_ = 4.4 Hz), 61.5, 19.8, 14.4. HRMS (ESI, m/z): calcd for C_18_H_14_ClF_3_N_2_O_2_ [M + H]^+^: 383.0769; found: 383.0767.

Ethyl 1-(4-bromophenyl)-6-(trifluoromethyl)-1*H*-indazole-3-carboxylate (**8g**), white needles; yield 79%, mp 129–130 °C; ^1^H NMR (300 MHz, CDCl_3_): δ 8.46 (d, 1H, J = 8.7 Hz), 7.96 (s, 1H), 7.77–7.74 (m, 2H), 7.67–7.62 (m, 3H), 4.59 (q, 2H, 7.2 Hz), 1.53 (t, 3H, J = 7.2 Hz). ^13^C NMR (75 MHz, CDCl_3_): δ 161.9, 139.2, 137.6, 133.0, 130.1 (q, ^2^J_CF_ = 32.4 Hz), 126.2, 125.4, 124.0 (q, ^1^J_CF_ = 271 Hz), 123.8, 122.4, 120.3 (q, ^3^J_CF_ = 2.9 Hz), 108.4 (q, ^3^J_CF_ = 4.5 Hz), 61.6, 14.4. HRMS (ESI, m/z): calcd for C_17_H_12_BrF_3_N_2_O_2_ [M + H]^+^: 413.0107; found: 413.0103.

3-Ethyl 6-methyl 1-(2-chlorophenyl)-1*H*-indazole-3,6-dicarboxylate (**8h**), white solid; yield 77%; mp 196–197 °C; ^1^H NMR (300 MHz, CDCl_3_): δ 8.36 (d, 1H, J = 8.4 Hz), 8.03 (d, 1H, J = 8.7 Hz), 7.97 (s, 1H), 7.65–7.46 (m, 4H), 4.57 (q, 2H, J = 7.2 Hz), 3.94 (s, 3H), 1.50 (t, 3H, J = 7.2 Hz). ^13^C NMR (75 MHz, CDCl_3_): δ 166.7, 162.1, 141.4, 137.6, 135.9, 132.0, 131.2, 130.8, 129.9, 129.5, 127.9, 126.0, 124.0, 122.4, 113.2, 61.5, 52.5, 14.5. HRMS (ESI, m/z): calcd for C_18_H_15_ClN_2_O_4_ [M + H]^+^: 359.0793; found: 359.0801.

3-Ethyl 6-methyl 1-(4-bromophenyl)-1*H*-indazole-3,6-dicarboxylate (**8i**), gray crystals; yield 81%; mp 158–159 °C; ^1^H NMR (300 MHz, CDCl_3_): δ 8.38 (s, 1H), 8.35 (d, 1H, J = 8.4 Hz), 8.03 (d, 1H, J = 8.1 Hz), 7.73 (d, 2H, J = 7.8 Hz), 7.66 (d, 2H, J = 7.8 Hz), 4.57 (q, 2H, J = 6.9 Hz), 3.98 (s, 3H), 1.52 (t, 3H, J = 6.9 Hz). ^13^C NMR (75 MHz, CDCl_3_): δ 166.6, 162.1, 139.7, 137.8, 137.5, 132.9, 129.7, 127.0, 125.4, 124.3, 122.6, 122.1, 112.9, 61.5, 52.6, 14.5. HRMS (ESI, m/z): calcd for C_18_H_15_BrN_2_O_4_ [M + H]^+^: 403.0288; found: 403.0286.

3-Ethyl 6-methyl 1-(4-methyl-2-nitrophenyl)-1*H*-indazole-3,6-dicarboxylate (**8j**), off-white solid; yield 60%; mp 163–165 °C; ^1^H NMR (300 MHz, CDCl_3_): δ 8.38 (d, 1H, J = 8.7 Hz), 8.07–8.02 (m, 3H), 7.67–7.59 (m, 2H), 4.56 (q, 2H, J = 7.2 Hz), 3.95 (s, 3H), 2.60 (s, 3H), 1.50 (t, 3H, J = 7.2 Hz). ^13^C NMR (75 MHz, CDCl_3_): δ 166.6, 161.9, 145.4, 141.8, 141.5, 138.2, 134.6, 129.9, 129.4, 129.2, 126.4, 124.3, 122.7, 112.1, 61.6, 52.5, 21.3, 14.4. HRMS (ESI, m/z): calcd for C_19_H_17_N_3_O_6_ [M + H]^+^: 384.1190; found: 384.1185.

Ethyl 7-(4-bromophenyl)-5-nitro-7*H*-pyrazolo [3,4-*h*]quinoline-9-carboxylate (**8k**), beige crystals; yield 64%; mp 206–208 °C; ^1^H NMR (300 MHz, CDCl_3_): δ 9.15 (dd, 1H, J = 4.2 Hz, J = 1.5 Hz), 8.92 (dd, 1H, J = 7.2 Hz, J = 1.5 Hz), 8.54 (s, 1H), 7.80 (d, 2H, J = 8.7 Hz), 7.70–7.65 (m, 3H), 4.67 (q, 2H, J = 6.9 Hz), 1.55 (t, 3H, J = 7.2 Hz). ^13^C NMR (75 MHz, CDCl_3_): δ 162.8, 151.9, 146.5, 142.9, 141.1, 137.0, 136.5, 133.2, 132.5, 125.7, 123.1, 122.5, 121.7, 117.6, 110.4, 62.3, 14.3. HRMS (ESI, m/z): calcd for C_19_H_13_BrN_4_O_4_ [M + H]^+^: 441.0193; found: 441.0189.

### 3.7. X-ray Crystallographic Data and Refinement Details

X-ray diffraction data for **2a, 5a′, 5q′** and **5c** were collected at 100K on a Rigaku Synergy S diffractometer (Wroclaw, Poland) equipped with a HyPix6000HE area-detector (kappa geometry, shutterless ω-scan technique), using monochromatized Cu K_α_-radiation. The intensity data were integrated and corrected for absorption and decay by the CrysAlisPro program [32]. X-ray diffraction data for **2b** were collected at 100K on a Bruker Quest D8 diffractometer (Karlsruhe, Germany) equipped with a Photon-III area-detector (shutterless φ- and ω-scan technique), using graphite-monochromatized Mo K_α_-radiation. The intensity data were integrated by the SAINT program [33] and were corrected for absorption and decay using SADABS [34]. All structures were solved by direct methods using SHELXT [35] and refined on *F*^2^ using SHELXL-2018 [36]. Positions of all atoms were found from the electron-density difference map. Atoms were refined with individual anisotropic (non-hydrogen atoms) or isotropic (hydrogen atoms) displacement parameters.

Crystal data, data collection and structure refinement details are summarized in Supplementary Materials. The structures were deposited at the Cambridge Crystallographic Data Center with the reference CCDC numbers 2232898-2232902; they also contain the supplementary crystallographic data. These data can be obtained free of charge from the CCDC via https://www.ccdc.cam.ac.uk/structures/ (accessed on 26 December 2022).

## 4. Conclusions

In summary, we have developed an efficient one-step method for the synthesis of pyrazolo [4,3-*b*]pyridines and indazoles on the basis of the modified Japp–Klingemann reaction and intramolecular nucleophilic substitution of the nitro group. The method comprises readily available starting materials, mild reaction conditions, easy work-up and high product yields. A plausible mechanism for the formation of both pyrazolo [4,3-*b*]pyridines and indazoles was proposed. As a result, a wide range of polyfunctional fused pyrazoles was synthesized which can be considered as prospective platforms for the design of pharmacology-oriented heterocyclic systems.

## Data Availability

Not applicable.

## Supplementary Materials

The following supporting information can be downloaded at: https://www.mdpi.com/article/10.3390/ijms24021758/s1. References [37,38] are cited in the supplementary materials.
