# Supplementary material for: Straightforward and Efficient Protocol for the Synthesis of Pyrazolo [4,3-b]pyridines and Indazoles
Source: Int J Mol Sci. 2023 Jan 16;24(2):1758. doi: 10.3390/ijms24021758 (PMC9860909; doi:10.3390/ijms24021758)

Supplementary materials.

Straightforward and efficient protocol for the synthesis of pyrazolo[4,3-b]pyridines and indazoles

Vladislav V. Nikol'skiy, Mikhail E. Minyaev, Maxim A. Bastrakov and Alexey M. Starosotnikov*

N.D. Zelinsky Institute of Organic Chemistry RAS, Leninsky prosp. 47, 11991 Moscow, Russia

E-mail: alexey41@list.ru

X-ray crystallographic data and refinement details.

X-ray diffraction data for **2a**, **5a'**, **5c** and **5q'** were collected at 100K on a Rigaku Synergy S diffractometer equipped with a HyPix6000HE area-detector (kappa geometry, shutterless ω-scan technique), using monochromatized Cu K_{α}-radiation. The intensity data were integrated and analytically corrected for absorption and decay by the CrysAlisPro program[1]. X-ray diffraction data for **2b** were collected at 100K on a Bruker Quest D8 diffractometer equipped with a Photon-III area-detector (shutterless φ- and ω-scan technique), using graphite-monochromatized Mo K_{α}-radiation. The intensity data were integrated by the SAINT program[2] and were corrected for absorption and decay using SADABS.[3] All structures were solved by direct methods using SHELXT[4] and refined on F^2 using SHELXL-2018[5] within the OLEX2 program[6] (for **2a**, **5a'**, **5c** and **5q'**) or the ShelXle program[7] (for **2b**). Positions of all atoms were found from the electron density-difference map. Atoms were refined with individual anisotropic (non-hydrogen atoms) or isotropic (hydrogen atoms) displacement parameters. The disordered fragment in **5a'** was modeled by applying similarity constraints on anisotropic displacement parameters on similar atoms and by constraining similar distances between neighboring atoms to be equal within the deviation of 0.003Å.

Crystal data, data collection and structure refinement details are summarized in Table S1. The structures have been deposited at the Cambridge Crystallographic Data Center with the reference CCDC numbers 2232898-2232902; they also contain the supplementary crystallographic data. These data can be obtained free of charge from the CCDC *via* https://www.ccdc.cam.ac.uk/structures/

References

1. CrysAlisPro. Version 1.171.42. *Rigaku Oxford Diffraction*, **2021**.
2. Bruker. APEX-III. *Bruker AXS Inc.*, Madison, Wisconsin, USA, **2019**.
3. Krause, L.; Herbst-Irmer, R.; Sheldrick, G. M.; Stalke, D. Comparison of silver and molybdenum microfocus X-ray sources for single-crystal structure determination. *J. Appl. Cryst.* **2015**, 48, 3–10. https://doi.org/10.1107/S1600576714022985
4. Sheldrick, G. M. SHELXT - Integrated space-group and crystal-structure determination. *Acta Cryst.* **2015**, A71(1), 3-8. https://doi.org/10.1107/S2053273314026370
5. Sheldrick, G. M. Crystal structure refinement with SHELXL. *Acta Cryst.* **2015**, C71(1), 3-8. https://doi.org/10.1107/S2053229614024218
6. Dolomanov, O.V.; Bourhis L.J.; Gildea R.J.; Howard J.A.K.; Puschmann H. OLEX2: a complete structure solution, refinement and analysis program. *J. Appl. Cryst.* **2009**, 42(2), 339-341. https://doi.org/10.1107/S0021889808042726
7. Hübschle, C.B.; Sheldrick, G. M.; Dittrich, B. ShelXle: a Qt graphical interface for SHELXL. *J. Appl. Cryst.* 2011, **44**, 1281-1284. https://doi.org/10.1107/S0021889811043202

Table S1. Crystal data and structure refinement for **2a**, **2b**, **5a'**, **5c** and **5q'**.

Identification code	**2a**	**2b**	**5a'**	**5c**	**5q'**
Empirical formula	$C_{11}H_{11}N_3O_7$	$C_{12}H_{11}F_3N_2O_5$	$C_{18}H_{14}N_6O_7$	$C_{17}H_{14}N_4O_6$	$C_{19}H_{17}FN_4O_7$
Formula weight	297.23	320.23	426.35	370.32	432.36
Temperature, K	100.0(1)	100(2)	100.0(1)	100.0(1)	100.0(1)
Wavelength, Å	1.54184	0.71073	1.54184	1.54184	1.54184
Crystal system	Triclinic	Orthorhombic	Triclinic	Triclinic	Triclinic
Space group	$P\bar{1}$	$P2_12_12_1$	$P\bar{1}$	P1	$P\bar{1}$
Unit cell dimensions					
a, Å	7.74419(6)	8.09850(10)	9.04170(10)	4.89510(10)	10.54644(11)
b, Å	10.79552(10)	10.8223(2)	9.63200(10)	9.4778(2)	12.45588(15)
c, Å	16.71942(16)	15.0333(3)	12.3591(2)	9.8235(2)	16.4948(2)
α, °	78.7491(8)	90.0	73.5870(10)	116.202(2)	79.7936(11)
β, °	78.6511(7)	90.0	85.1740(10)	92.5690(10)	80.1675(10)
γ, °	70.7659(8)	90.0	69.2910(10)	96.3260(10)	68.9608(10)
Volume, $Å^3$	1281.08(2)	1317.58(4)	965.63(2)	404.198(16)	1976.80(4)
Z	4	4	2	1	4
Calcd density, $g\cdot cm^{-3}$	1.541	1.614	1.466	1.521	1.453
μ, mm^{-1}	1.138	0.152	0.993	1.003	1.019
F(000)	616	656	440	192	896
Crystal size, mm	0.71×0.40×0.33	0.50×0.29×0.24	0.11×0.08×0.06	0.41×0.24×0.09	0.21×0.13×0.11
θ range, °	2.723-7.850	2.319-33.740	3.729-77.897	5.046-77.748	2.741-77.857
Complentess θ_{full} / θ_{max}	0.999 / 0.992	0.999 / 1.000	0.999 / 0.989	1.000 / 0.984	1.000 / 0.992
Index ranges	$-8 \le h \le 9$,<br>$-13 \le k \le 13$,<br>$-21 \le l \le 21$	$-12 \le h \le 12$,<br>$-16 \le k \le 16$,<br>$-23 \le l \le 23$	$-11 \le h \le 11$,<br>$-12 \le k \le 12$,<br>$-15 \le l \le 15$	$-6 \le h \le 5$,<br>$-11 \le k \le 11$,<br>$-12 \le l \le 12$	$-13 \le h \le 11$,<br>$-15 \le k \le 15$,<br>$-20 \le l \le 20$
Reflections					
collected	32895	48435	21004	16296	52168
independent [R_{int}]	5411 [0.0386]	5269 [0.0392]	4097 [0.0320]	3174 [0.0402]	8381 [0.0268]
observed, I>2σ(I)	5309	4804	3844	3160	7985
T_{max} / T_{min}	0.802 / 0.603	0.6957 / 0.6630	0.956 / 0.934	0.773 / 0.451	0.927 / 0.869
Data / restr. / param.	5411 / 0 / 466	5269 / 0 / 243	4097 / 4 / 346	3174 / 3 / 300	8381 / 0 / 696
GooF on F^2	1.063	1.064	1.025	1.085	1.038
R1/wR2 (I>2σ(I)]	0.0362 / 0.0953	0.0320 / 0.0772	0.0416 / 0.1133	0.0284 / 0.0773	0.0314 / 0.0841
R1/wR2 (all data)	0.0368 / 0.0958	0.0378 / 0.0811	0.0434 / 0.1148	0.0284 / 0.0773	0.0326 / 0.0851
Extinction coeff.	0.0057(4)	-	-	-	0.00088(11)
Flack x	-	0.03(17)	-	0.15(9)	-
$\Delta\rho_{max}$ / $\Delta\rho_{min}$, $\bar{e}\cdot Å^{-3}$	0.350 / -0.243	0.330 / -0.227	0.360 / -0.295	0.165 / -0.194	0.297 / -0.196
CCDC number	2232898	2232899	2232900	2232901	2232902

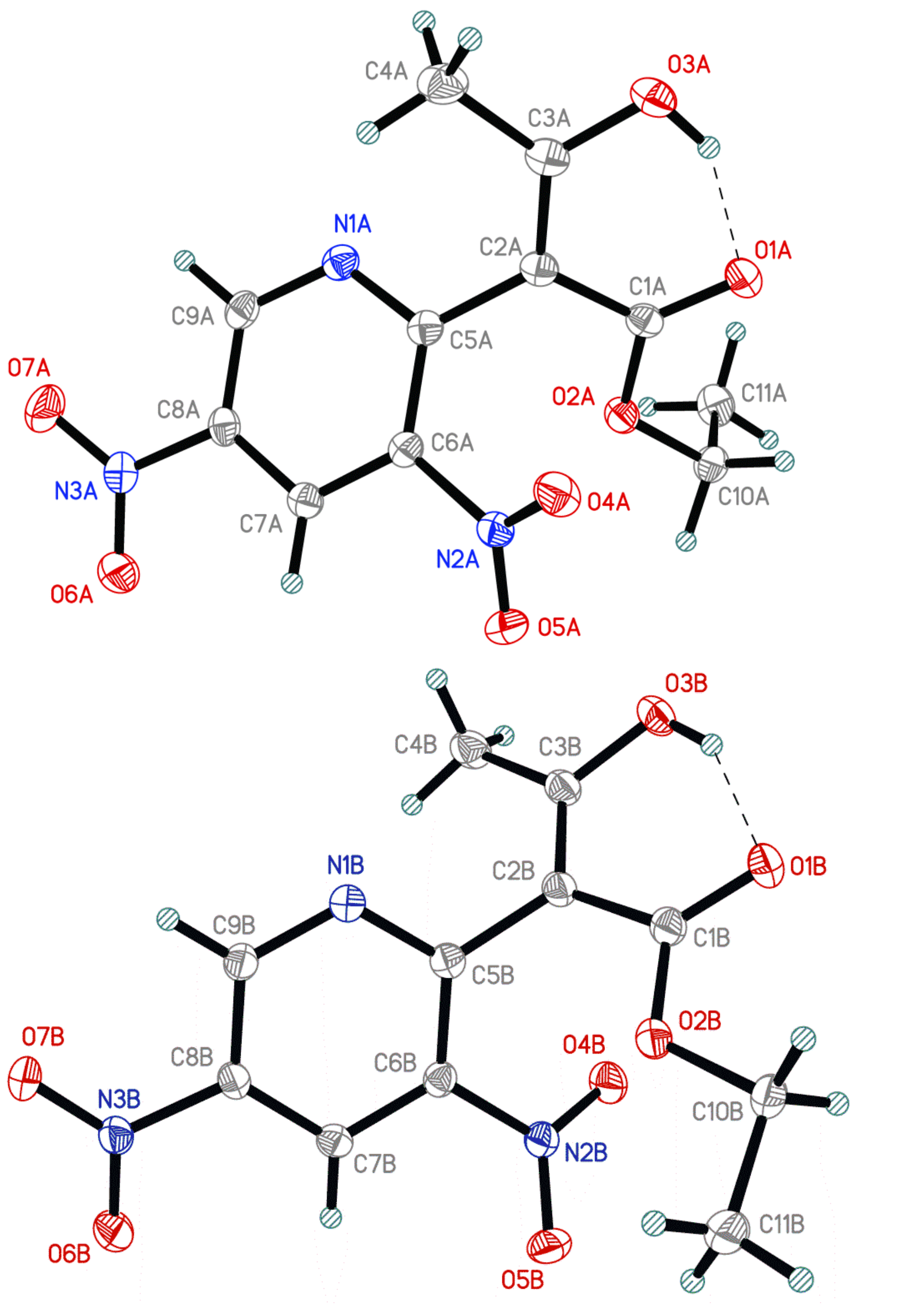

Figure S1. Structures of two crystallographically non-equivalent molecules in crystals of **2a**. A thermal ellipsoids probability level is set to 50%.

Table S2. Selected bond distances for **2a**, Å

O1A-C1A	1.2317(15)	O7A-N3A	1.2242(15)	C2A-C5A	1.4790(16)
O2A-C1A	1.3290(14)	N1A-C5A	1.3454(15)	C3A-C4A	1.4917(18)
O2A-C10A	1.4609(14)	N1A-C9A	1.3333(16)	C5A-C6A	1.4061(16)
O3A-C3A	1.3312(15)	N2A-C6A	1.4671(14)	C6A-C7A	1.3779(16)
O4A-N2A	1.2273(14)	N3A-C8A	1.4649(15)	C7A-C8A	1.3802(17)
O5A-N2A	1.2249(14)	C1A-C2A	1.4634(16)	C8A-C9A	1.3900(17)

O6A-N3A	1.2252(15)	C2A-C3A	1.3719(16)	C10A-C11A	1.5042(17)
O1B-C1B	1.2289(15)	O7B-N3B	1.2258(14)	C2B-C5B	1.4826(15)
O2B-C1B	1.3330(15)	N1B-C5B	1.3479(15)	C3B-C4B	1.4912(17)
O2B-C10B	1.4585(14)	N1B-C9B	1.3338(15)	C5B-C6B	1.4026(16)
O3B-C3B	1.3402(14)	N2B-C6B	1.4718(14)	C6B-C7B	1.3836(16)
O4B-N2B	1.2244(13)	N3B-C8B	1.4627(14)	C7B-C8B	1.3785(16)
O5B-N2B	1.2238(14)	C1B-C2B	1.4617(16)	C8B-C9B	1.3880(16)
O6B-N3B	1.2274(14)	C2B-C3B	1.3598(17)	C10B-C11B	1.5027(18)

Table S2. Hydrogen bond parameters for **2a**, Å and °.

D-H...A	d(D-H)	d(H...A)	d(D...A)	<(DHA)
O3A-H3A...O1A	0.91(2)	1.72(2)	2.5483(13)	150.9(18)
O3B-H3B...O1B	0.87(2)	1.78(2)	2.5745(13)	150.1(18)

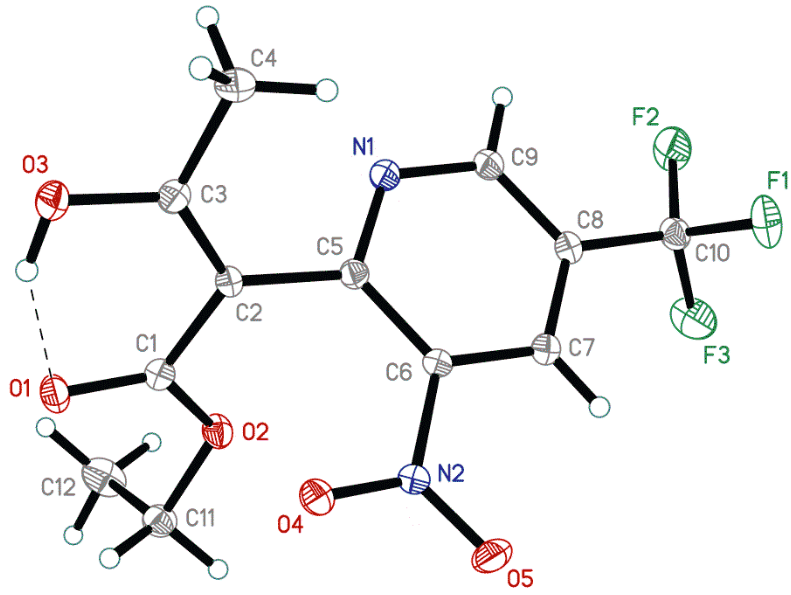

Figure S2. The crystal structure of **2b** (p=50%).

Table S3. Selected bond distances for **2b**, Å

O1-C1	1.2317(16)	C5-N1	1.3510(16)	C9-N1	1.3389(17)
O2-C1	1.3336(16)	C5-C6	1.4102(17)	N2-O4	1.2234(14)
O2-C11	1.4655(16)	C6-C7	1.3824(17)	N2-O5	1.2273(15)
C1-C2	1.4626(18)	C6-N2	1.4779(16)	C10-F1	1.3371(16)
C2-C3	1.3696(18)	C7-C8	1.3898(18)	C10-F2	1.3379(16)
C2-C5	1.4776(17)	C8-C9	1.3869(18)	C10-F3	1.3382(17)
C3-O3	1.3346(16)	C8-C10	1.4979(18)	C11-C12	1.505(2)
C3-C4	1.4929(19)				

Table S4. Hydrogen bond parameters for **2b**, Å and °.

D-H...A	d(D-H)	d(H...A)	d(D...A)	<(DHA)
O3-H3...O1	0.90(3)	1.74(3)	2.5674(15)	151(3)
O3-H3...O5#1	0.90(3)	2.45(3)	2.9027(16)	111(2)

Symmetry transformation used to generate equivalent atoms: #1 -x+3/2, -y+1, +z+1/2.

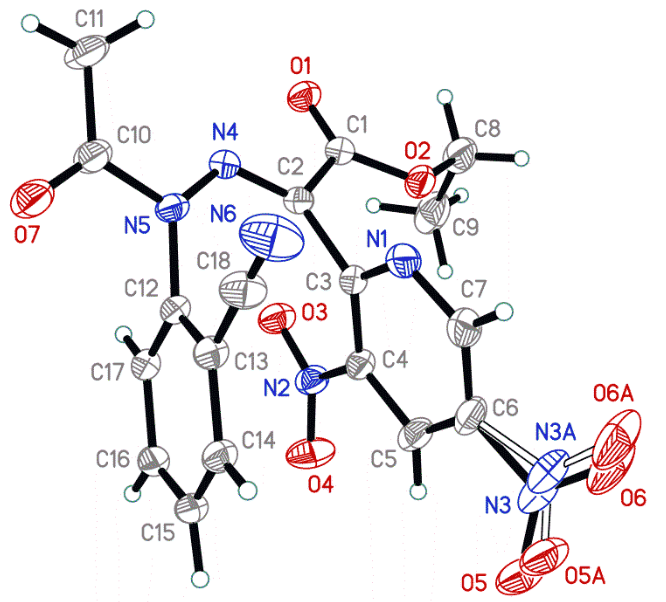

Figure S3. The crystal structure of **5a'** (p=50%). The disorder ratio for atoms N3, O5, O6 and N3A, O5A, O6A is 0.786(14):0.214(14).

Table S5. Selected bond distances for **5a'**, Å

C1-O1	1.2024(16)	C6-N3	1.4732(19)	N5-C12	1.4409(15)
C1-O2	1.3347(16)	C6-N3A	1.477(3)	C10-O7	1.2126(18)
C1-C2	1.5096(18)	C7-N1	1.3367(19)	C10-C11	1.4972(19)
O2-C8	1.4677(18)	N2-O3	1.2266(15)	C12-C13	1.3996(18)
C2-C3	1.5038(17)	N2-O4	1.2260(15)	C12-C17	1.3817(18)
C2-N4	1.2819(17)	N3-O5	1.227(3)	C13-C14	1.3976(18)
C3-C4	1.4008(18)	N3-O6	1.209(3)	C13-C18	1.4396(19)
C3-N1	1.3355(17)	N3A-O5A	1.228(4)	C14-C15	1.387(2)
C4-C5	1.3831(18)	N3A-O6A	1.211(4)	C15-C16	1.390(2)
C4-N2	1.4645(17)	C8-C9	1.499(3)	C16-C17	1.3940(18)
C5-C6	1.375(2)	N4-N5	1.3737(15)	C18-N6	1.147(2)
C6-C7	1.382(2)	N5-C10	1.3984(17)		

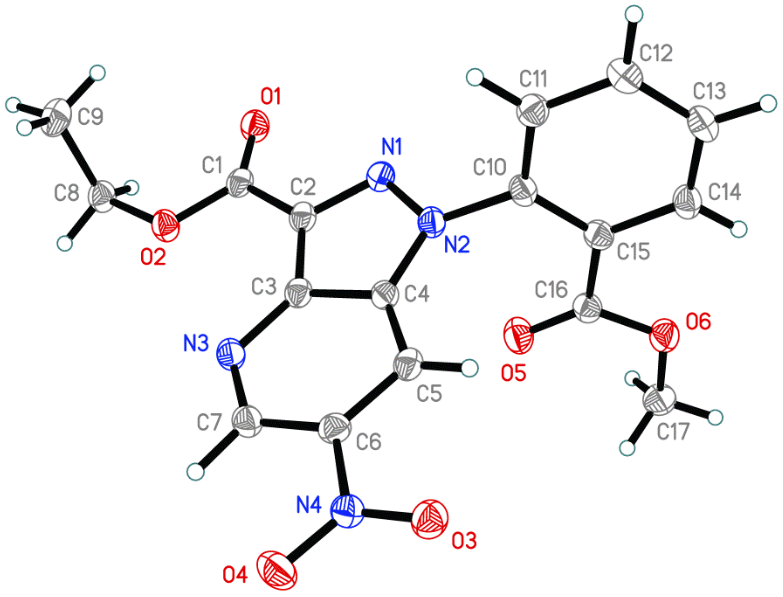

Figure S4. The crystal structure of **5c** (p=50%).

Table S6. Selected bond distances for **5c**, Å

O1-C1	1.206(3)	N2-C4	1.372(3)	C6-C7	1.414(3)
O2-C1	1.340(3)	N2-C10	1.438(3)	C8-C9	1.505(3)
O2-C8	1.460(3)	N3-C3	1.356(3)	C10-C11	1.385(3)
O3-N4	1.228(3)	N3-C7	1.324(3)	C10-C15	1.406(3)
O4-N4	1.229(2)	N4-C6	1.465(3)	C11-C12	1.393(3)
O5-C16	1.211(3)	C1-C2	1.481(3)	C12-C13	1.385(3)
O6-C16	1.334(3)	C2-C3	1.424(3)	C13-C14	1.387(3)
O6-C17	1.448(3)	C3-C4	1.408(3)	C14-C15	1.400(3)
N1-N2	1.345(3)	C4-C5	1.393(3)	C15-C16	1.493(3)
N1-C2	1.334(3)	C5-C6	1.378(3)		

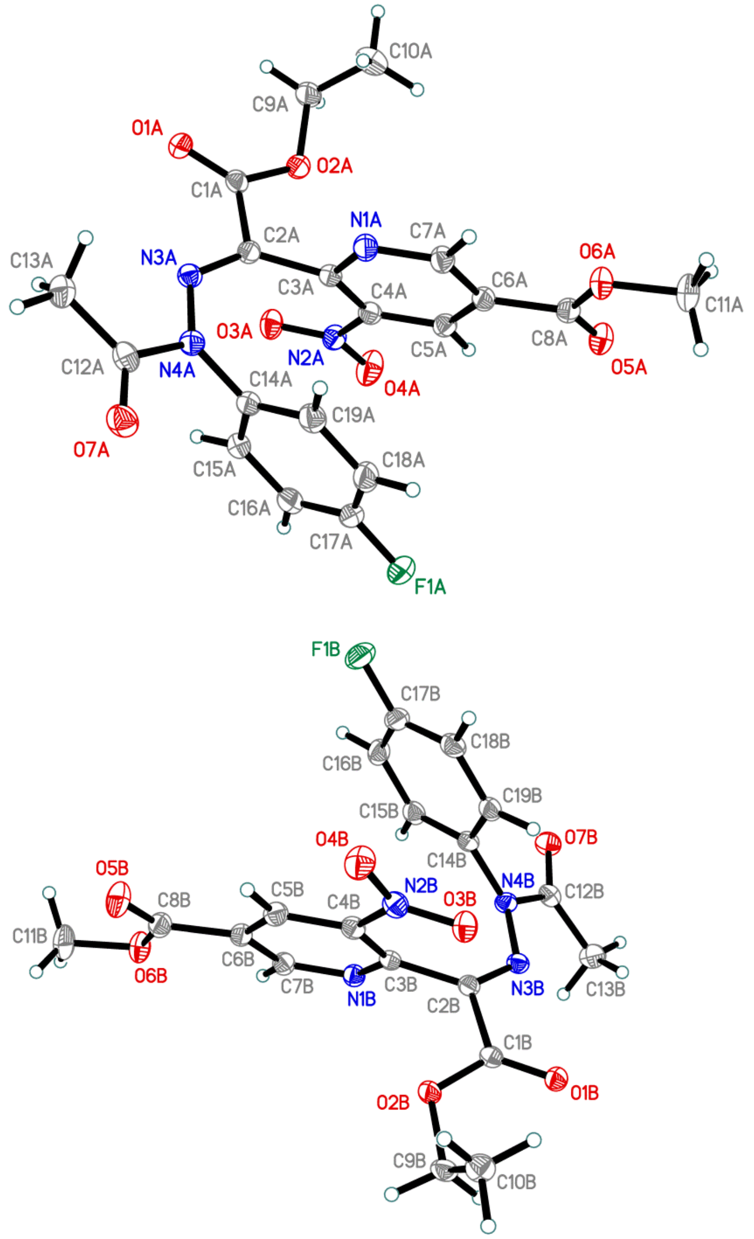

Figure S5. The crystal structure of **5q'** (p=50%). Two crystallographically non-equivalent molecules are shown.

Table S7. Selected bond distances for **5q'**, Å

F1A-C17A	1.3621(12)	N1A-C7A	1.3366(13)	C6A-C7A	1.3909(14)
O1A-C1A	1.2036(12)	N2A-C4A	1.4668(13)	C6A-C8A	1.4971(13)
O2A-C1A	1.3291(12)	N3A-N4A	1.3725(11)	C9A-C10A	1.4972(16)
O2A-C9A	1.4602(12)	N3A-C2A	1.2817(13)	C12A-C13A	1.5034(15)
O3A-N2A	1.2270(11)	N4A-C12A	1.3998(13)	C14A-C15A	1.3864(15)
O4A-N2A	1.2228(12)	N4A-C14A	1.4428(13)	C14A-C19A	1.3906(14)
O5A-C8A	1.2051(13)	C1A-C2A	1.5086(13)	C15A-C16A	1.3895(15)
O6A-C8A	1.3315(12)	C2A-C3A	1.5060(13)	C16A-C17A	1.3771(17)
O6A-C11A	1.4532(12)	C3A-C4A	1.3965(14)	C17A-C18A	1.3778(19)

O7A-C12A	1.2125(13)	C4A-C5A	1.3839(14)	C18A-C19A	1.3900(16)
N1A-C3A	1.3398(13)	C5A-C6A	1.3867(14)		
F1B-C17B	1.3630(12)	N1B-C7B	1.3384(12)	C6B-C7B	1.3914(14)
O1B-C1B	1.2032(12)	N2B-C4B	1.4664(12)	C6B-C8B	1.4963(13)
O2B-C1B	1.3315(11)	N3B-N4B	1.3797(11)	C9B-C10B	1.5069(14)
O2B-C9B	1.4670(11)	N3B-C2B	1.2812(12)	C12B-C13B	1.5011(14)
O3B-N2B	1.2287(11)	N4B-C12B	1.3996(12)	C14B-C15B	1.3897(14)
O4B-N2B	1.2252(11)	N4B-C14B	1.4403(12)	C14B-C19B	1.3907(14)
O5B-C8B	1.2035(13)	C1B-C2B	1.5089(12)	C15B-C16B	1.3925(15)
O6B-C8B	1.3312(13)	C2B-C3B	1.5007(12)	C16B-C17B	1.3773(16)
O6B-C11B	1.4558(12)	C3B-C4B	1.3966(13)	C17B-C18B	1.3783(16)
O7B-C12B	1.2149(12)	C4B-C5B	1.3829(13)	C18B-C19B	1.3885(14)
N1B-C3B	1.3384(13)	C5B-C6B	1.3860(14)		

NMR spectra of synthesized compounds.

^{1}H and ^{13}C NMR spectra of compound 2**a** in $CDCl_3$

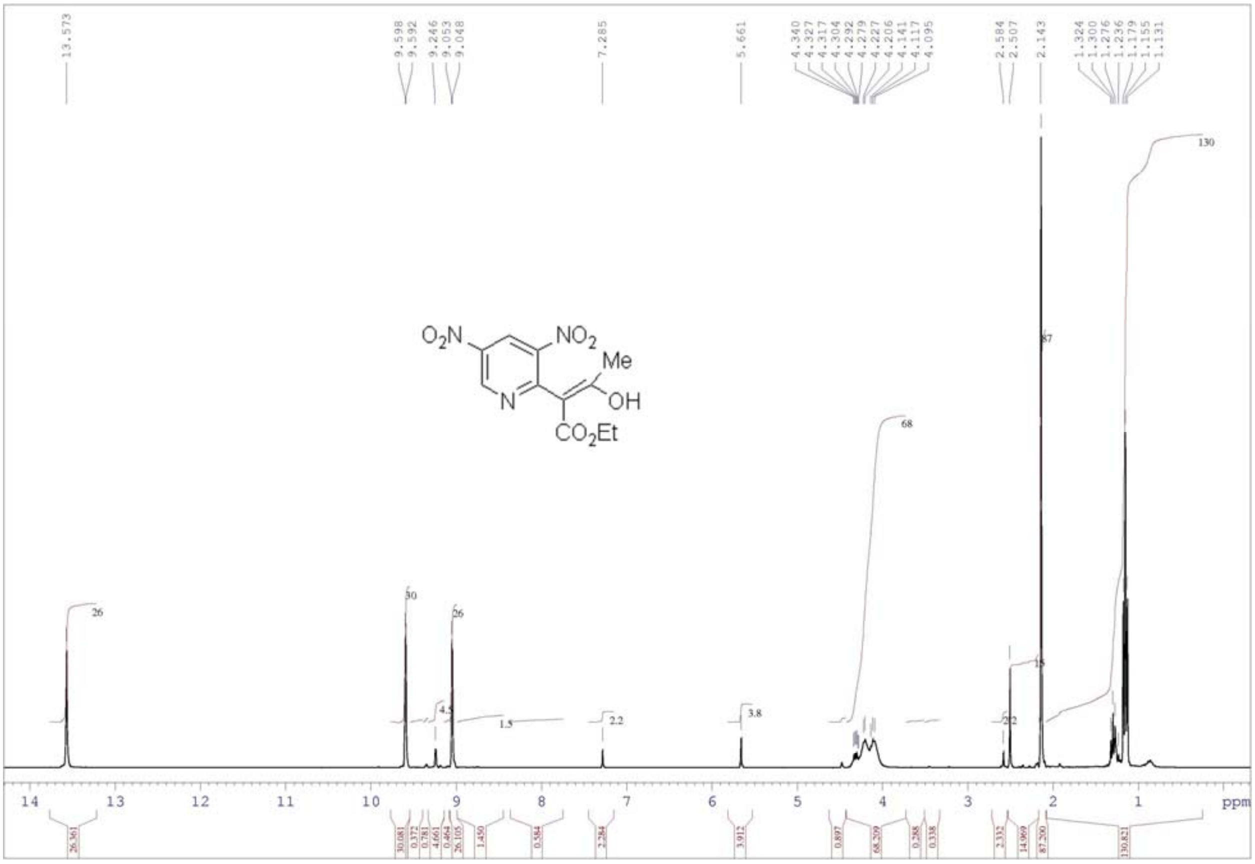

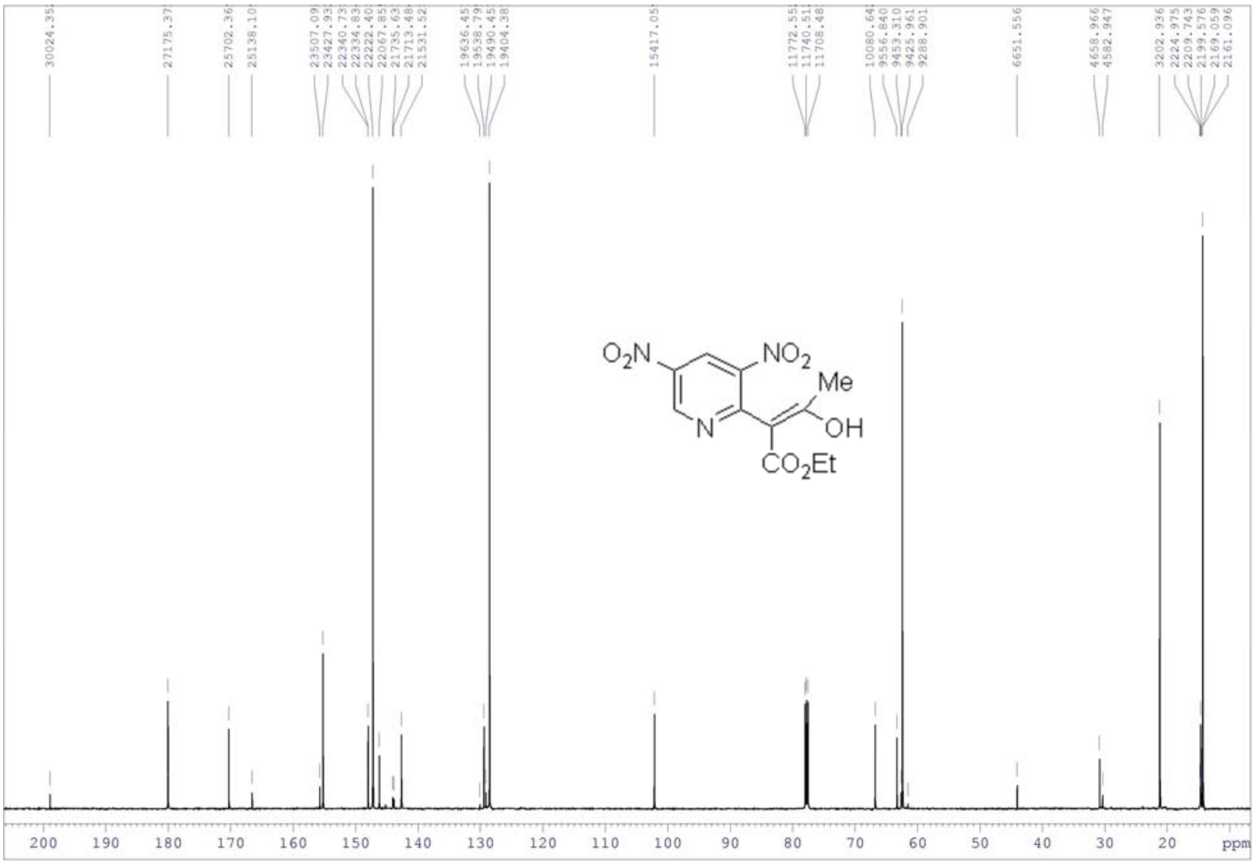

HRMS spectrum of compound **2a**

Display Report

Analysis Info

Analysis Name	D:\Data\Kolotyrkina\2022\Bastrakov\0412023.d	Acquisition Date	12.04.2022 14:37:29
Method	tune_50-1600_pos_15_12.m	Operator	BDAL@DE
Sample Name	/LPIK VN-497	Instrument / Ser#	micrOTOF 10248
Comment	C11H11N3O7 mH 298.0669 clb added CH3CN		

Acquisition Parameter

Source Type	ESI	Ion Polarity	Positive	Set Nebulizer	0.4 Bar
Focus	Not active			Set Dry Heater	180 °C
Scan Begin	50 m/z	Set Capillary	4500 V	Set Dry Gas	4.0 l/min
Scan End	1600 m/z	Set End Plate Offset	-500 V	Set Divert Valve	Waste

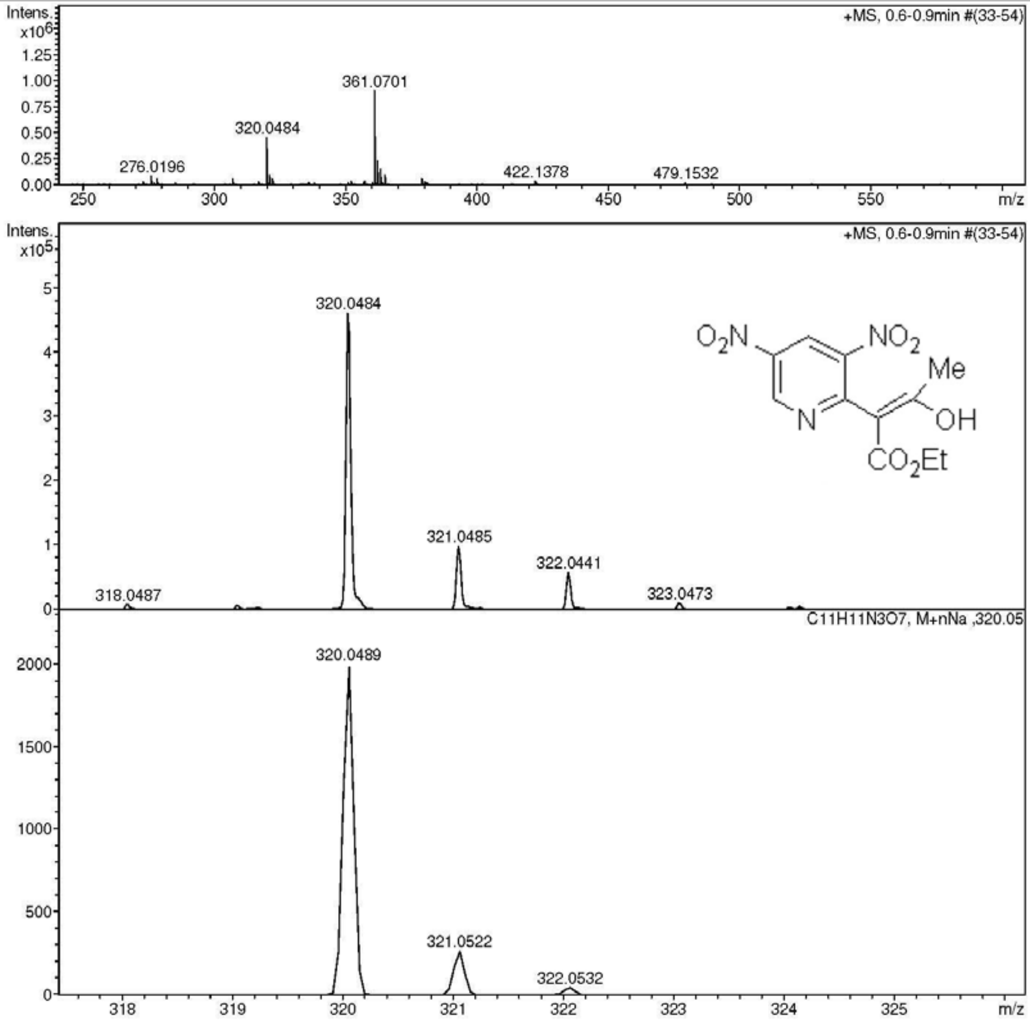

^{1}H spectrum of compound **2b** in $CDCl_3$

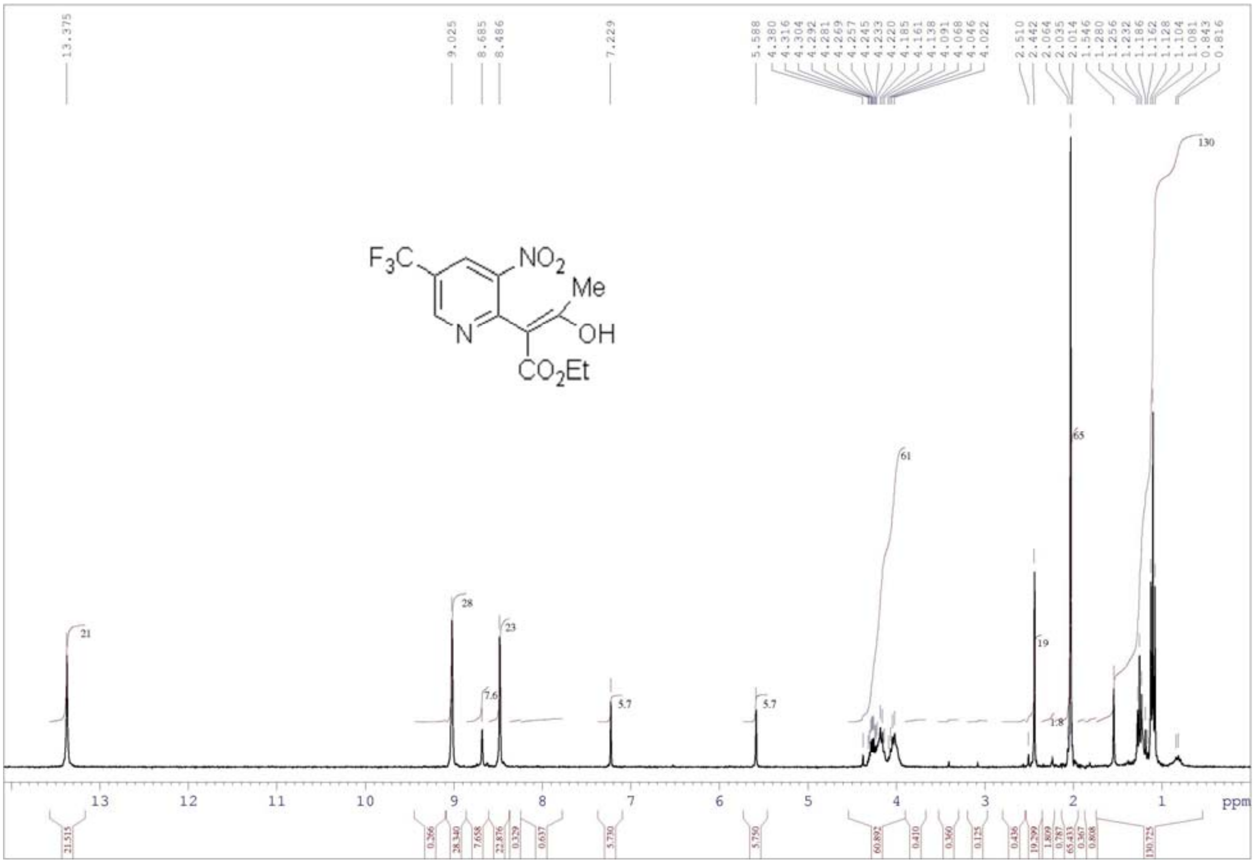

HRMS spectrum of compound **2b**

Display Report

Analysis Info

Analysis Name	D:\Data\Kolotyrkina\2022\Bastrakov\1221002.d	Acquisition Date	21.12.2022 9:40:14
Method	tune_low.m	Operator	BDAL@DE
Sample Name	/LPIK VN-583	Instrument / Ser#	micrOTOF 10248
Comment	C12H11F3N2O5 mH 321.0692 calibrant added CH3CN		

Acquisition Parameter

Source Type	ESI	Ion Polarity	Positive	Set Nebulizer	0.4 Bar
Focus	Not active			Set Dry Heater	180 °C
Scan Begin	50 m/z	Set Capillary	4500 V	Set Dry Gas	4.0 l/min
Scan End	2500 m/z	Set End Plate Offset	-500 V	Set Divert Valve	Waste

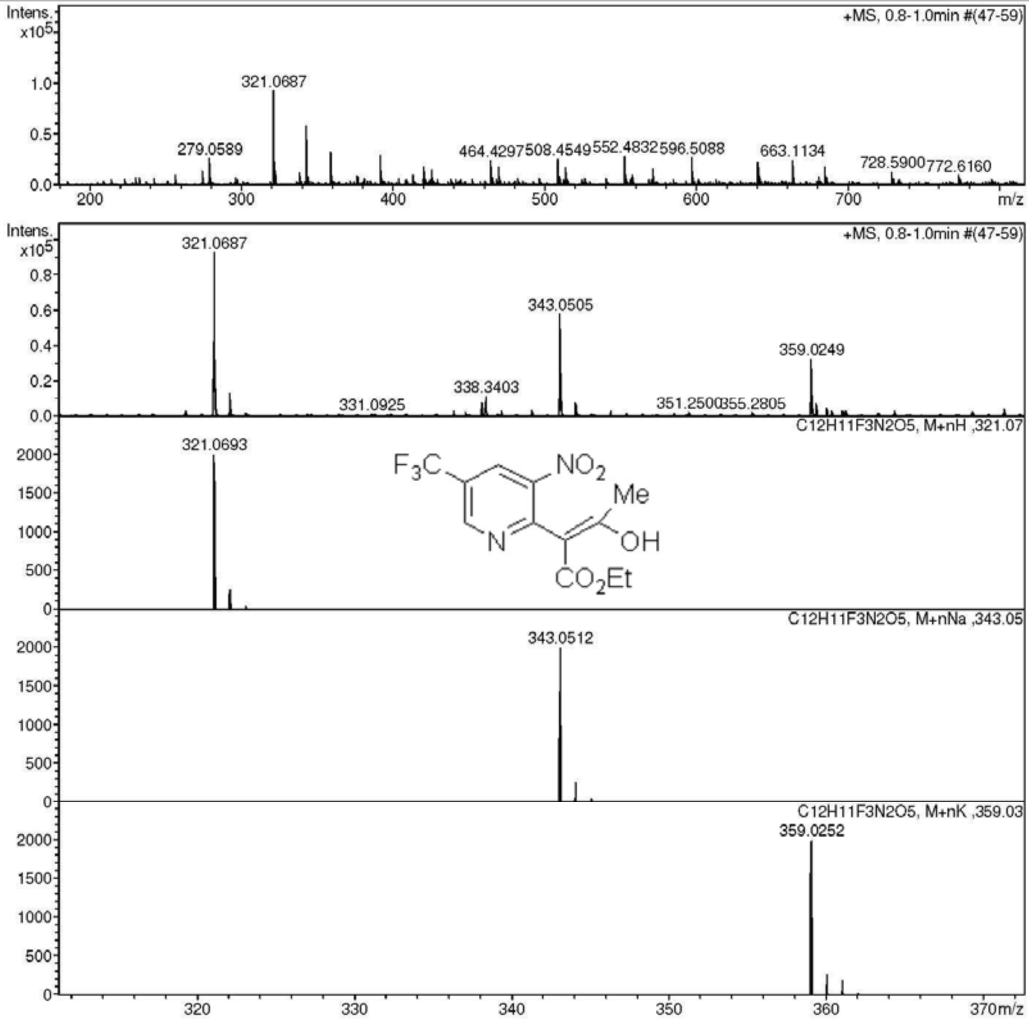

^{1}H spectrum of compound **2c** in $CDCl_3$

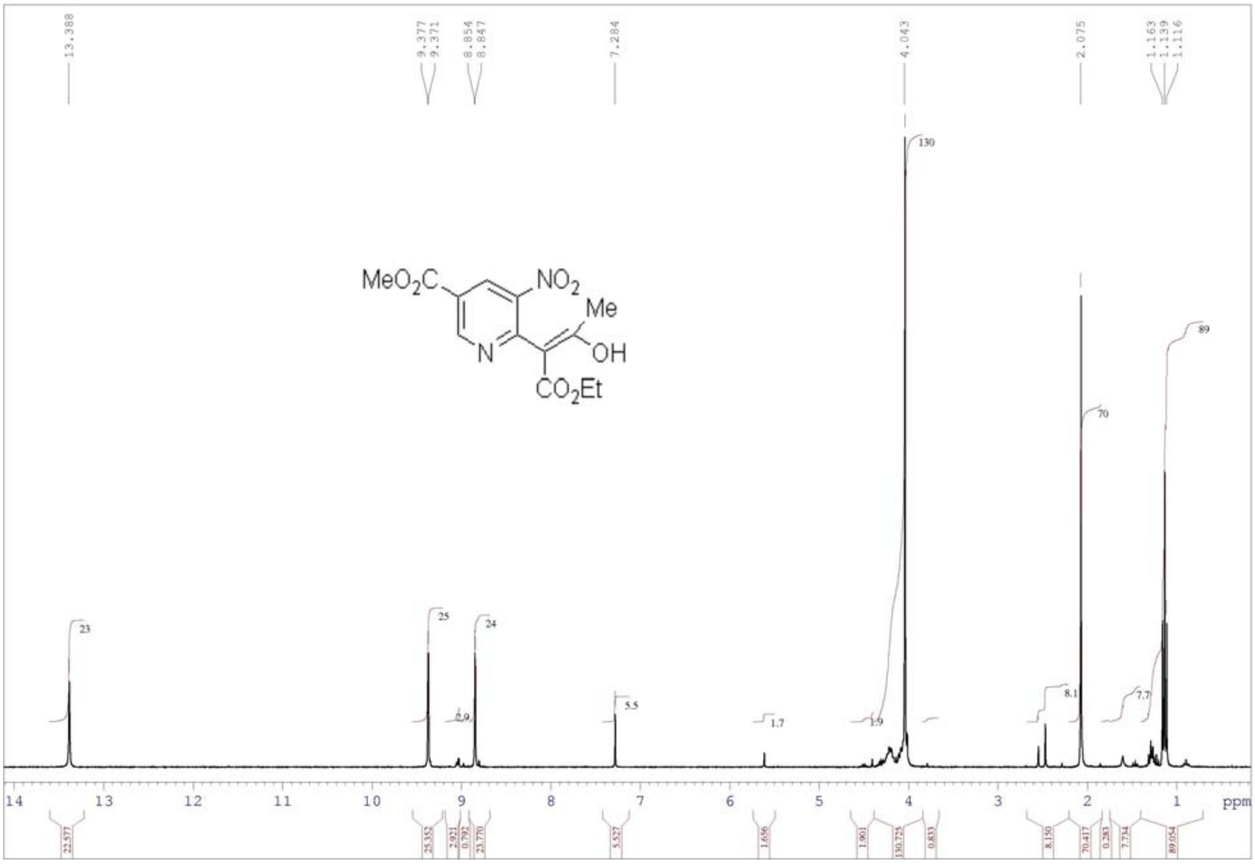

HRMS spectrum of compound **2c**

Display Report

Analysis Info

Analysis Name	D:\Data\Kolotyrkina\2022\Bastrakov\1221003.d	Acquisition Date	21.12.2022 9:48:09
Method	tune_low.m	Operator	BDAL@DE
Sample Name	/LPIK VN-652	Instrument / Ser#	micrOTOF 10248
Comment	C13H14N2O7 mH 311.0873 calibrant added CH3CN		

Acquisition Parameter

Source Type	ESI	Ion Polarity	Positive	Set Nebulizer	0.4 Bar
Focus	Not active			Set Dry Heater	180 °C
Scan Begin	50 m/z	Set Capillary	4500 V	Set Dry Gas	4.0 l/min
Scan End	2500 m/z	Set End Plate Offset	-500 V	Set Divert Valve	Waste

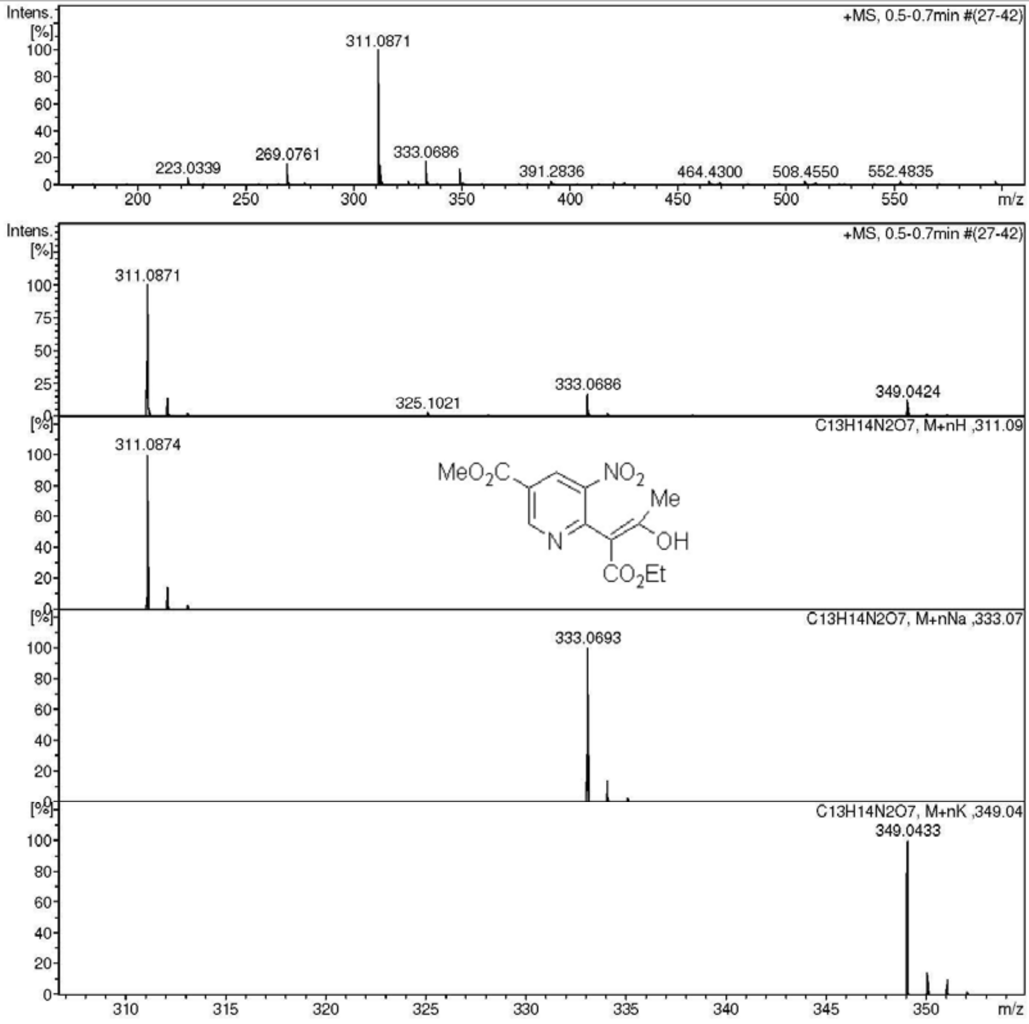

Bruker Compass DataAnalysis 4.0 printed: 21.12.2022 9:50:59 Page 1 of 1

^1H NMR spectrum of compound **4a** in $CDCl_3$

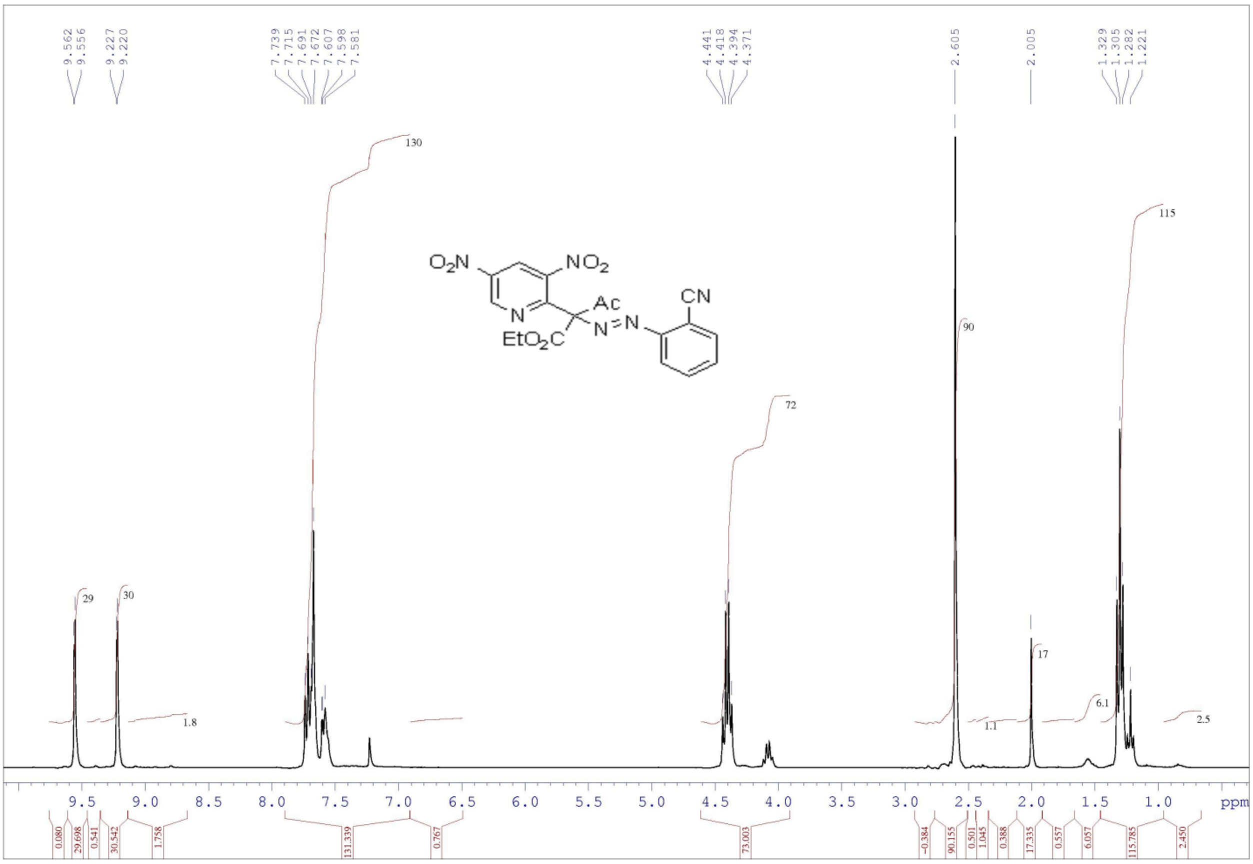

HRMS spectrum of compound **4a**

Display Report

Analysis Info

Analysis Name	D:\Data\Chizhov\Dalinger\Nikol'skiy\vn-520_&clblow.d
Method	tune_low.m
Sample Name	/LPIK VN-520
Comment	CH3OH 100 %, dil. 200, calibrant added
Acquisition Date	24.01.2022 17:23:05
Operator	BDAL@DE
Instrument / Ser#	micrOTOF 10248

Acquisition Parameter

Source Type	ESI	Ion Polarity	Positive	Set Nebulizer	0.4 Bar
Focus	Not active			Set Dry Heater	180 °C
Scan Begin	50 m/z	Set Capillary	4500 V	Set Dry Gas	4.0 l/min
Scan End	3000 m/z	Set End Plate Offset	-500 V	Set Divert Valve	Waste

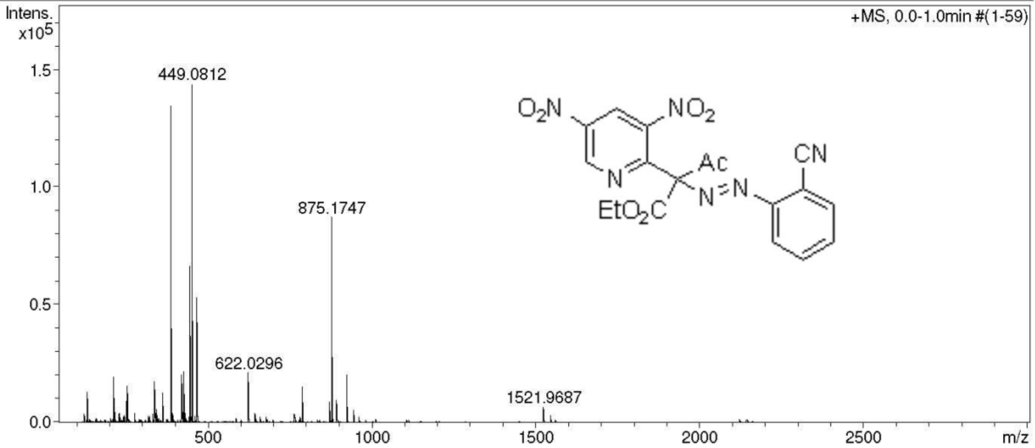

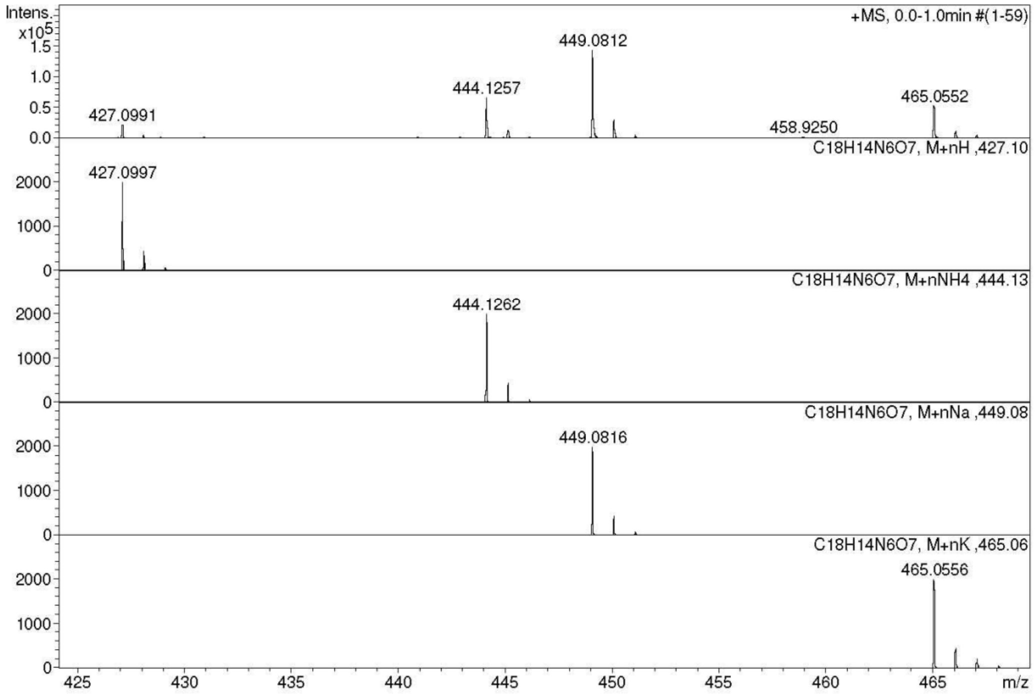

^{1}H and ^{13}C NMR spectra of compound **5a** in $CDCl_3$

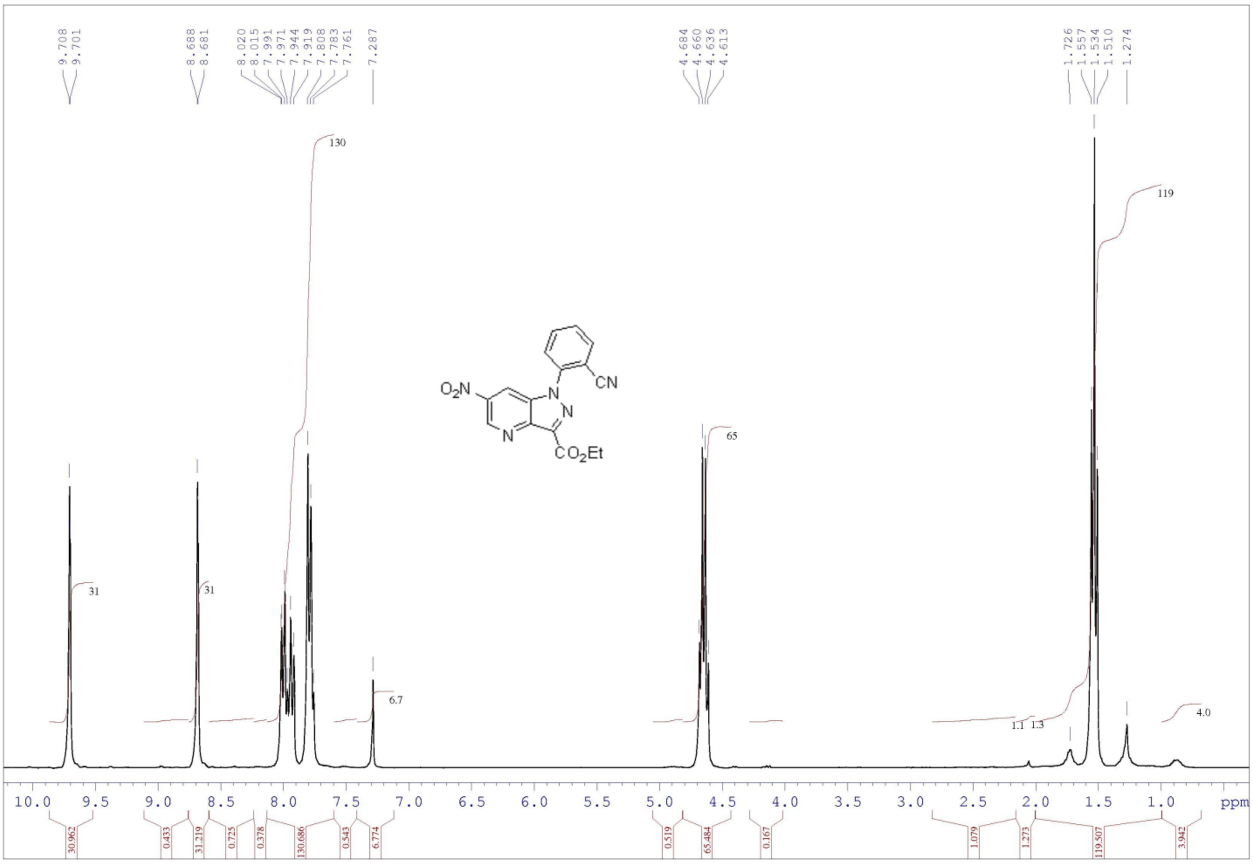

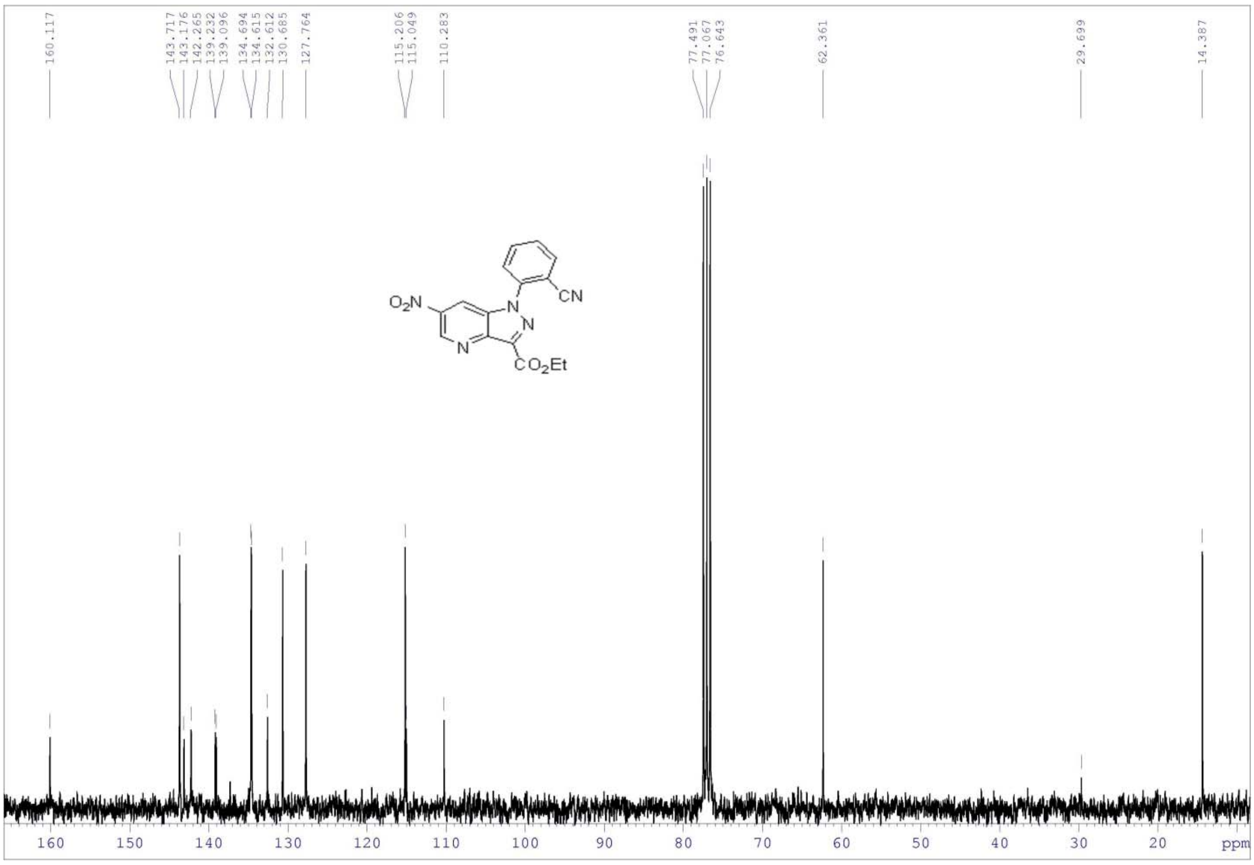

HRMS spectrum of compound **5a**

Display Report

Analysis Info

Analysis Name	D:\Data\Chizhov\Dalinger\Nikol'skiy\vn-517_&clblow.d	Acquisition Date	24.01.2022 17:16:16
Method	tune_low.m	Operator	BDAL@DE
Sample Name	/LPIK VN-517	Instrument / Ser#	micrOTOF 10248
Comment	CH3OH 100 %, dil. 200, calibrant added		

Acquisition Parameter

Source Type	ESI	Ion Polarity	Positive	Set Nebulizer	0.4 Bar
Focus	Not active			Set Dry Heater	180 °C
Scan Begin	50 m/z	Set Capillary	4500 V	Set Dry Gas	4.0 l/min
Scan End	3000 m/z	Set End Plate Offset	-500 V	Set Divert Valve	Waste

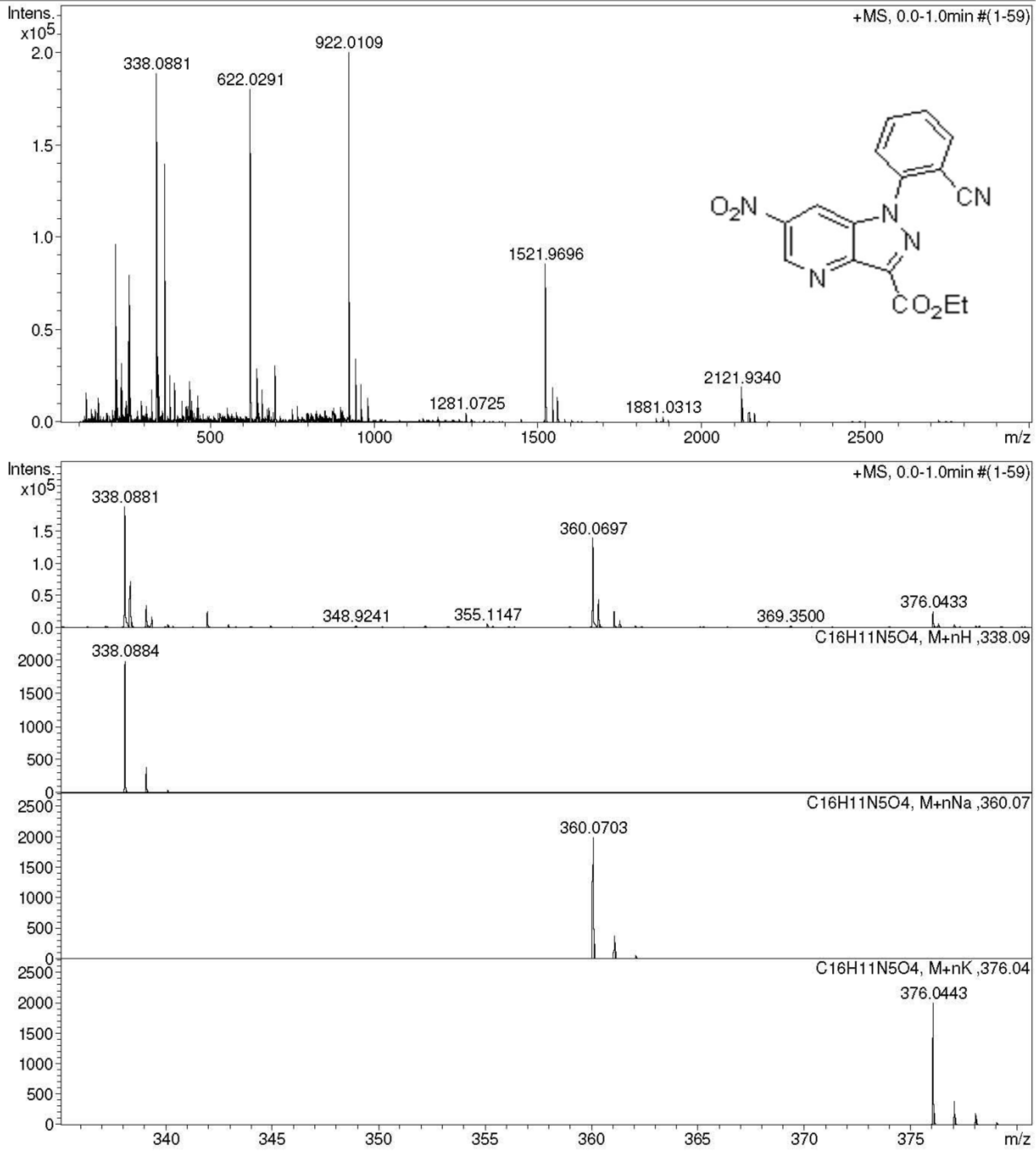

^{1}H NMR spectrum of compound **5a'** in $CDCl_3$

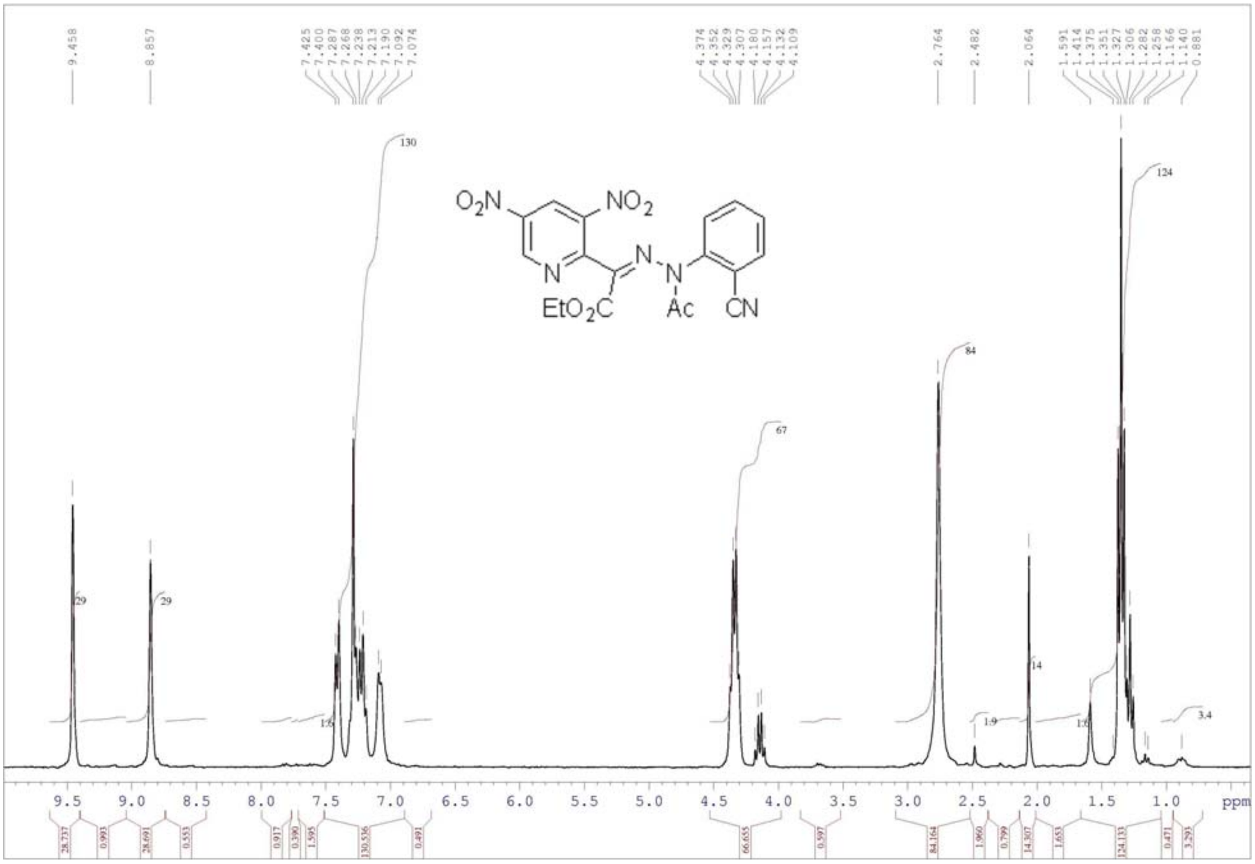

HRMS spectrum of compound 5a'

Display Report

Analysis Info

Analysis Name	D:\Data\Chizhov\Dalinger\Nikol'skiy\vn-519_&clblow.d	Acquisition Date	24.01.2022 17:20:03
Method	tune_low.m	Operator	BDAL@DE
Sample Name	/LPIK VN-519	Instrument / Ser#	micrOTOF 10248
Comment	CH3OH 100 %, dil. 200, calibrant added		

Acquisition Parameter

Source Type	ESI	Ion Polarity	Positive	Set Nebulizer	0.4 Bar
Focus	Not active			Set Dry Heater	180 °C
Scan Begin	50 m/z	Set Capillary	4500 V	Set Dry Gas	4.0 l/min
Scan End	3000 m/z	Set End Plate Offset	-500 V	Set Divert Valve	Waste

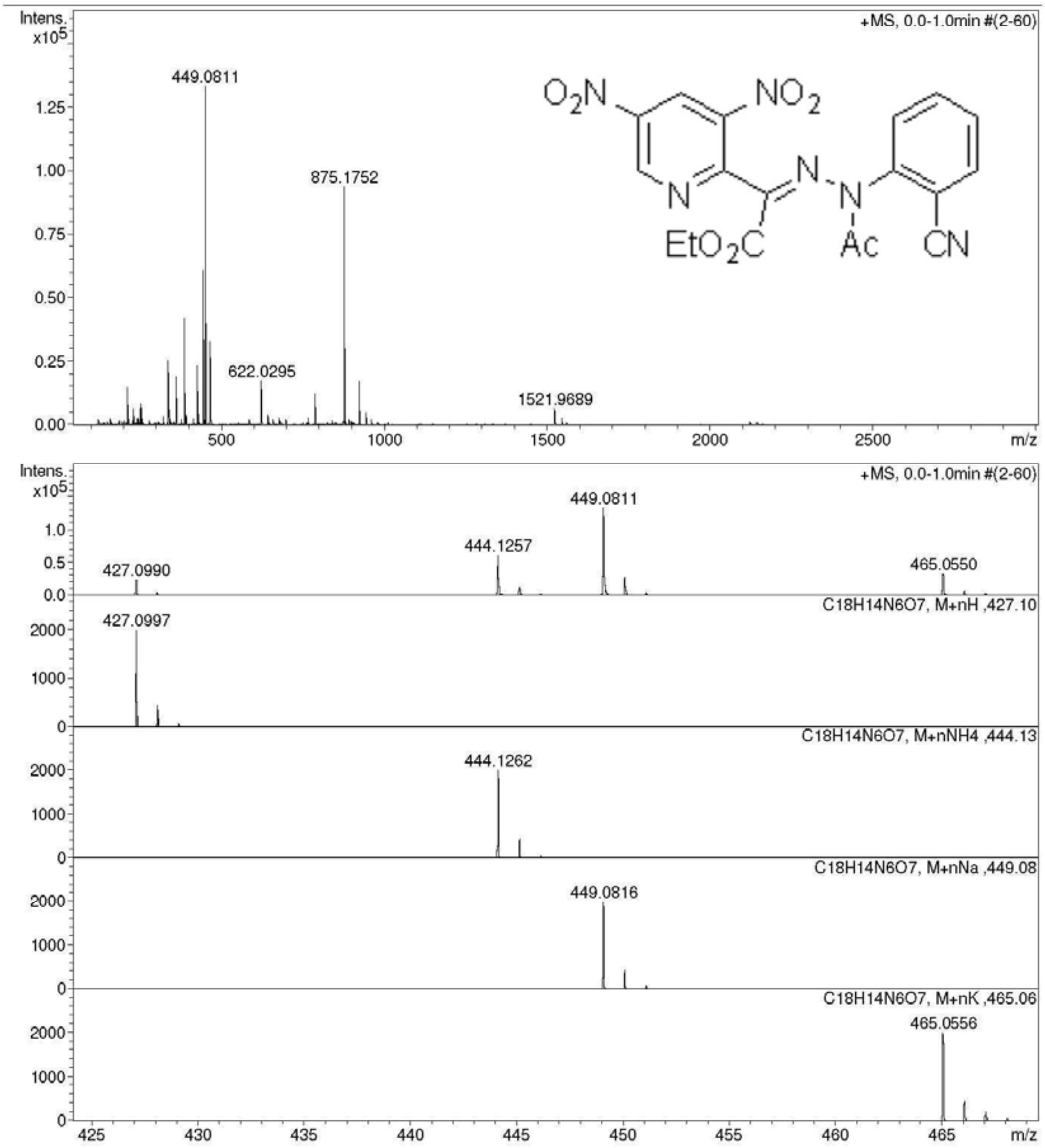

^{1}H and ^{13}C NMR spectra of compound **5b** in $CDCl_3$

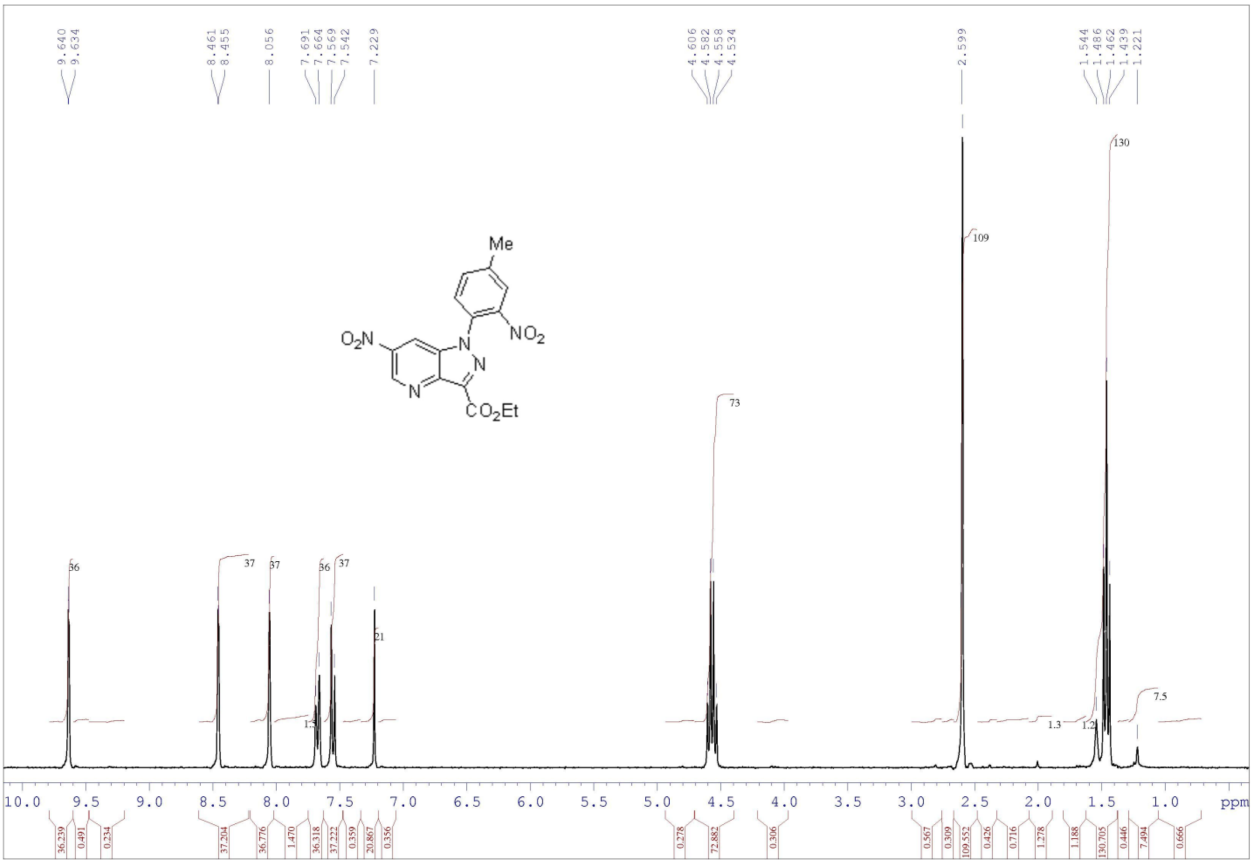

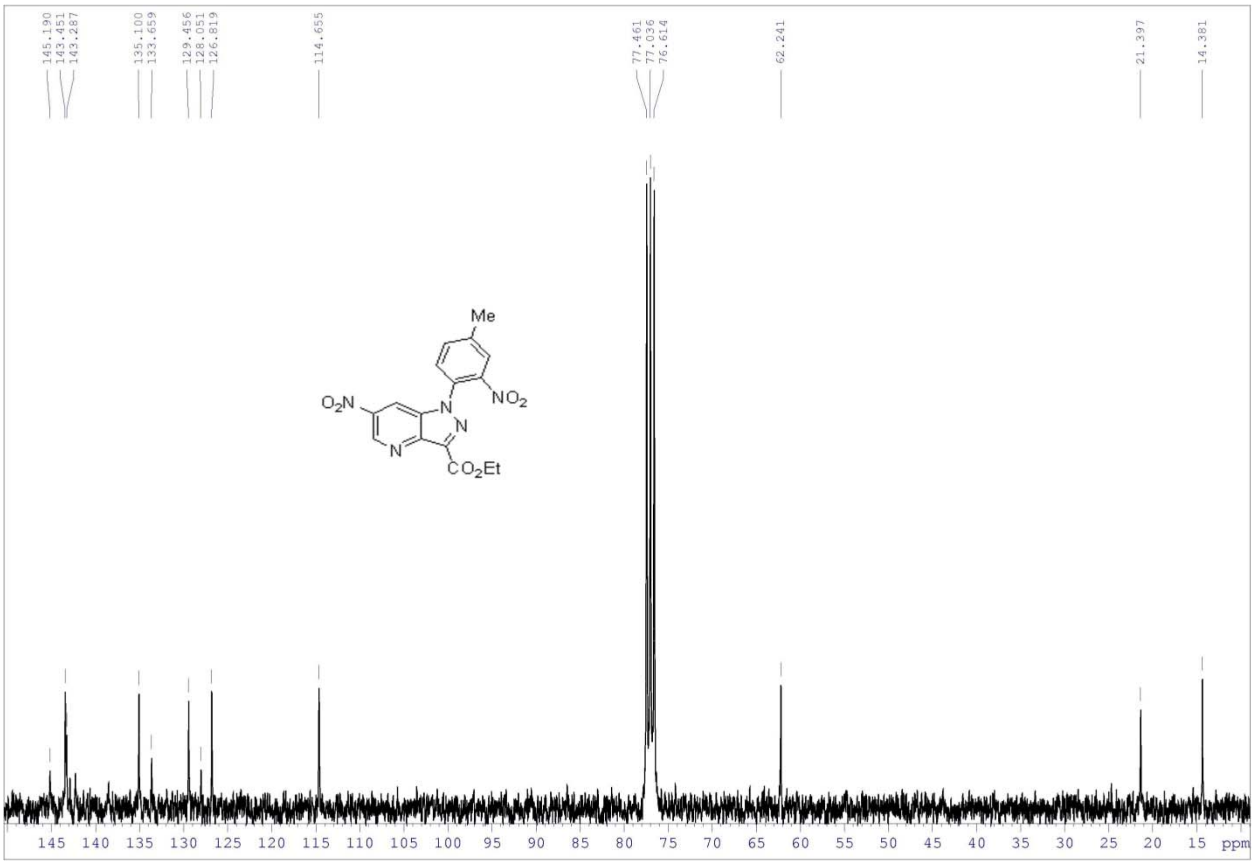

HRMS spectrum of compound **5b**

Display Report

Analysis Info

Analysis Name	D:\Data\Kolotyrkina\2022\Bastrakov\0412027.d	Acquisition Date	12.04.2022 15:12:31
Method	tune_50-1600_pos_15_12.m	Operator	BDAL@DE
Sample Name	/LPIK VN-555	Instrument / Ser#	micrOTOF 10248
Comment	C16H13N5O6 mH 372.0938 clb added CH3OH		

Acquisition Parameter

Source Type	ESI	Ion Polarity	Positive	Set Nebulizer	0.4 Bar
Focus	Not active			Set Dry Heater	180 °C
Scan Begin	50 m/z	Set Capillary	4500 V	Set Dry Gas	4.0 l/min
Scan End	1600 m/z	Set End Plate Offset	-500 V	Set Divert Valve	Waste

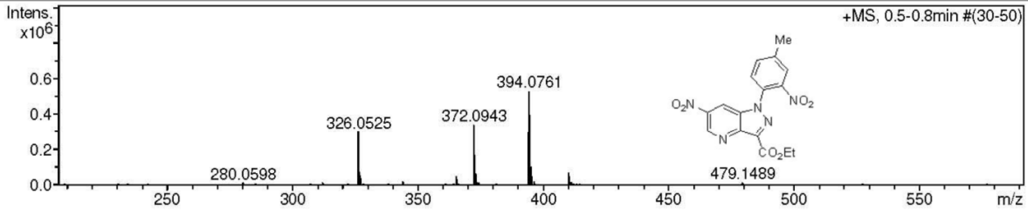

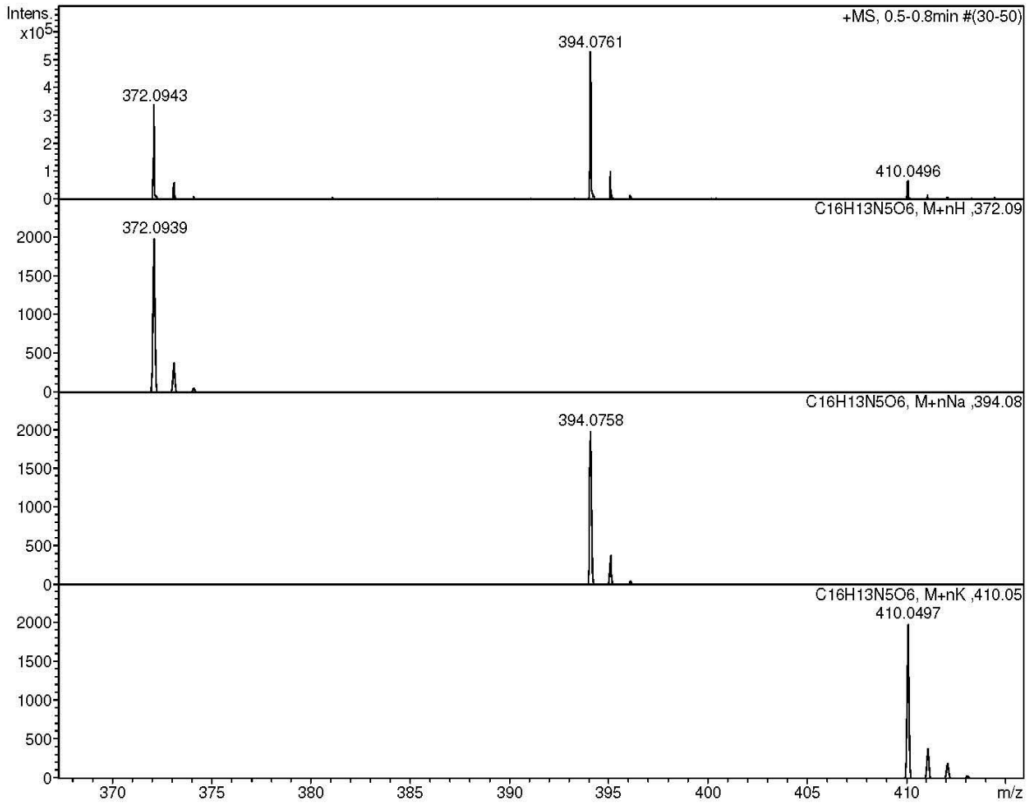

^{1}H and ^{13}C NMR spectra of compound **5c** in $CDCl_3$

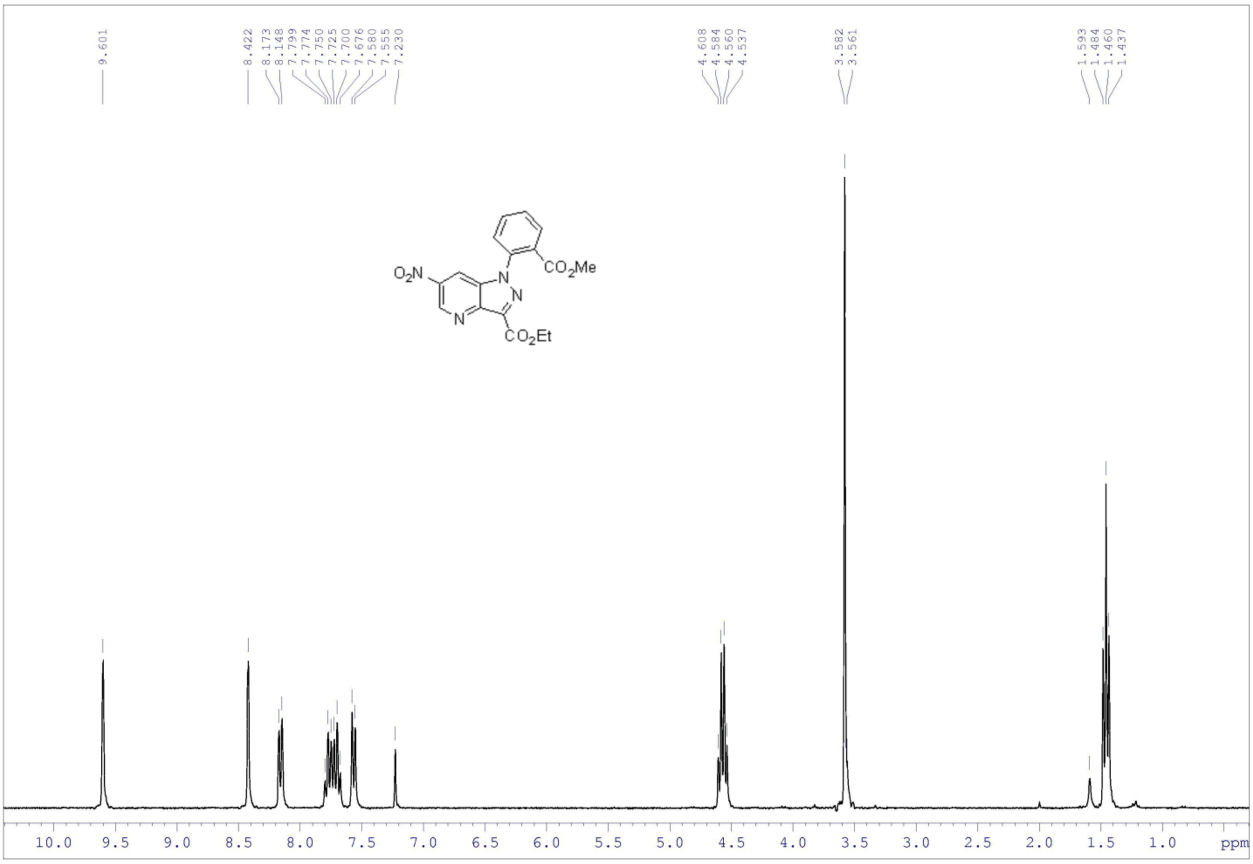

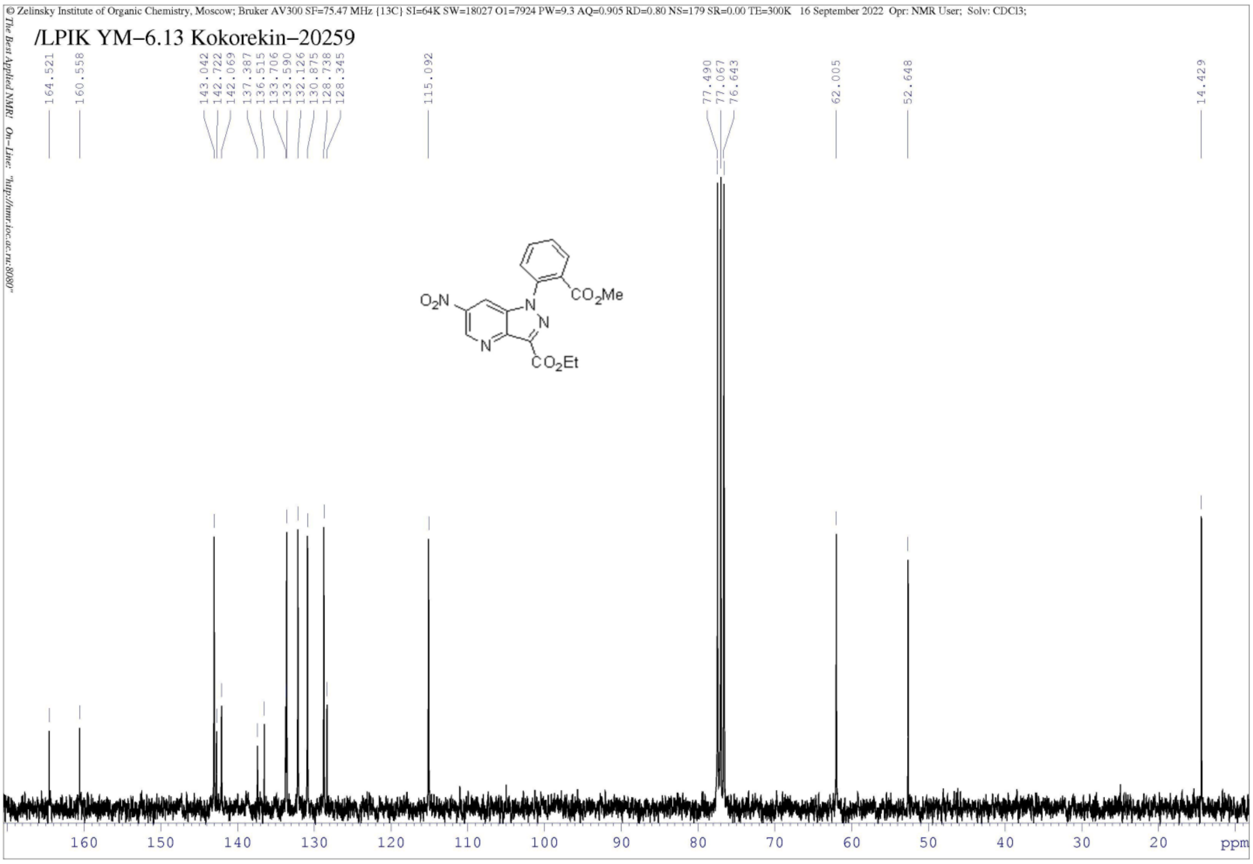

HRMS spectrum of compound 5c

Display Report

Analysis Info

Analysis Name	D:\Data\Kolotyrkina\2022\Bastrakov\1129002.d	Acquisition Date	29.11.2022 10:16:26
Method	tune_wide.m	Operator	BDAL@DE
Sample Name	/LPIK YM-6	Instrument / Ser#	micrOTOF 10248
Comment	C17H14N4O6 mH 371.0986 calibrant added CH3CN		

Acquisition Parameter

Source Type	ESI	Ion Polarity	Positive	Set Nebulizer	0.4 Bar
Focus	Not active			Set Dry Heater	180 °C
Scan Begin	50 m/z	Set Capillary	4500 V	Set Dry Gas	4.0 l/min
Scan End	3000 m/z	Set End Plate Offset	-500 V	Set Divert Valve	Waste

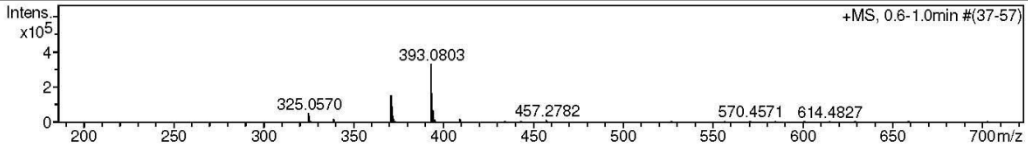

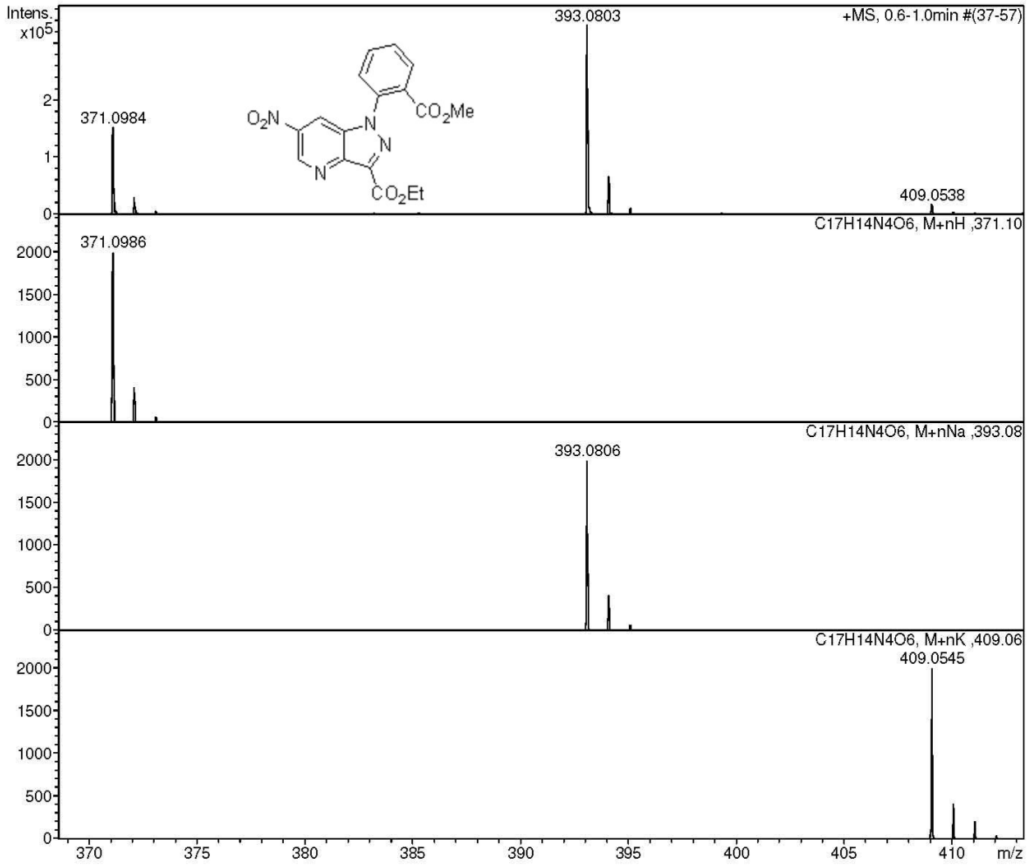

^{1}H and ^{13}C NMR spectra of compound **5d** in $CDCl_3$

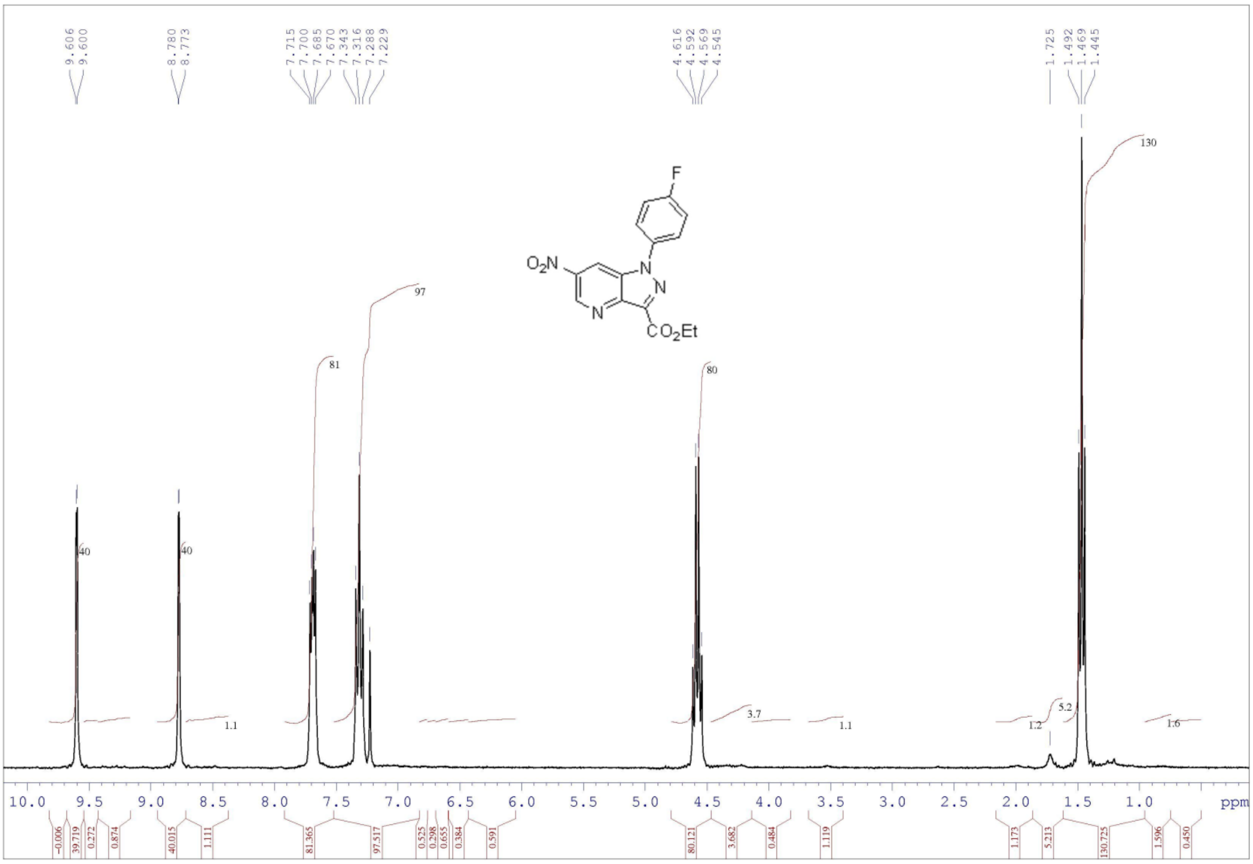

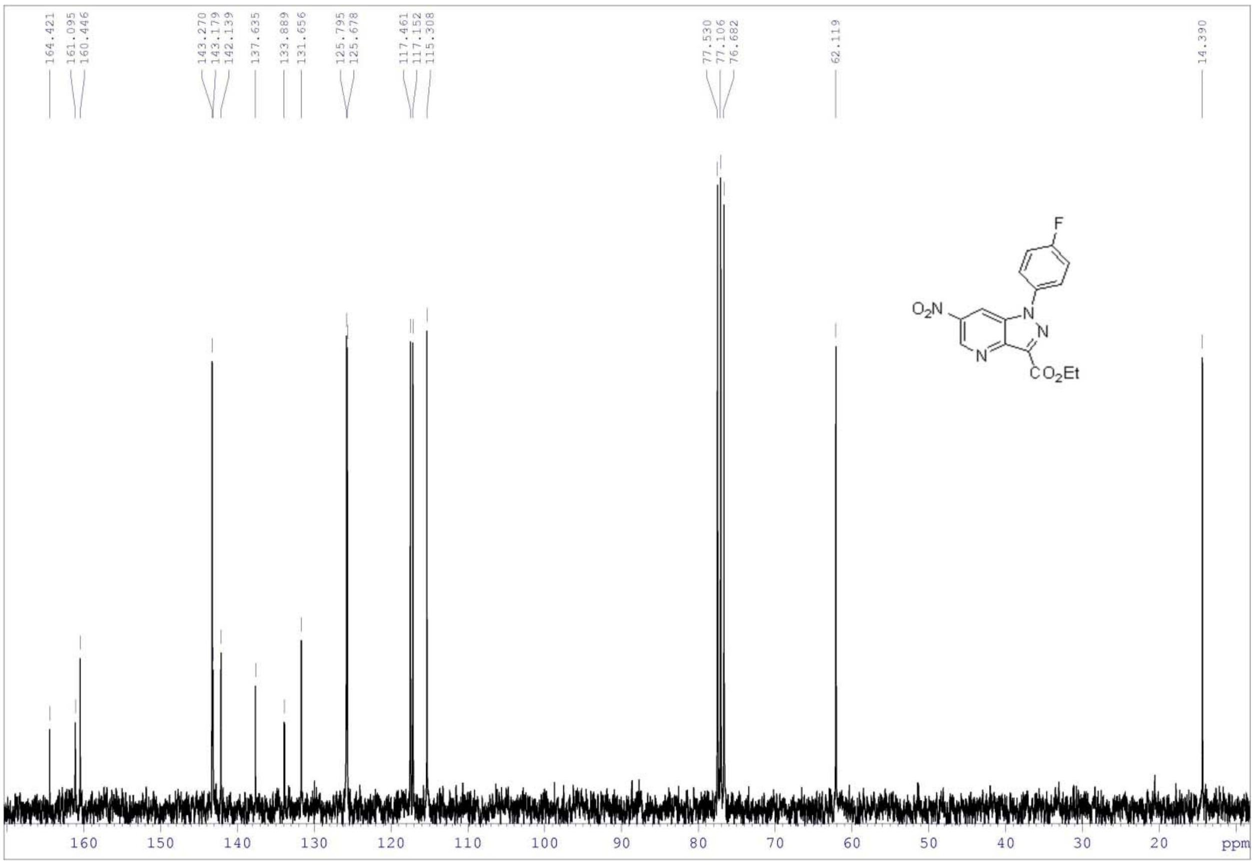

HRMS spectrum of compound 5d

Display Report

Analysis Info

Analysis Name	D:\Data\Kolotyrkina\2022\Bastrakov\1129003.d	Acquisition Date	29.11.2022 10:21:44
Method	tune_wide.m	Operator	BDAL@DE
Sample Name	/LPIK YM-8	Instrument / Ser#	micrOTOF 10248
Comment	C15H11FN4O4 mH 331.0837 calibrant added CH3CN		

Acquisition Parameter

Source Type	ESI	Ion Polarity	Positive	Set Nebulizer	0.4 Bar
Focus	Not active			Set Dry Heater	180 °C
Scan Begin	50 m/z	Set Capillary	4500 V	Set Dry Gas	4.0 l/min
Scan End	3000 m/z	Set End Plate Offset	-500 V	Set Divert Valve	Waste

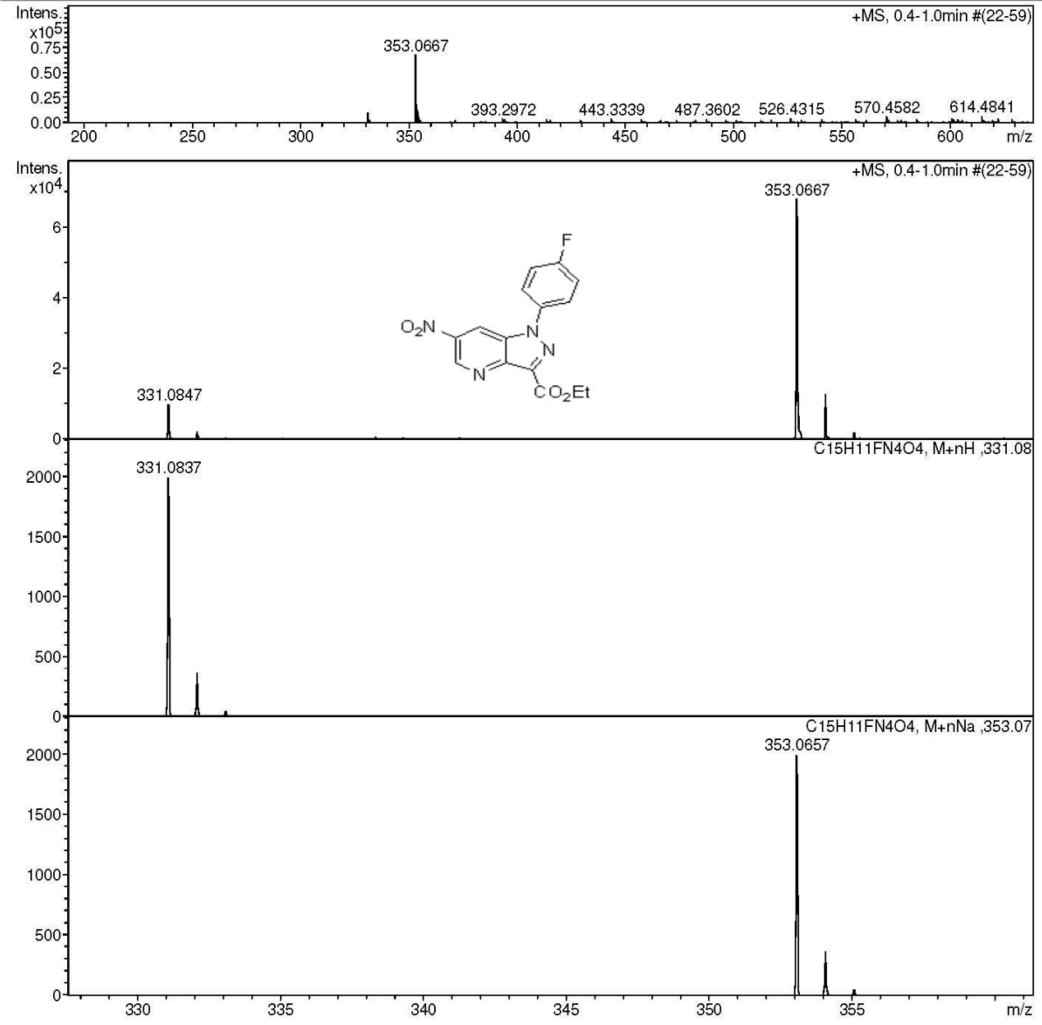

^{1}H and ^{13}C NMR spectra of compound **5e** in CDCl$_3$

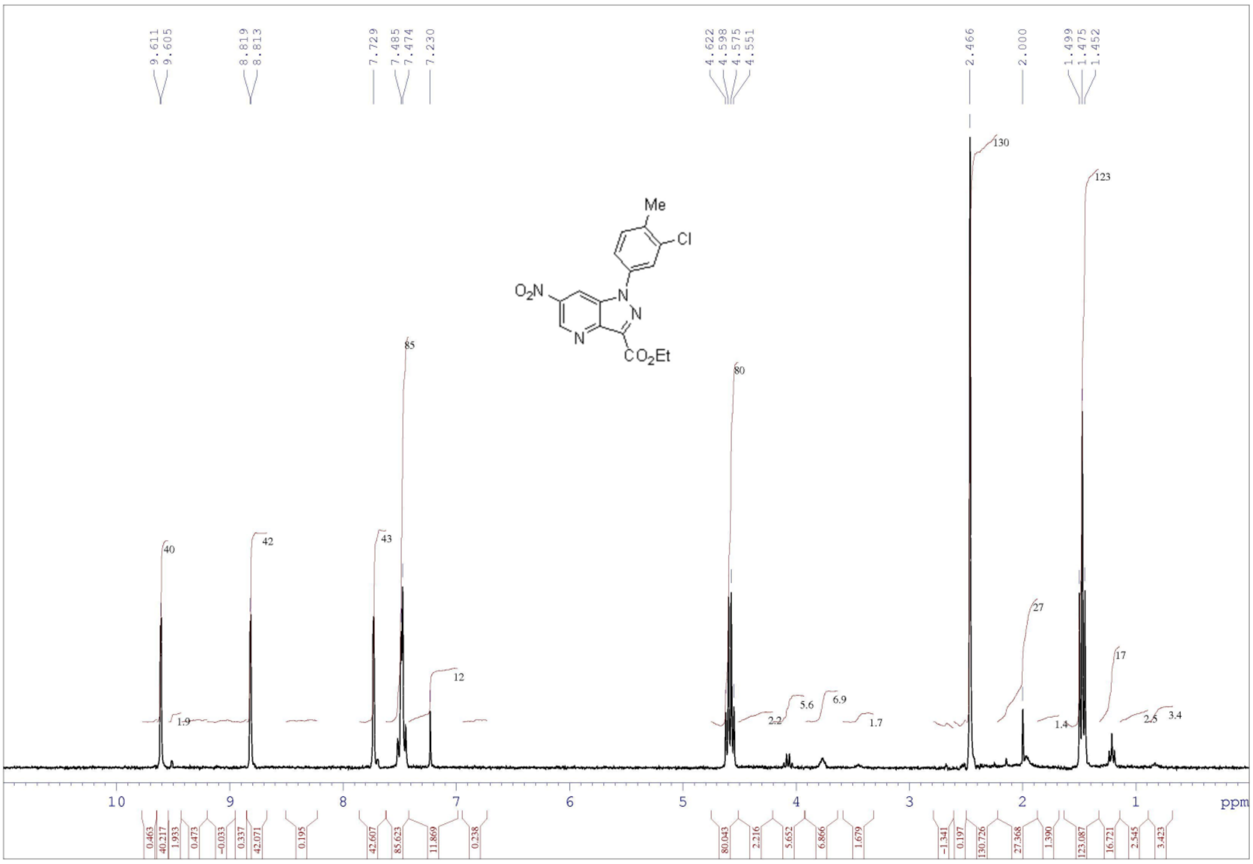

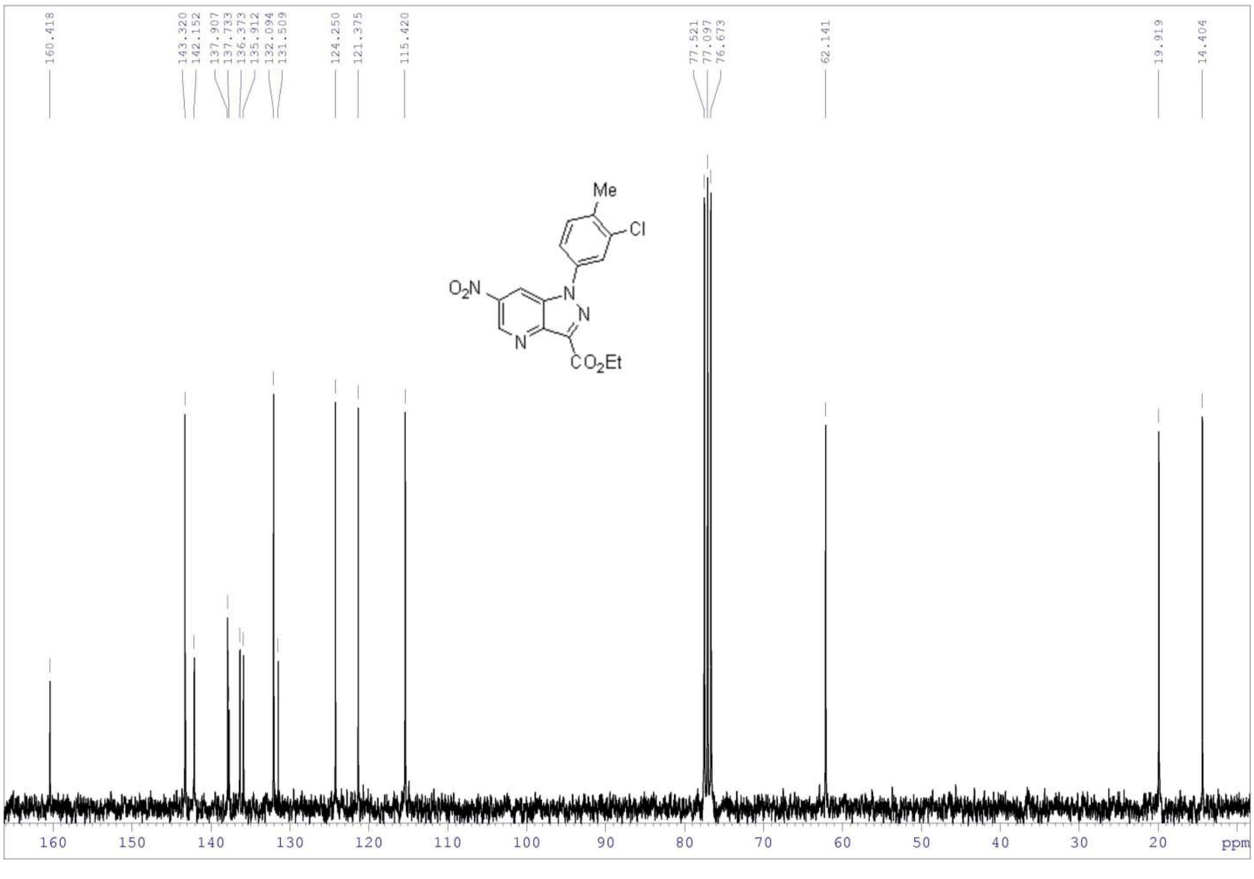

HRMS spectrum of compound 5e

Display Report

Analysis Info

Analysis Name	D:\Data\Kolotyrkina\2022\Bastrakov\0705020.d	Acquisition Date	05.07.2022 15:06:12
Method	tune_50-1600_pos_15_12.m	Operator	BDAL@DE
Sample Name	/LPIK YM-9	Instrument / Ser#	micrOTOF 10248
Comment	C16H13ClN4O4 mH 361.0698 calibrant added CH3CN		

Acquisition Parameter

Source Type	ESI	Ion Polarity	Positive	Set Nebulizer	0.4 Bar
Focus	Not active			Set Dry Heater	180 °C
Scan Begin	50 m/z	Set Capillary	4500 V	Set Dry Gas	4.0 l/min
Scan End	1600 m/z	Set End Plate Offset	-500 V	Set Divert Valve	Waste

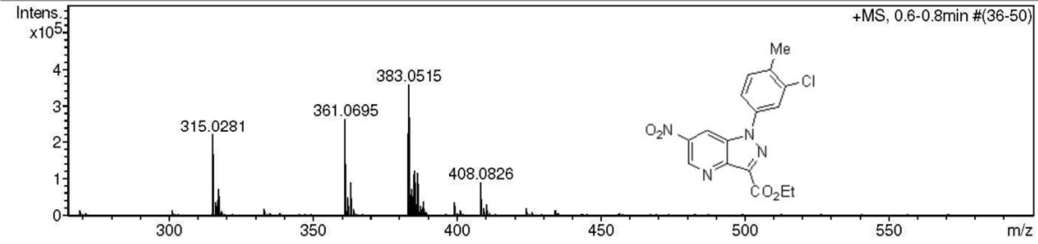

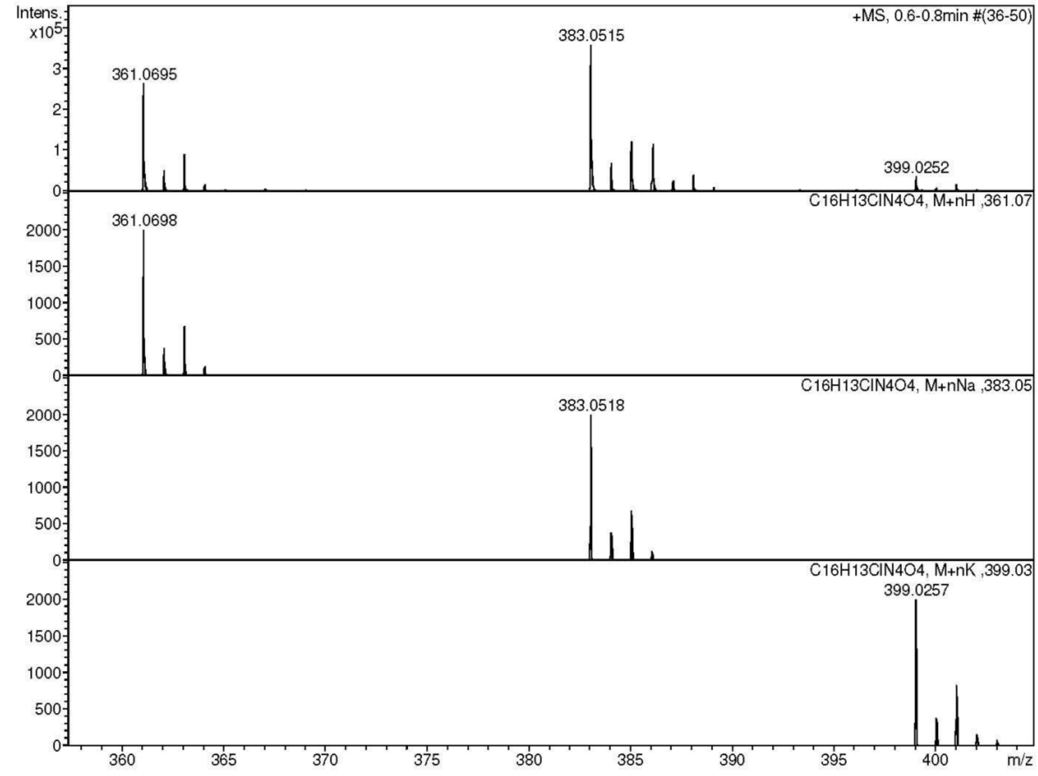

^{1}H and ^{13}C NMR spectra of compound **5f** in $CDCl_3$

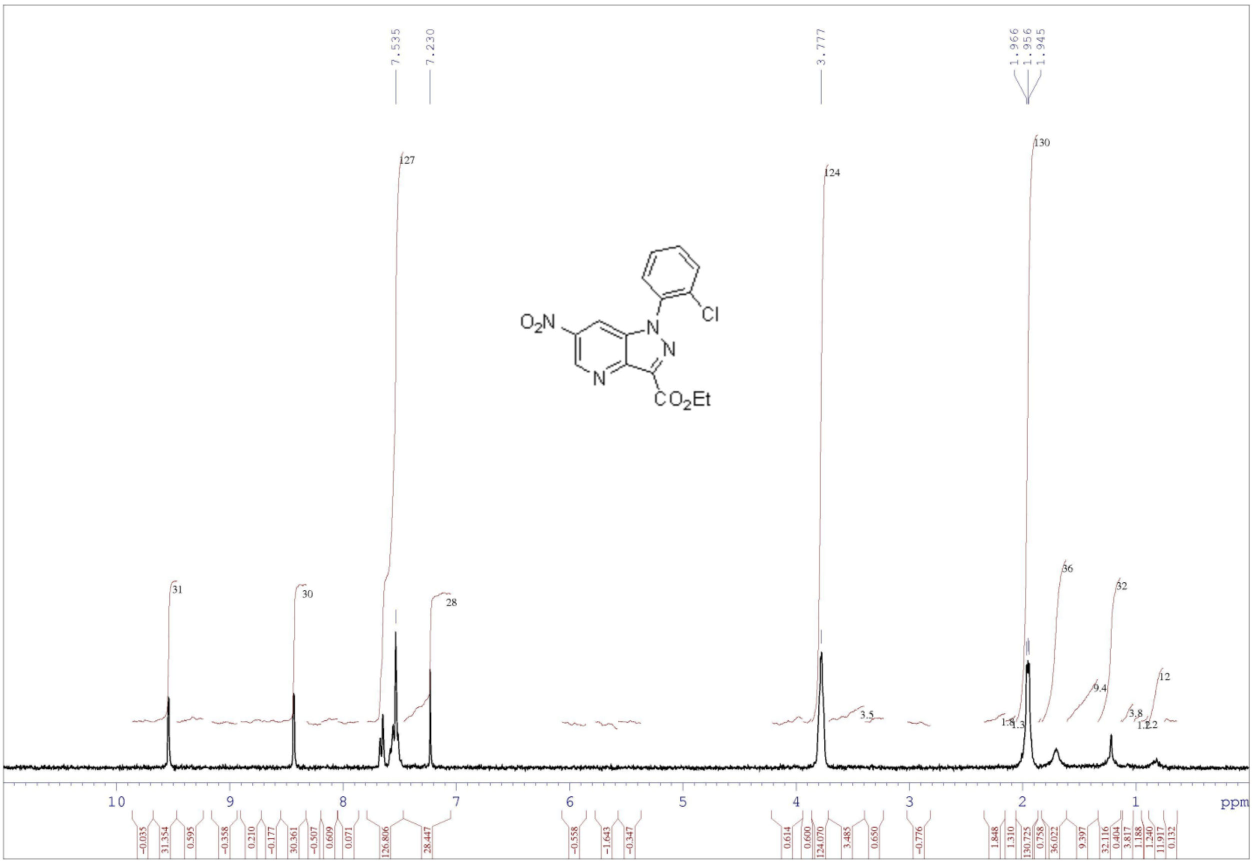

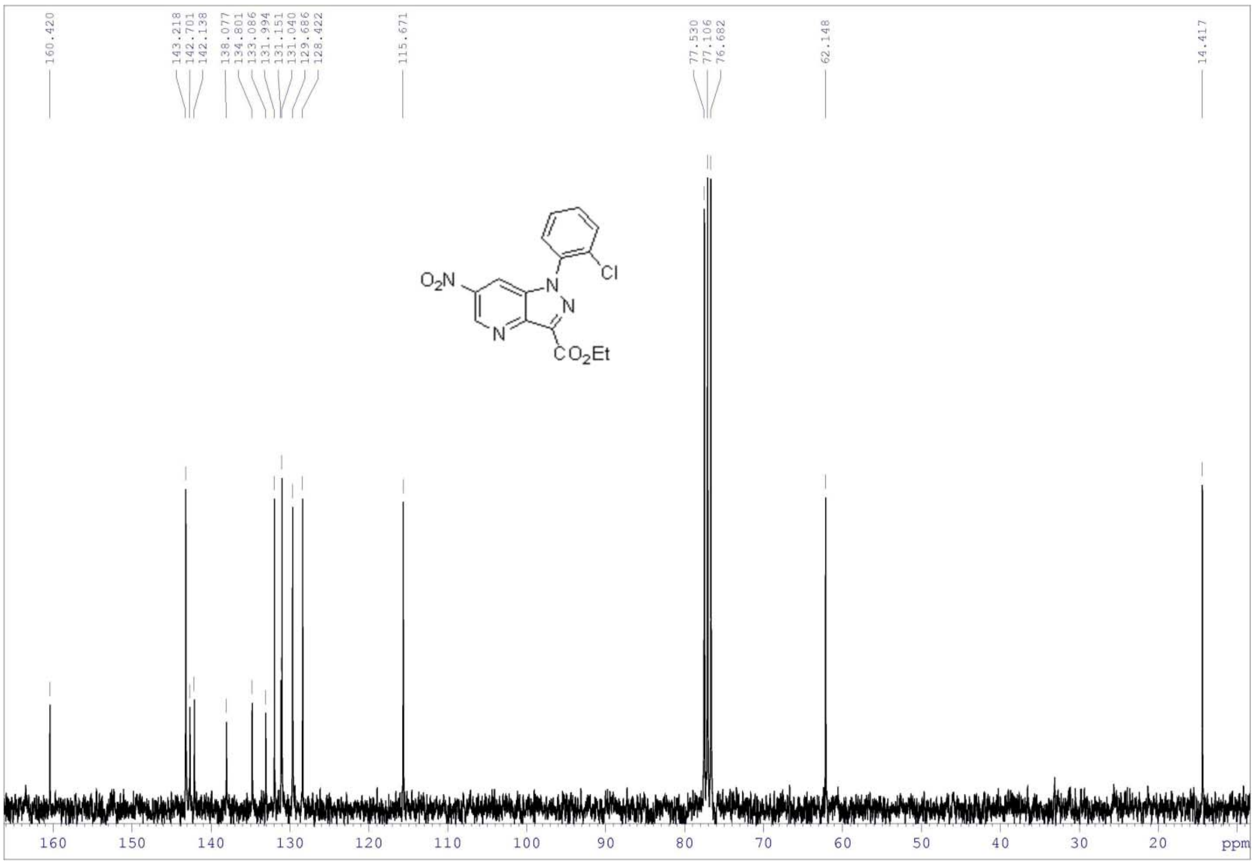

HRMS spectrum of compound 5f

Display Report

Analysis Info

Analysis Name	D:\Data\Kolotyrkina\2022\Bastrakov\0705021.d	Acquisition Date	05.07.2022 15:11:29
Method	tune_50-1600_pos_15_12.m	Operator	BDAL@DE
Sample Name	/LPIK YM-11.1	Instrument / Ser#	micrOTOF 10248
Comment	C15H11ClN4O4 mH 347.0541 calibrant added CH3CN		

Acquisition Parameter

Source Type	ESI	Ion Polarity	Positive	Set Nebulizer	0.4 Bar
Focus	Not active			Set Dry Heater	180 °C
Scan Begin	50 m/z	Set Capillary	4500 V	Set Dry Gas	4.0 l/min
Scan End	1600 m/z	Set End Plate Offset	-500 V	Set Divert Valve	Waste

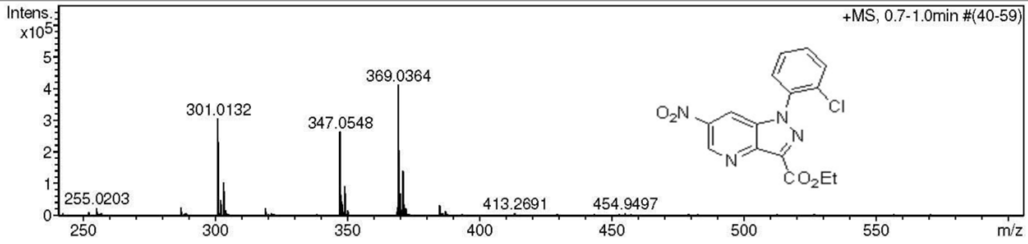

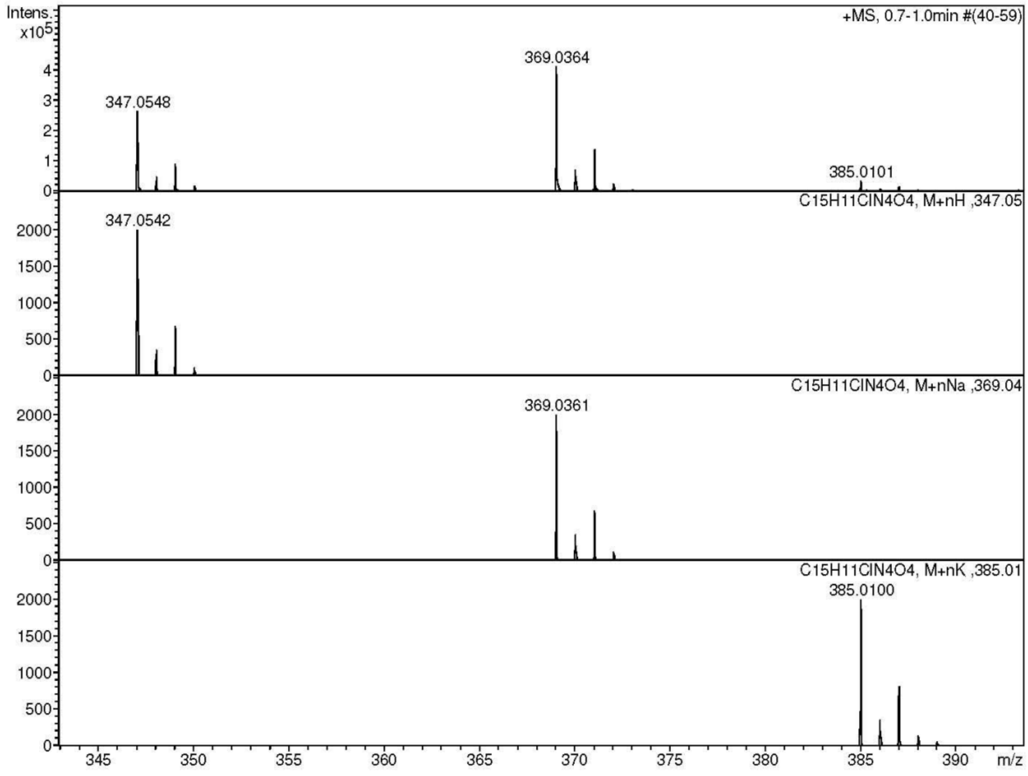

^1H and ^{13}C NMR spectra of compound **5g** in $CDCl_3$

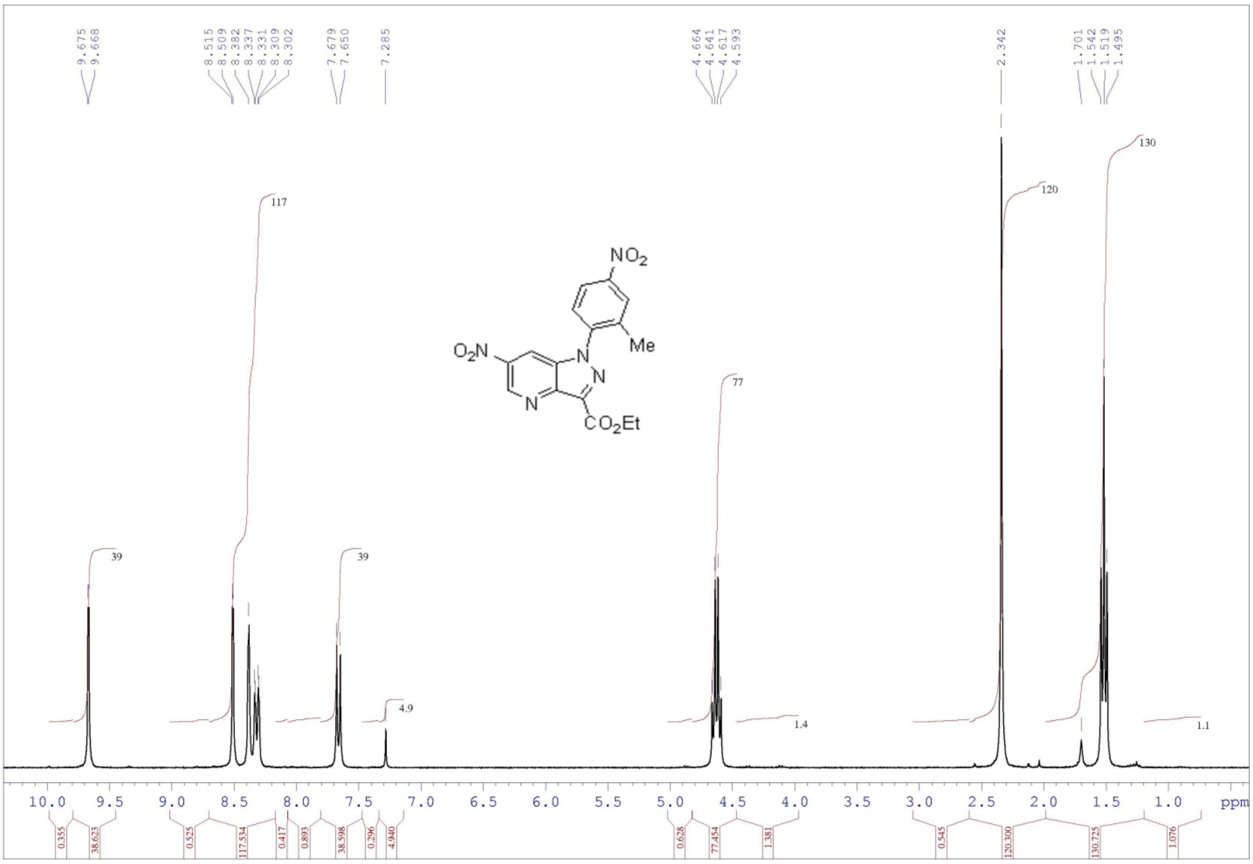

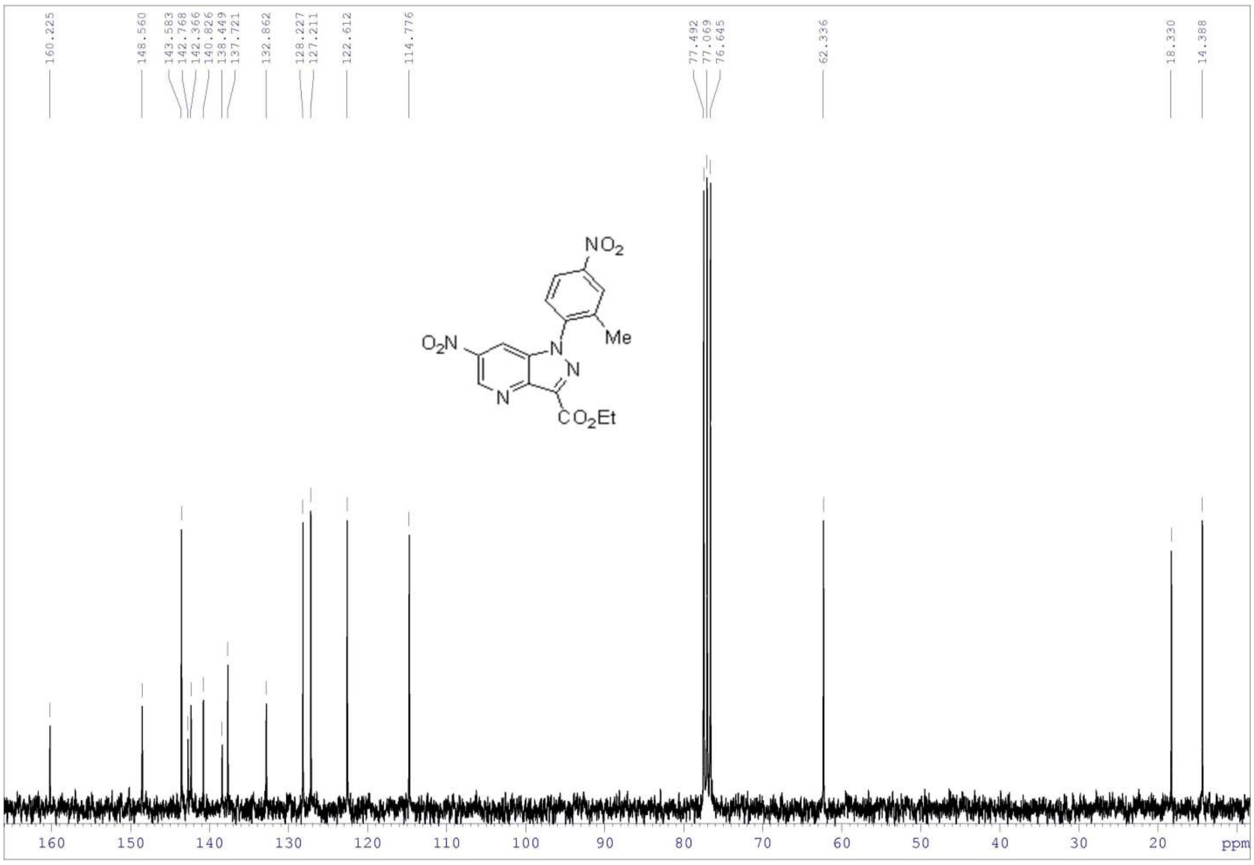

HRMS spectrum of compound 5g

Display Report

Analysis Info

Analysis Name	D:\Data\Kolotyrkina\2022\Bastrakov\1221007.d	Acquisition Date	21.12.2022 10:09:22
Method	tune_low.m	Operator	BDAL@DE
Sample Name	/LPIK VN-606	Instrument / Ser#	micrOTOF 10248
Comment	C16H13N5O6 mH 372.0938 calibrant added CH3CN		

Acquisition Parameter

Source Type	ESI	Ion Polarity	Positive	Set Nebulizer	0.4 Bar
Focus	Not active			Set Dry Heater	180 °C
Scan Begin	50 m/z	Set Capillary	4500 V	Set Dry Gas	4.0 l/min
Scan End	2500 m/z	Set End Plate Offset	-500 V	Set Divert Valve	Waste

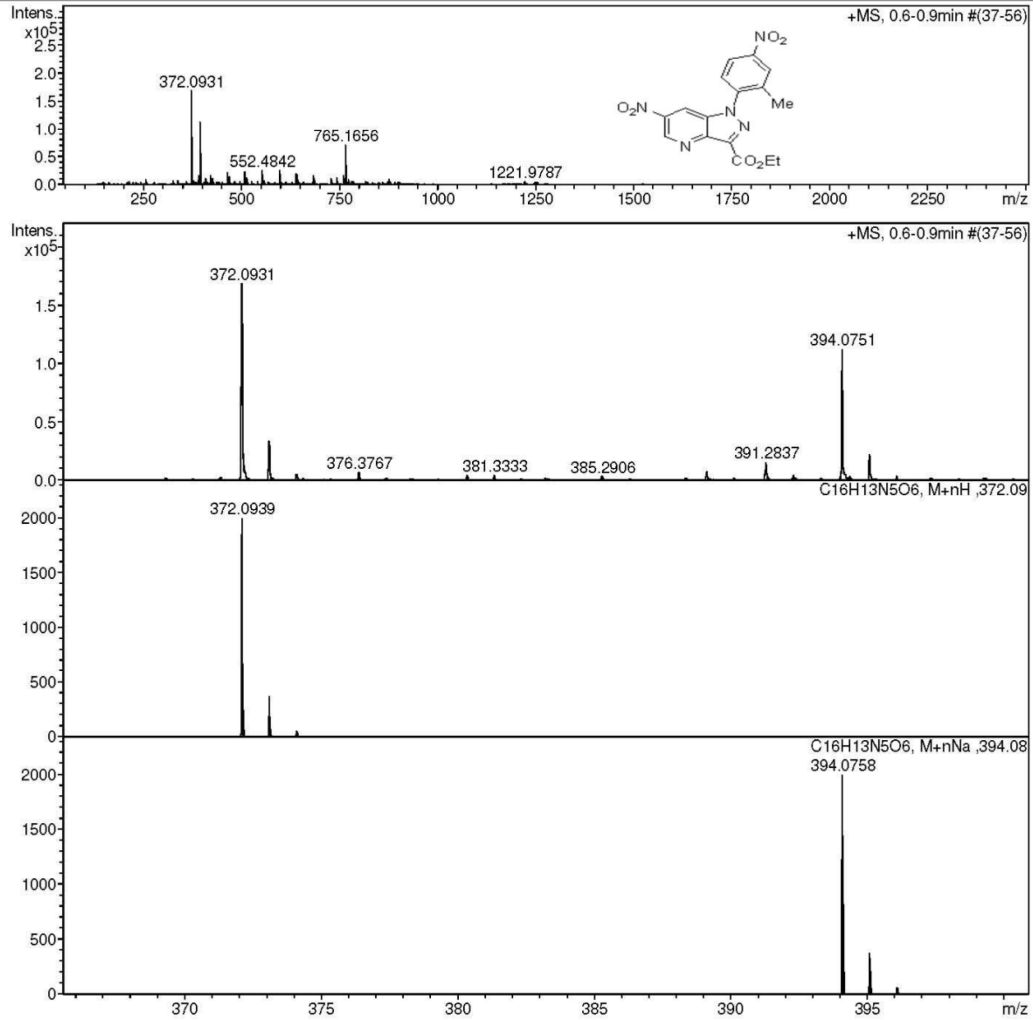

Bruker Compass DataAnalysis 4.0 printed: 21.12.2022 10:13:11 Page 1 of 1

^{1}H and ^{13}C NMR spectra of compound **5h** in $CDCl_3$

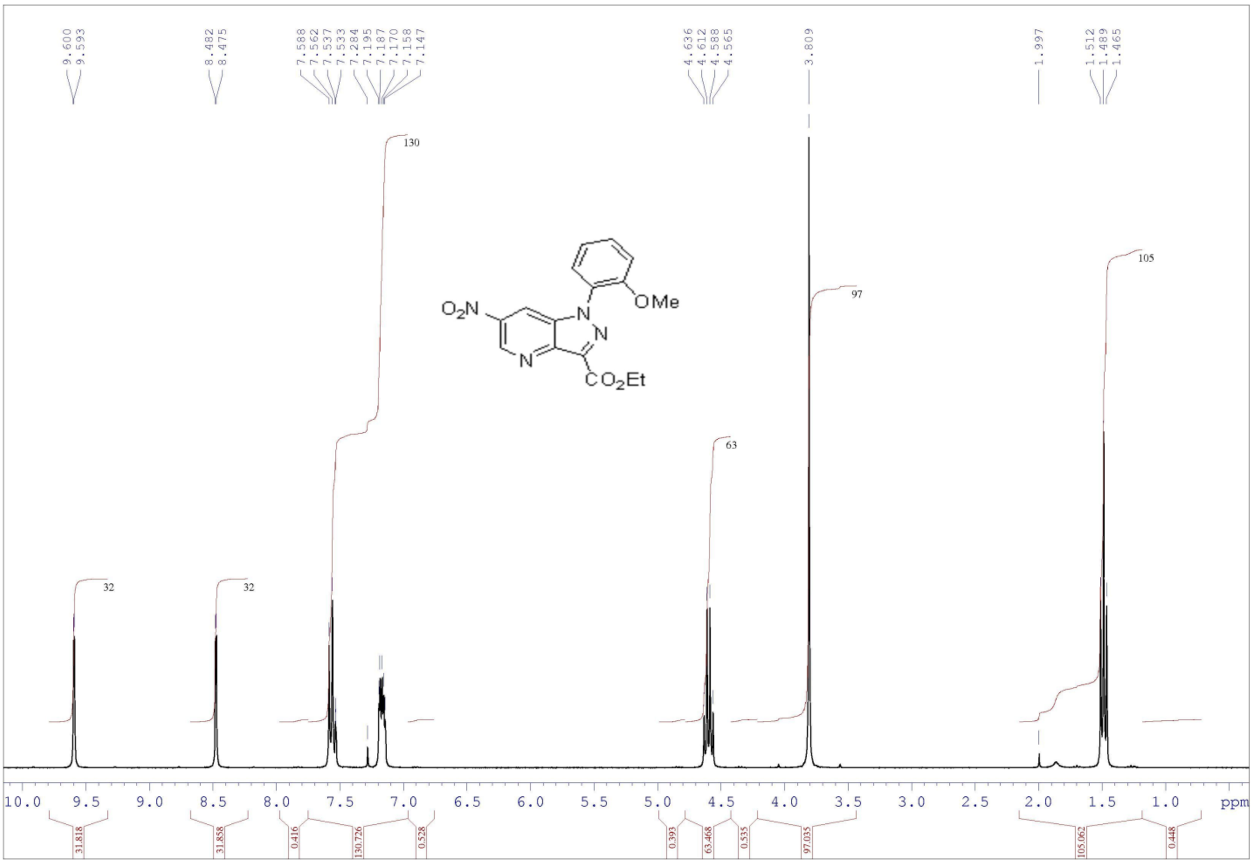

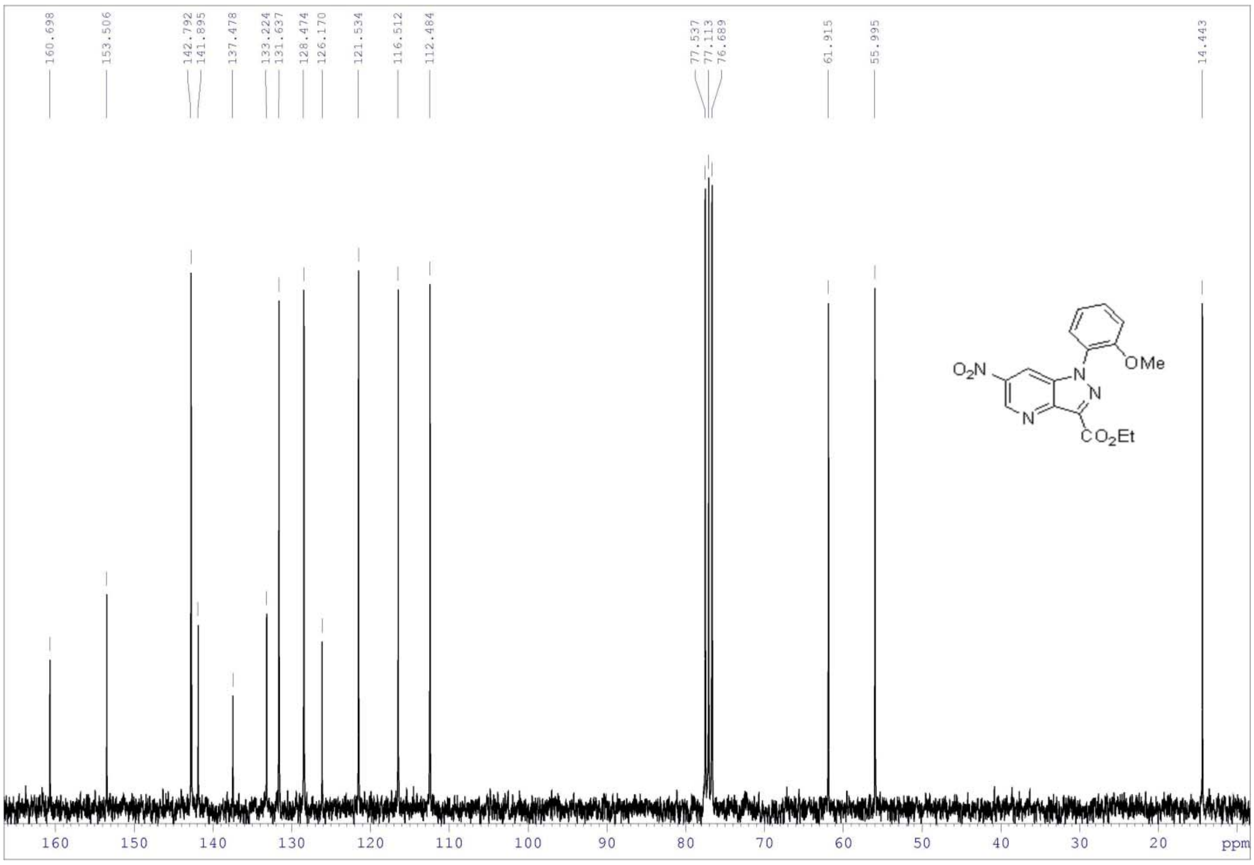

HRMS spectrum of compound 5h

Display Report

Analysis Info

Analysis Name	D:\Data\Kolotyrkina\2022\Bastrakov\1221008.d	Acquisition Date	21.12.2022 10:15:07
Method	tune_low.m	Operator	BDAL@DE
Sample Name	/LPIK VN-611	Instrument / Ser#	micrOTOF 10248
Comment	C16H14N4O5 mH 343.1036 calibrant added CH3CN		

Acquisition Parameter

Source Type	ESI	Ion Polarity	Positive	Set Nebulizer	0.4 Bar
Focus	Not active			Set Dry Heater	180 °C
Scan Begin	50 m/z	Set Capillary	4500 V	Set Dry Gas	4.0 l/min
Scan End	2500 m/z	Set End Plate Offset	-500 V	Set Divert Valve	Waste

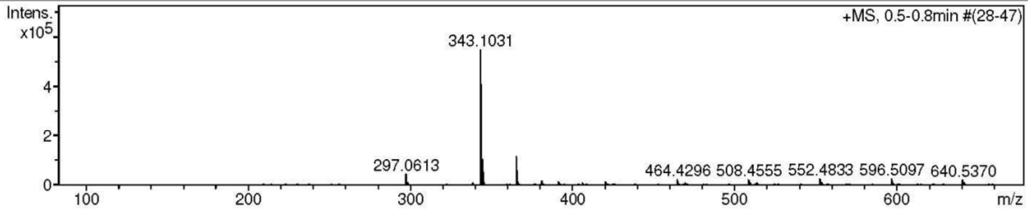

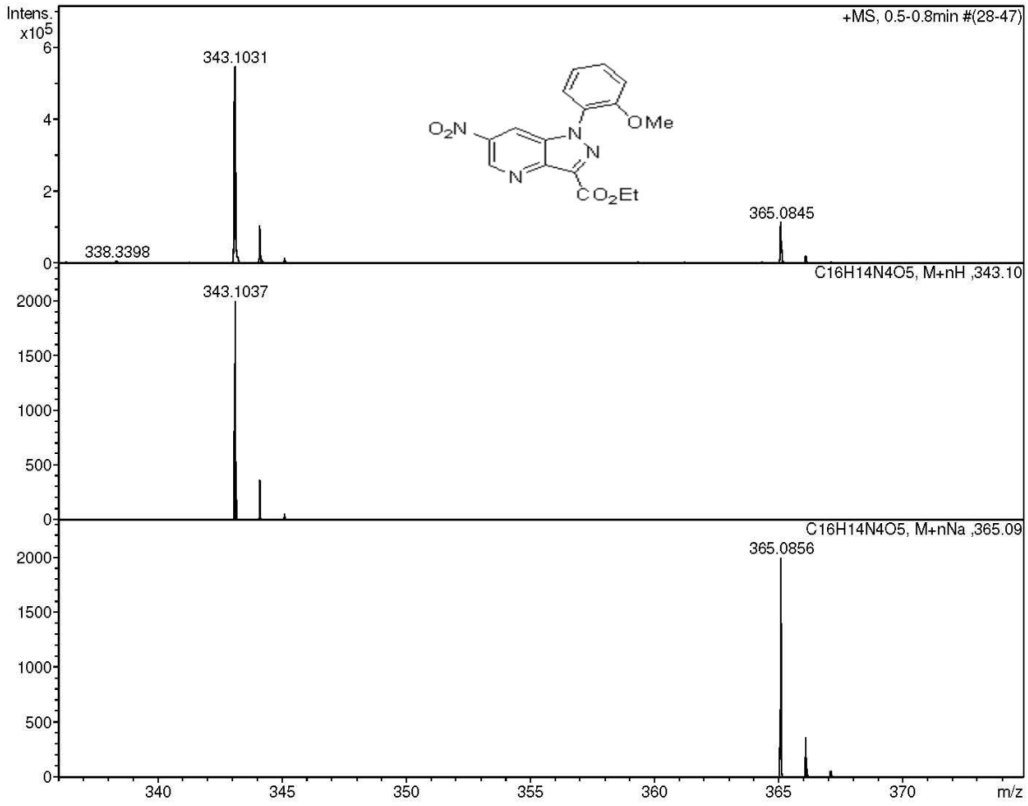

^{1}H and ^{13}C NMR spectra of compound **5i** in $CDCl_3$

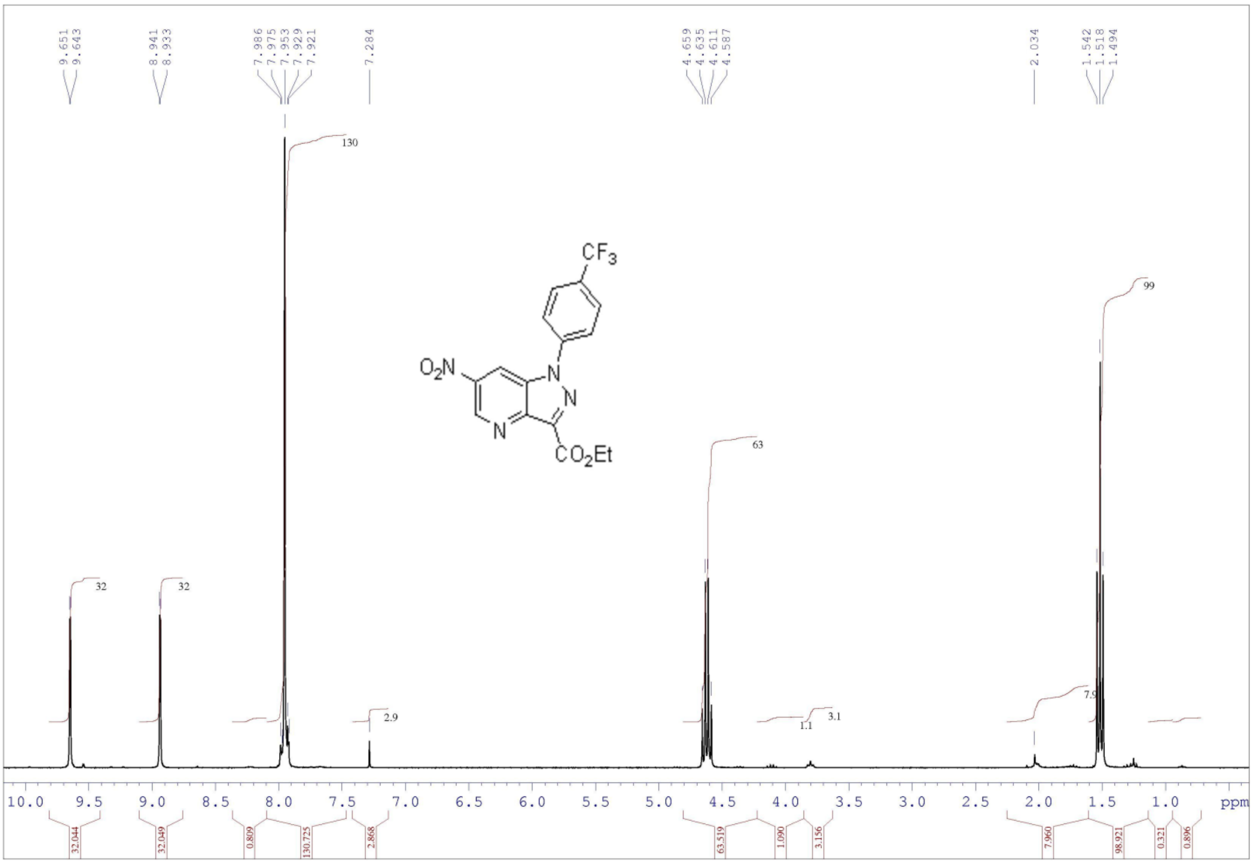

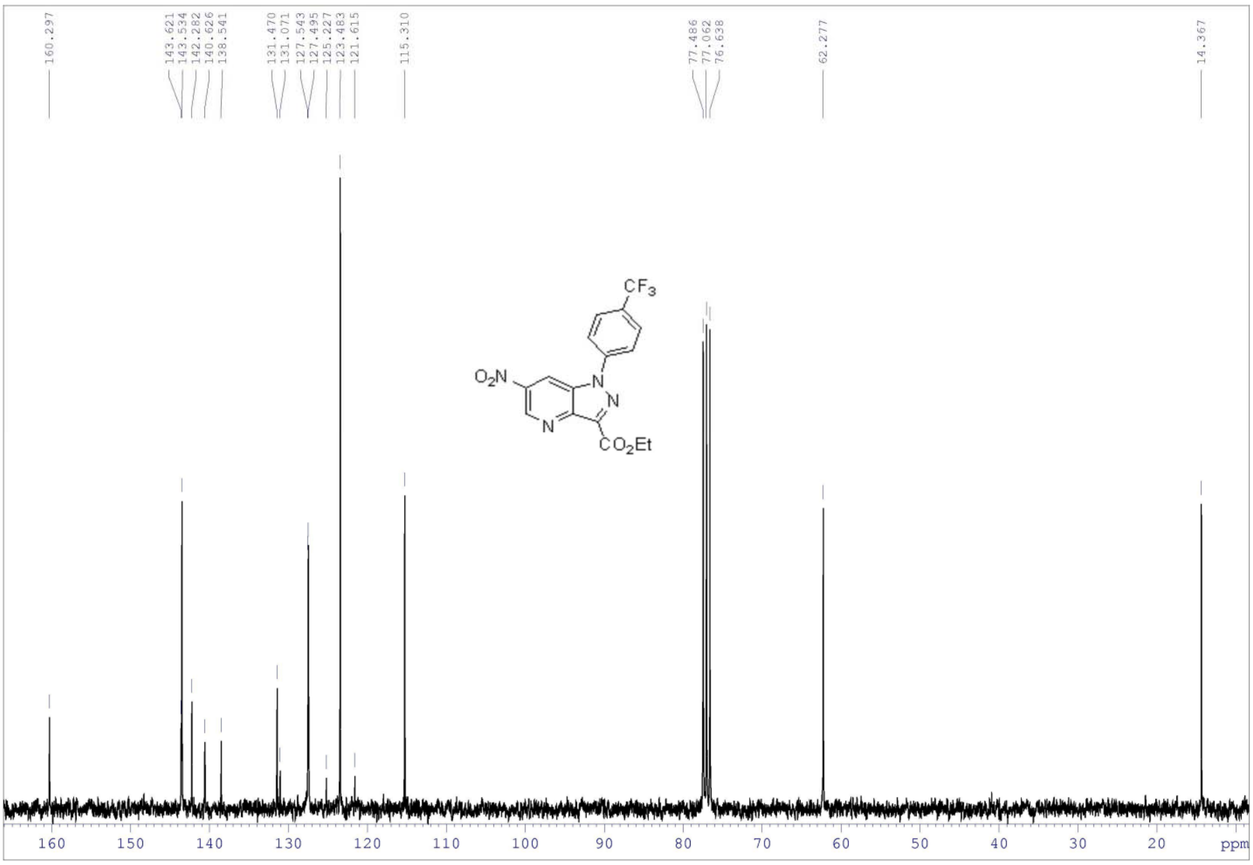

HRMS spectrum of compound 5i

Display Report

Analysis Info

Analysis Name	D:\Data\Kolotyrkina\2022\Bastrakov\1221009.d	Acquisition Date	21.12.2022 10:19:11
Method	tune_low.m	Operator	BDAL@DE
Sample Name	/LPIK VN-624	Instrument / Ser#	micrOTOF 10248
Comment	C16H11F3N4O4 mH 381.0805 calibrant added CH3CN		

Acquisition Parameter

Source Type	ESI	Ion Polarity	Positive	Set Nebulizer	0.4 Bar
Focus	Not active			Set Dry Heater	180 °C
Scan Begin	50 m/z	Set Capillary	4500 V	Set Dry Gas	4.0 l/min
Scan End	2500 m/z	Set End Plate Offset	-500 V	Set Divert Valve	Waste

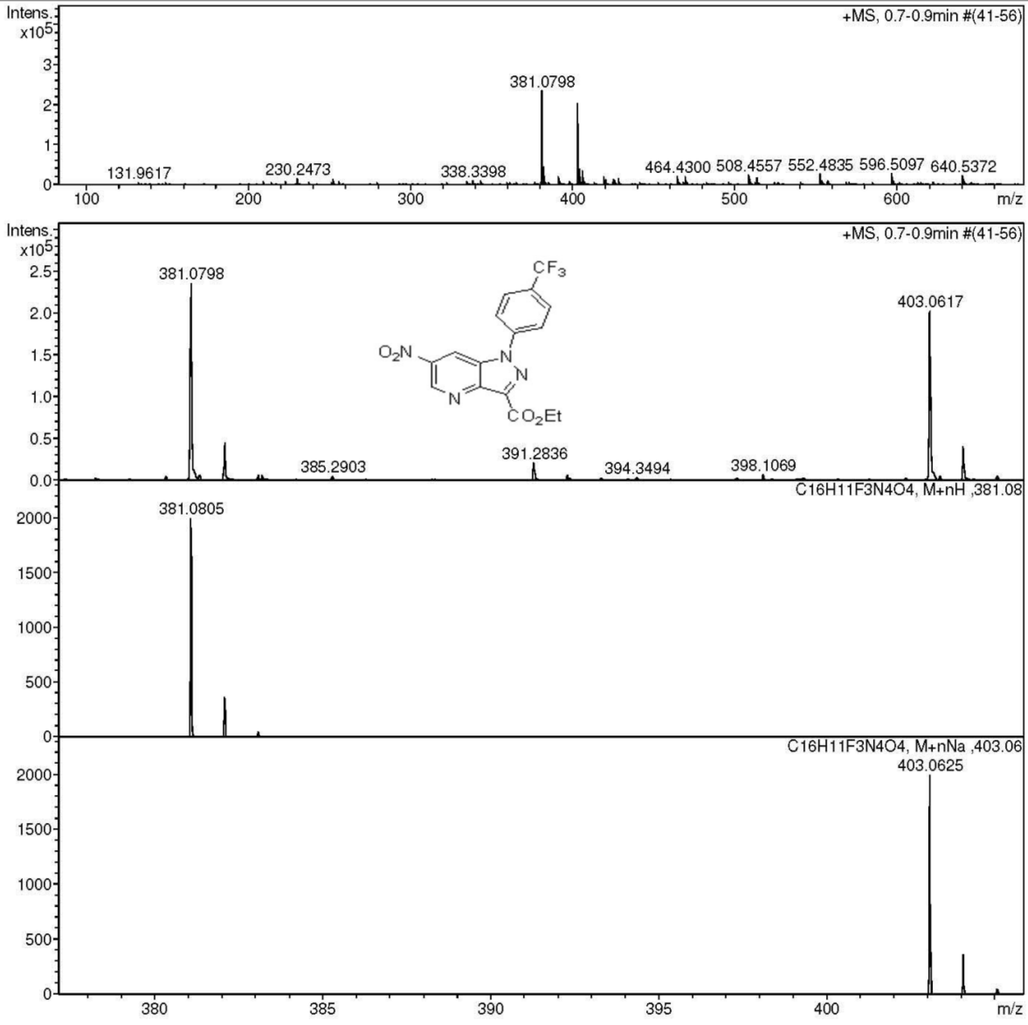

^1H and ^{13}C NMR spectra of compound **5j** in $CDCl_3$

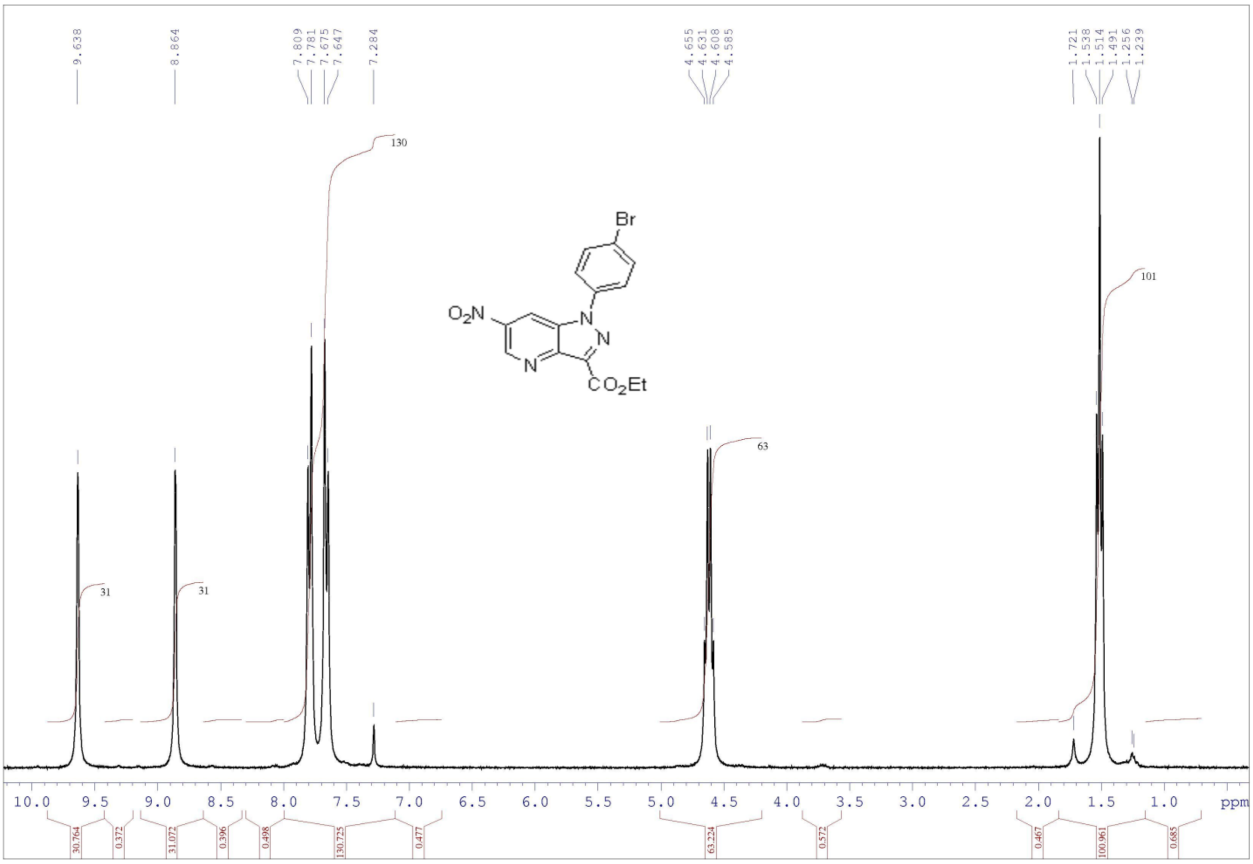

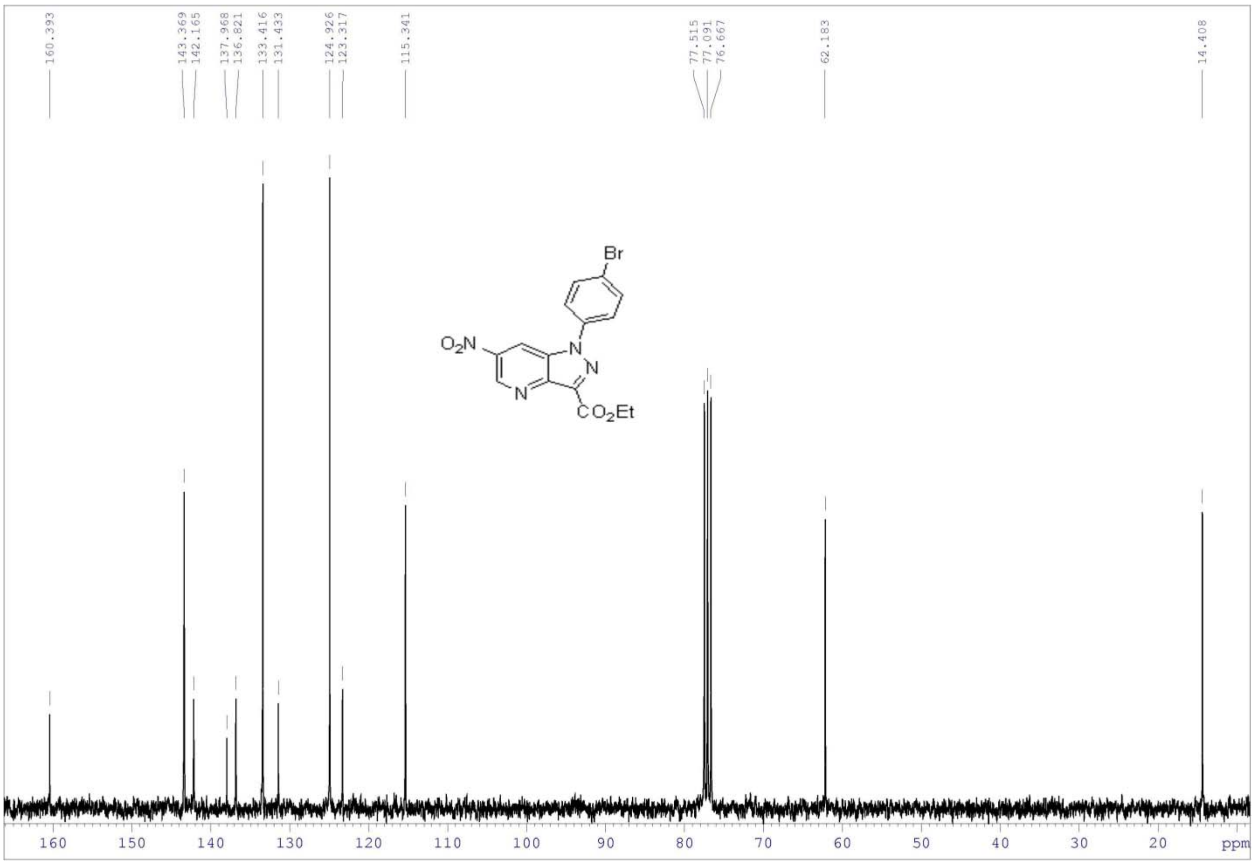

HRMS spectrum of compound 5j

Display Report

Analysis Info

Analysis Name	D:\Data\Kolotyrkina\2022\Bastrakov\1221010.d	Acquisition Date	21.12.2022 10:23:52
Method	tune_low.m	Operator	BDAL@DE
Sample Name	/LPIK VN-643	Instrument / Ser#	micrOTOF 10248
Comment	C15H11BrN4O4 mH 391.0036 calibrant added CH3CN		

Acquisition Parameter

Source Type	ESI	Ion Polarity	Positive	Set Nebulizer	0.4 Bar
Focus	Not active			Set Dry Heater	180 °C
Scan Begin	50 m/z	Set Capillary	4500 V	Set Dry Gas	4.0 l/min
Scan End	2500 m/z	Set End Plate Offset	-500 V	Set Divert Valve	Waste

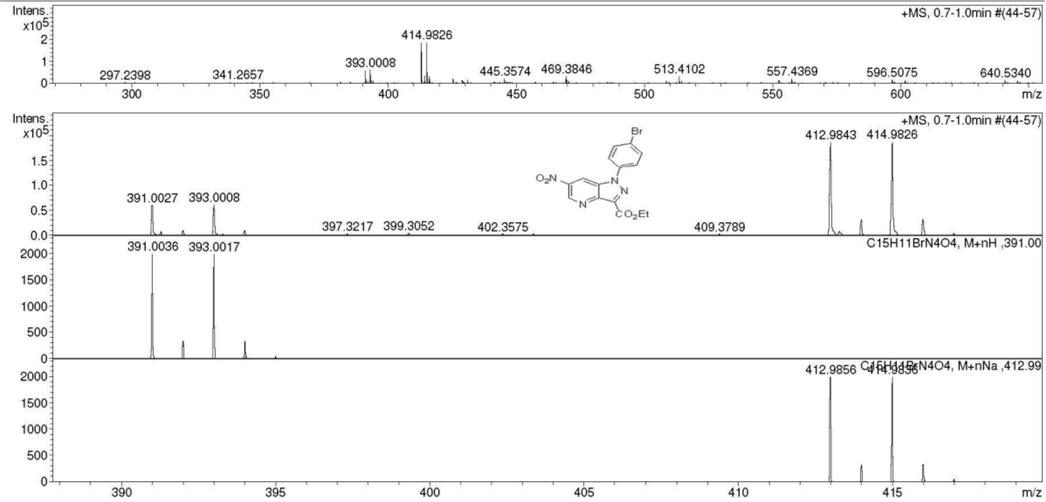

^{1}H and ^{13}C NMR spectra of compound **5k** in $CDCl_3$

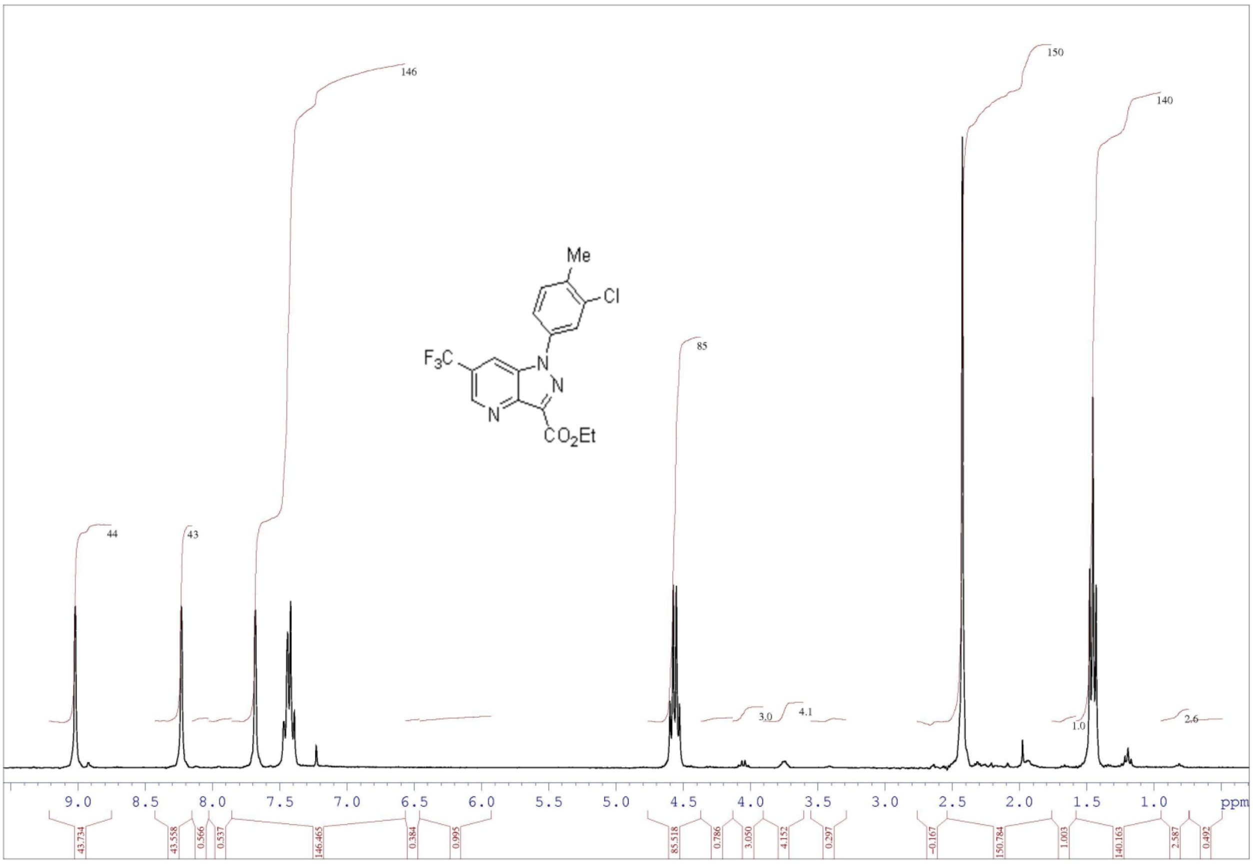

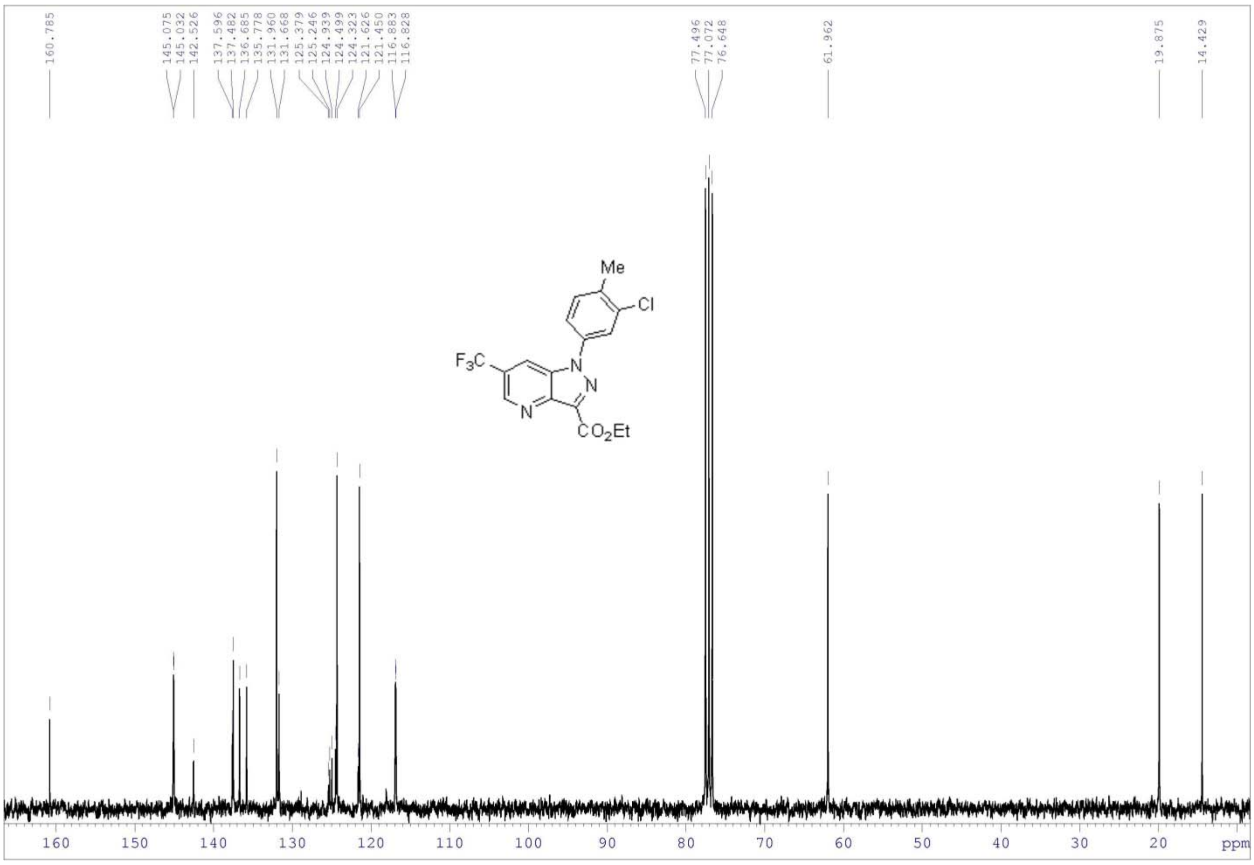

HRMS spectrum of compound 5k

Display Report

Analysis Info

Analysis Name	D:\Data\Kolotyrkina\2022\Bastrakov\1221014.d	Acquisition Date	21.12.2022 10:53:19
Method	tune_low.m	Operator	BDAL@DE
Sample Name	/LPIK VN-588	Instrument / Ser#	micrOTOF 10248
Comment	C17H13ClF3N3O2 mH 384.0721 calibrant added CH3CN		

Acquisition Parameter

Source Type	ESI	Ion Polarity	Positive	Set Nebulizer	0.4 Bar
Focus	Not active			Set Dry Heater	180 °C
Scan Begin	50 m/z	Set Capillary	4500 V	Set Dry Gas	4.0 l/min
Scan End	2500 m/z	Set End Plate Offset	-500 V	Set Divert Valve	Waste

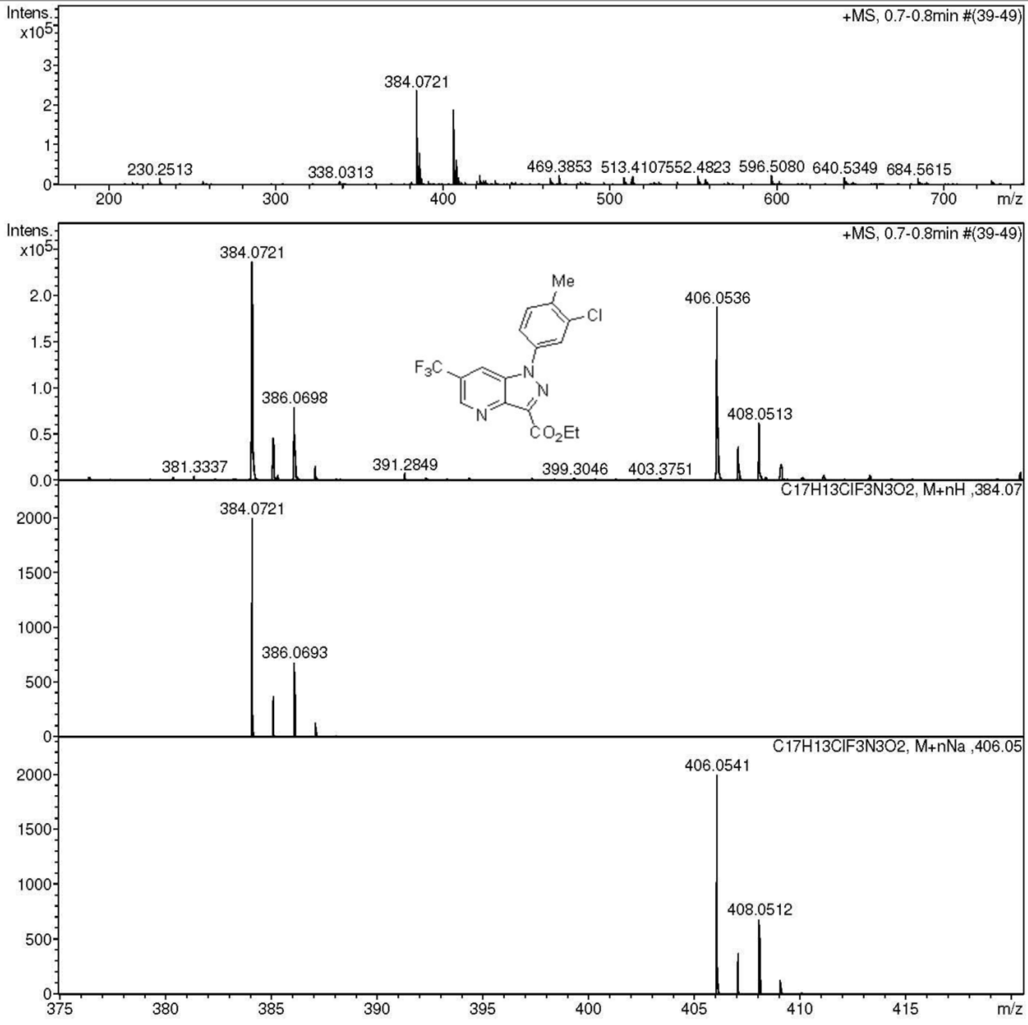

^{1}H and ^{13}C NMR spectra of compound **5l** in $CDCl_3$

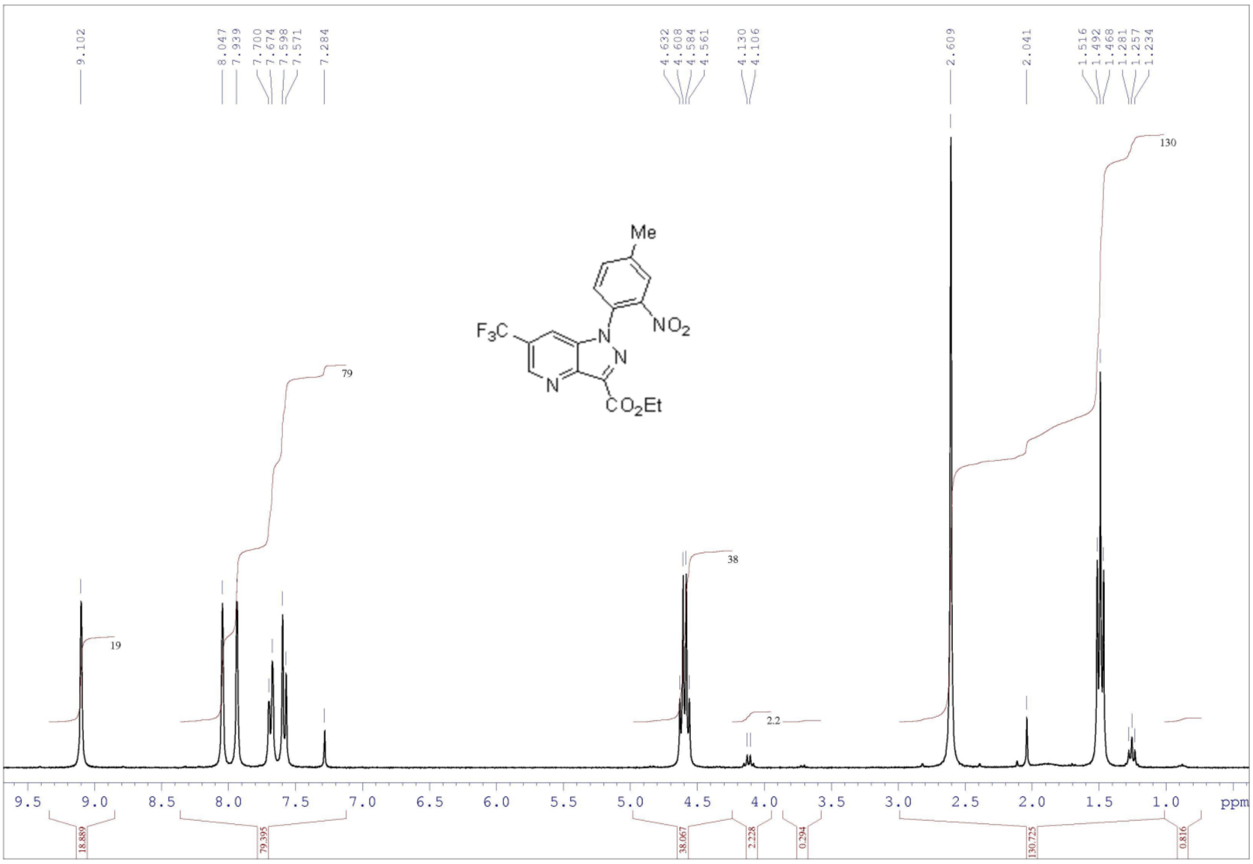

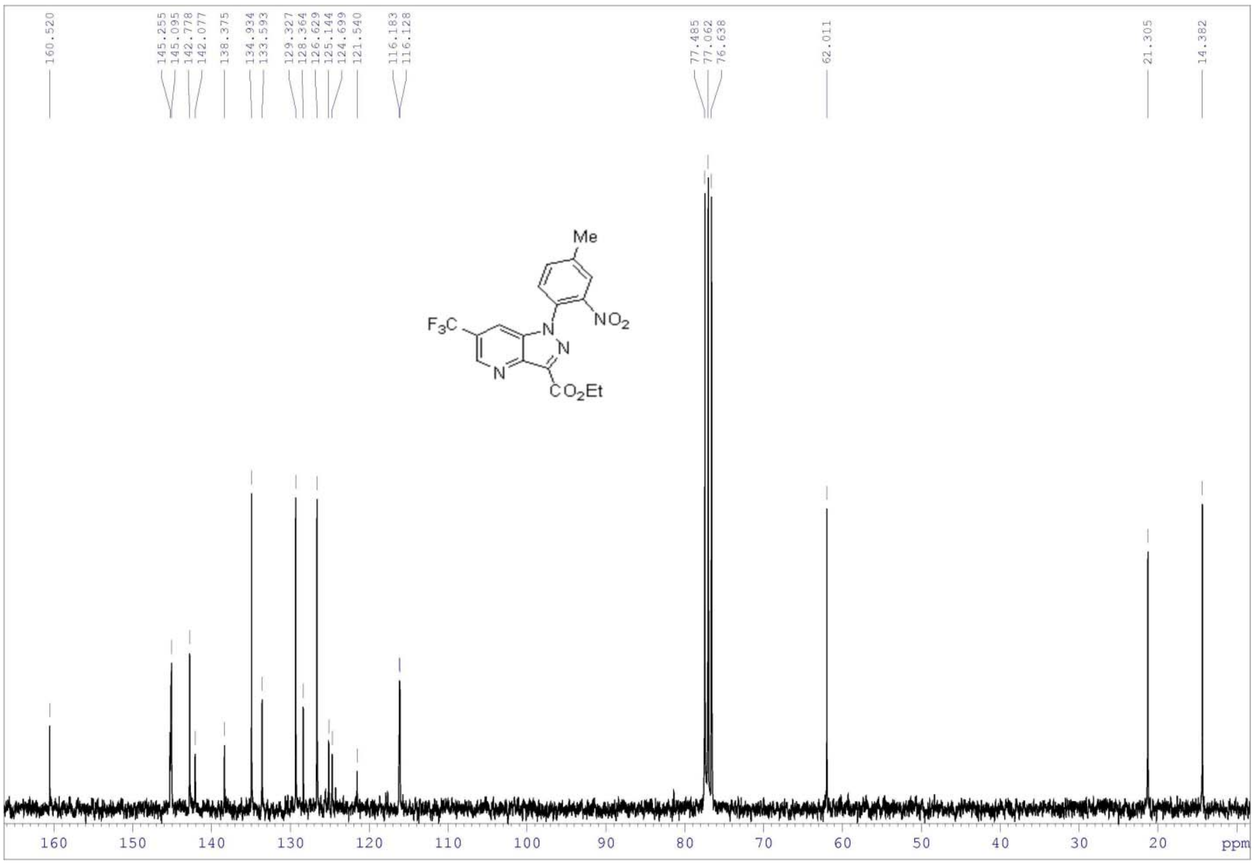

HRMS spectrum of compound 5l

Display Report

Analysis Info

Analysis Name	D:\Data\Kolotyrkina\2022\Bastrakov\1221015.d	Acquisition Date	21.12.2022 11:04:41
Method	tune_low.m	Operator	BDAL@DE
Sample Name	/LPIK VN-589	Instrument / Ser#	micrOTOF 10248
Comment	C17H13F3N4O4 mH 395.0961 calibrant added CH3CN		

Acquisition Parameter

Source Type	ESI	Ion Polarity	Positive	Set Nebulizer	0.4 Bar
Focus	Not active			Set Dry Heater	180 °C
Scan Begin	50 m/z	Set Capillary	4500 V	Set Dry Gas	4.0 l/min
Scan End	2500 m/z	Set End Plate Offset	-500 V	Set Divert Valve	Waste

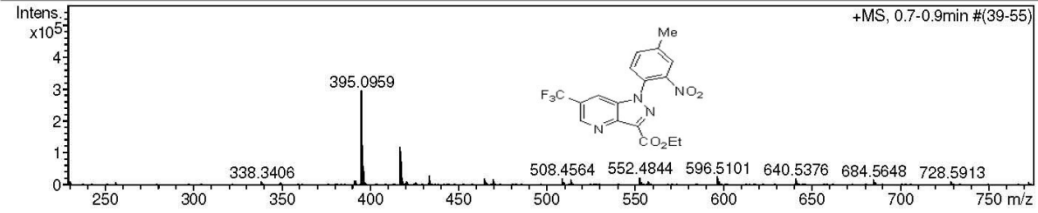

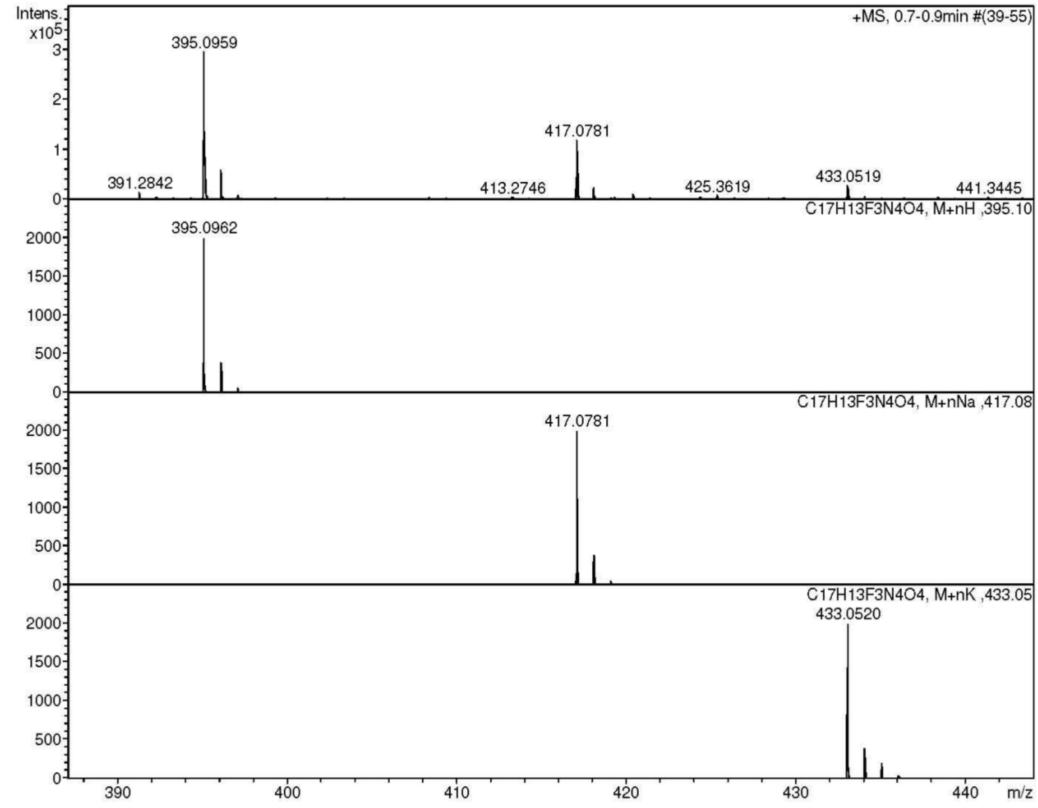

^{1}H and ^{13}C NMR spectra of compound **5m** in $CDCl_3$

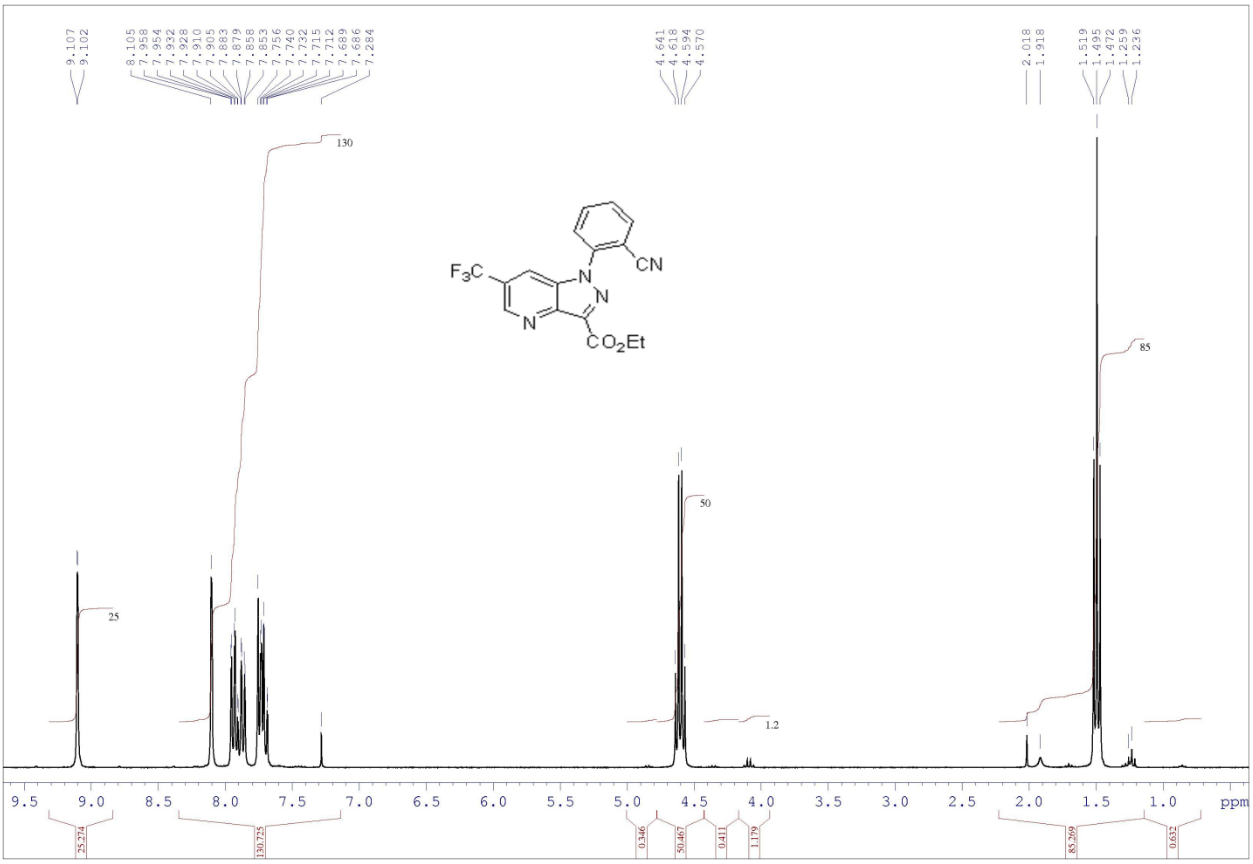

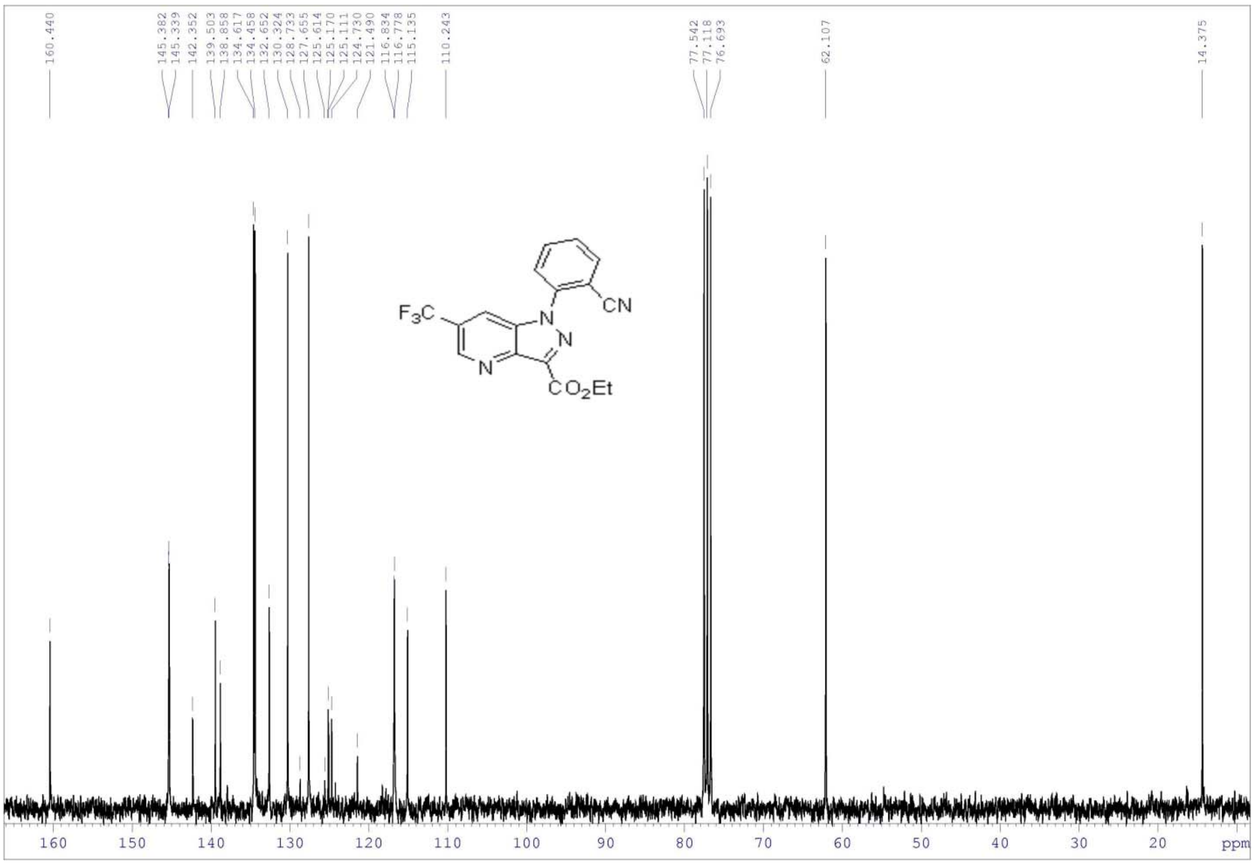

HRMS spectrum of compound 5m

Display Report

Analysis Info

Analysis Name	D:\Data\Kolotyrkina\2022\Bastrakov\1221016.d	Acquisition Date	21.12.2022 11:09:08
Method	tune_low.m	Operator	BDAL@DE
Sample Name	/LPIK VN-610	Instrument / Ser#	micrOTOF 10248
Comment	C17H11F3N4O2 mH 361.0906 calibrant added CH3CN		

Acquisition Parameter

Source Type	ESI	Ion Polarity	Positive	Set Nebulizer	0.4 Bar
Focus	Not active			Set Dry Heater	180 °C
Scan Begin	50 m/z	Set Capillary	4500 V	Set Dry Gas	4.0 l/min
Scan End	2500 m/z	Set End Plate Offset	-500 V	Set Divert Valve	Waste

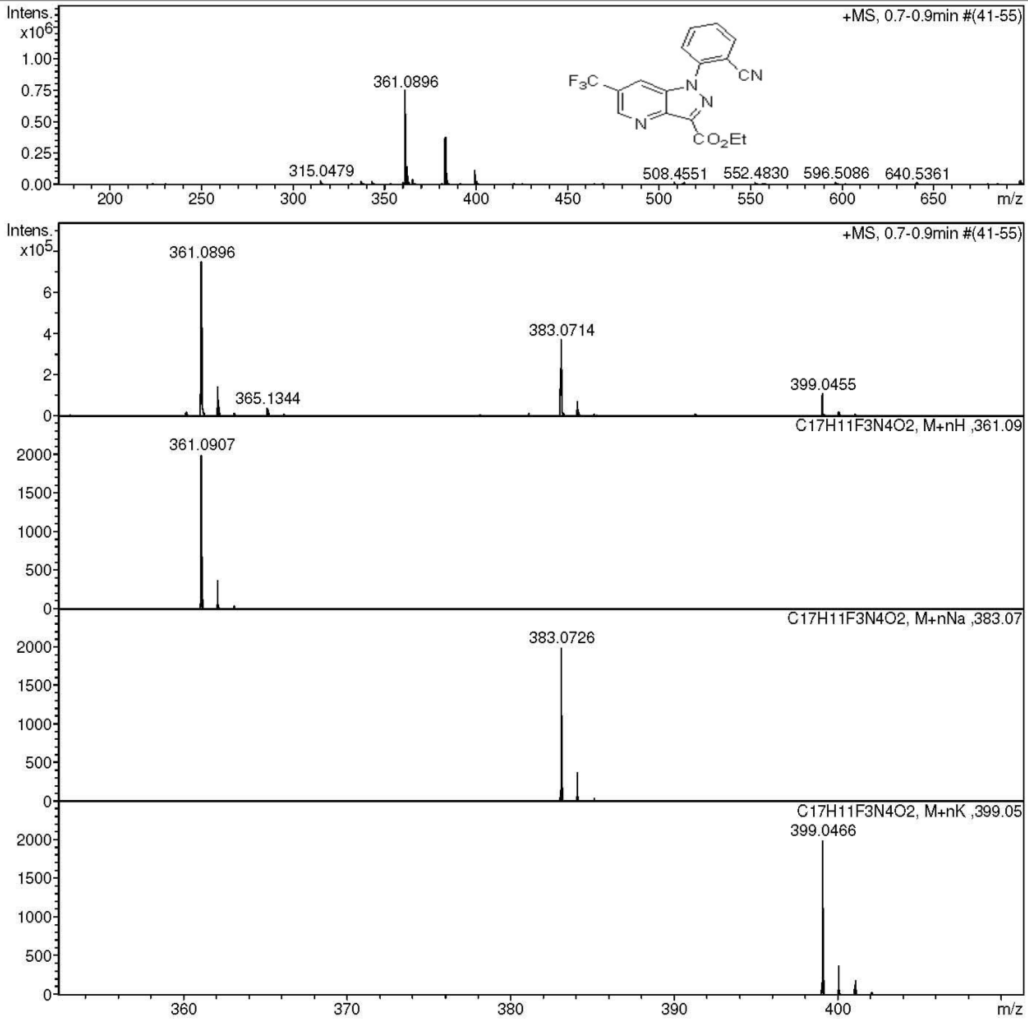

^{1}H and ^{13}C NMR spectra of compound **5n** in $CDCl_3$

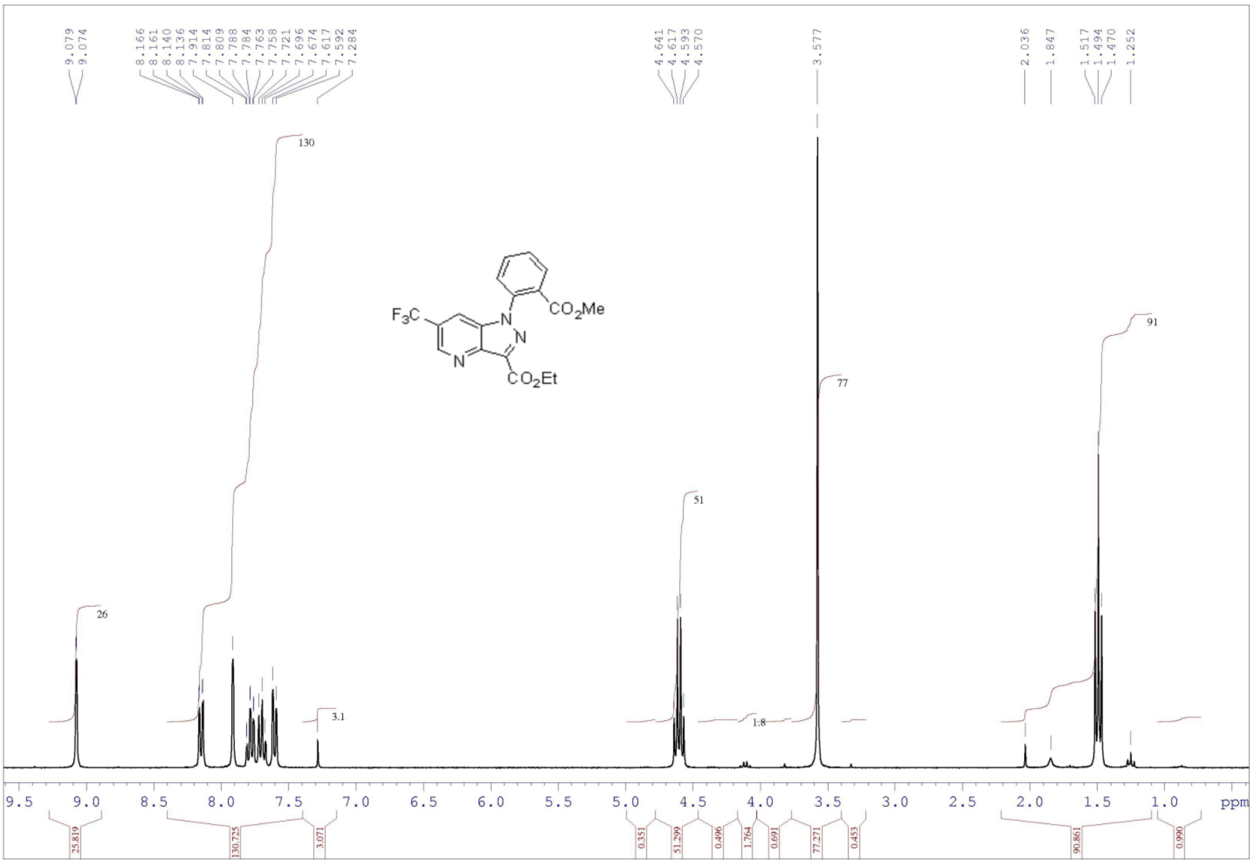

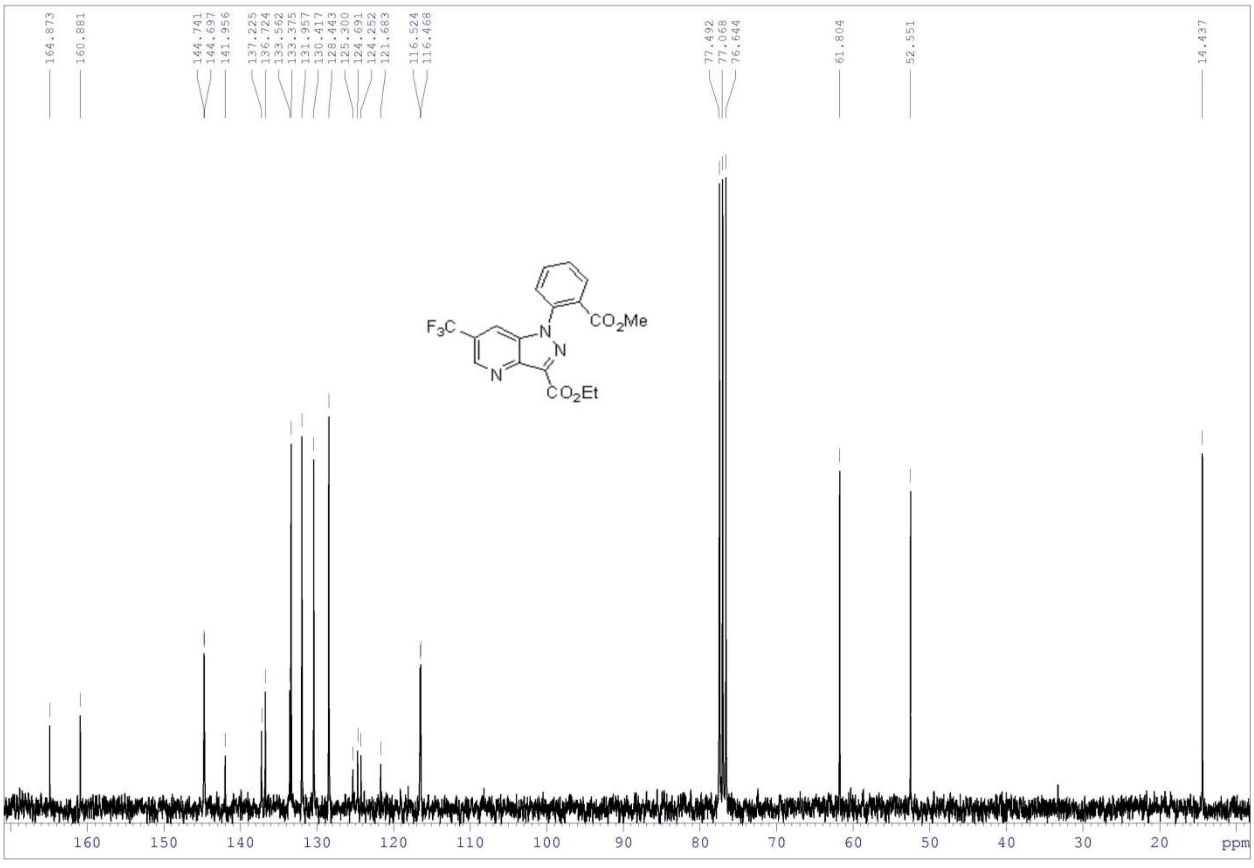

HRMS spectrum of compound 5n

Display Report

Analysis Info

Analysis Name	D:\Data\Kolotyrkina\2022\Bastrakov\1221017.d	Acquisition Date	21.12.2022 11:15:21
Method	tune_low.m	Operator	BDAL@DE
Sample Name	/LPIK VN-613	Instrument / Ser#	micrOTOF 10248
Comment	C18H14F3N3O4 mH 394.1009 calibrant added CH3CN		

Acquisition Parameter

Source Type	ESI	Ion Polarity	Positive	Set Nebulizer	0.4 Bar
Focus	Not active			Set Dry Heater	180 °C
Scan Begin	50 m/z	Set Capillary	4500 V	Set Dry Gas	4.0 l/min
Scan End	2500 m/z	Set End Plate Offset	-500 V	Set Divert Valve	Waste

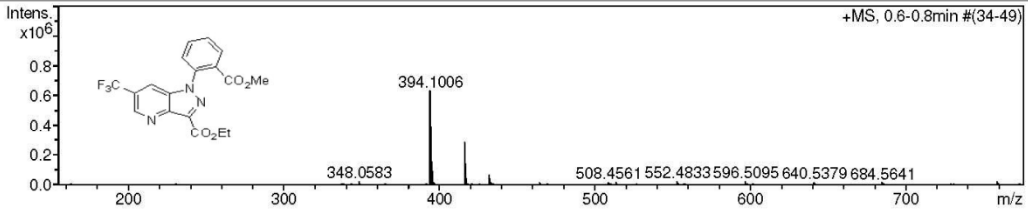

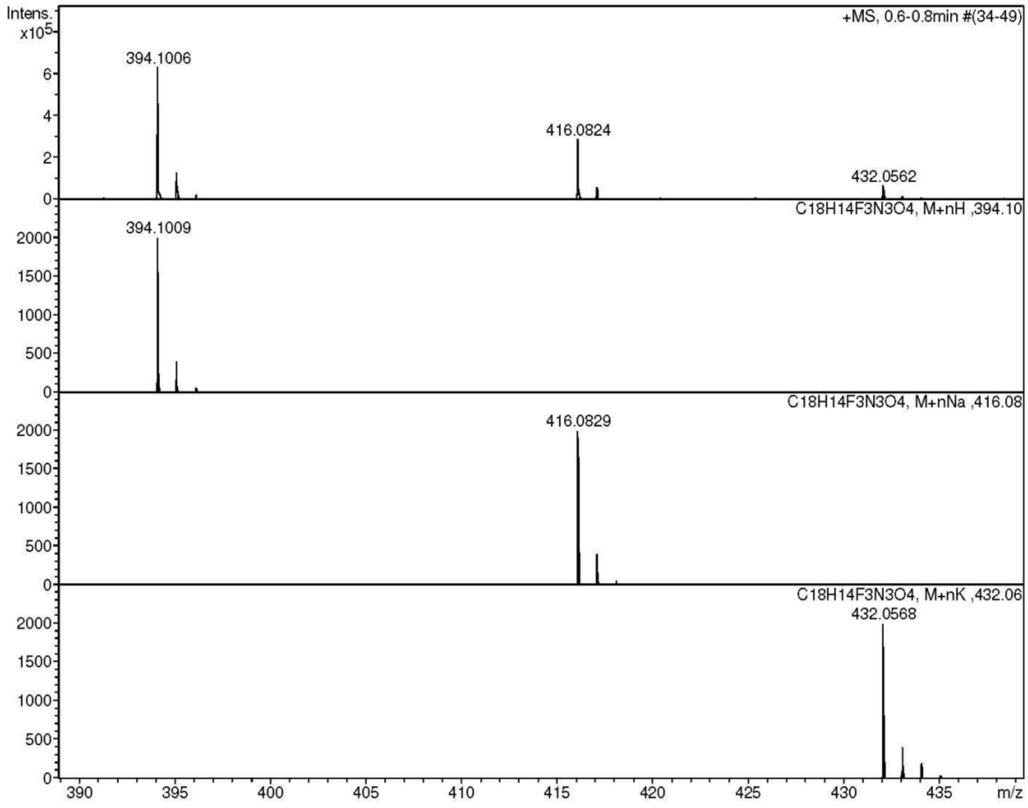

^{1}H and ^{13}C NMR spectra of compound **5o** in $CDCl_3$

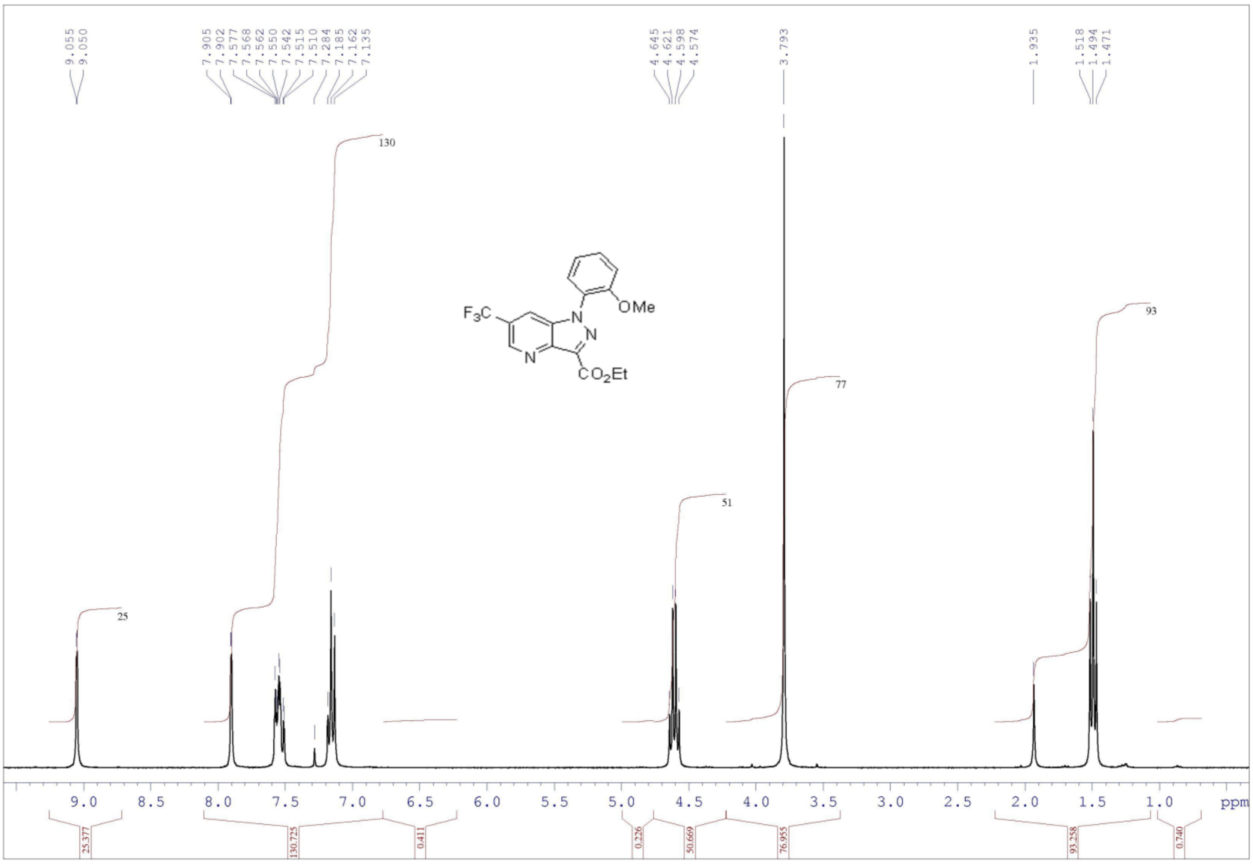

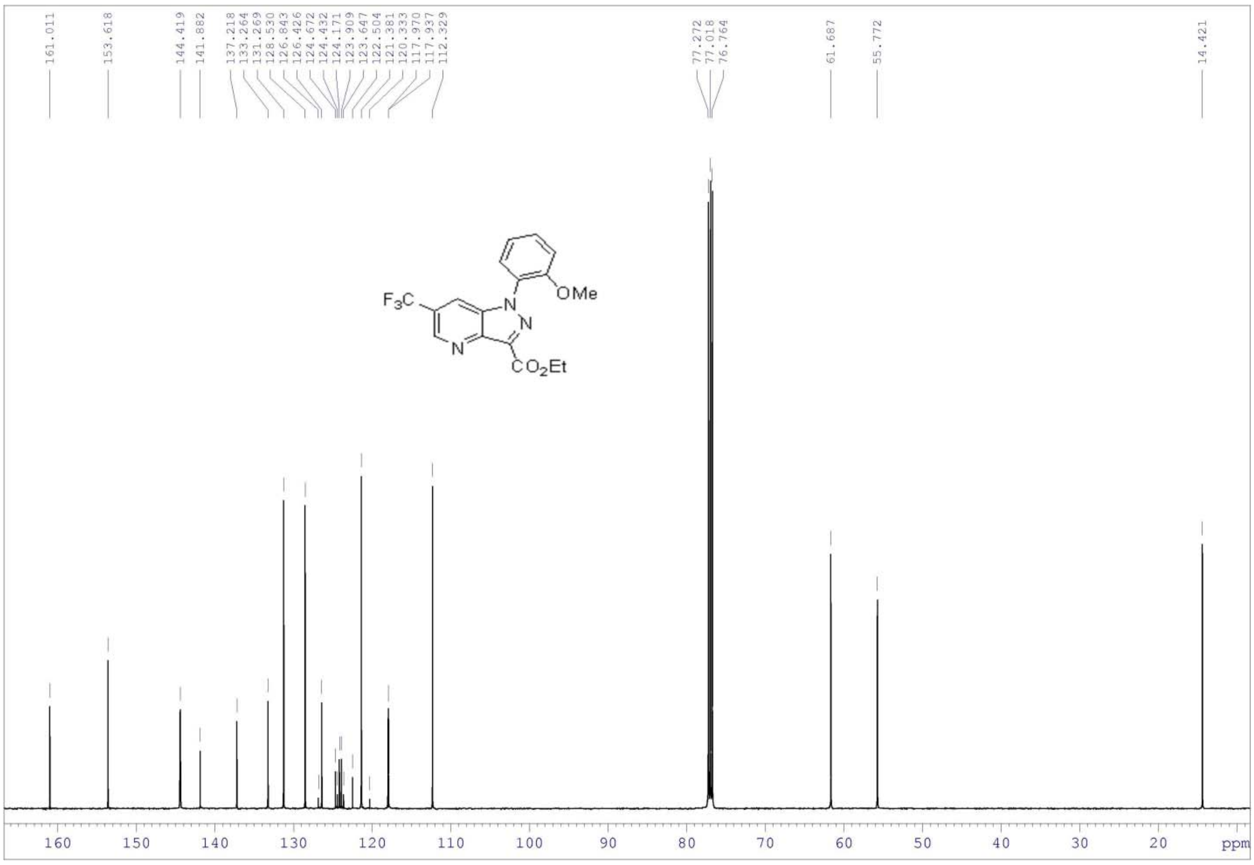

HRMS spectrum of compound 5o

Display Report

Analysis Info

Analysis Name	D:\Data\Kolotyrkina\2022\Bastrakov\1221028.d	Acquisition Date	21.12.2022 14:40:42
Method	tune_low.m	Operator	BDAL@DE
Sample Name	/LPIK VN-615	Instrument / Ser#	micrOTOF 10248
Comment	C17H14F3N3O3 mH 366.1060 calibrant added CH3CN		

Acquisition Parameter

Source Type	ESI	Ion Polarity	Positive	Set Nebulizer	0.4 Bar
Focus	Not active			Set Dry Heater	180 °C
Scan Begin	50 m/z	Set Capillary	4500 V	Set Dry Gas	4.0 l/min
Scan End	2500 m/z	Set End Plate Offset	-500 V	Set Divert Valve	Waste

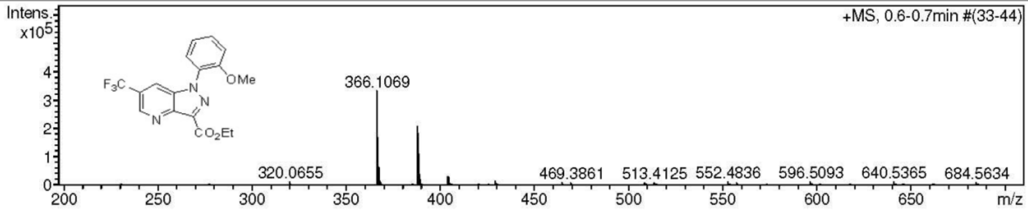

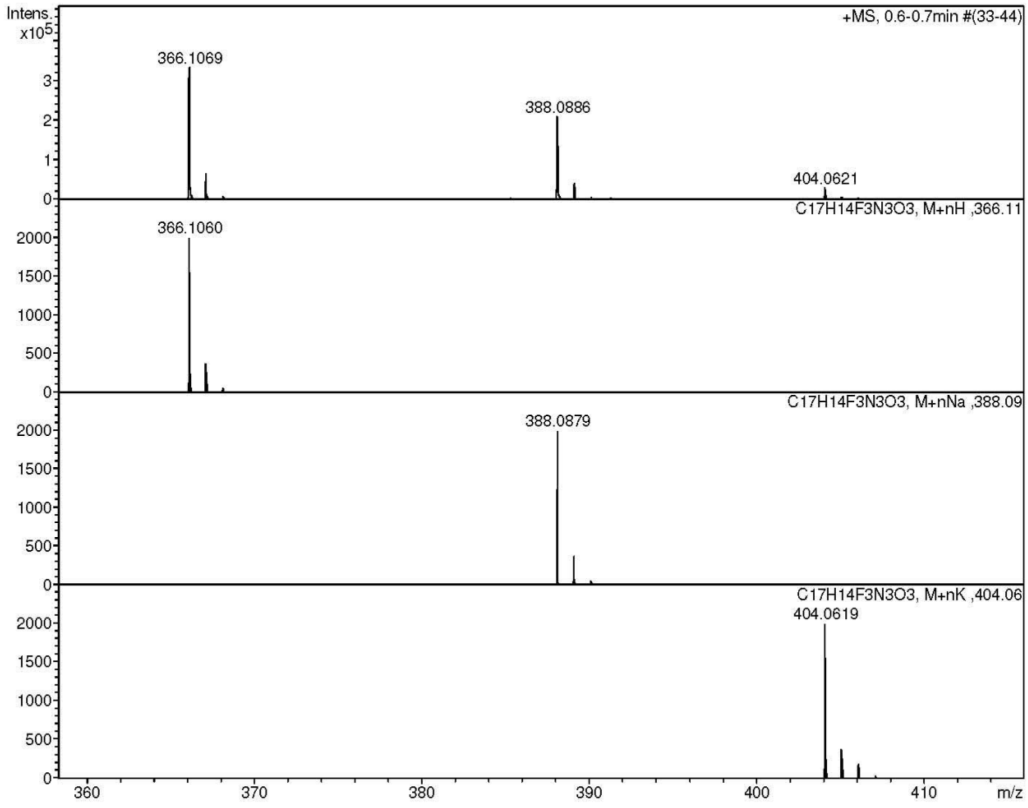

^{1}H and ^{13}C NMR spectra of compound **5p** in $CDCl_3$

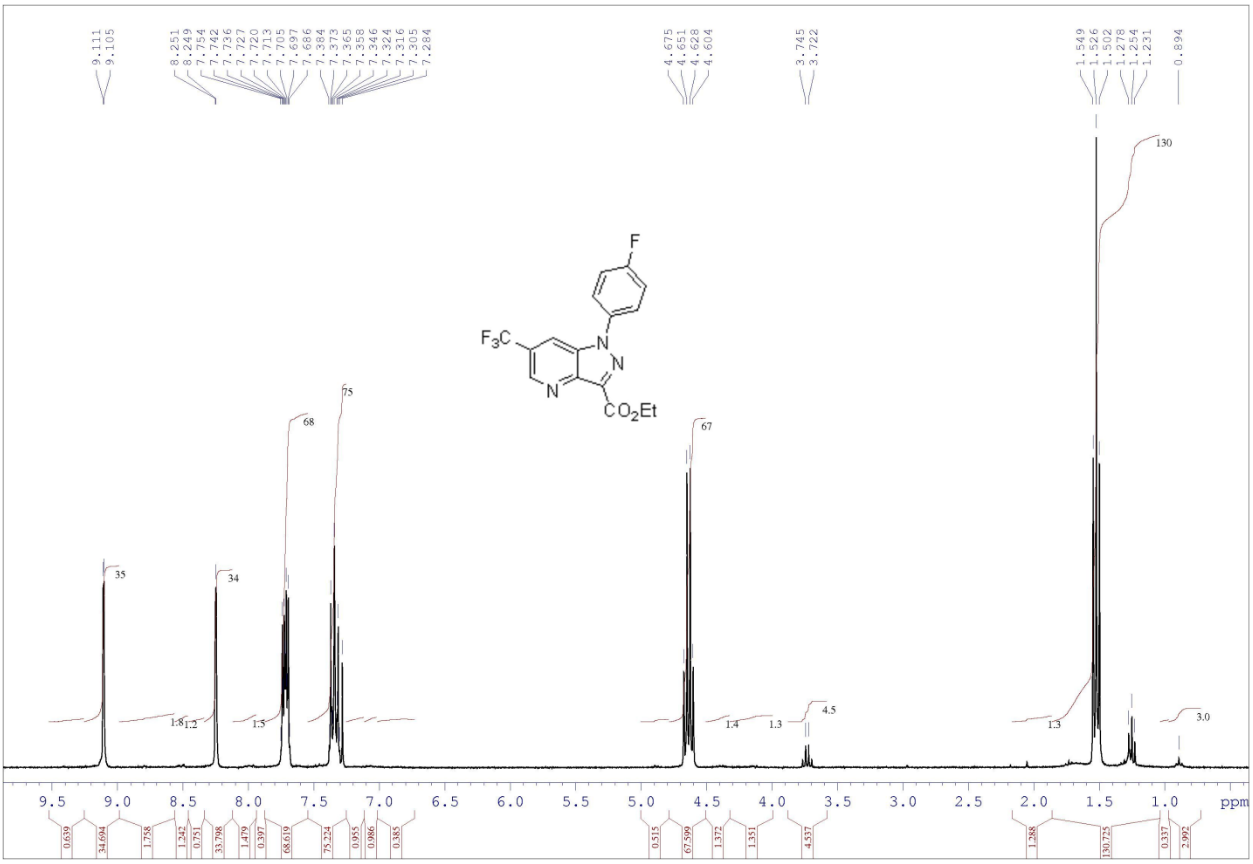

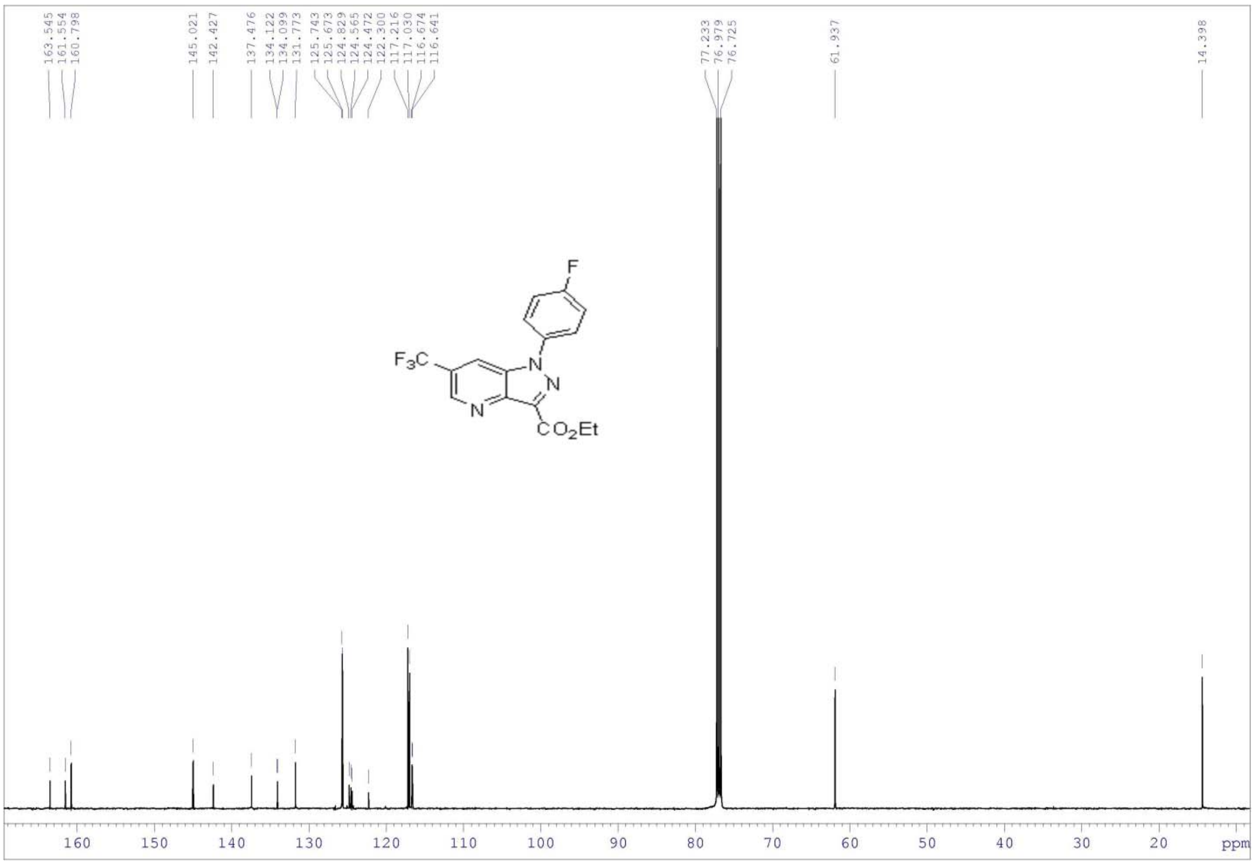

HRMS spectrum of compound 5p

Display Report

Analysis Info

Analysis Name	D:\Data\Kolotyrkina\2022\Bastrakov\1221029.d	Acquisition Date	21.12.2022 14:45:02
Method	tune_low.m	Operator	BDAL@DE
Sample Name	/LPIK VN-639	Instrument / Ser#	micrOTOF 10248
Comment	C16H11F4N3O2 mH 354.0860 calibrant added CH3CN		

Acquisition Parameter

Source Type	ESI	Ion Polarity	Positive	Set Nebulizer	0.4 Bar
Focus	Not active			Set Dry Heater	180 °C
Scan Begin	50 m/z	Set Capillary	4500 V	Set Dry Gas	4.0 l/min
Scan End	2500 m/z	Set End Plate Offset	-500 V	Set Divert Valve	Waste

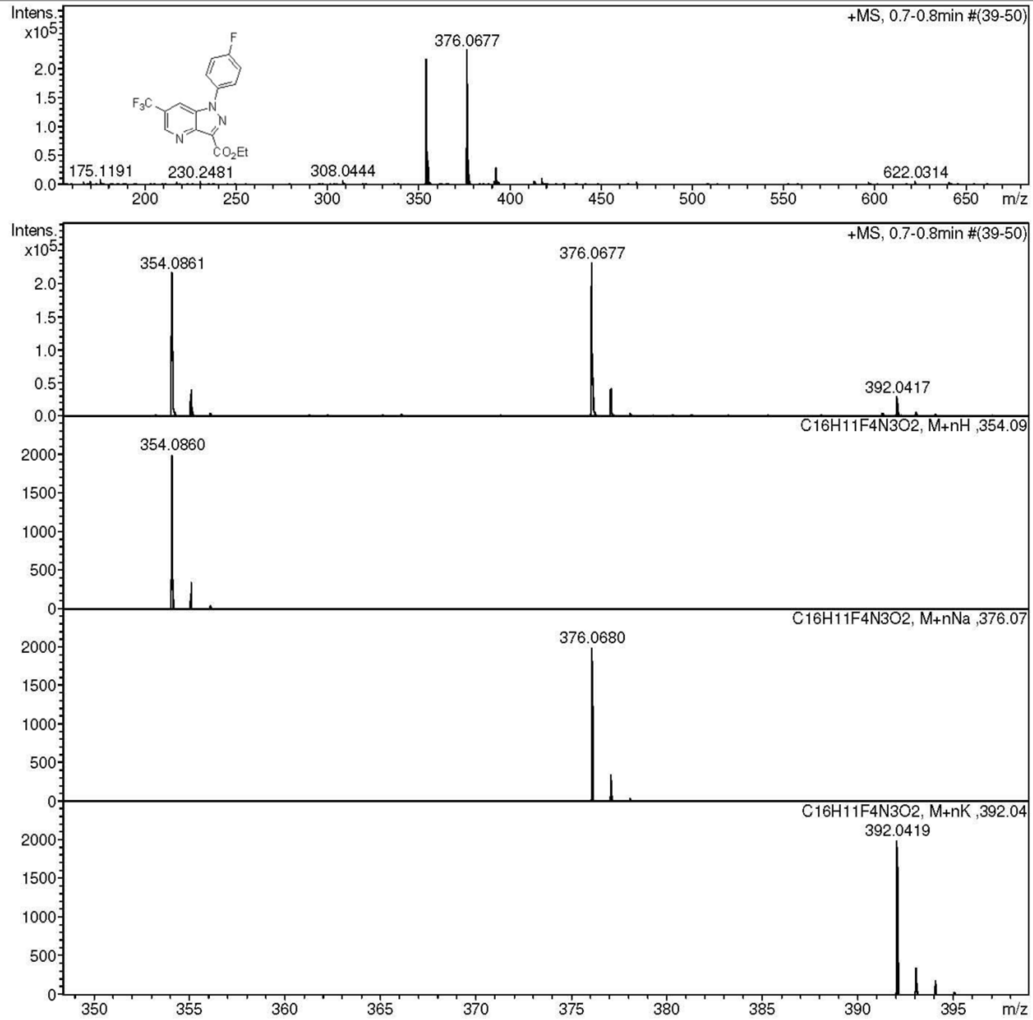

^{1}H and ^{13}C NMR spectra of compound **5q** in $CDCl_3$

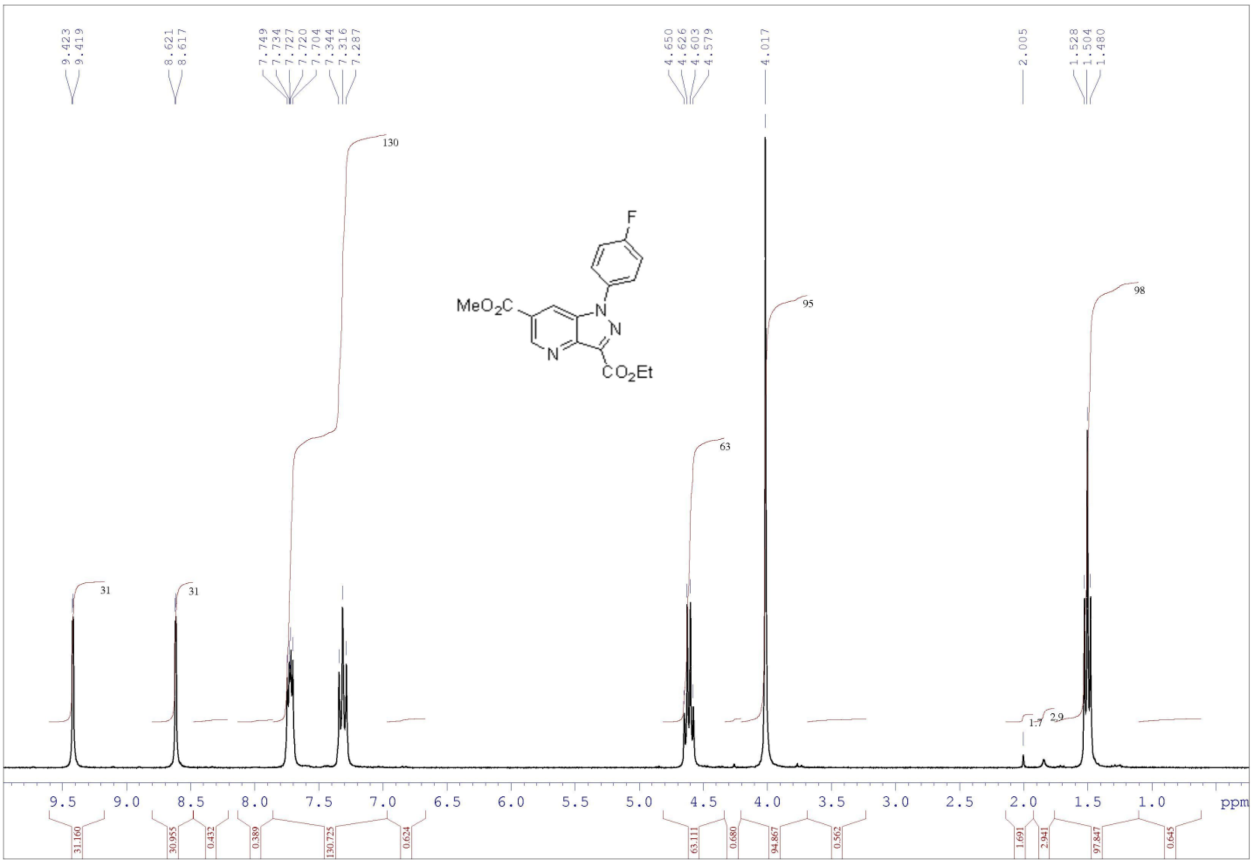

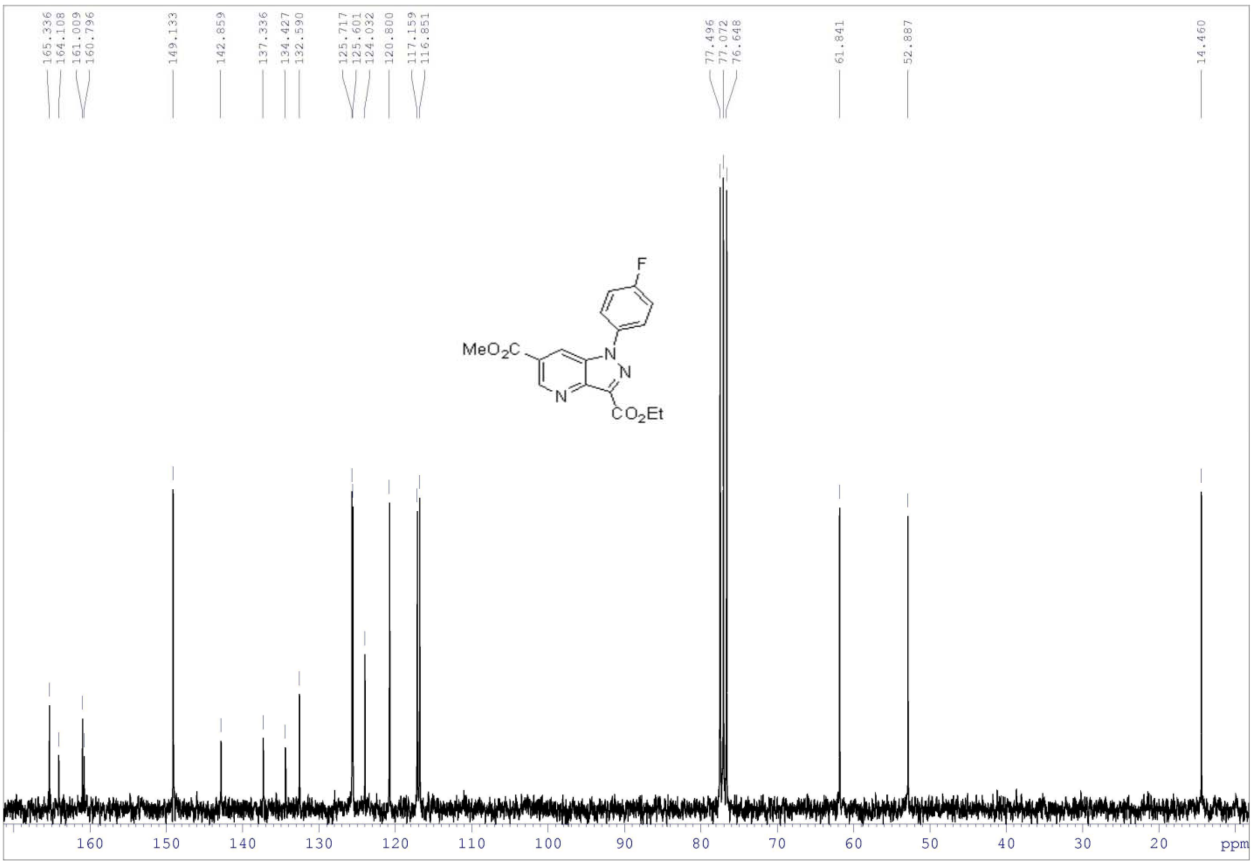

HRMS spectrum of compound 5q

Display Report

Analysis Info

Analysis Name	D:\Data\Kolotyrkina\2022\Bastrakov\1220018.d	Acquisition Date	20.12.2022 15:09:44
Method	tune_low.m	Operator	BDAL@DE
Sample Name	/LPIK VN-653	Instrument / Ser#	micrOTOF 10248
Comment	C17H14FN3O4 mH 344.1041 calibrant added CH3CN		

Acquisition Parameter

Source Type	ESI	Ion Polarity	Positive	Set Nebulizer	0.4 Bar
Focus	Not active			Set Dry Heater	180 °C
Scan Begin	50 m/z	Set Capillary	4500 V	Set Dry Gas	4.0 l/min
Scan End	2500 m/z	Set End Plate Offset	-500 V	Set Divert Valve	Waste

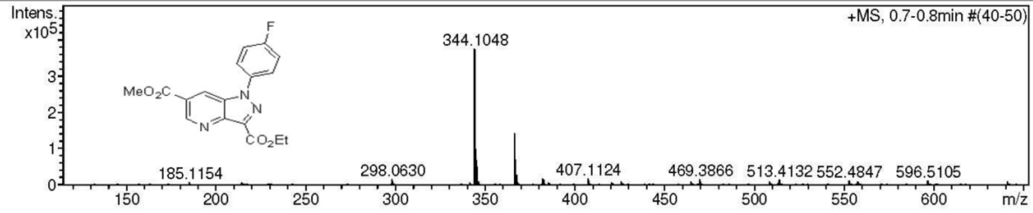

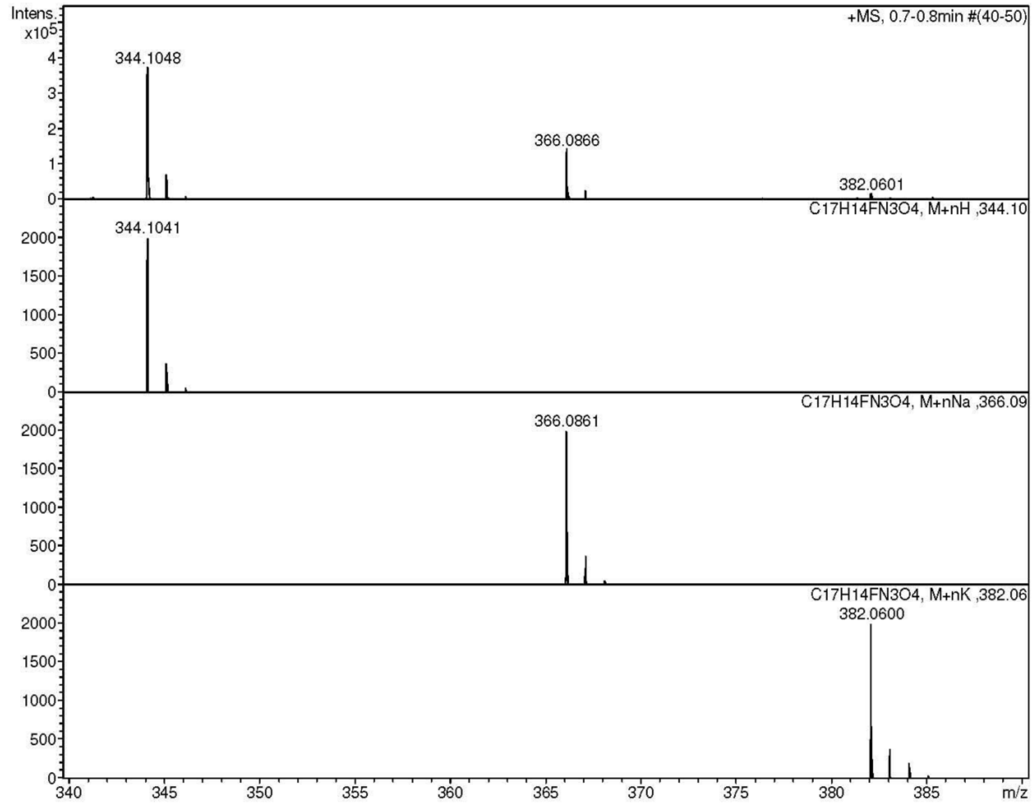

^{1}H and ^{13}C NMR spectra of compound **5q'** in $CDCl_3$

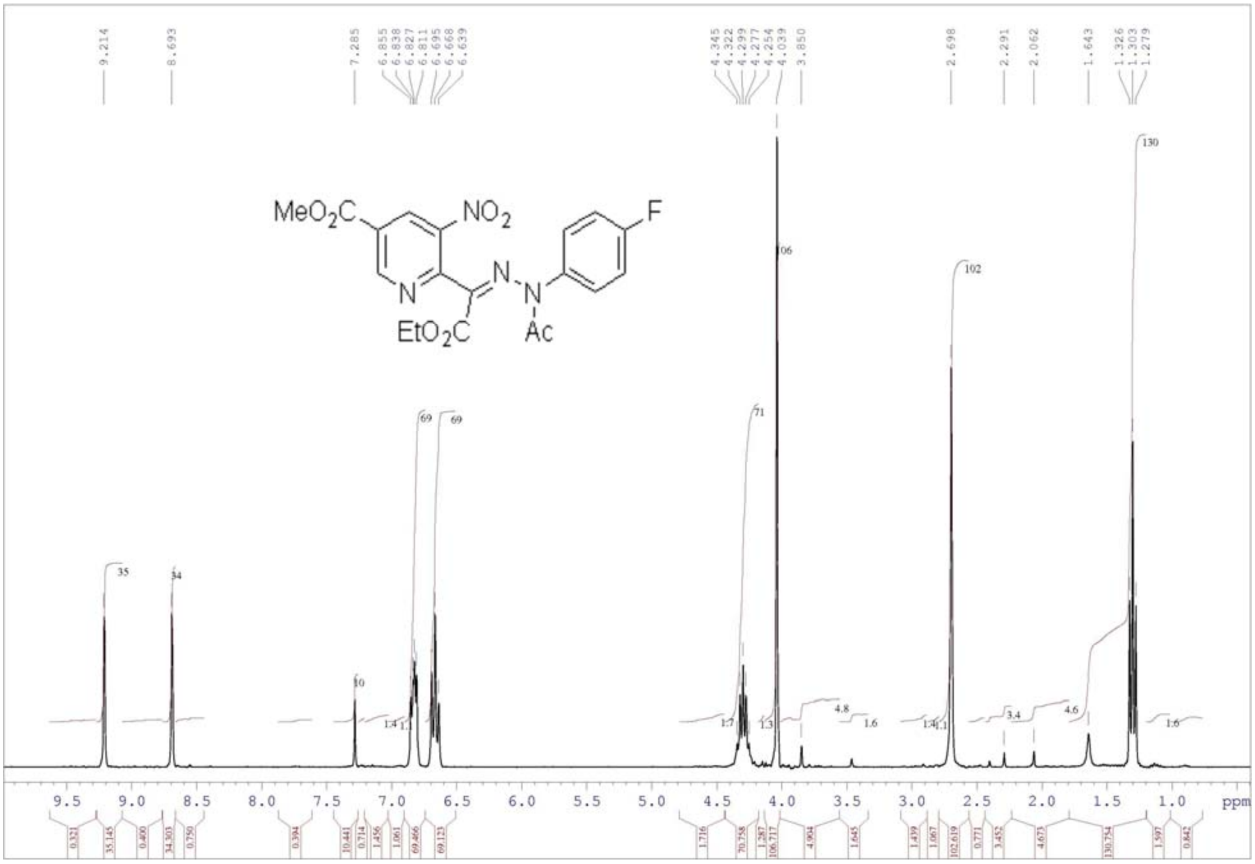

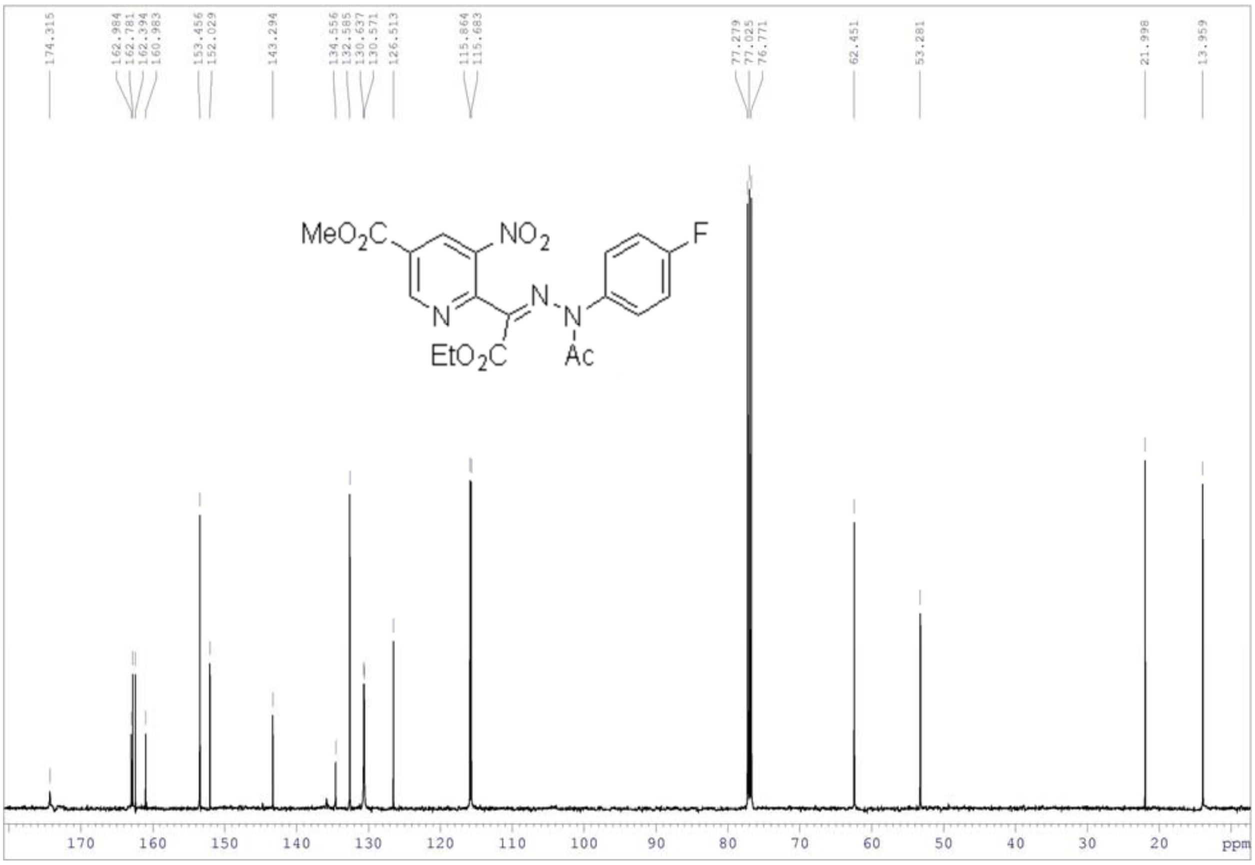

HRMS spectrum of compound 5q’

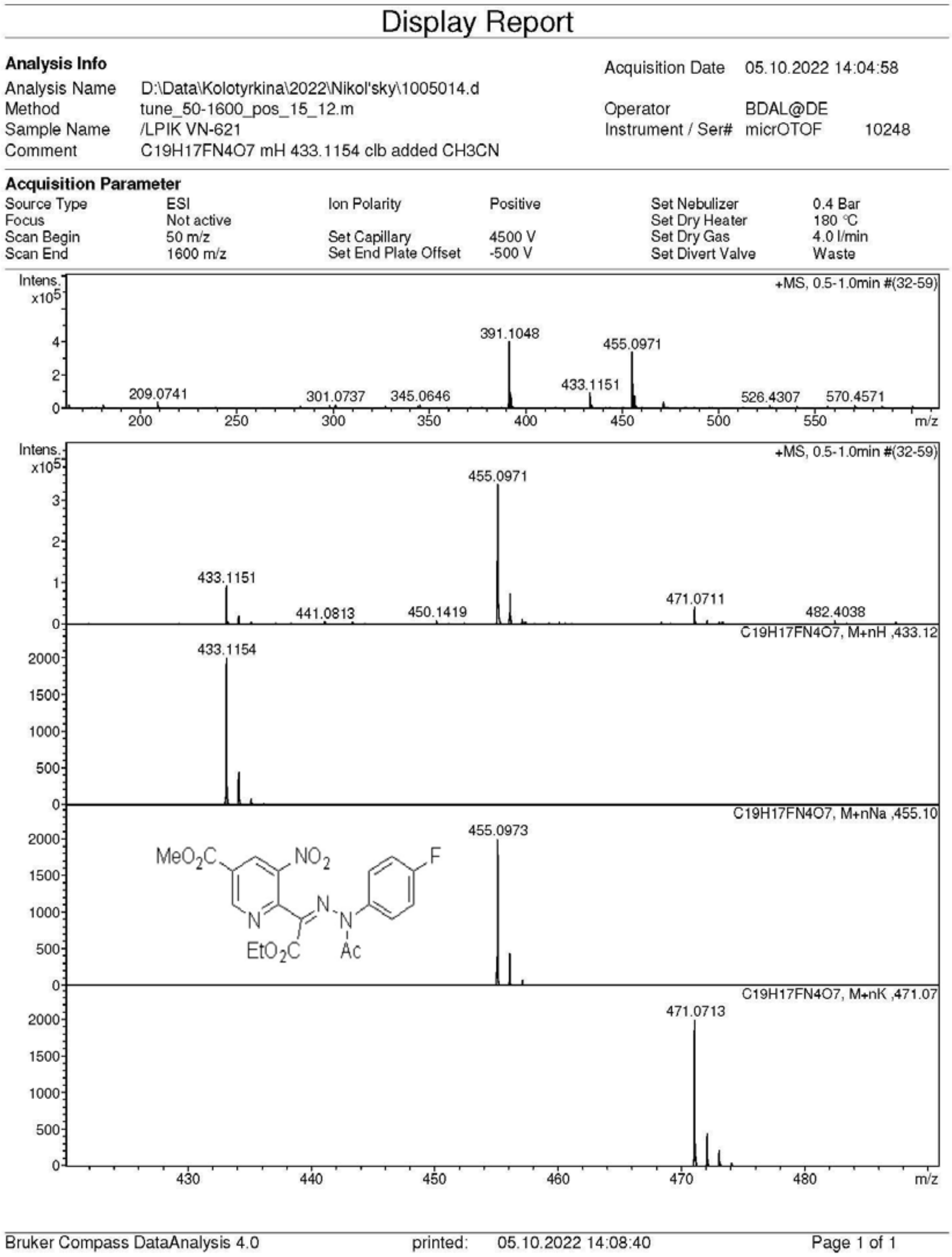

^1H and ^{13}C NMR spectra of compound **5r** in DMSO-d_6

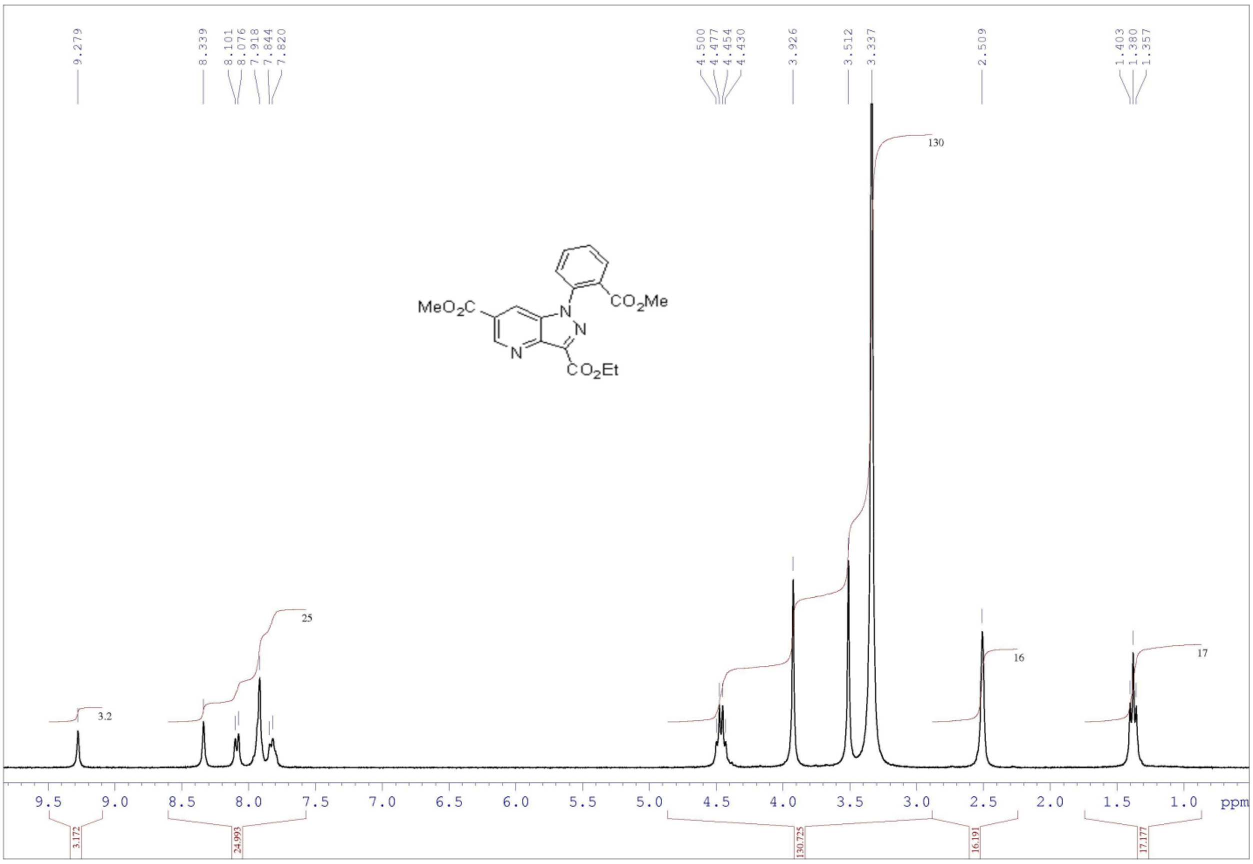

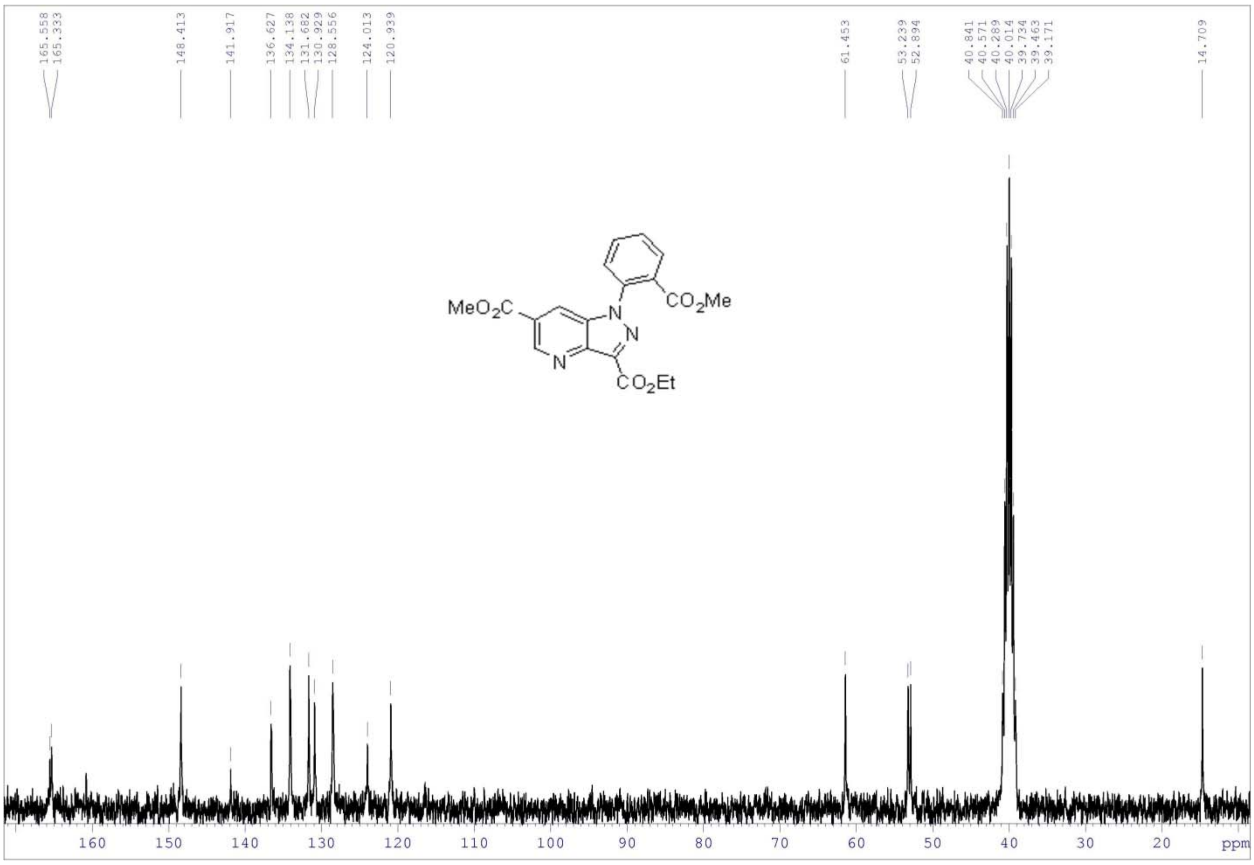

HRMS spectrum of compound 5r

Display Report

Analysis Info

Analysis Name	D:\Data\Kolotyrkina\2022\Bastrakov\1221033.d	Acquisition Date	21.12.2022 15:03:57
Method	tune_low.m	Operator	BDAL@DE
Sample Name	/LPIK VN-618	Instrument / Ser#	micrOTOF 10248
Comment	C19H18N3O6 mH 384.1190 calibrant added CH3CN		

Acquisition Parameter

Source Type	ESI	Ion Polarity	Positive	Set Nebulizer	0.4 Bar
Focus	Not active			Set Dry Heater	180 °C
Scan Begin	50 m/z	Set Capillary	4500 V	Set Dry Gas	4.0 l/min
Scan End	2500 m/z	Set End Plate Offset	-500 V	Set Divert Valve	Waste

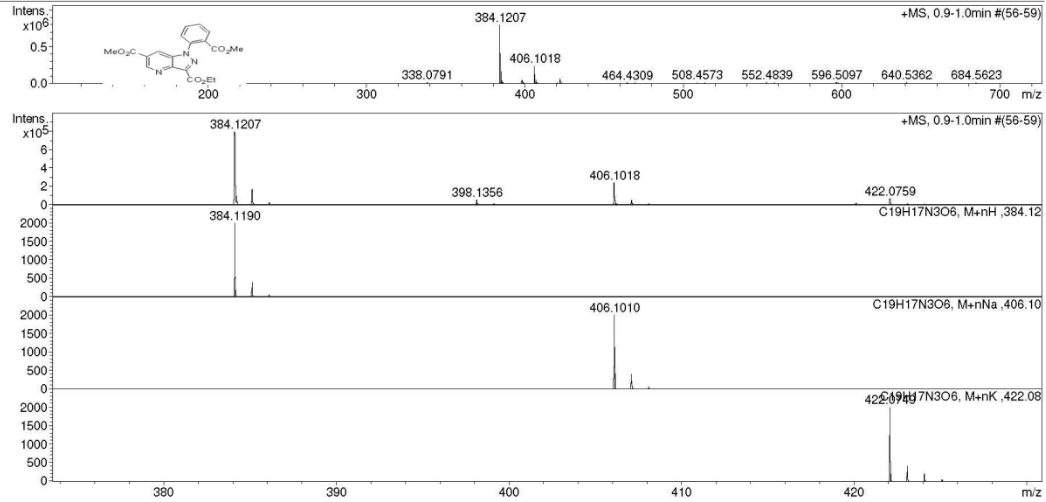

^{1}H and ^{13}C NMR spectra of compound **5s** in $CDCl_3$

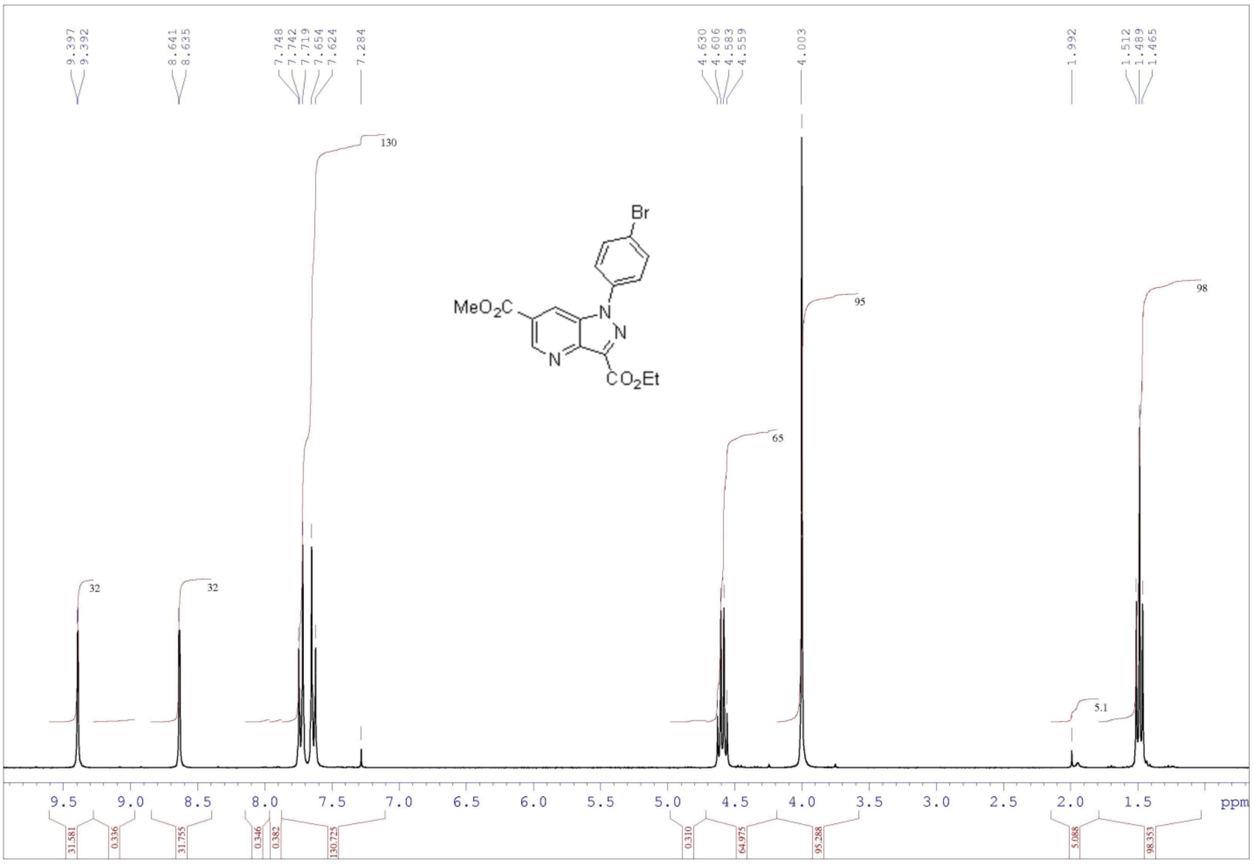

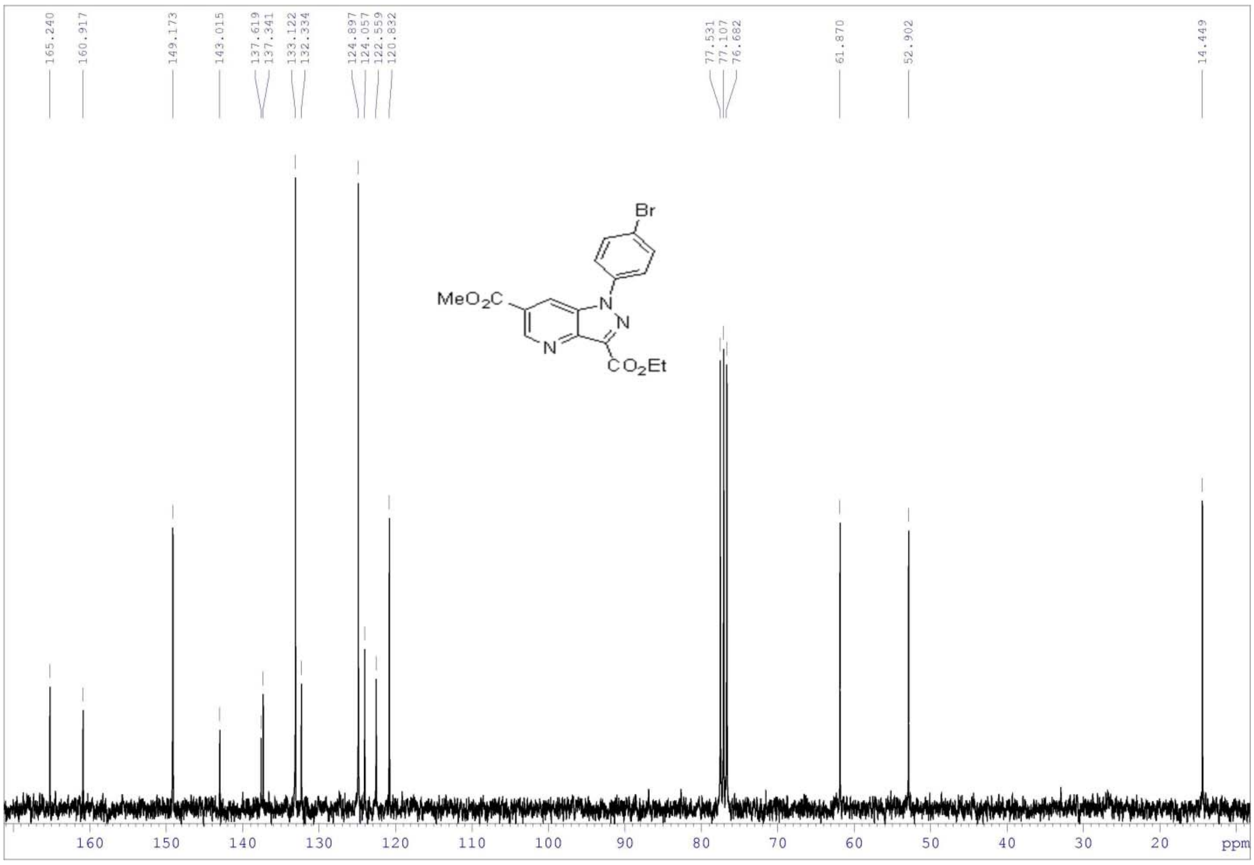

HRMS spectrum of compound 5s

Display Report

Analysis Info

Analysis Name	D:\Data\Kolotyrkina\2022\Bastrakov\1221034.d	Acquisition Date	21.12.2022 15:10:39
Method	tune_low.m	Operator	BDAL@DE
Sample Name	/LPIK VN-654	Instrument / Ser#	micrOTOF 10248
Comment	C17H14BrN3O4 mH 404.0240 calibrant added CH3CN		

Acquisition Parameter

Source Type	ESI	Ion Polarity	Positive	Set Nebulizer	0.4 Bar
Focus	Not active			Set Dry Heater	180 °C
Scan Begin	50 m/z	Set Capillary	4500 V	Set Dry Gas	4.0 l/min
Scan End	2500 m/z	Set End Plate Offset	-500 V	Set Divert Valve	Waste

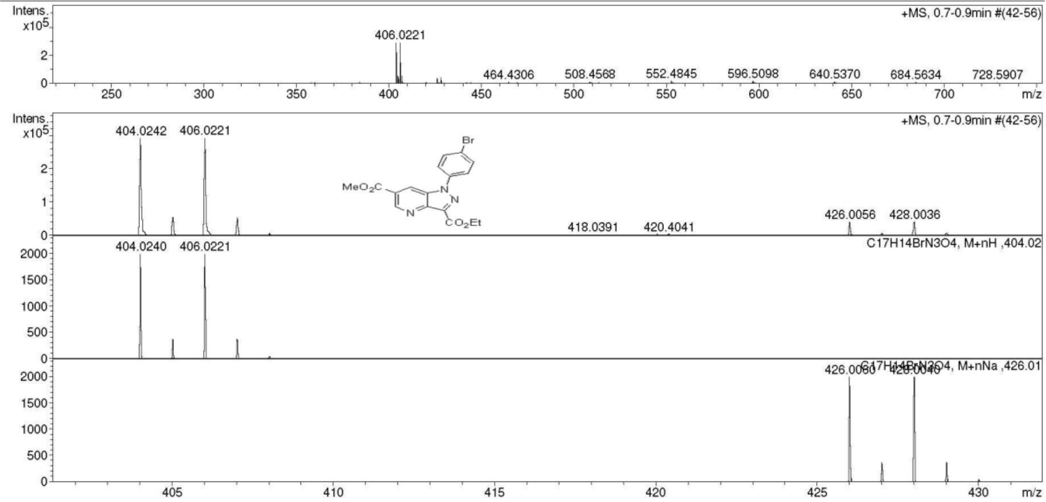

^{1}H spectrum of compound **7a** in $CDCl_3$

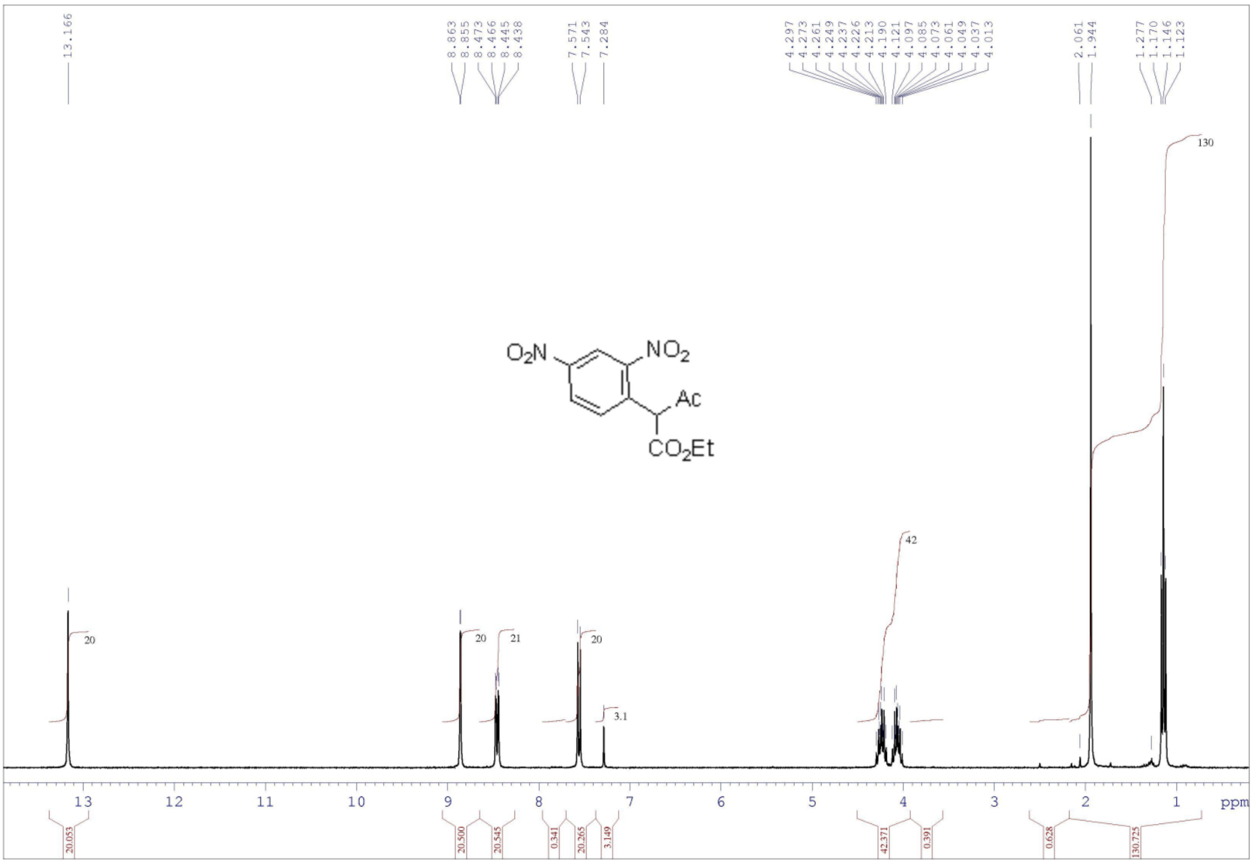

^{1}H and ^{13}C NMR spectra of compound **7b** in $CDCl_3$

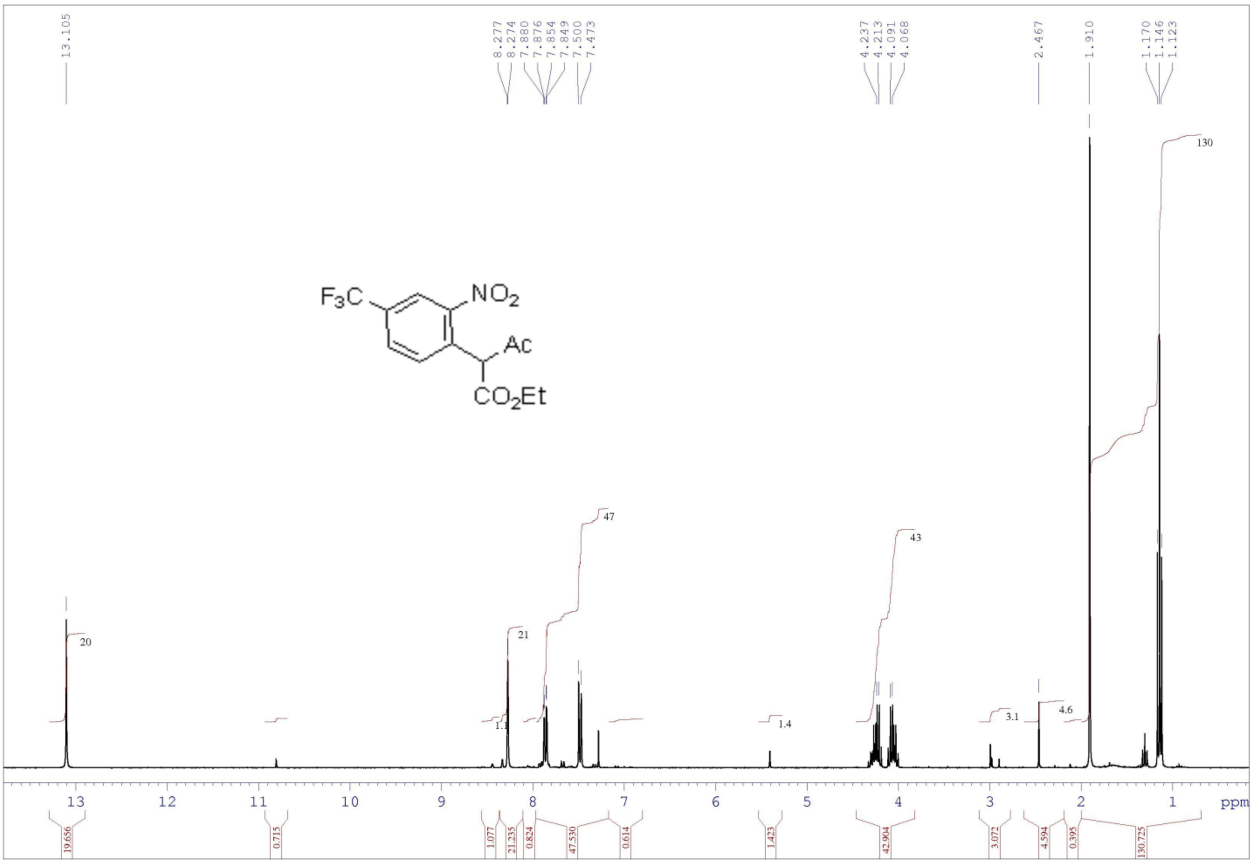

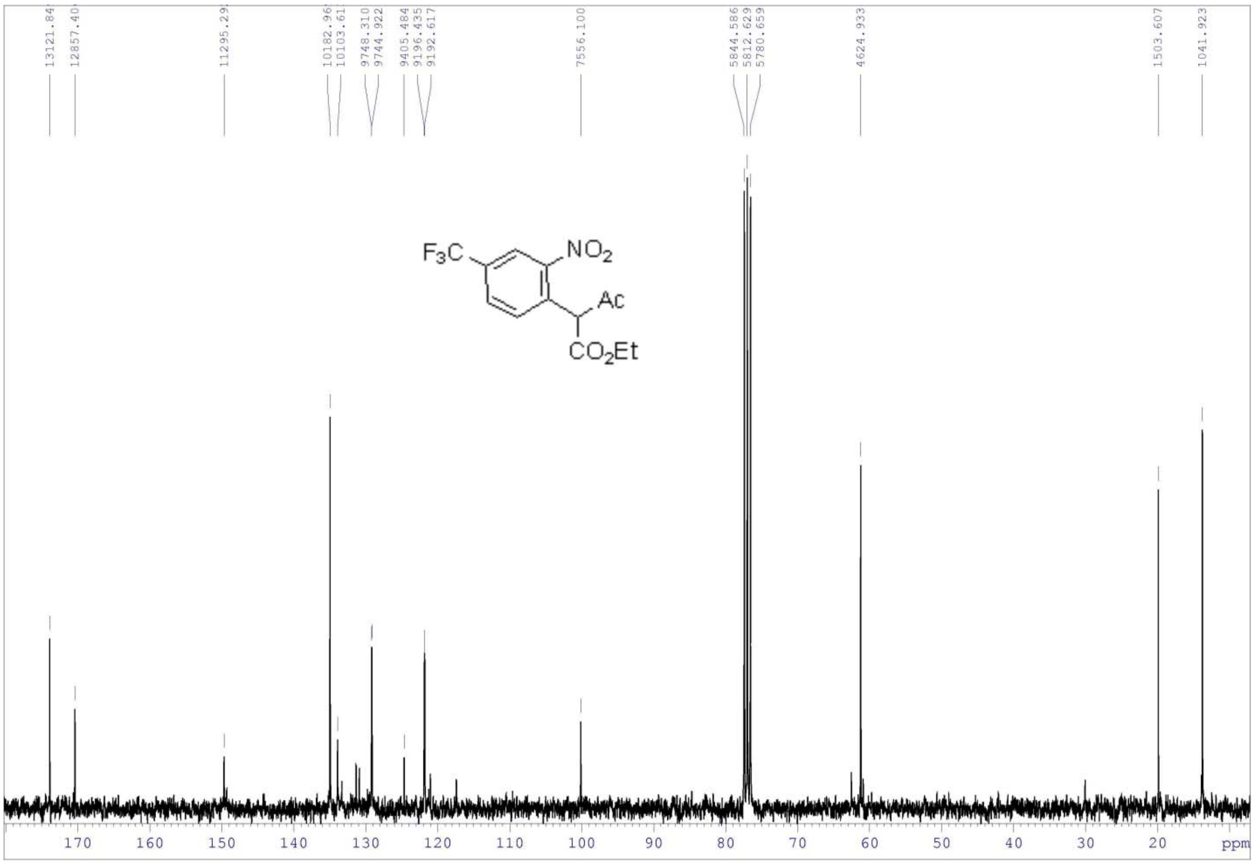

^{1}H NMR spectrum of compound 7c

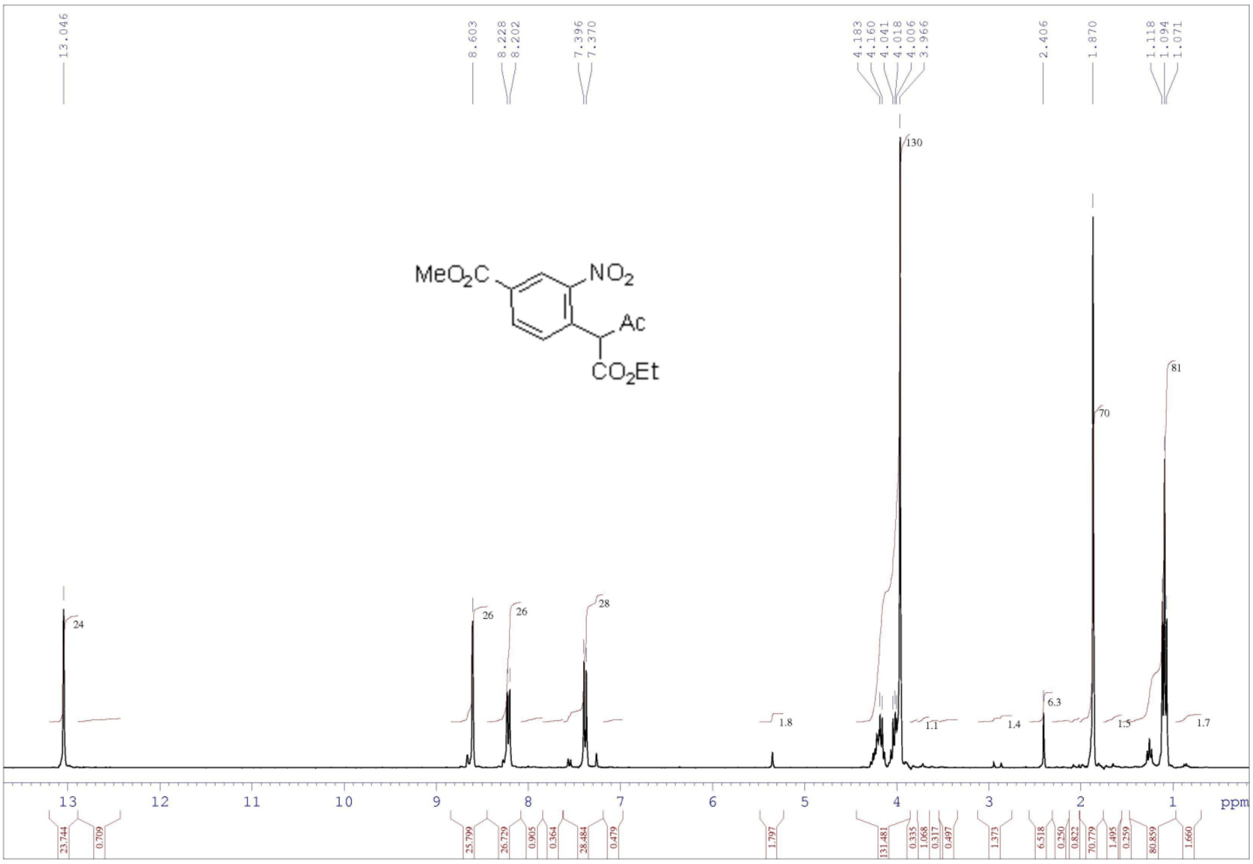

HRMS spectrum of compound 7c

Display Report

Analysis Info

Analysis Name	D:\Data\Kolotyrkina\2022\Bastrakov\1221004.d	Acquisition Date	21.12.2022 9:53:27
Method	tune_low.m	Operator	BDAL@DE
Sample Name	/LPIK YM-19	Instrument / Ser#	micrOTOF 10248
Comment	C14H15NO7 mH 310.0921 calibrant added CH3CN		

Acquisition Parameter

Source Type	ESI	Ion Polarity	Positive	Set Nebulizer	0.4 Bar
Focus	Not active			Set Dry Heater	180 °C
Scan Begin	50 m/z	Set Capillary	4500 V	Set Dry Gas	4.0 l/min
Scan End	2500 m/z	Set End Plate Offset	-500 V	Set Divert Valve	Waste

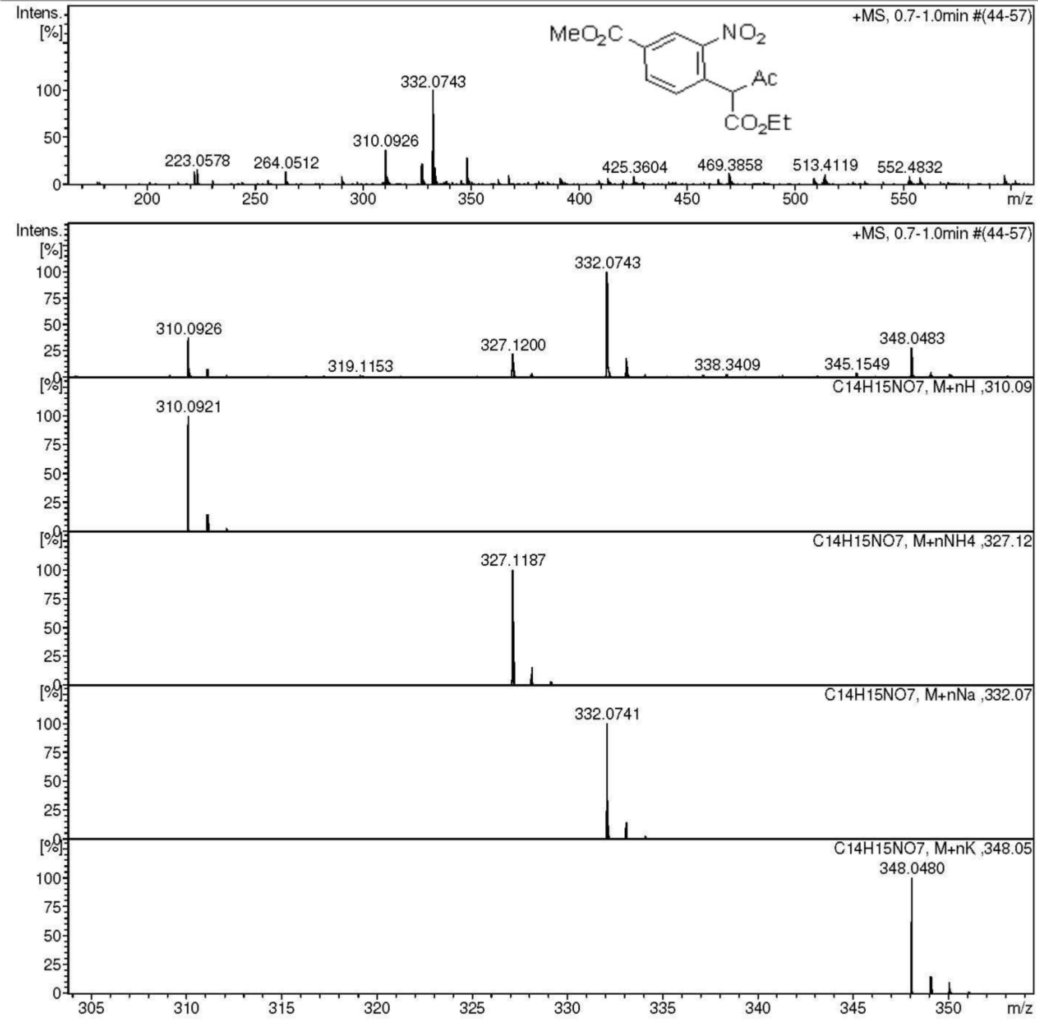

^{1}H NMR spectrum of compound 7d

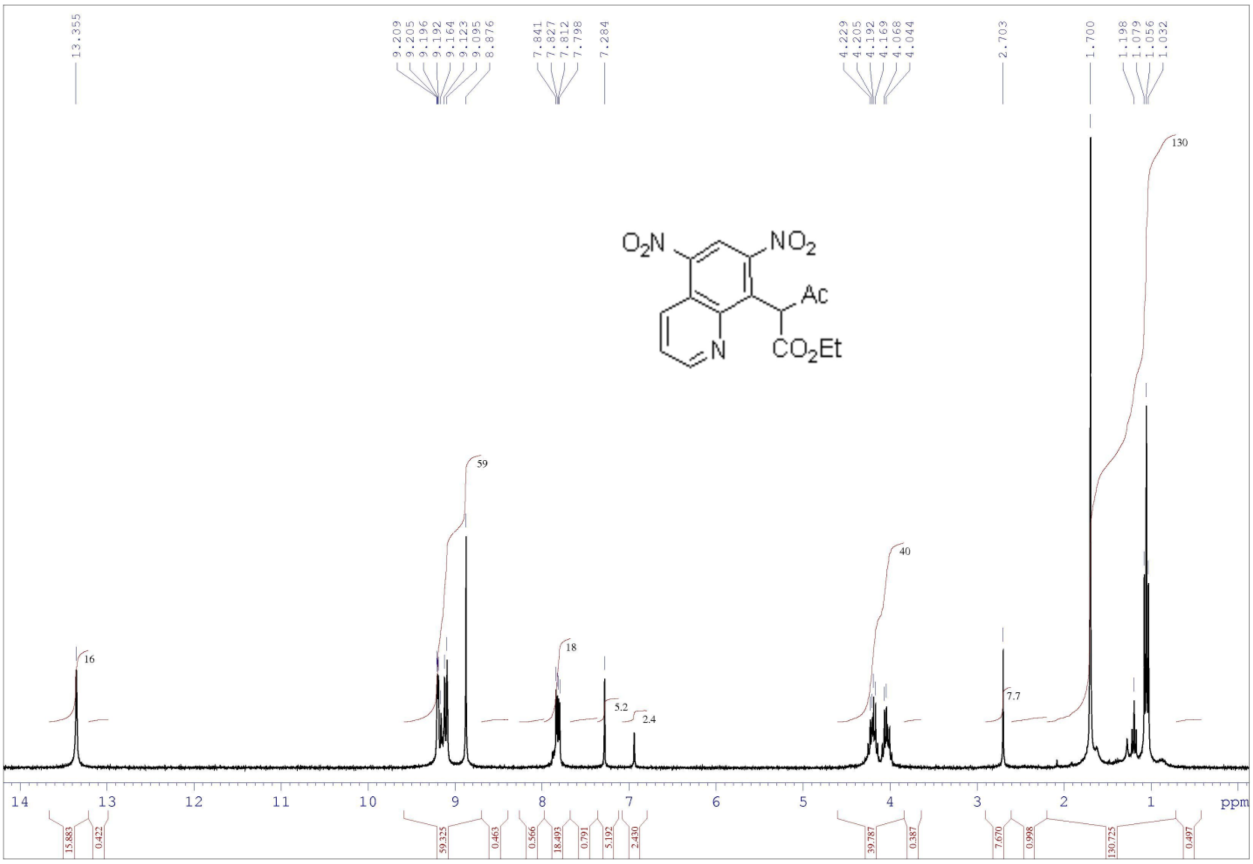

HRMS spectrum of compound 7d

Display Report

Analysis Info

Analysis Name	D:\Data\Kolotyrkina\2022\Bastrakov\1221005.d	Acquisition Date	21.12.2022 10:00:22
Method	tune_low.m	Operator	BDAL@DE
Sample Name	/LPIK YM-25	Instrument / Ser#	micrOTOF 10248
Comment	C15H13N3O7 mH 348.0826 calibrant added CH3CN		

Acquisition Parameter

Source Type	ESI	Ion Polarity	Positive	Set Nebulizer	0.4 Bar
Focus	Not active			Set Dry Heater	180 °C
Scan Begin	50 m/z	Set Capillary	4500 V	Set Dry Gas	4.0 l/min
Scan End	2500 m/z	Set End Plate Offset	-500 V	Set Divert Valve	Waste

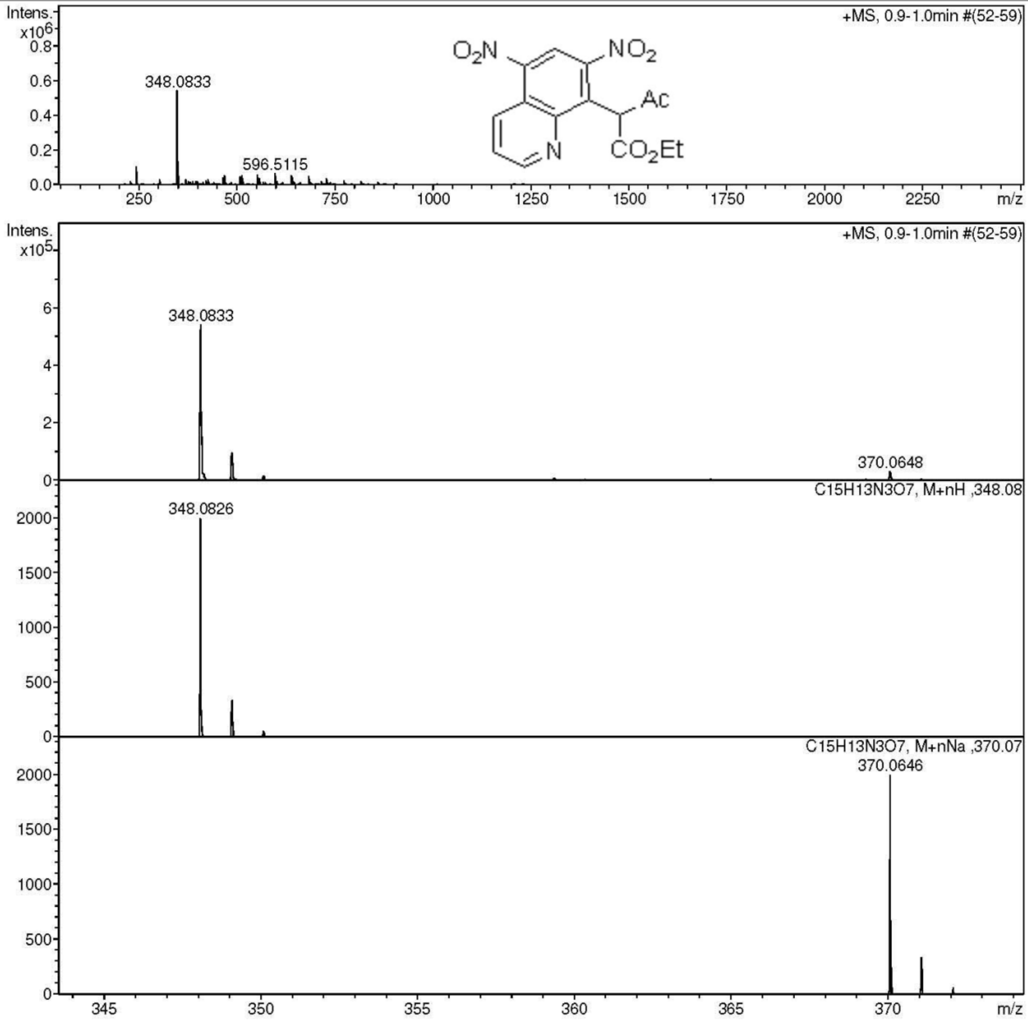

^{1}H and ^{13}C NMR spectra of compound **8a** in $CDCl_3$

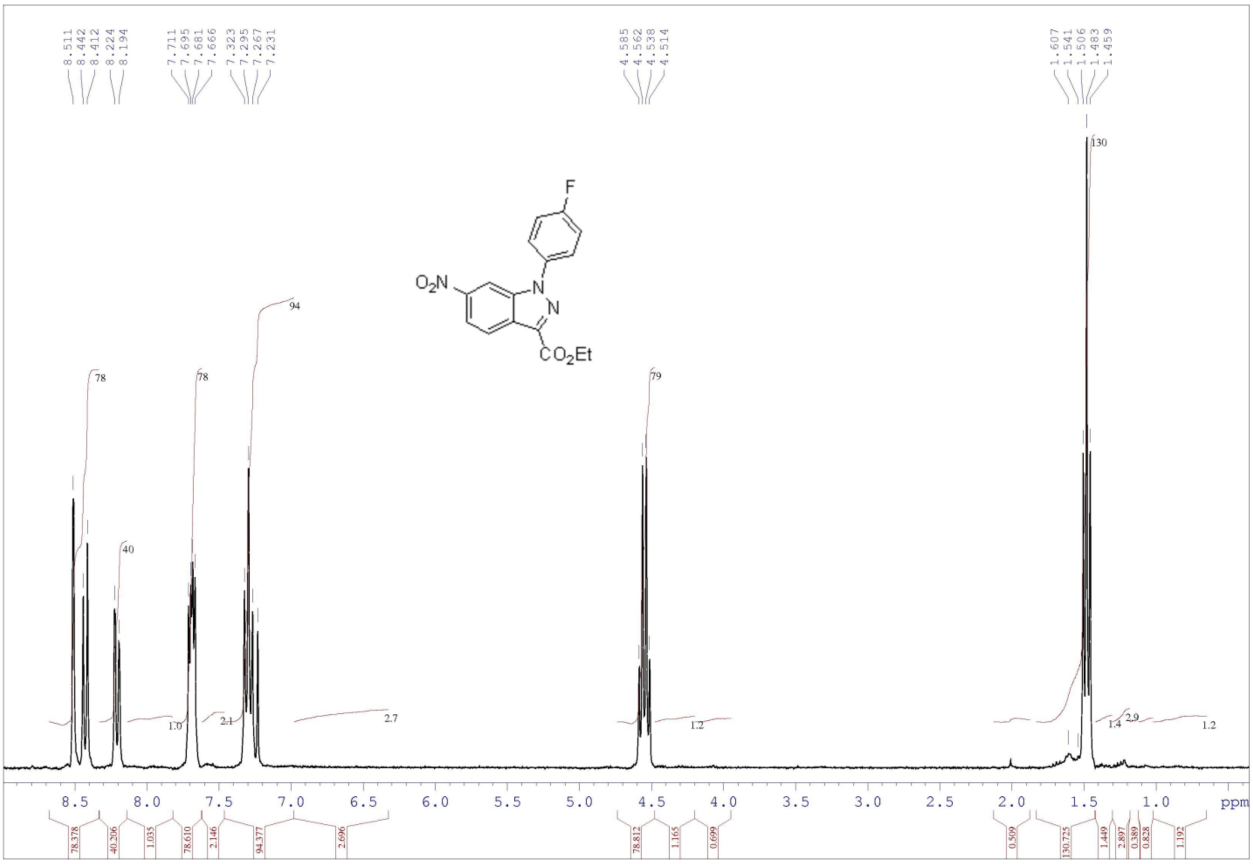

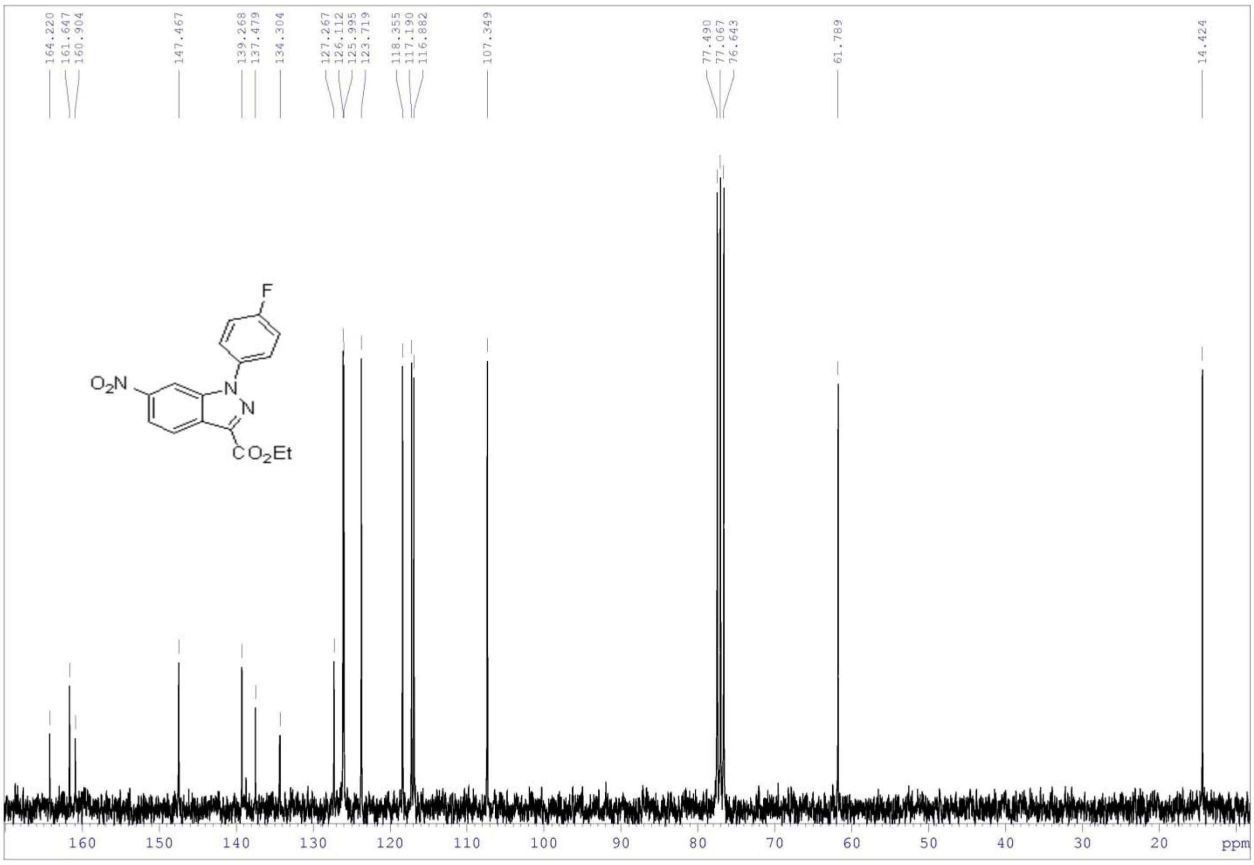

HRMS spectrum of compound 8a

Display Report

Analysis Info

Analysis Name	D:\Data\Kolotyrkina\2022\Bastrakov\0705022.d	Acquisition Date	05.07.2022 15:23:31
Method	tune_50-1600_pos_15_12.m	Operator	BDAL@DE
Sample Name	/LPIK VN-572	Instrument / Ser#	micrOTOF 10248
Comment	C16H12FN3O4 mH 330.0884 calibrant added CH3CN		

Acquisition Parameter

Source Type	ESI	Ion Polarity	Positive	Set Nebulizer	0.4 Bar
Focus	Not active			Set Dry Heater	180 °C
Scan Begin	50 m/z	Set Capillary	4500 V	Set Dry Gas	4.0 l/min
Scan End	1600 m/z	Set End Plate Offset	-500 V	Set Divert Valve	Waste

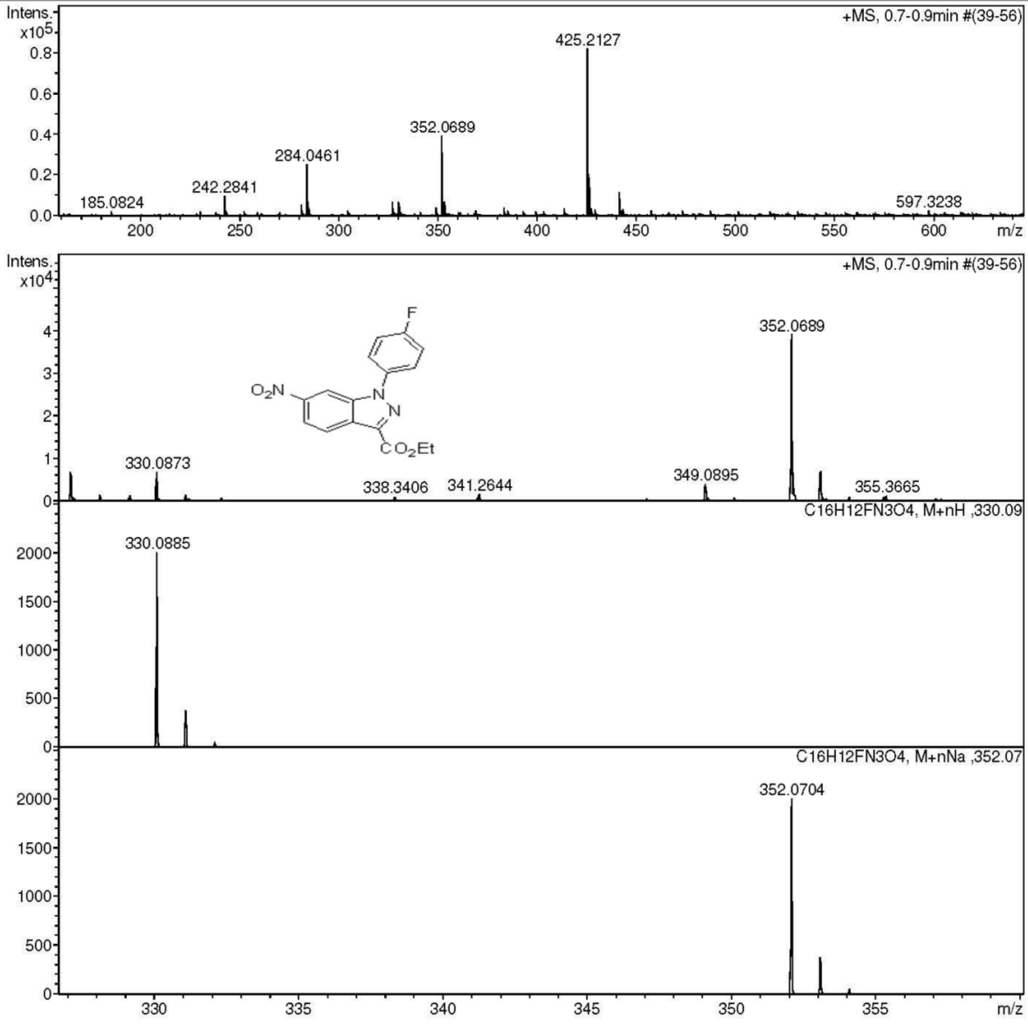

^{1}H and ^{13}C NMR spectra of compound **8b** in $CDCl_3$

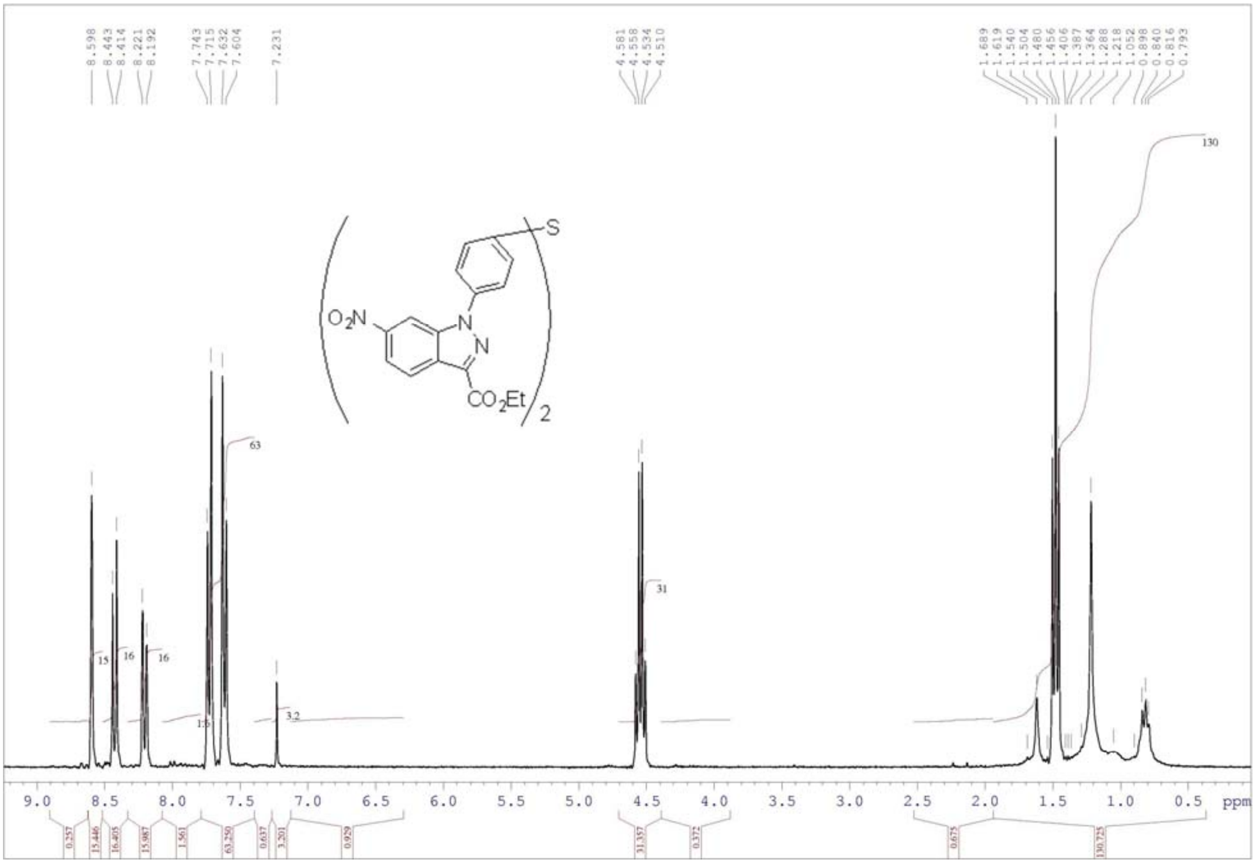

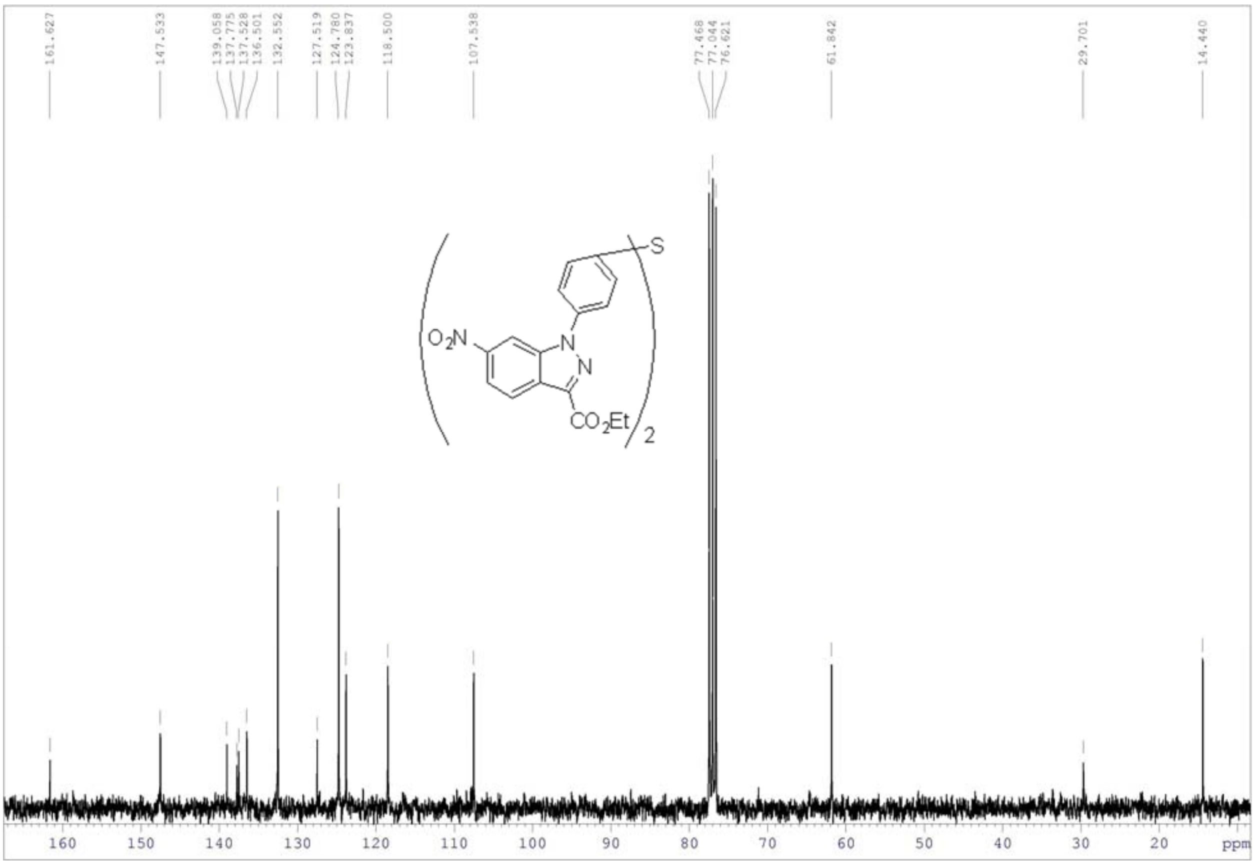

HRMS spectrum of compound 8b

Display Report

Analysis Info

Analysis Name	D:\Data\Kolotyrkina\2022\Bastrakov\1221011.d	Acquisition Date	21.12.2022 10:30:08
Method	tune_low.m	Operator	BDAL@DE
Sample Name	/LPIK VN-584	Instrument / Ser#	micrOTOF 10248
Comment	C32H24N6O8S mH 653.1449 calibrant added CH3CN		

Acquisition Parameter

Source Type	ESI	Ion Polarity	Positive	Set Nebulizer	0.4 Bar
Focus	Not active			Set Dry Heater	180 °C
Scan Begin	50 m/z	Set Capillary	4500 V	Set Dry Gas	4.0 l/min
Scan End	2500 m/z	Set End Plate Offset	-500 V	Set Divert Valve	Waste

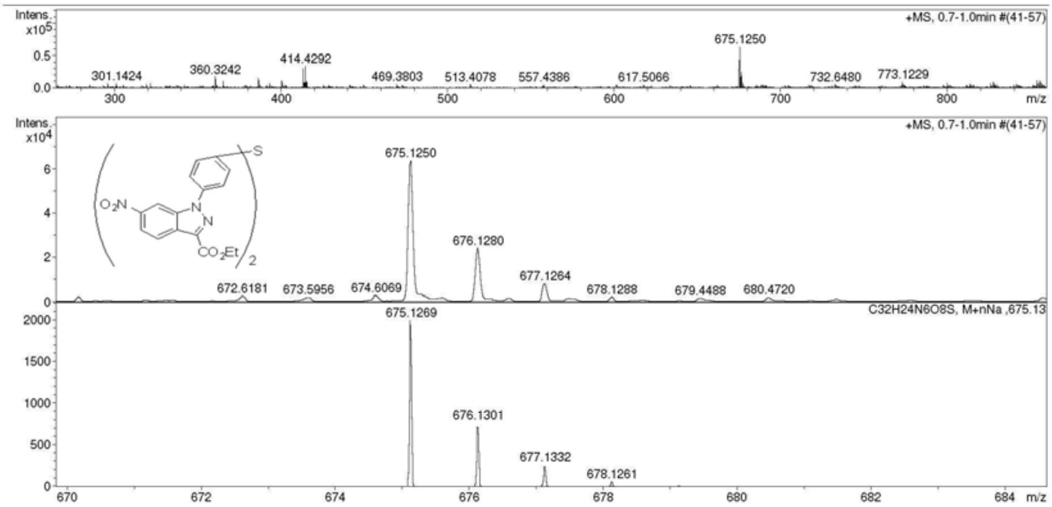

^{1}H and ^{13}C NMR spectra of compound **8c** in $CDCl_3$

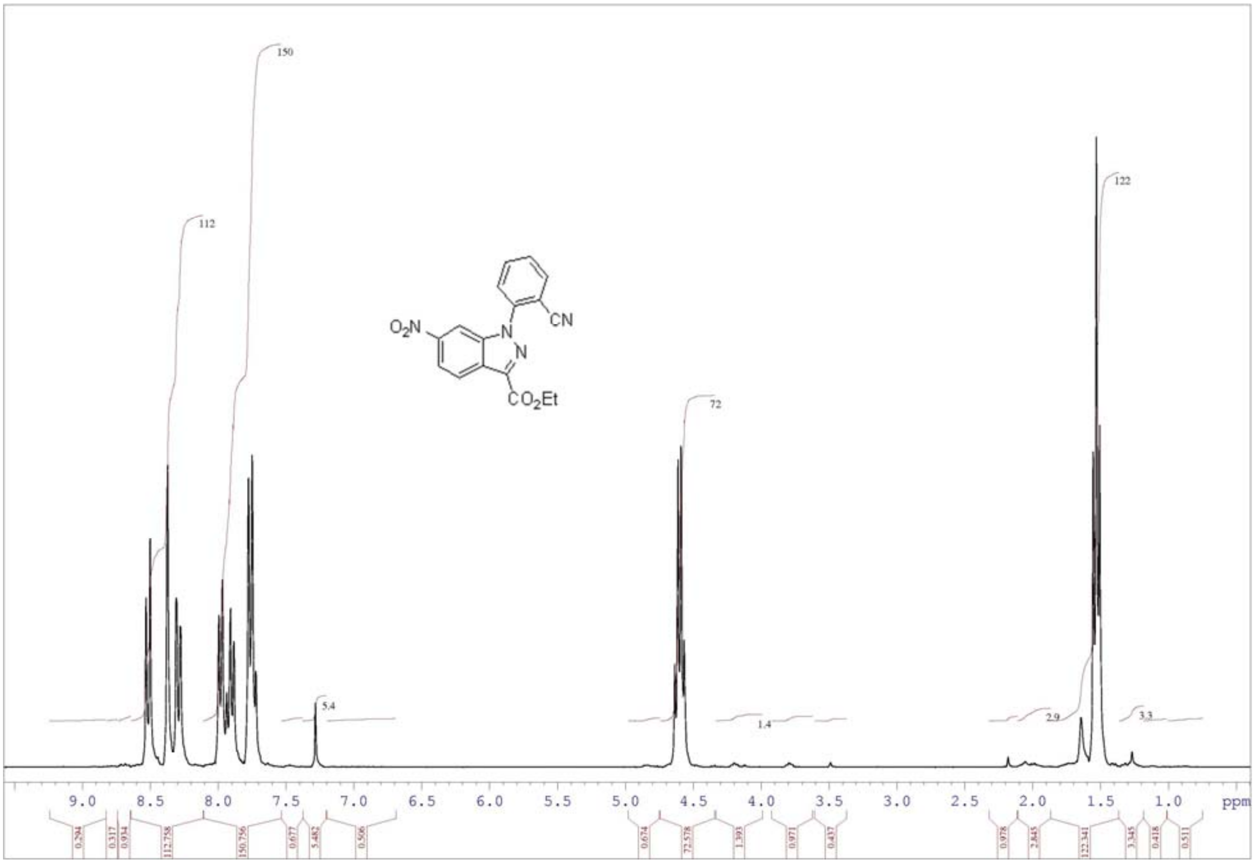

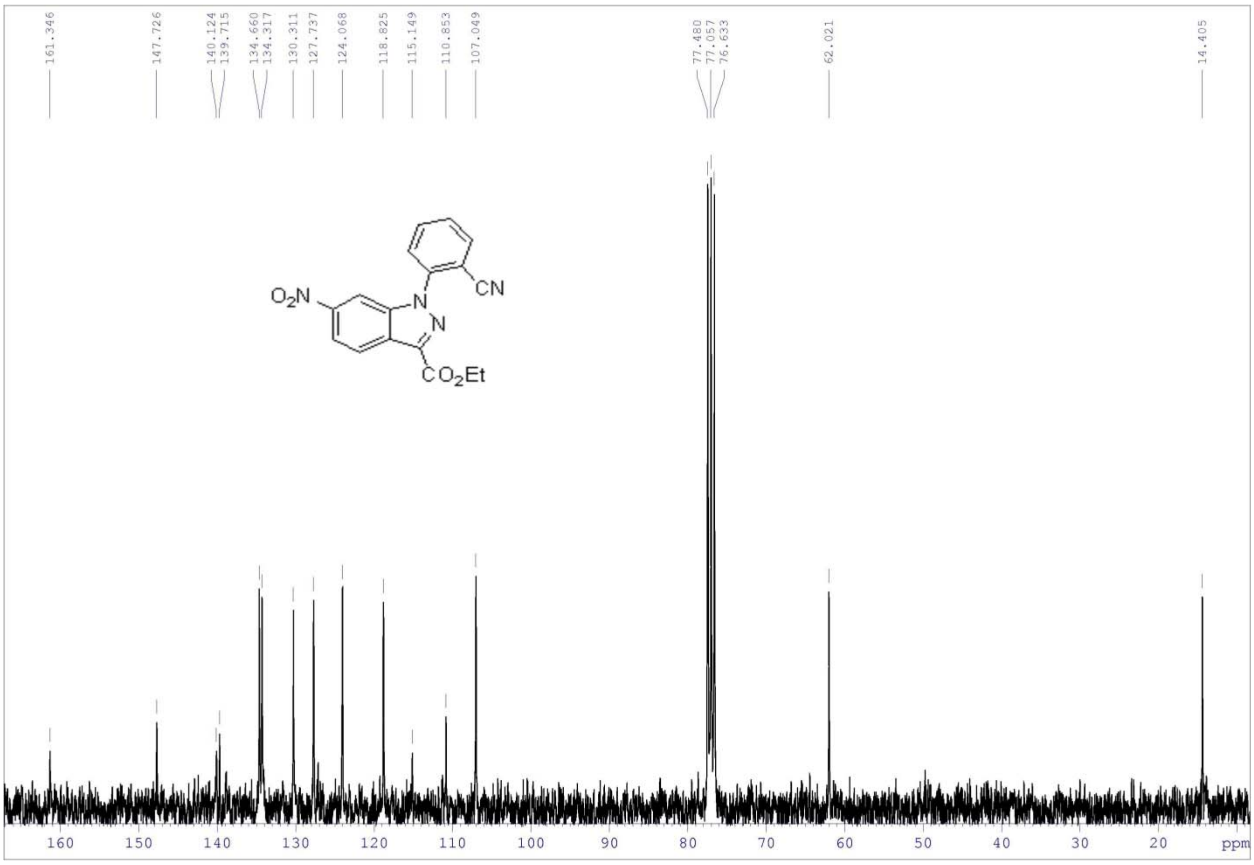

HRMS spectrum of compound 8c

Display Report

Analysis Info

Analysis Name	D:\Data\Kolotyrkina\2022\Bastrakov\1221012.d	Acquisition Date	21.12.2022 10:40:15
Method	tune_low.m	Operator	BDAL@DE
Sample Name	/LPIK VN-605	Instrument / Ser#	micrOTOF 10248
Comment	C17H12N4O4 mH 337.0931 calibrant added CH3CN		

Acquisition Parameter

Source Type	ESI	Ion Polarity	Positive	Set Nebulizer	0.4 Bar
Focus	Not active			Set Dry Heater	180 °C
Scan Begin	50 m/z	Set Capillary	4500 V	Set Dry Gas	4.0 l/min
Scan End	2500 m/z	Set End Plate Offset	-500 V	Set Divert Valve	Waste

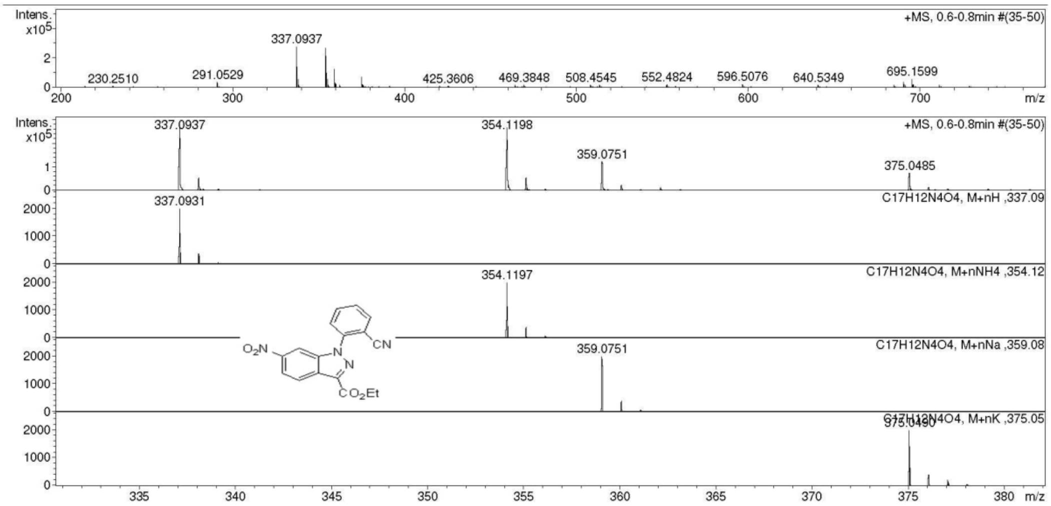

^{1}H and spectrum of compound **8d** in DMSO-d_6

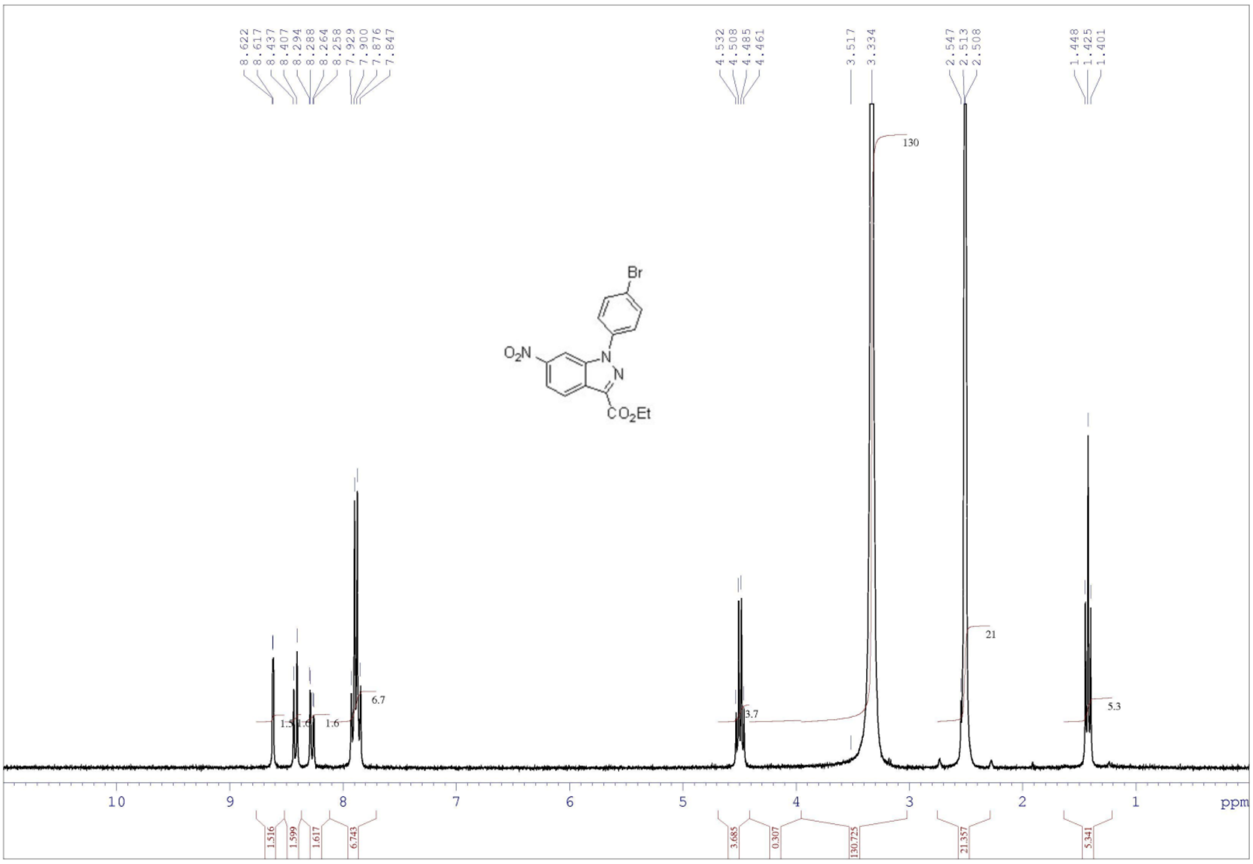

HRMS spectrum of compound 8d

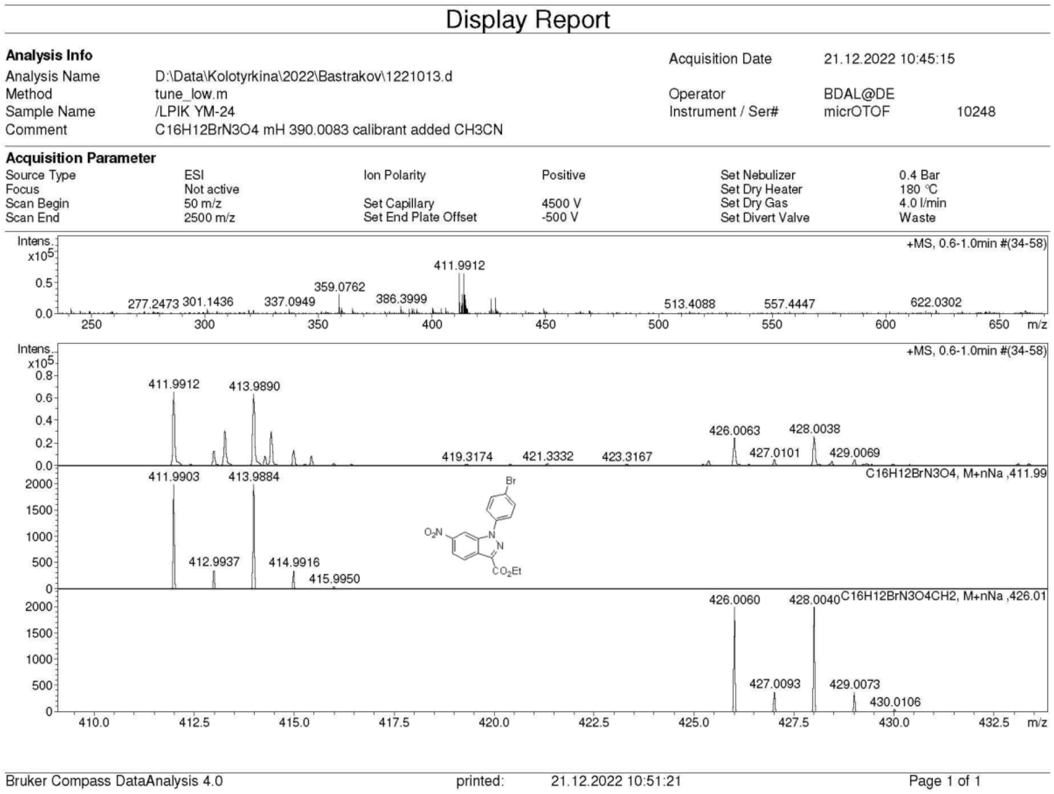

^{1}H and ^{13}C NMR spectra of compound **8e** in $CDCl_3$

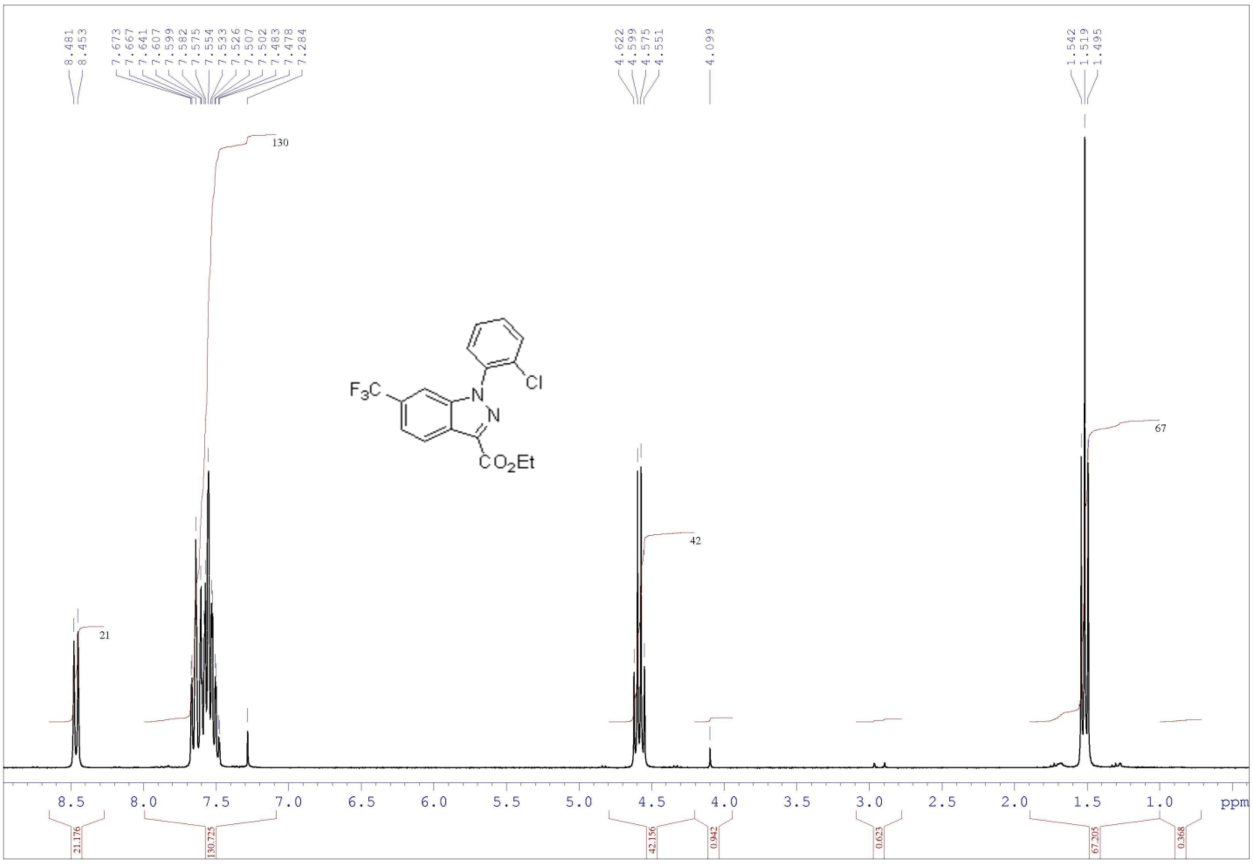

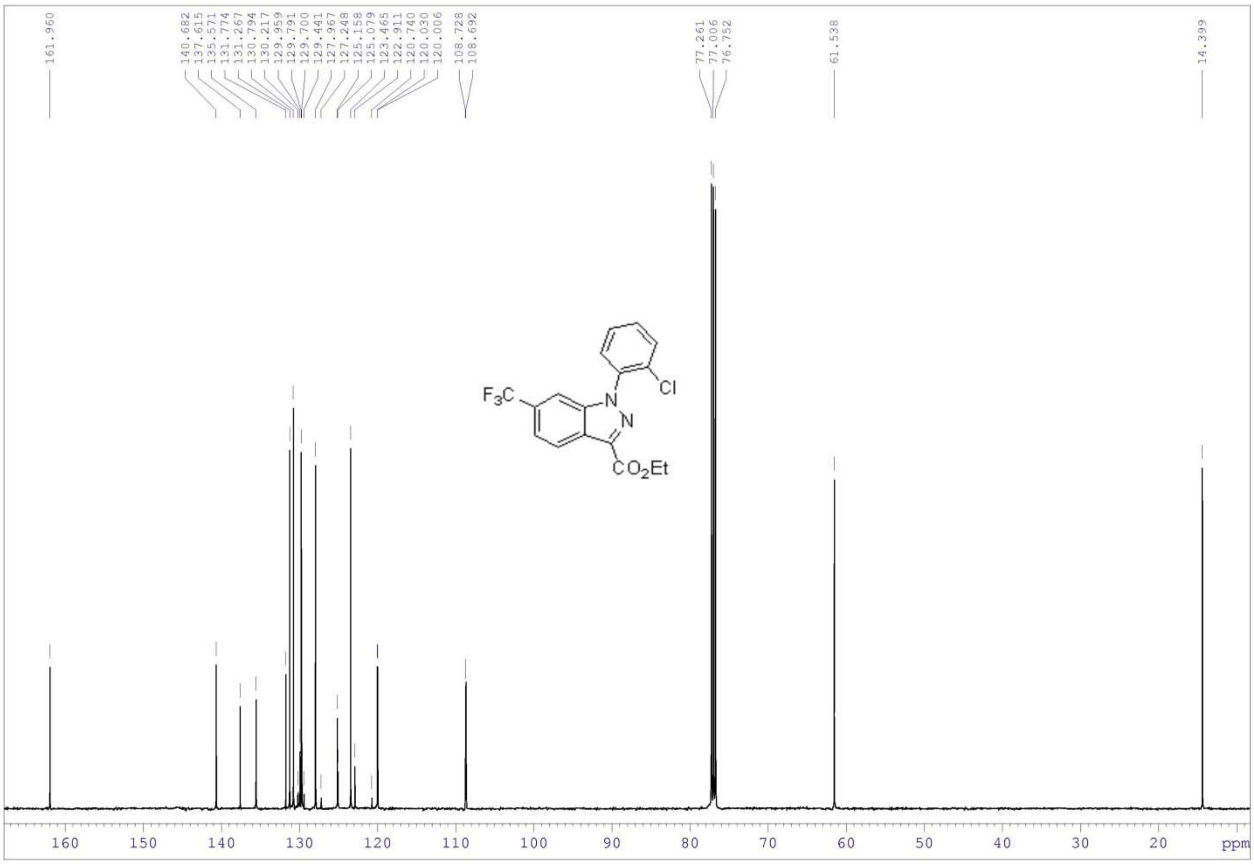

HRMS spectrum of compound 8e

Display Report

Analysis Info

Analysis Name	D:\Data\Kolotyrkina\2022\Bastrakov\1221030.d	Acquisition Date	21.12.2022 14:49:14
Method	tune_low.m	Operator	BDAL@DE
Sample Name	/LPIK VN-617	Instrument / Ser#	micrOTOF 10248
Comment	C17H12ClF3N2O2 mH 369.0612 calibrant added CH3CN		

Acquisition Parameter

Source Type	ESI	Ion Polarity	Positive	Set Nebulizer	0.4 Bar
Focus	Not active			Set Dry Heater	180 °C
Scan Begin	50 m/z	Set Capillary	4500 V	Set Dry Gas	4.0 l/min
Scan End	2500 m/z	Set End Plate Offset	-500 V	Set Divert Valve	Waste

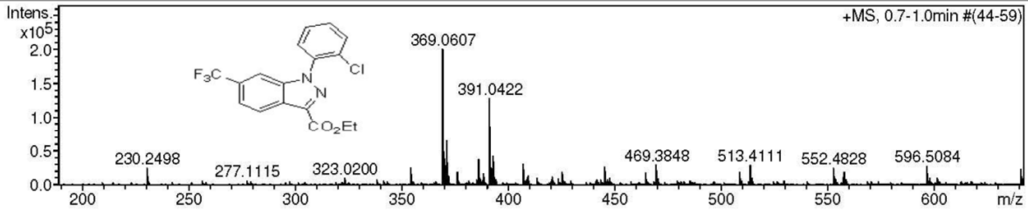

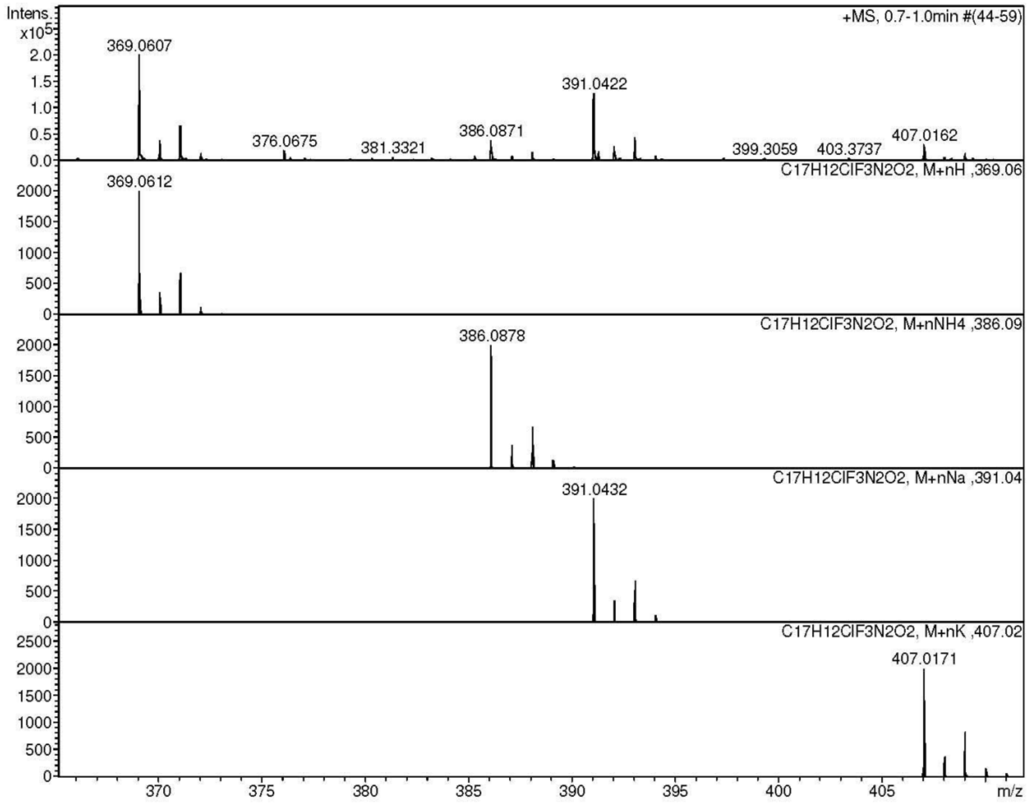

^{1}H and ^{13}C NMR spectra of compound **8f** in $CDCl_3$

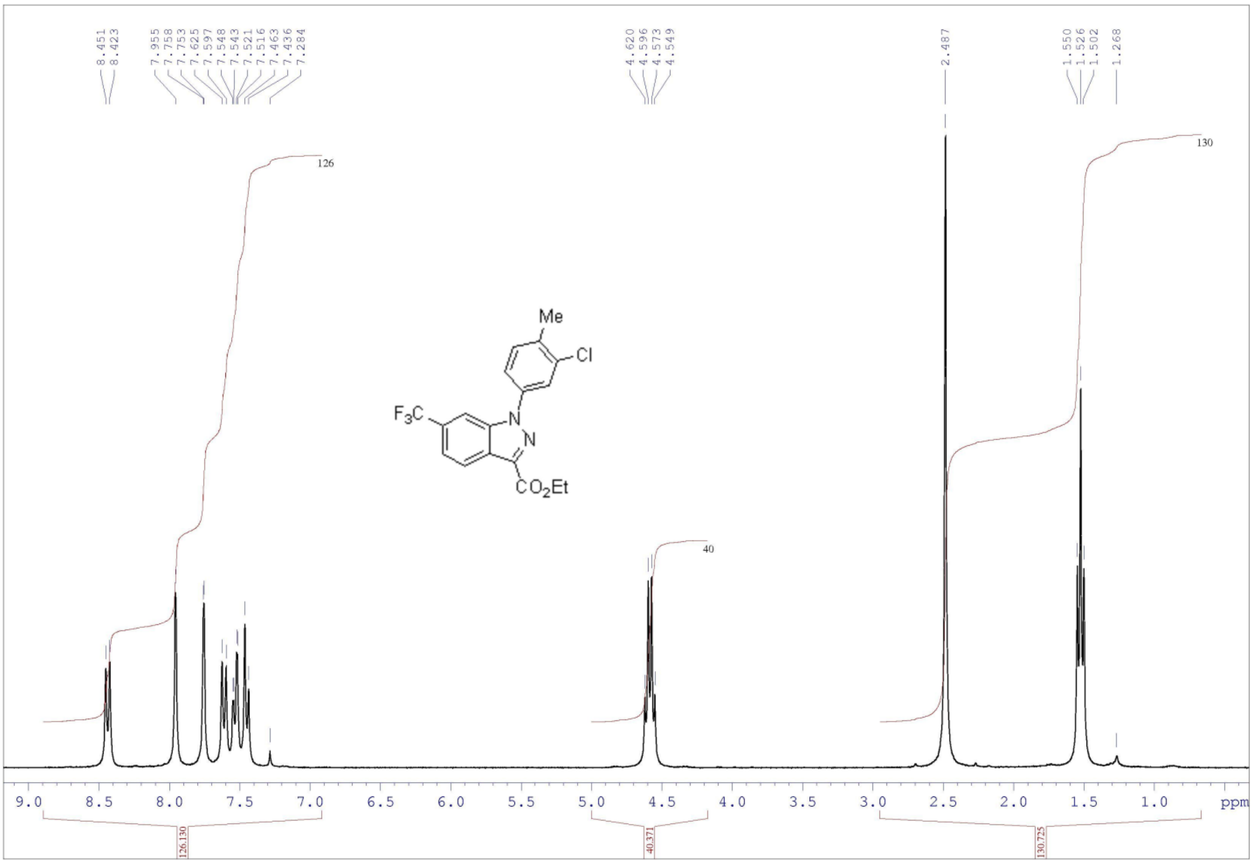

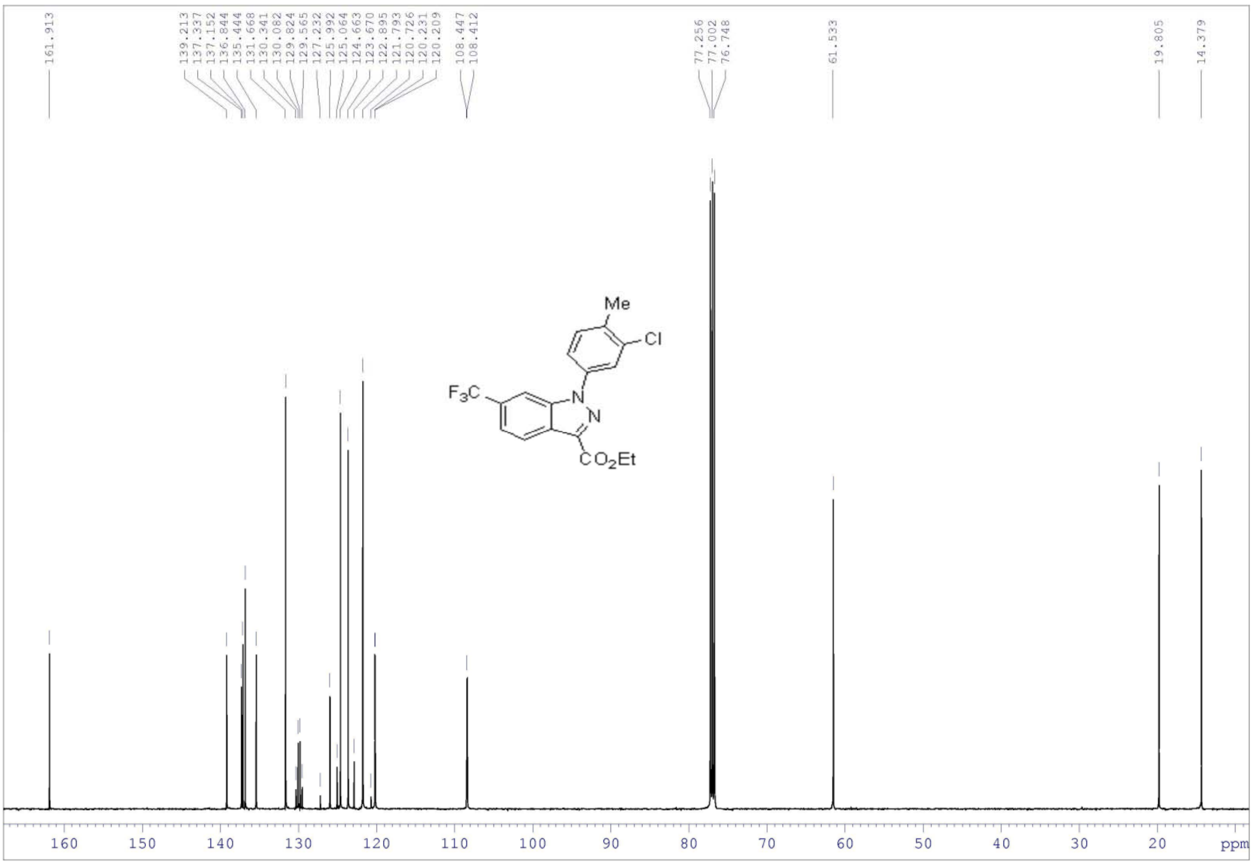

HRMS spectrum of compound 8f

Display Report

Analysis Info

Analysis Name	D:\Data\Kolotyrkina\2022\Bastrakov\1221031.d	Acquisition Date	21.12.2022 14:53:49
Method	tune_low.m	Operator	BDAL@DE
Sample Name	/LPIK YM-18	Instrument / Ser#	micrOTOF 10248
Comment	C18H14ClF3N2O2 mH 383.0768 calibrant added CH3CN		

Acquisition Parameter

Source Type	ESI	Ion Polarity	Positive	Set Nebulizer	0.4 Bar
Focus	Not active			Set Dry Heater	180 °C
Scan Begin	50 m/z	Set Capillary	4500 V	Set Dry Gas	4.0 l/min
Scan End	2500 m/z	Set End Plate Offset	-500 V	Set Divert Valve	Waste

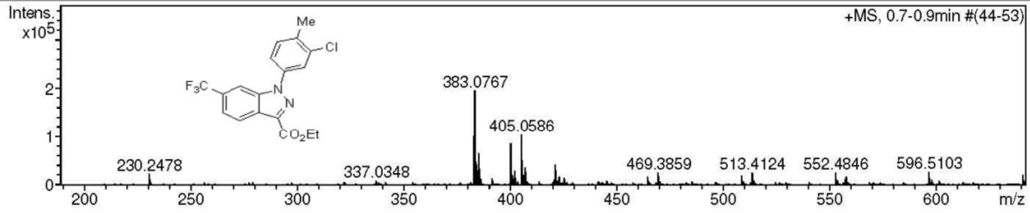

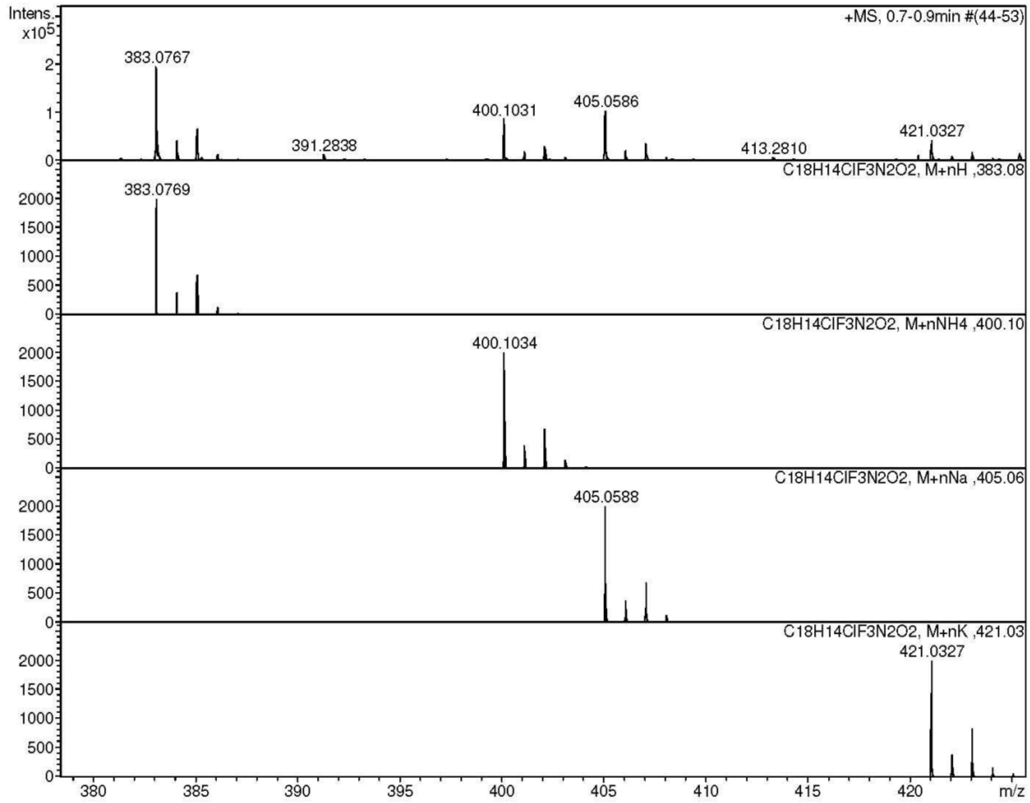

^{1}H and ^{13}C NMR spectra of compound **8g** in $CDCl_3$

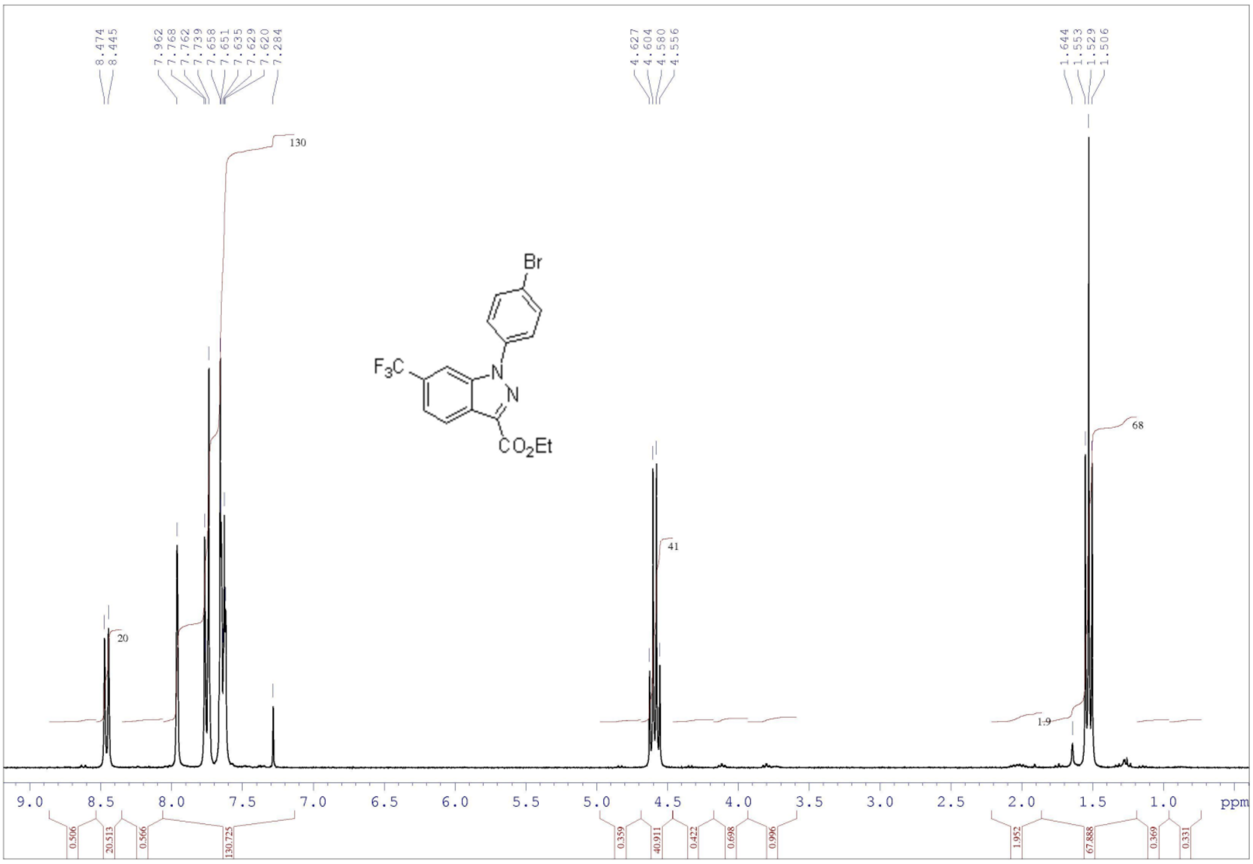

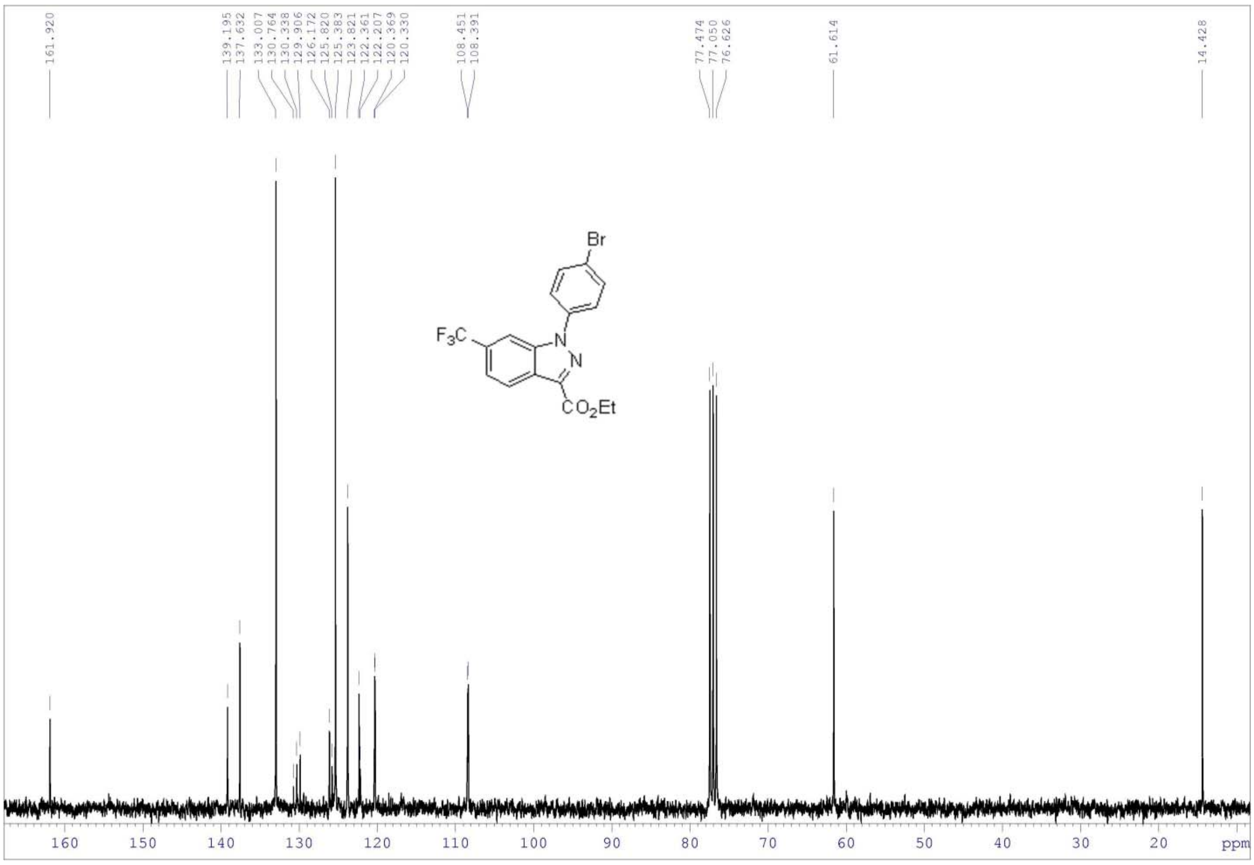

HRMS spectrum of compound 8g

Display Report

Analysis Info

Analysis Name	D:\Data\Kolotyrkina\2022\Bastrakov\1221032.d	Acquisition Date	21.12.2022 14:58:24
Method	tune_low.m	Operator	BDAL@DE
Sample Name	/LPIK VN-642	Instrument / Ser#	micrOTOF 10248
Comment	C17H12BrF3N2O2 mH 413.0107 calibrant added CH3CN		

Acquisition Parameter

Source Type	ESI	Ion Polarity	Positive	Set Nebulizer	0.4 Bar
Focus	Not active			Set Dry Heater	180 °C
Scan Begin	50 m/z	Set Capillary	4500 V	Set Dry Gas	4.0 l/min
Scan End	2500 m/z	Set End Plate Offset	-500 V	Set Divert Valve	Waste

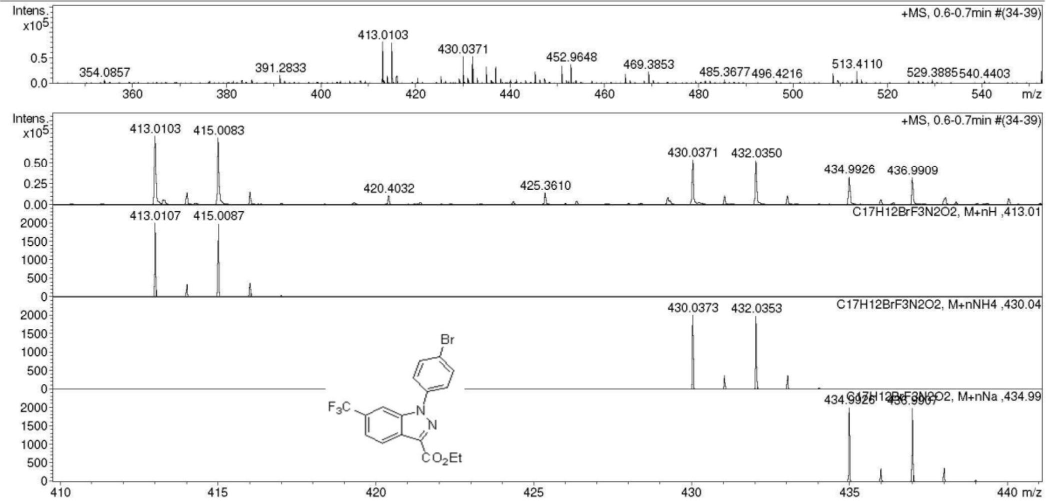

^{1}H and ^{13}C NMR spectra of compound **8h** in $CDCl_3$

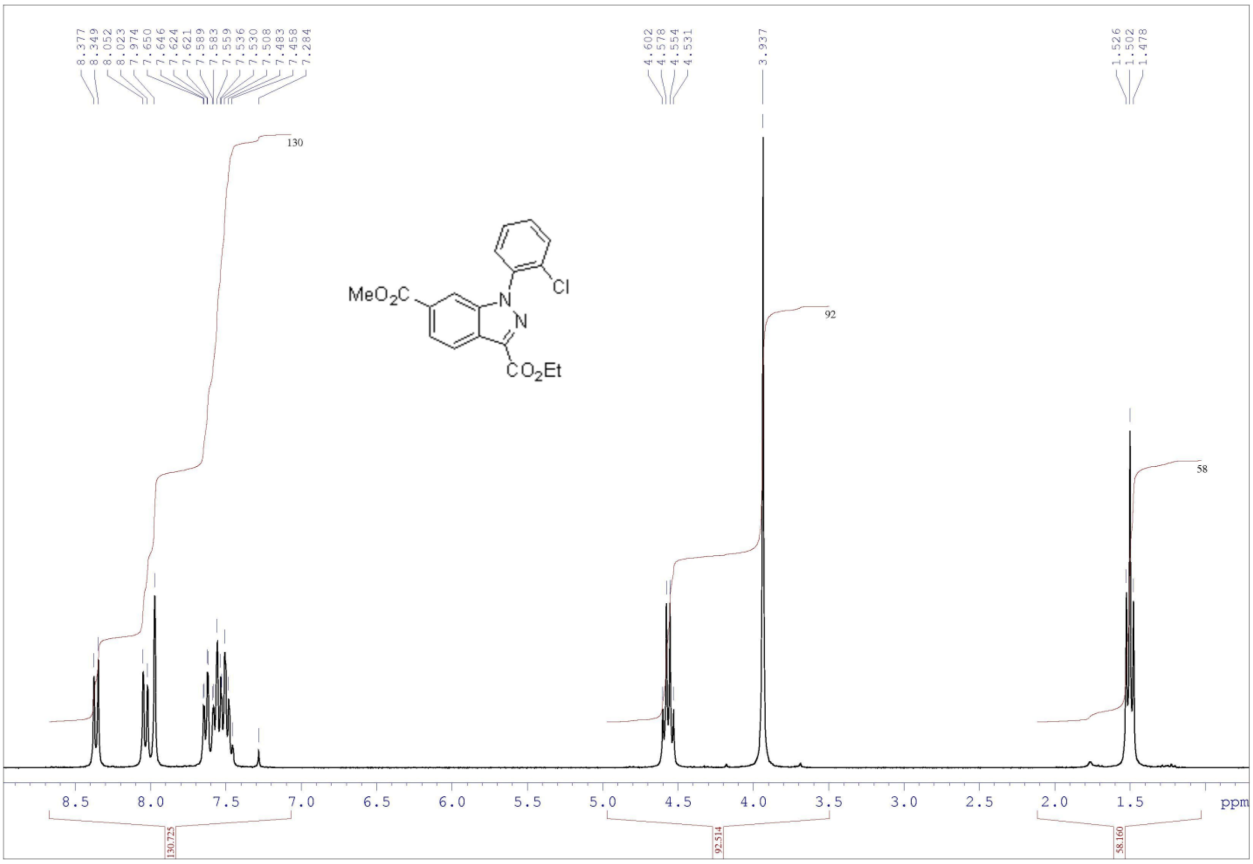

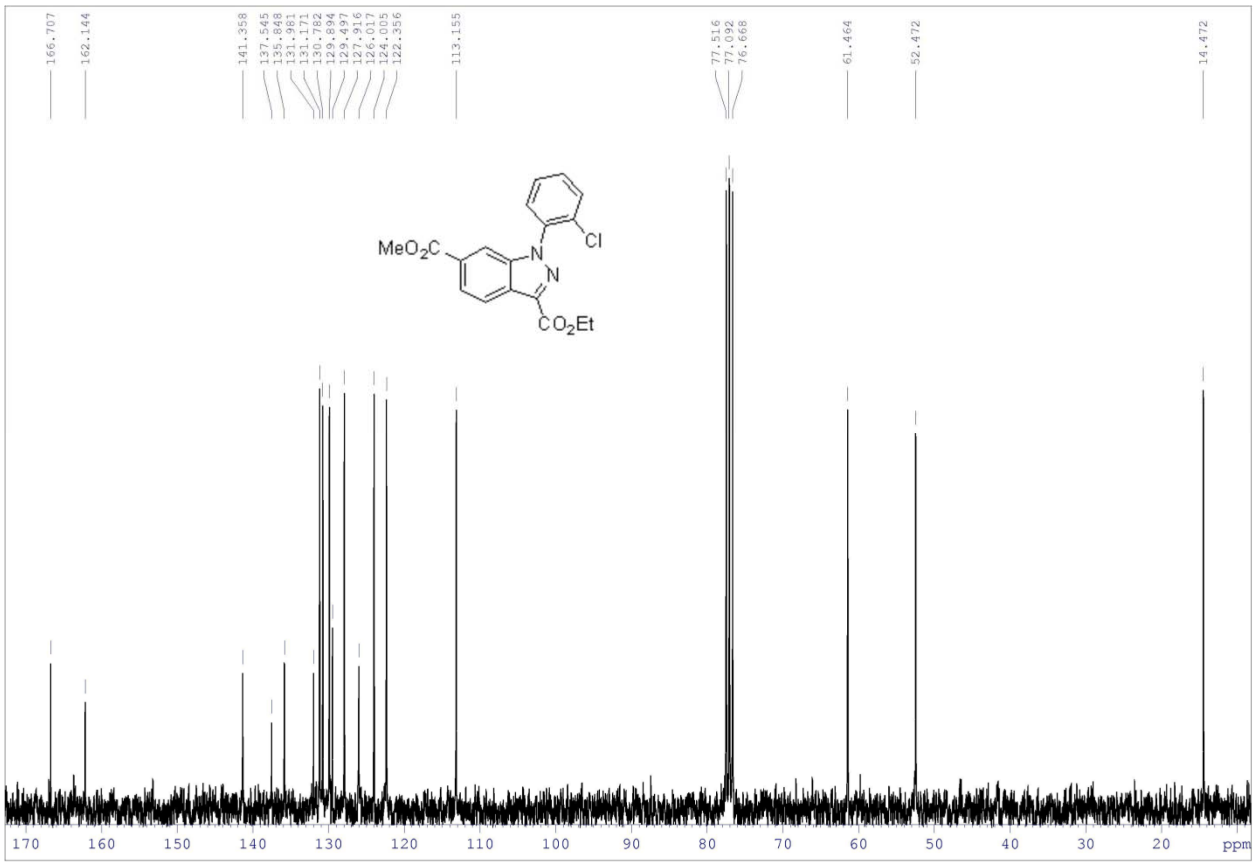

HRMS spectrum of compound 8h

Display Report

Analysis Info

Analysis Name	D:\Data\Kolotyrkina\2022\Bastrakov\1221035.d	Acquisition Date	21.12.2022 15:20:24
Method	tune_low.m	Operator	BDAL@DE
Sample Name	/LPIK YM-21	Instrument / Ser#	micrOTOF 10248
Comment	C18H15ClN2O4 mH 359.0793 calibrant added CH3CN		

Acquisition Parameter

Source Type	ESI	Ion Polarity	Positive	Set Nebulizer	0.4 Bar
Focus	Not active			Set Dry Heater	180 °C
Scan Begin	50 m/z	Set Capillary	4500 V	Set Dry Gas	4.0 l/min
Scan End	2500 m/z	Set End Plate Offset	-500 V	Set Divert Valve	Waste

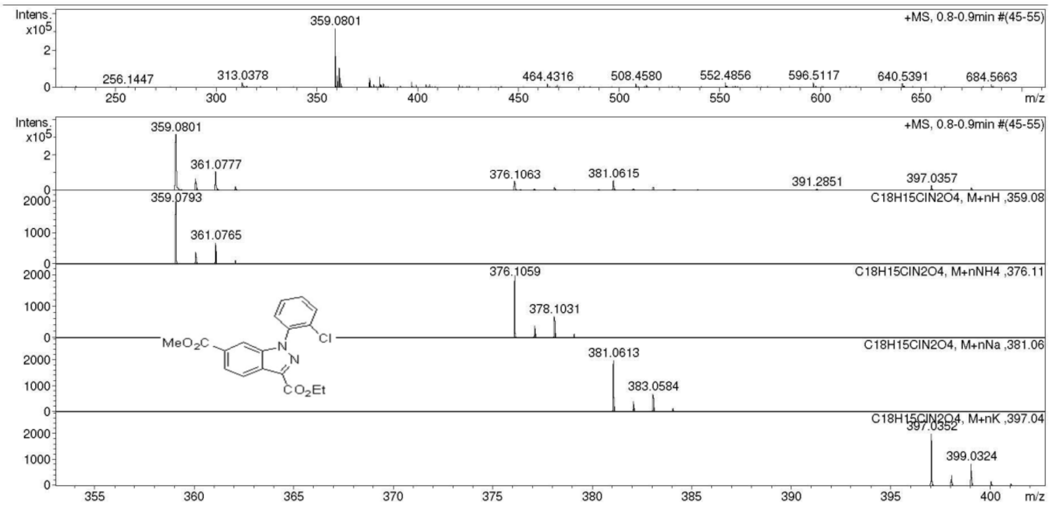

^{1}H and ^{13}C NMR spectra of compound **8i** in $CDCl_3$

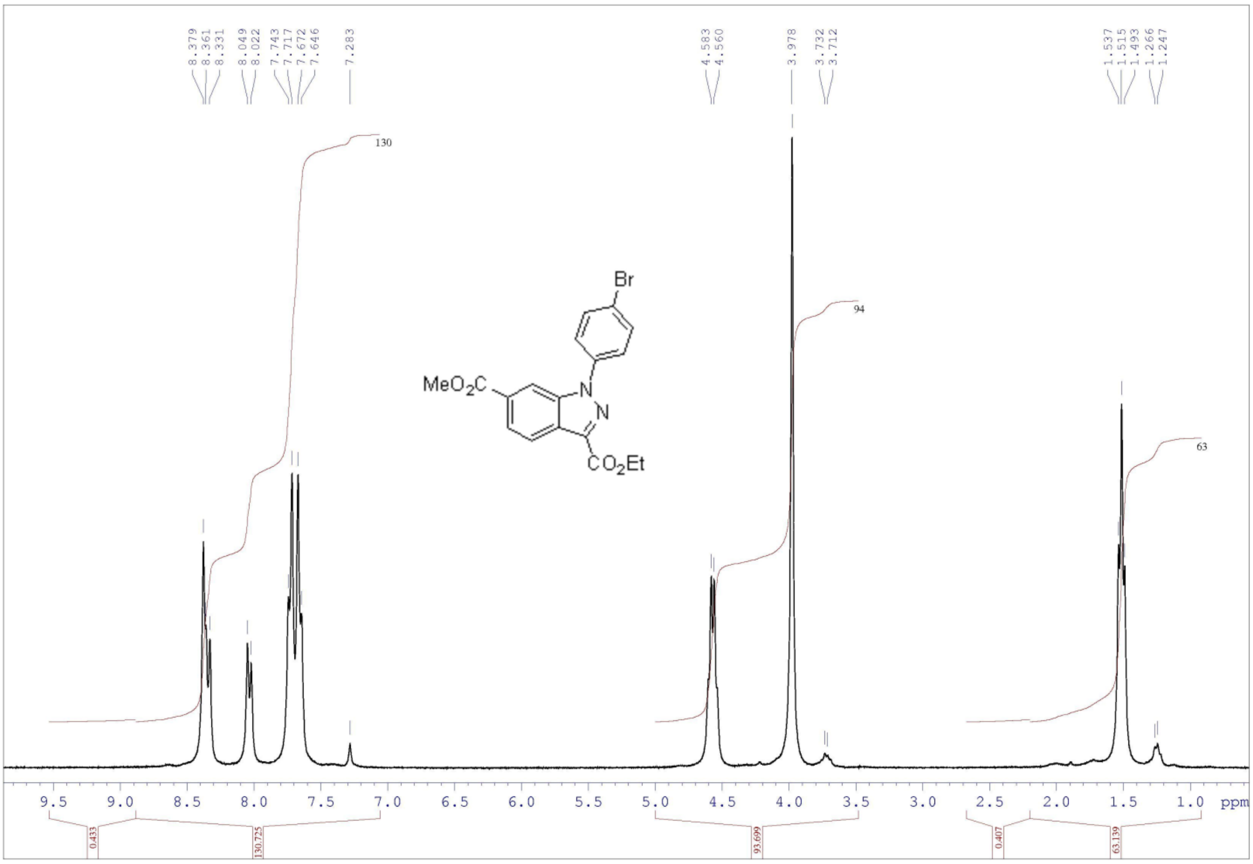

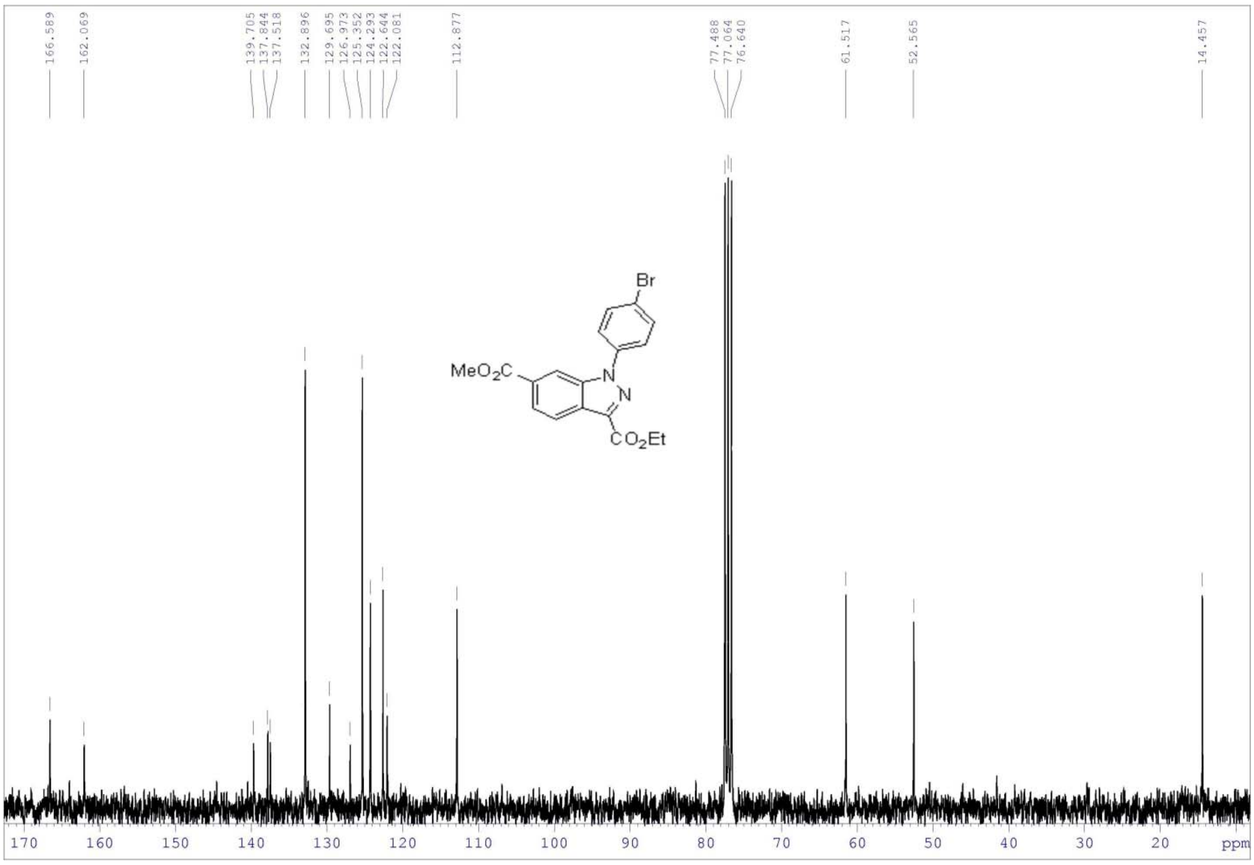

HRMS spectrum of compound 8i

Display Report

Analysis Info

Analysis Name	D:\Data\Kolotyrkina\2022\Bastrakov\1221036.d	Acquisition Date	21.12.2022 15:26:09
Method	tune_low.m	Operator	BDAL@DE
Sample Name	/LPIK YM-27	Instrument / Ser#	micrOTOF 10248
Comment	C18H15BrN2O4 mH 403.0287 calibrant added CH3CN		

Acquisition Parameter

Source Type	ESI	Ion Polarity	Positive	Set Nebulizer	0.4 Bar
Focus	Not active			Set Dry Heater	180 °C
Scan Begin	50 m/z	Set Capillary	4500 V	Set Dry Gas	4.0 l/min
Scan End	2500 m/z	Set End Plate Offset	-500 V	Set Divert Valve	Waste

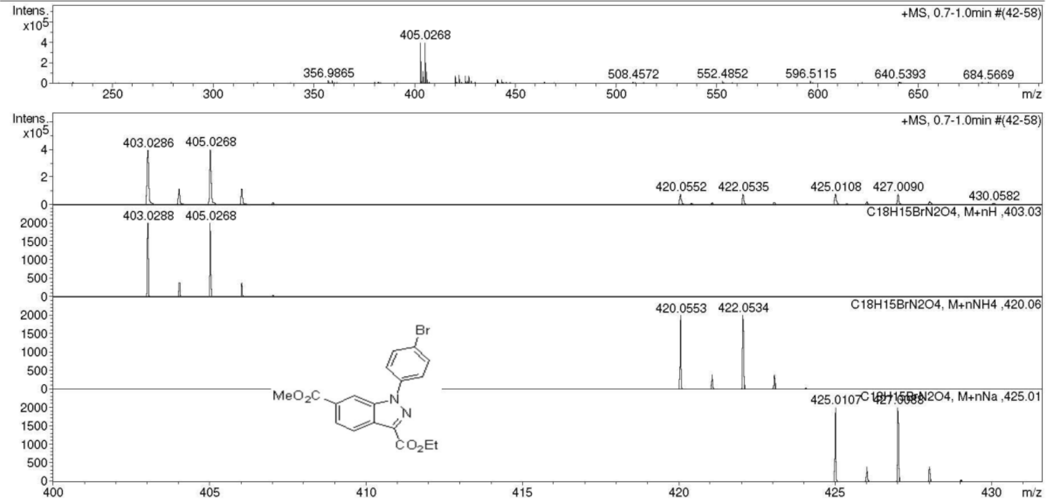

^{1}H and ^{13}C NMR spectra of compound **8j** in $CDCl_3$

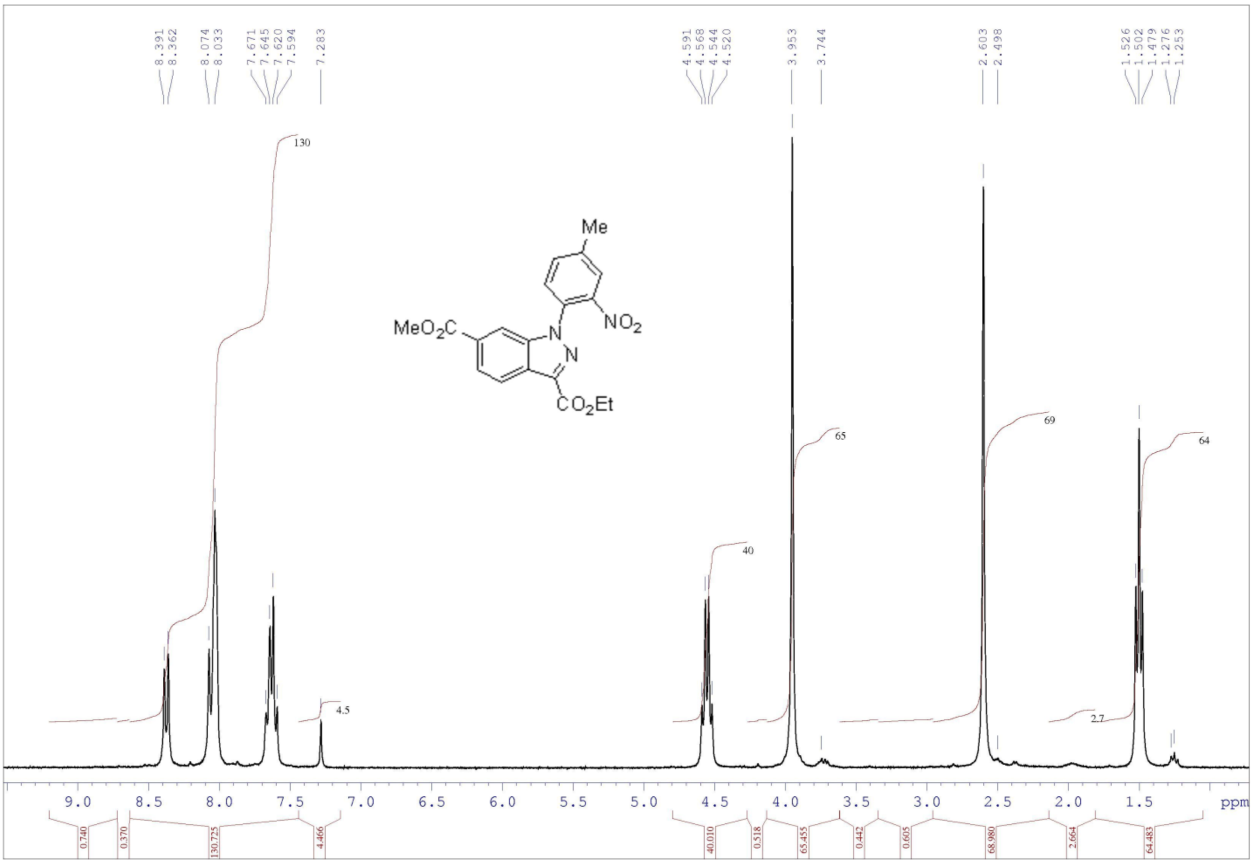

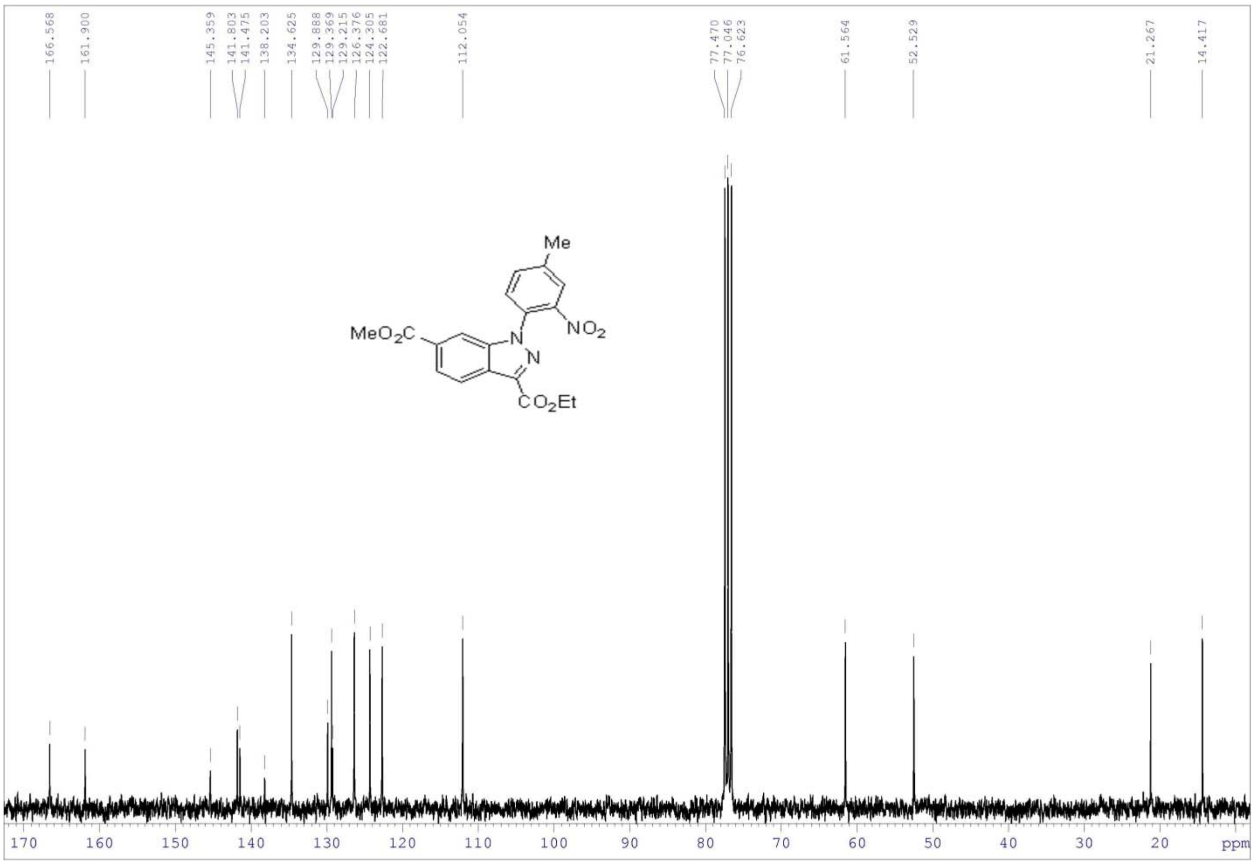

HRMS spectrum of compound 8j

Display Report

Analysis Info

Analysis Name	D:\Data\Kolotyrkina\2022\Bastrakov\1221037.d	Acquisition Date	21.12.2022 15:31:56
Method	tune_low.m	Operator	BDAL@DE
Sample Name	/LPIK YM-28	Instrument / Ser#	micrOTOF 10248
Comment	C19H17N3O6 mH 384.1190 calibrant added CH3CN		

Acquisition Parameter

Source Type	ESI	Ion Polarity	Positive	Set Nebulizer	0.4 Bar
Focus	Not active			Set Dry Heater	180 °C
Scan Begin	50 m/z	Set Capillary	4500 V	Set Dry Gas	4.0 l/min
Scan End	2500 m/z	Set End Plate Offset	-500 V	Set Divert Valve	Waste

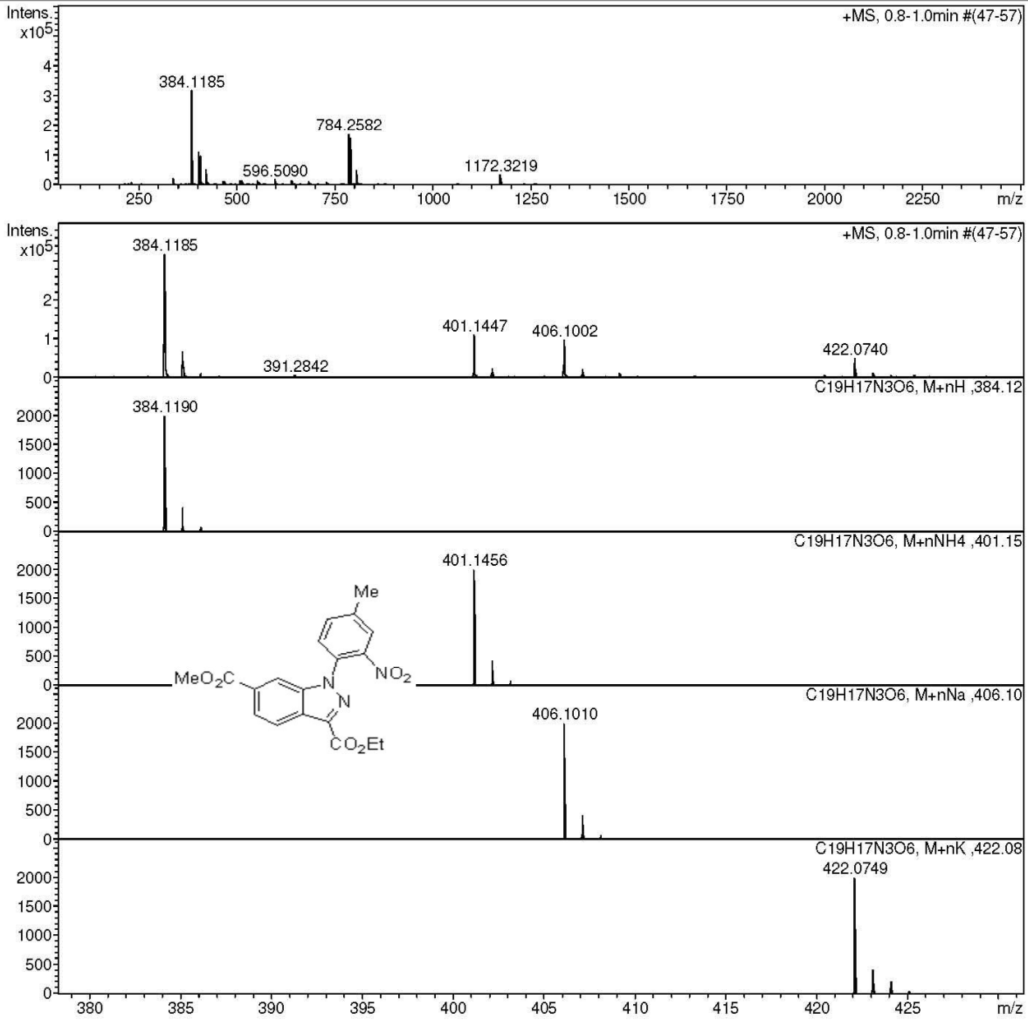

^{1}H and ^{13}C NMR spectra of compound **8k** in $CDCl_3$

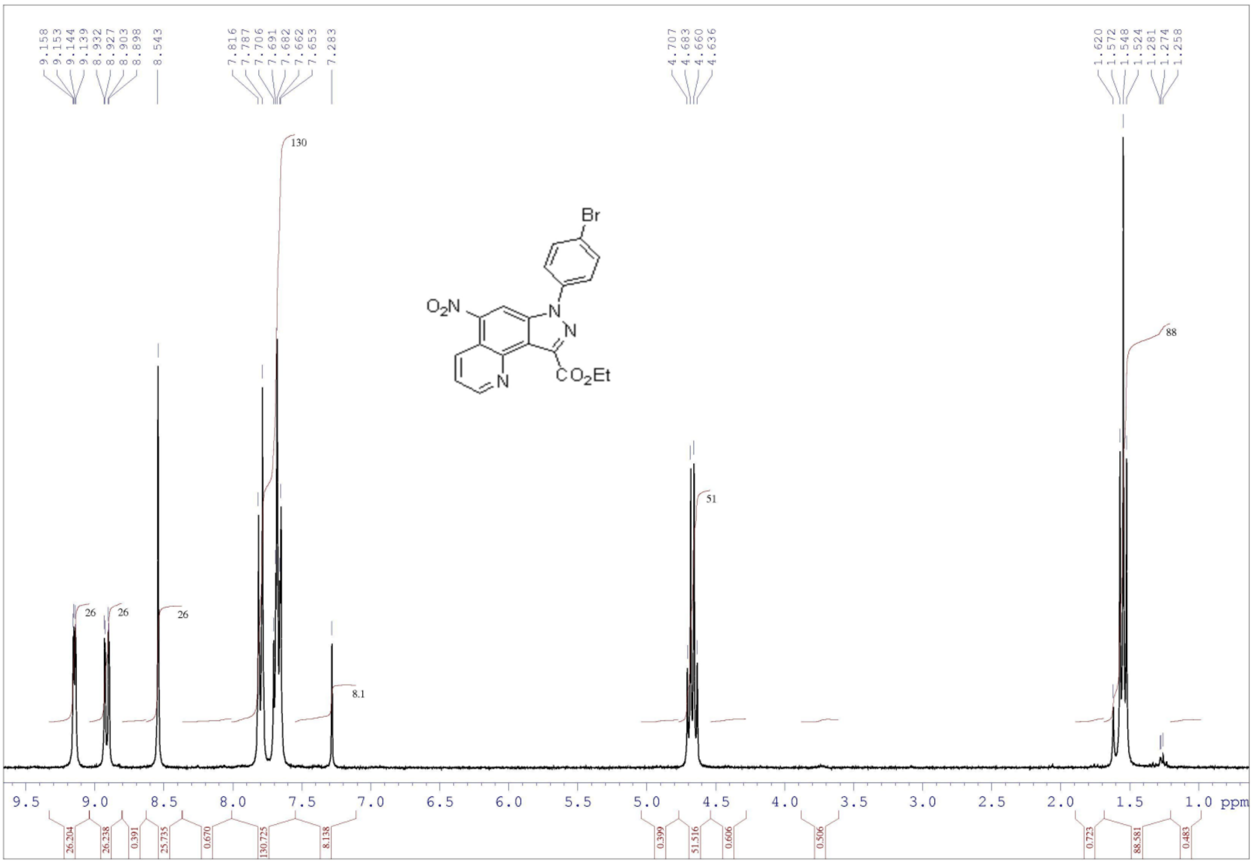

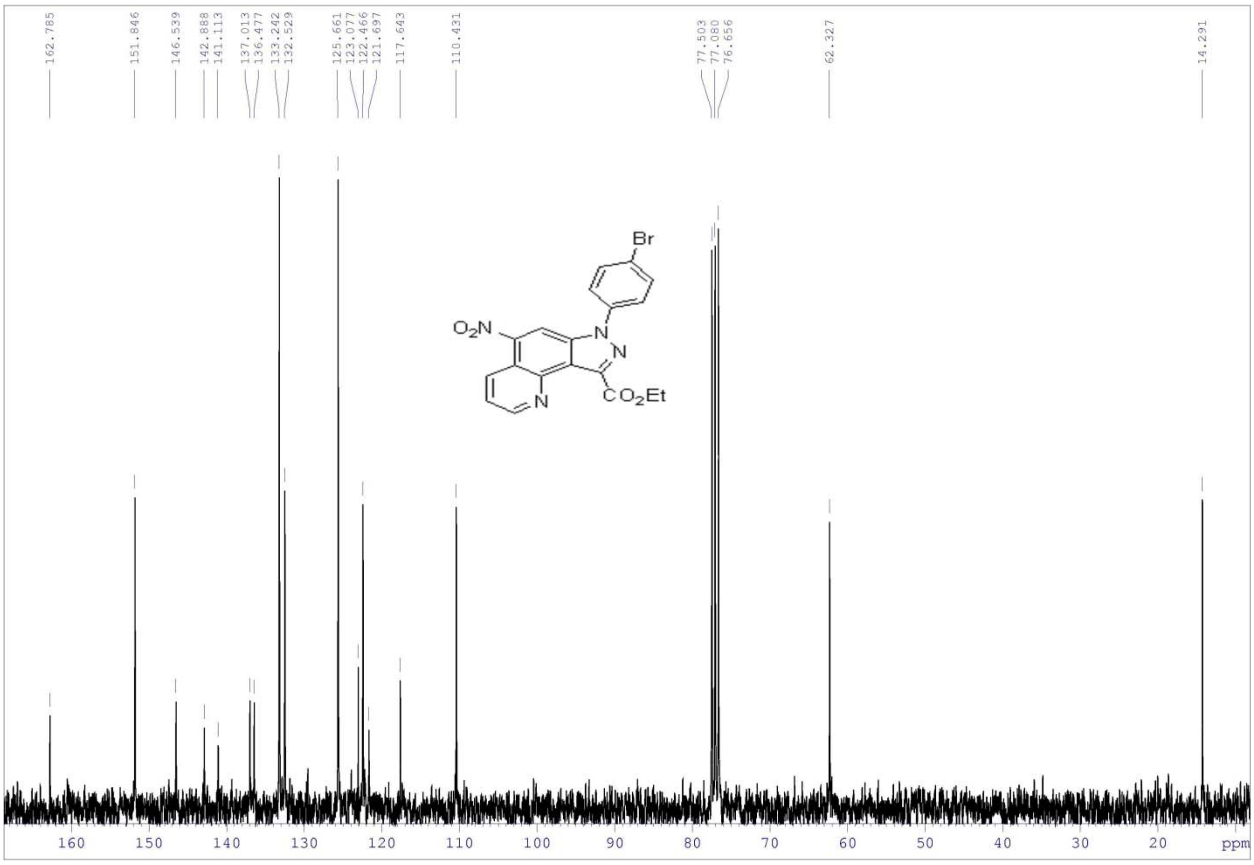

HRMS spectrum of compound 8k

Display Report

Analysis Info

Analysis Name	D:\Data\Kolotyrkina\2022\Bastrakov\1221038.d	Acquisition Date	21.12.2022 15:36:55
Method	tune_low.m	Operator	BDAL@DE
Sample Name	/LPIK YM-32	Instrument / Ser#	micrOTOF 10248
Comment	C19H13BrN4O4 mH 441.0192 calibrant added CH3CN		

Acquisition Parameter

Source Type	ESI	Ion Polarity	Positive	Set Nebulizer	0.4 Bar
Focus	Not active			Set Dry Heater	180 °C
Scan Begin	50 m/z	Set Capillary	4500 V	Set Dry Gas	4.0 l/min
Scan End	2500 m/z	Set End Plate Offset	-500 V	Set Divert Valve	Waste

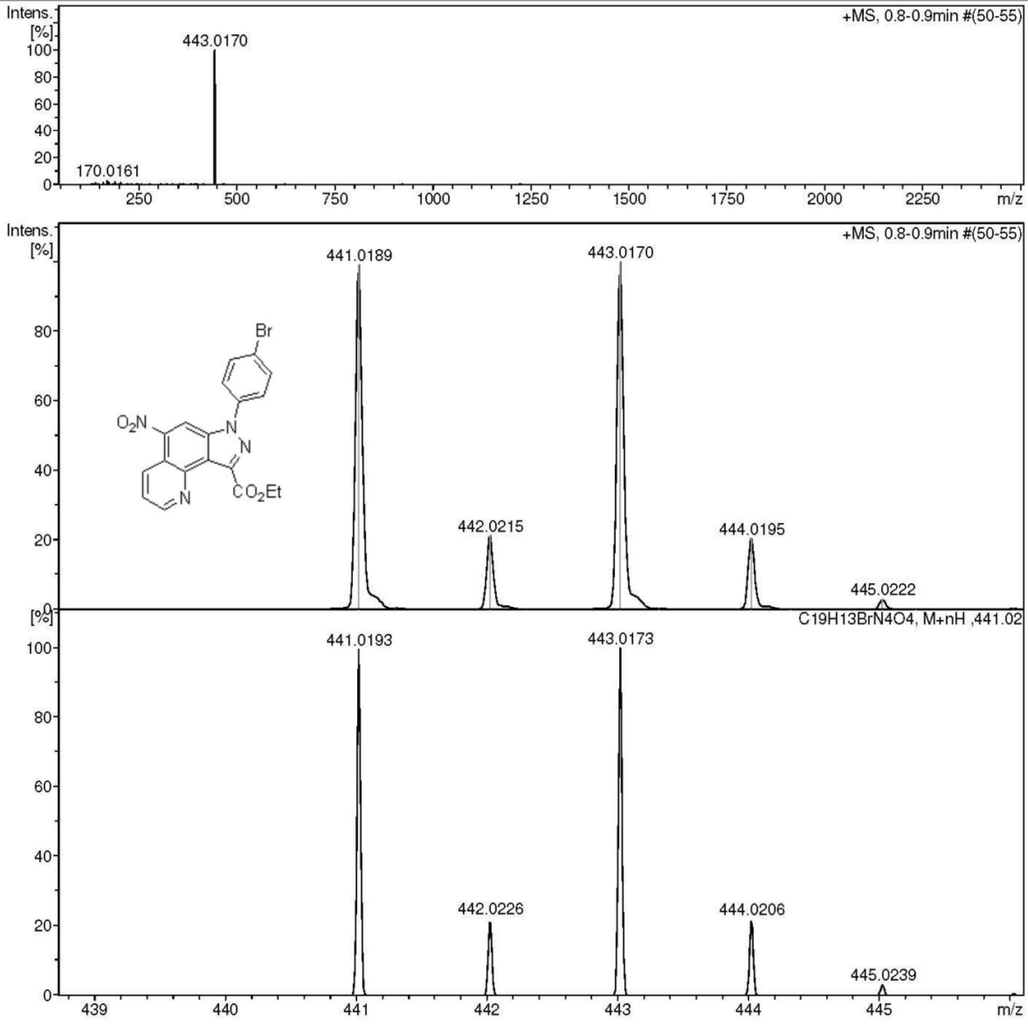

^{1}H and ^{13}C NMR spectra of compound **10e** in $CDCl_3$

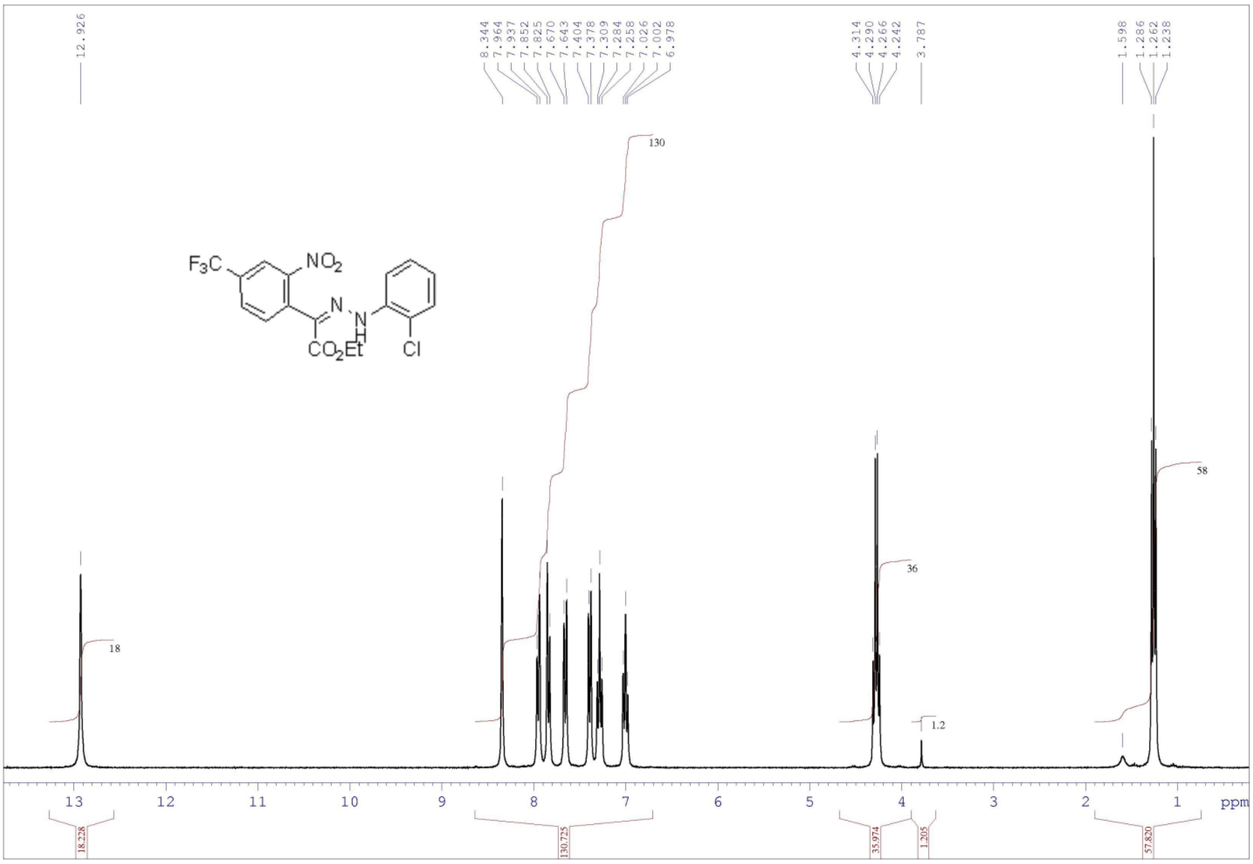

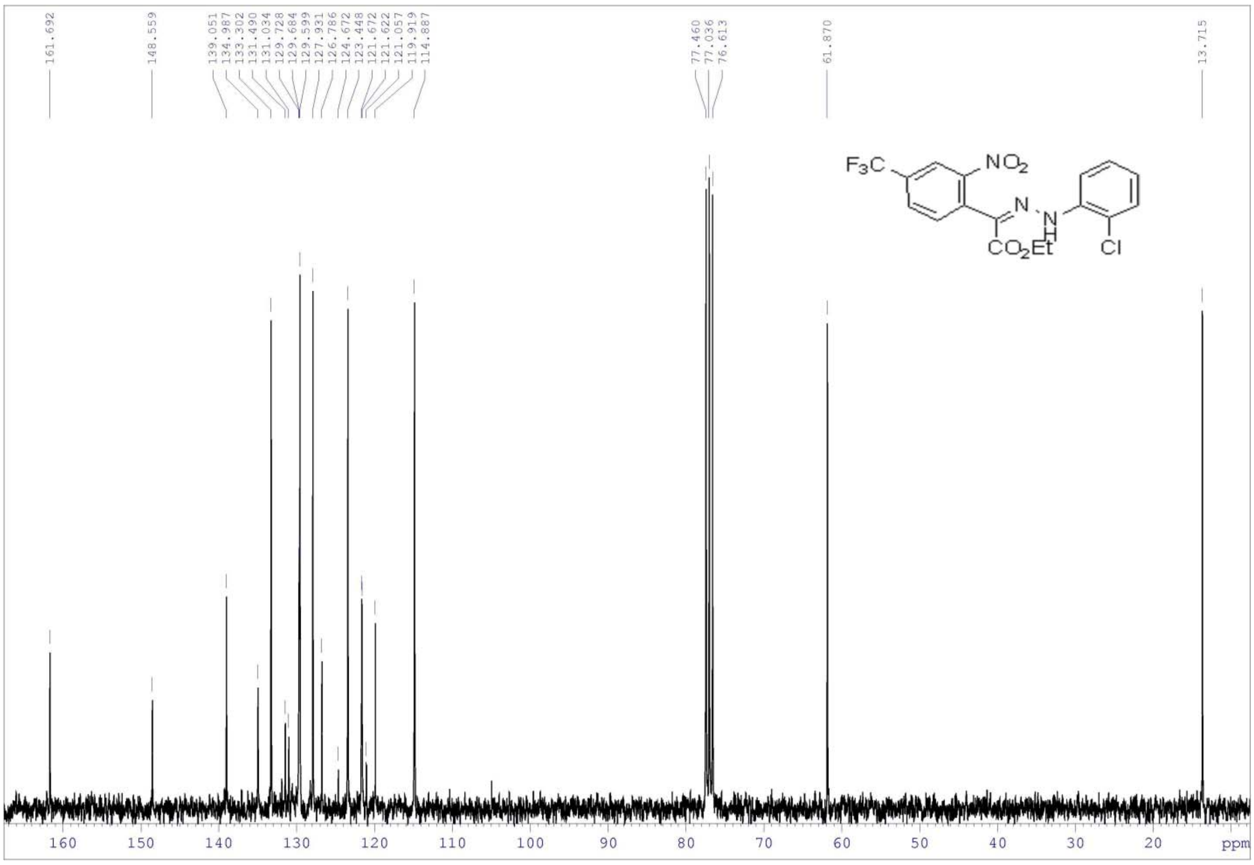

HRMS spectrum of compound 10e

Display Report

Analysis Info

Analysis Name	D:\Data\Kolotyrkina\2022\Nikol'sky\1222009.d	Acquisition Date	22.12.2022 13:42:25
Method	tune_low.m	Operator	BDAL@DE
Sample Name	/LPIK VN-655	Instrument / Ser#	micrOTOF 10248
Comment	C17H13ClF3N3O4 mH 416.0619 calibrant added CH3OH		

Acquisition Parameter

Source Type	ESI	Ion Polarity	Positive	Set Nebulizer	0.4 Bar
Focus	Not active			Set Dry Heater	180 °C
Scan Begin	50 m/z	Set Capillary	4500 V	Set Dry Gas	4.0 l/min
Scan End	2500 m/z	Set End Plate Offset	-500 V	Set Divert Valve	Waste

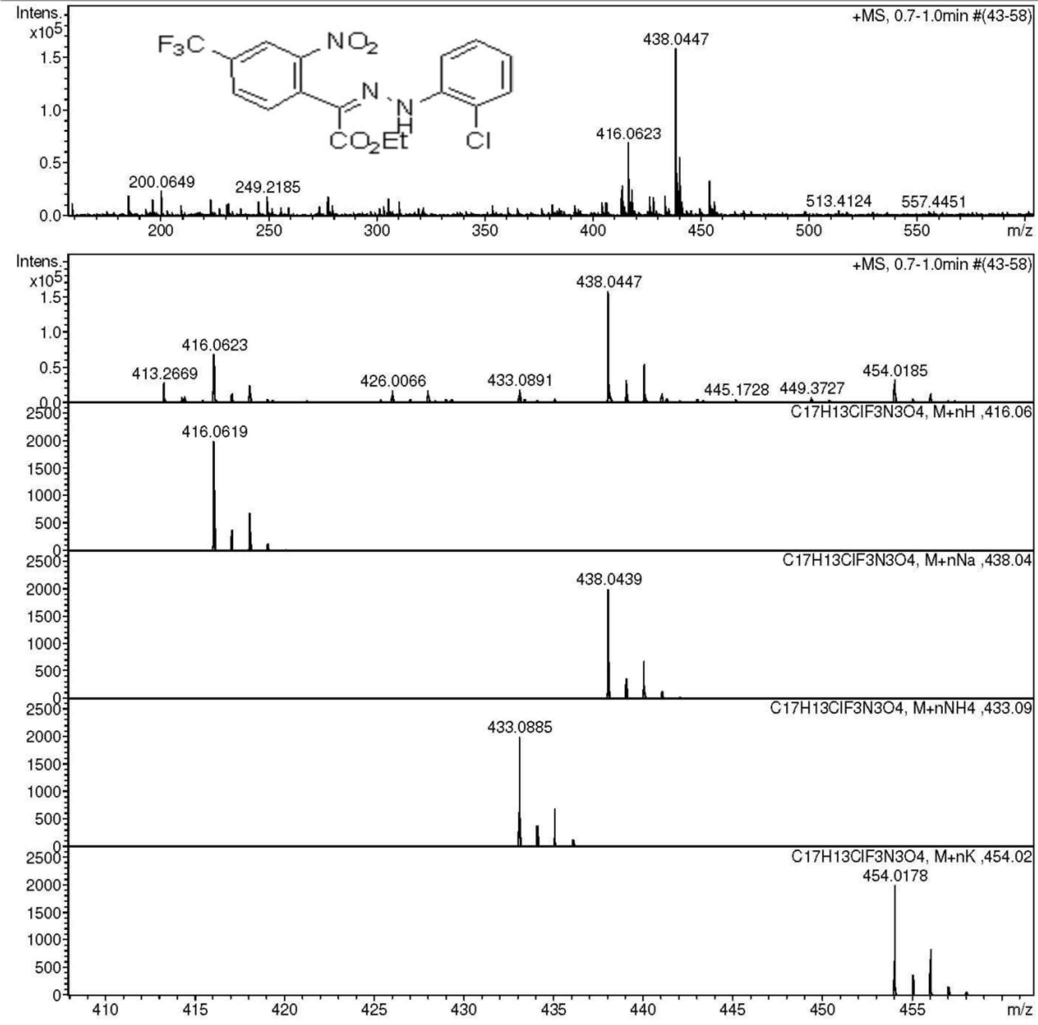

Supplement: Supplementary file 1 [file ijms-24-01758-s001.zip › ijms-2158155-supplementary.pdf]
